# rDNA Clusters Make Contact with Genes that Are Involved in Differentiation and Cancer and Change Contacts after Heat Shock Treatment

**DOI:** 10.3390/cells8111393

**Published:** 2019-11-05

**Authors:** Nickolai A. Tchurikov, Daria M. Fedoseeva, Elena S. Klushevskaya, Ivan Y. Slovohotov, Vladimir R. Chechetkin, Yuri V. Kravatsky, Olga V. Kretova

**Affiliations:** Department of Epigenetic Mechanisms of Gene Expression Regulation, Engelhardt Institute of Molecular Biology Russian Academy of Sciences, 119334 Moscow, Russia; dfedoseeva86@yandex.ru (D.M.F.); giedre@inbox.ru (E.S.K.); ivan_slovohotov@mail.ru (I.Y.S.); vladimir_chechet@mail.ru (V.R.C.); jiri@eimb.ru (Y.V.K.); ovkretova@mail.ru (O.V.K.)

**Keywords:** rDNA clusters, 4C, gene ontology, chromatin marks, epigenetics, development, differentiation, heat shock

## Abstract

Human rDNA clusters form numerous contacts with different chromosomal regions as evidenced by chromosome conformation capture data. Heterochromatization of rDNA genes leads to heterochromatization in different chromosomal regions coupled with the activation of the transcription of genes related to differentiation. These data suggest a role for rDNA clusters in the regulation of many human genes. However, the genes that reside within the rDNA-contacting regions have not been identified. The purpose of this study was to detect and characterize the regions where rDNA clusters make frequent contacts and to identify and categorize genes located in these regions. We analyzed the regions that contact rDNA using 4C data and show that these regions are enriched with genes specifying transcription factors and non-coding RNAs involved in differentiation and development. The rDNA-contacting genes are involved in neuronal development and are associated with different cancers. Heat shock treatment led to dramatic changes in the pattern of rDNA-contacting sites, especially in the regions possessing long stretches of H3K27ac marks. Whole-genome analysis of rDNA-contacting sites revealed specific epigenetic marks and the transcription sites of 20–100 nt non-coding RNAs in these regions. The rDNA-contacting genes jointly regulate many genes that are involved in the control of transcription by RNA polymerase II and the development of neurons. Our data suggest a role for rDNA clusters in the differentiation of human cells and carcinogenesis.

## 1. Introduction

Nucleoli are the largest organelles in nuclei. They are responsible for the massive synthesis of ribosomal RNA that accounts for up to 80% of all cellular RNA production [1]. Microscopically, nucleoli form contacts with both near and distant chromosomal regions [2]. There are five clusters of ribosomal genes (rDNA) in the human genome that are located in the middle of the short arms of the five acrocentric chromosomes 13, 14, 15, 21, and 22 [3]. These clusters shape nucleoli in interphase chromosomes and, thus, are called nucleoli organizing regions (NORs).

It was observed in different organisms that about half of rDNA clusters are silent and form heterochromatin structures that require nucleolar repressor TIP5 factor (TTF1-interacting protein 5), non-coding promoter RNA (pRNA), and PARP1 [4,5,6,7]. The chain of events includes the recruitment of NoRC, an SNF2h-containing remodeling complex, by TIP5 associated with pRNA, which guides the repressor to rDNA genes. These events are followed by deacetylation of histone H4 and the dimethylation of histone H3-Lys9 as well as methylation of rDNA [4,7].

The formation of epigenetically silenced rDNA is associated with heterochromatization of centric and pericentric chromosomal repeats [5]. TTF1-interacting protein 5 is indispensable for the formation of heterochromatin in the centric and pericentric chromosomal regions, although it is unknown whether this is due to the direct interaction of TIP5 with the corresponding chromosomal regions, either by in cis or in trans spreading mechanisms coming from the silenced rDNA clusters or by creating nucleolar/perinucleolar compartments enriched in chromatin repressor complexes. The localized heterochromatin condensation of rDNA clusters initiates the formation of highly condensed chromatin structures outside of the nucleolus, as well as transcriptional activation of a set of differentiation genes [7]. The data strongly suggest the existence of mechanisms by which nucleoli are involved in the epigenetic regulation of different genomic loci.

In order to identify the genomic regions interacting with rDNA clusters, the so-called nucleolar-associated domains were isolated. These domains, which comprise very large (up to 1 Mb) stretches of DNA, were co-purified with the nucleoli preparations in human and plant cells [8,9,10,11]. This low-resolution method demonstrated that between 4% and 38% of the human genome co-purified with nucleoli. It was also shown that these large DNA stretches contain heterochromatic regions, zinc finger, and other genes and a high density of AT-rich sequence elements.

Recently, the 4C (circular chromosome conformation capture) approach was used to determine the whole-genome contacts of rDNA clusters (4C-rDNA) more precisely. and it was demonstrated that they are important for epigenetic regulation of gene expression [12]. The most frequent and stable contact sites in different chromosomes occur in the pericentromeric regions, regions possessing hot spots of DSBs (double-strand breaks), regions possessing H3K27ac and H3K4me3 marks, regions possessing CTCF and RNA polymerase II binding sites [12,13]. However, the detailed study of 4C-rDNA data, namely, the identification of particular gene families and different epigenetic marks at the rDNA contact sites genome-wide, was not performed. In this study, we analyzed the genetic and epigenetic features of rDNA-contacting regions in more detail. Our data strongly suggest that the genes at these sites play an important role in the regulation of gene expression, differentiation, and development.

## 2. Materials and methods

### 2.1. 4C Procedure

The DNA samples for the 4C experiments were isolated according to procedures described previously [14,15]. The HEK293T cells were fixed in 1.5% formaldehyde, and nuclei were isolated, followed by digestion with *Eco*RI enzyme and ligation of extensively diluted DNA to favor intramolecular ligations. To shorten the ligation products, digestion with *Fae*I was performed followed by ligation of diluted DNA samples to favor circularization. The details are described in the Supplementary Materials. The primers for 4C-rDNA were selected inside the IGS (intergenic spacer) as described previously [12]. The final DNA samples were used for the preparation of DNA libraries that were subjected to deep sequencing using a HiSeq1500 (Illumina, San Diego, CA, USA) using up to 150 nt long reads. The 4C-rDNA raw data corresponding to four biological replicates corresponding to untreated or heat-shock-treated cells were deposited under accession numbers GSE121413 and GSE121417.

### 2.2. Computer Treatments

The data on 4C-rDNA were processed in the following way. The raw data obtained using an Illumina system were decoded to FASTQ format using native Illumina Casava software, v. 1.8. Initially, we used FastQC [16] to check the quality of the reads which was very good (for almost all nucleotides in the reads, the quality parameter Q was above 28; see Supplementary Materials for additional comments). The next step was to remove the nested PCR primers: primers 5′GCCTAAGCCTGCTGAGAACTTTC 3′, 5′CAGCATTCTGTAGGGAGATCAAATC 3′ (used for PCR-2) and primers 5′TCTTTGAAAAAAATCCCAGAAGTGGT 3′ and 5′AAGTCCAGAAATCAACTCGCCAGT 3′ (used for PCR-1). This was done by cutadapt software [17]. We removed the primer sequences by assuming that any of the primers could be at either end of a read in direct or reverse-complement coding. Only sequences with the detected and removed primers were selected for further analysis. Cutadapt version 1.15 [17] was used in paired-end mode for primer removal and quality trimming with the following parameters: --trim-n --minimum-length 20 -q 26,26 --pair-filter=both -g GCCTAAGCCTGCTGAGAACTTTC -g CAGCATTCTGTAGGGAGATCAAATC -a GAAAGTTCTCAGCAGGCTTAGGC -a GATTTGATCTCCCTACAGAATGCTG -G GCCTAAGCCTGCTGAGAACTTTC -G CAGCATTCTGTAGGGAGATCAAATC -A GAAAGTTCTCAGCAGGCTTAGGC -A GATTTGATCTCCCTACAGAATGCTG. At the second step of adapter removal, the untrimmed output sequences from the first step were trimmed again with the following parameters: --trim-n --minimum-length 20 -q 26,26 --pair-filter=both -g TCTTTGAAAAAAATCCCAGAAGTGGT -g AAGTCCAGAAATCAACTCGCCAGT -a ACTGGCGAGTTGATTTCTGGACTT -a ACCACTTCTGGGATTTTTTTCAAAGA -G TCTTTGAAAAAAATCCCAGAAGTGGT -G AAGTCCAGAAATCAACTCGCCAGT -A ACTGGCGAGTTGATTTCTGGACTT -A ACCACTTCTGGGATTTTTTTCAAAGA. Then, the results of the first and second primer-removing steps were united for further processing.

We tested the following programs for the mapping of the reads: bowtie2 and BWA [18,19] and methods bwasw and mem, respectively. The best results for genome-wide 4C read mapping were produced by the BWA method mem which produced a larger number of unique and non-unique alignments and, thus, the mappings were performed using the BWA 0.7.12 method mem [20]. Further processing was done by samtools [21]. Samtools 1.6 [22] was used to remove unaligned reads from the SAM file, to convert it to BAM format, and to call variants depending on possible differences (SNPs/INDELs) between the sequenced reads and the reference genome sequence used (hg19/GRCh37.p13). At the end of the processing, the files were in a bedGraph format for further analysis.

### 2.3. Control of Reproducibility of 4C Sequencing

To assess the reproducibility of 4C sequencing, all experiments were repeated twice (biological replicates). The BAM files with aligned reads for each replicate were analyzed by deepTools2 [23]. All files were RPKM normalized using the bamCoverage instrument with the options --effectiveGenomeSize 2864785220 --normalizeUsing RPKM –exactScaling. All unique reads (single 4C contacts) were eliminated and multiBigwigSummary and plotCorrelation instruments were used to calculate Pearson correlation coefficients and to build scatterplot images. The Pearson correlation coefficient was 0.99 for 4C untreated replicates and was 0.98 for 4C heat shock replicates.

### 2.4. Filtering for Contact Detection and Search for Gene-Contact Coordinates

The following approach was used to choose the most prominent 4C contacts for further genetic analysis. The reads mapped onto the genome were attributed to the same contact if they intersected by at least one nucleotide. The reads associated with detected 4C contacts were united into a dataset from the bedGraph files with the 4C replicates using the BEDTools [24] intersect, merge tools, and our own Perl scripts. We picked up the reads associated with 4C contacts that were reproducible in both replicates. The coverage of a contact fragment corresponded to the mean of the covering reads over replicates. The threshold for the mean was taken as 40 to ensure reliable detection [25]. In our 4C-rDNA experiments, we used the six-cutter *Eco*RI enzyme and, thus, we assumed that the contacts were mapped at a resolution of ±2.5 kb. Therefore, each dataset was mapped onto the hg19 list of genes with each contact size extended to 5 kb using BEDTools [24] and our own mapping pipeline that was created in Perl. Each gene can be characterized by the corresponding complete number of associated contact reads, and the resulting set of genes can be ranked according to these numbers. We chose the genes with the numbers of associated reads exceeding 100. As a result, this approach produced 4920 genes which were used for gene ontology searches, ChIP-Seq profile plotting, and RNA-Seq differential analysis. Figure S1 shows that all chromosomes are involved in the contacts with the selected rDNA-contacting genes.

### 2.5. Viewpoint and Estimation of Proximity Effects for Cis-Interactions

We also investigated the possible proximity effects for the viewpoint (“anchor” or “bait”) in our results. In the human genome, rDNA clusters are located on the short arms of acrocentric (13, 14, 15, 21, 22) chromosomes. We tested the amount of the most prominent 4C contacts (those that mapped to genes with a threshold of above 100 reads) in the 5 Mb vicinity from rDNA locations in all acrocentric chromosomes and found the absence of such contacts. This indicates that near-bait and cis-interactions did not affect our results and were neglected in the further analysis.

### 2.6. Gene Quantification and Differential 4C Analysis

As mentioned above, we assumed that the contacts were mapped at a resolution of ±2.5 kb. Therefore, we extended each 4C-rDNA mapped contact fragment to the length of 5 kb for the study of the association between 4C contacts and the genes in the hg19 genome list. The hg19 genome annotation was obtained in the GTF format from Ensemble, release 87 for the GRch37/hg19 genome build. Quantification of genes associated with 4C contacts was performed by featureCounts [26] with the following options: -a hg19.87.gtf -t gene -g gene_id -M --readExtension5 2500 --readExtension3 2500. The quantified genes list was analyzed by the DESeq2 R package [27] using two 4C replicates as “control” and two 4C heat-shock replicates as “experiment”. The results of the differential analysis were used for further analysis with GeneOntology. Volcano plots were created by EnhancedVolcano R package [28].

### 2.7. Genome-Wide Profiles

All ChIP-Seq data analyzed in this study were processed uniformly by the following routine. In the first step, all reads downloaded from Encode and SRA projects were length and quality trimmed by Trimmomatic 0.36 [29] with the following parameters: SLIDINGWINDOW:4:22 MINLEN:20 (scan the read with a four-base-wide sliding window, cutting when the average quality per base drops below 22, all reads shorter than 20 are omitted). Bowtie2 2.3.2 was used to map reads to the genome with the preset --very-sensitive. In the next step, we excluded all unaligned reads from the output SAM file by samtools (key –F4) and converted it to BAM. Duplication removal, which is essential for ChIP-Seq data processing, was performed by the samtools manual’s recommended pipeline: a) sort BAM by name: samtools sort -n; b) add ms and MC tags for markdup to use later: samtools fixmate -m; c) sort BAM by position: samtools sort; d) finally, mark and remove duplicates: samtools markdup –r. Finally, MACS2 v. 2.1.1 [30] was used for peak calling and genome-wide profile creation as follows: macs2 was called with parameters callpeak -f BAM --bdg --gsize hs --call-summits to detect peaks, their summits and build signal and input genome-wide profiles (--bdg parameter). Then macs2 was used again to obtain genome-wide profiles with input removed: macs2 bdgcmp -t *treat_pileup.bdg -c *control_lambda.bdg -m FE. The obtained profiles were converted from bedGraph to BigWig format by UCSC Genome Tools (45. Kuhn) and were applied to plot graphics by SeqPlots [31]. The following ChIP-Seq data were used for profiling: GEO signal GSM1895987, input GSM1895989 (for TAF15 in HEK293 cells); EncodeProject ENCSR906PEI (for SP1 in HEK293T cells); GEO signals GSM2571787, and GSM2571788, inputs GSM2571785, and GSM2571786 (for NCAPG2 in HEK293 cells); GEO signal GSM1895988, input GSM1895989 (for H3K9ac in HEK293 cells); GEO signal GSM1250375, input GSM1250377 (for FOXK1 in HEK293T cells); GEO signal GSM1895985, input GSM1895989 (for FUS in HEK293 cells); EncodeProject ENCSR000EVD (for ZNF263 in HEK293 cells); GEO signal GSM1081542, input GSM1081543 (for RAD21 in HEK293 cells); EncodeProject ENCSR000EZA (for POLR2A in HEK293 cells); GEO signals GSM1239071, and GSM1239072, input GSM1239078 (for p300 in HEK293 cells); EncodeProject ENCSR000FCK (for H3K36me3 in HEK293 cells); GEO signals GSM2171414, and GSM2171415, input GSM2171418 (for H3K14ac in HEK293 cells); GEO signals GSM1239075, and SM1239076, input GSM1239078 (for CBP in HEK293 cells); EncodeProject ENCSR000DTW (for CTCF in HEK293 cells). For profiling of small RNAs isolated from in HEK293T cells, the GEO data with accession number GSM955512 were used. Venn diagrams were created by the website [32].

### 2.8. RNA-Seq Analysis

All RNA-Seq data analyzed in this study (Accession numbers GSE130262 and GSE130493) were processed uniformly by the following scheme. Trimmomatic [29] was used to filter low-quality reads with the following options: LEADING:18 TRAILING:18 SLIDINGWINDOW:4:22 MINLEN:20. Then, filtered reads were aligned to the GRCh37/hg19 genome using the STAR RNA-Seq aligner [33]. The package featureCounts was used to quantify alignments to the GRCh37/hg19 Ensemble v.87 list of genes with the options: -a hg19.87.gtf -t exon -g gene_id *.bam. After that, the list of quantified genes was filtered using the threshold of more than 100 reads associated with 4C contacts (see above). This provided the list of 4920 genes. Finally, this list of genes was used for differential RNA-Seq analysis by Deseq2 R package with heat shock treatments and 6 h recovery data. Volcano plots were created by EnhancedVolcano R package.

### 2.9. Statistical Calculations

To ensure that the selected rDNA-contacting 4920 genes corresponded to a non-random set of genes, we performed a comparison of this list of genes with ten randomly generated lists of human genes, each also containing 4920 genes. The following procedure was used to estimate the standard deviation of randomly intersecting gene lists. The *H. sapiens* hg19 gene list was obtained from the UCSC genome project server. Only unique names were left in the list by using an in-house Perl script. The size of the unique gene list was 47,234 gene names. The list was shuffled randomly once by the Fisher–Yates algorithm, and the first 4920 elements were chosen to be the reference list. At each step, we shuffled the list again randomly using the Fisher–Yates algorithm and chose the first 4920 elements and then calculated the number of coinciding elements with the reference list. The procedure was performed 100,000 times for the 4920 genes in the list. The basic statistics calculation was performed to estimate the mean value and normal deviation of the distribution. The following results were obtained. For the randomly generated list of 4920 genes, the overlapping part = 0.06259 ± 0.00434, min = 0.04567, max = 0.08119. Thus, in the most extreme case, we would have an overlap of 0.0811 (i.e., 8.11%), while we observed an overlap of 39% among the gene lists for the selected 4290 genes in the 4C-rDNA experiments. These data indicate that the *p*-values were <<10^−6^ for the random selection of genes generated during the 4C-rDNA procedure.

## 3. Results

### 3.1. rDNA Clusters Contact the Genes Involved in Differentiation of Neurons in HEK293T Cells

Previously, we performed 4C-rDNA experiments using DNA primers located downstream of the selected *Eco*RI site in the IGS (intergenic spacer) inside rDNA units [12]. The contact sites of rDNA clusters in HEK293T cells were previously described, but the nature of genes located at these sites was not studied. In this study, in order to use HEK293T cells of the same passage number for both 4C-rDNA and RNA-Seq experiments, we performed two new independent 4C-rDNA experiments using the same set of primers for amplification of rDNA-contacting regions. In this way, two biological replicates were sequenced, and a set of rDNA-contacting genes was identified by computer treatments (see Methods and references 16–33 therein) and selected for analysis.

To date, the only tool for the study of the nature of thousands of genes is the gene ontology search. To study what kinds of genes are located at the detected rDNA-containing sites in human chromosomes, we used several gene ontology resources. One of them, g:Profiler, was designed to search for enriched Gene Ontology (GO) terms in a target list of genes [34]. For the current study, we used a threshold of 100 or more 4C-rDNA reads for selection of the rDNA-contacting sites and for identification of the genes located at these sites (see Materials and Methods and Supplementary Materials). The corresponding set of genes revealed statistically significant enrichment results.

The list of these 4920 genes that are located in the 5 kb vicinity of rDNA-contacting sites in HEK293T cells is shown in Table S1. The list includes 126 genes specifying long intergenic non-protein coding RNAs (linc RNAs), 122 genes for miRNAs, 136 zinc finger genes, and many other genes encoding different transcription factors (TFs), several RNA polymerases (*POLR2A, POLR2F, POLE, POLQ, POLR3C, POLR1A*), genes involved in RNAi (*DICER1*, *DICER1-AS1, PIWIL3, PIWIL4*, *AGO3*). These data clearly suggest that rDNA-contacting genes could be involved in the control of gene expression.

**Figure 1:**
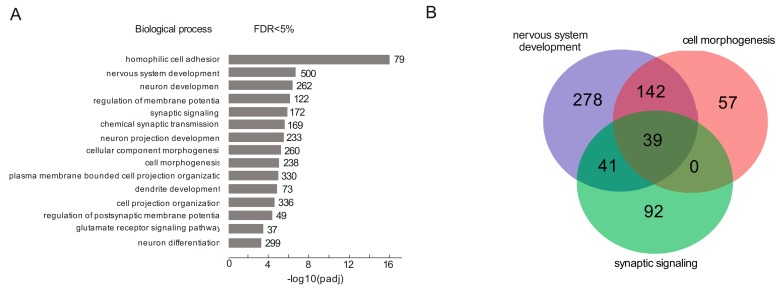
Search of rDNA-contacting genes for enriched Gene Ontology (GO) terms using g:Profiler. (**A**) The top 15 detected terms of biological processes associated with rDNA-contacting genes. The values to the right of bars show the number of rDNA-contacting genes associated with a term. The list of the corresponding genes is shown in Table S2. (**B**) The Venn diagram shows the intersections among rDNA-contacting genes associated with three terms (the list of the corresponding genes is shown in Table S3.

Using the GO search for the biological processes in which rDNA-contacting genes are involved, we detected statistically significant enrichment (padj < 0.003) for the top fifteen GO terms. Figure 1A shows the results of a search ranked by *p*-value score for GO terms giving from 37 to 500 genes. In particular, we observed an enrichment of a group of genes involved in the development of neural cells, which could probably be explained by the neuronal origin of HEK293T cells [35].

The largest groups of genes (Table S2) are involved in nervous system development (500 genes) and cell morphogenesis (338 genes). There were also 262 genes involved in neuron development. One group of genes is involved in homophilic cell adhesion, i.e., the process of attachment of a plasma membrane adhesion molecule in one cell to an identical molecule in an adjacent cell. These genes are involved in calcium-dependent cell adhesion which may be involved in the establishment and maintenance of specific neuronal connections in the brain [36]. Figure 1B shows that the majority of rDNA-contacting genes that are associated with cell morphogenesis overlaps with genes involved in nervous system development (181 of 238) while many more genes (319 of 500) are specific to nervous system development (Table S3).

The search of a random group of genes in GO resources produced no GO terms with enrichment above the specified *p*-value (10^−3^). Independently, to ensure that the selected 4920 genes represent a non-random set of genes, we performed a comparison of this list of genes with ten randomly generated lists of human genes, each also containing 4920 genes. About 6% of genes were found within regions that overlapped with different random groups and selected genes. The same overlap values (6%) were observed among different random groups of genes. These data strongly suggest that the selected 4920 genes represent a specific non-random set of genes (*p*-values <<10^−6^; the calculations are presented in the Methods section).

**Figure 2:**
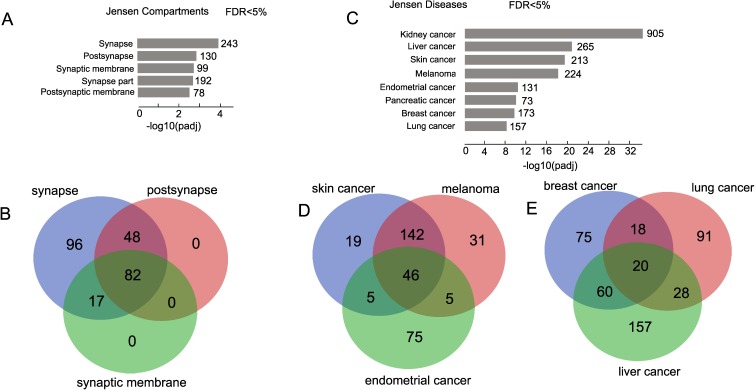
Search of rDNA-contacting genes for enriched terms using the Jensen Compartments and Jensen Diseases libraries of Enrichr. (**A**) The top five compartments associated with rDNA-contacting genes in the library. The values to the right of the bars show the number of rDNA-contacting genes associated with a term. The list of the corresponding genes is shown in Table S4. (**B**) The diagram shows the number of rDNA-contacting genes associated with three compartments. The list of the corresponding genes is shown in Table S5. (**C**) The top eight diseases highly associated with rDNA-contacting genes in the library. The values to the right of the bars show the number of rDNA-contacting genes associated with a term. The list of the corresponding genes is shown in Table S6. (**D**) Venn diagram showing the intersections between rDNA-contacting genes associated with skin cancer, melanoma, and endometrial cancer. The list of the corresponding genes is shown in Table S7. (**E**) Venn diagram showing the intersections between rDNA-contacting genes associated with breast, lung, and liver cancers. The list of the corresponding genes is shown in Table S8.

The data on the involvement of rDNA-contacting genes in the differentiation of neural cells were independently confirmed by searching in the Jensen Compartments library (Enrichr databases) [37]. We detected statistically significant enrichment for the top five GO items. Interestingly, all five items are associated with the synapse (Figure 2A). The complete list of these genes is shown in Table S4.

The largest group of rDNA-contacting genes were found in the synapse (243). Post-synapse and synapse membrane groups of genes entirely overlapped with the group of synapse genes (Figure 2B, Table S5). The data clearly support the conclusion that rDNA-contacting genes in HEK293T cells are associated with the differentiation of neural cells.

Previously, it was demonstrated that HEK293T cells, even though they were originally isolated from human embryonic kidney, have an unexpected relationship to neurons but not to typical kidney epithelial cells [35,38]. Our results are clearly in agreement with this notion.

### 3.2. rDNA-Contacting Genes Are Associated with Different Cancers

To further investigate the possible involvement of genes that are regulated by rDNA-mediated mechanisms, we used the Enrichr search of the Jensen Diseases library. The top eight examples of diseases were extremely highly associated (adjusted *p*-value up to 10^−33^) with different cancers (Figure 2C). The corresponding genes are shown in Table S6. The largest set of genes (905 genes) was found to be associated with kidney cancer. The Venn diagrams demonstrate that the number of the genes corresponding to a single particular cancer is not as large. The most significant overlapping was observed between melanoma and skin cancer cells (Figure 2D, Table S7) which supports the general view that the related cell types should be controlled by very similar sets of genes. These results suggest that a more detailed analysis of rDNA-contacting genes that are common for different cancers may potentially identify new cancer-associated genes.

### 3.3. rDNA-Contacting Sites at Genes Possess a Specific Set of Epigenetic Marks

For independent validation of the data obtained by gene ontology searches, we searched for characteristic epigenetic features at rDNA-contacting sites possessing the genes. For the genome-wide study of the epigenetic features at rDNA-contacting sites in the HEK293T cells from which our 4C-rDNA data were obtained, we used the available ChIP-Seq and RNA-Seq data that were also obtained in these cells. The profiles of epigenetic features were determined ±1.5 kb around the centers of rDNA-contacting regions using SeqPlots interactive software [31].

**Figure 3:**
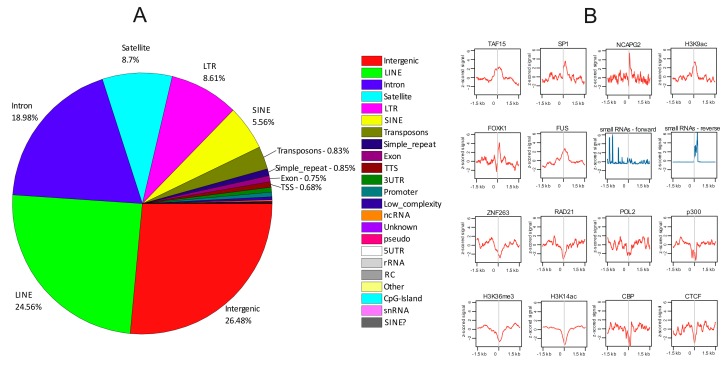
Distribution of rDNA-contacting sites in different portions of the human genome (**A**) and profilers of different transcription factors or RNA molecules around the central parts of rDNA-contacting sites where 4920 rDNA-contacting genes reside (**B**). The profiles shown in red and in blue correspond to ChIP-Seq and RNA-Seq data, respectively.

Figure 3A shows that these sites correspond mainly to intergenic regions, LINEs, and introns. We observed that, generally, these sites are enriched by TAF15, SP1, NCAPG2, FOXK1, and FUS which are involved in different cellular processes including transcription, differentiation, and chromosome assembly (Figure 3B). The central regions of rDNA-contacting sites are enriched with the H3K9ac mark, which is associated with active transcription [39] and with small non-coding RNAs. Interestingly, these 20–100 nt small RNAs that are highly enriched at rDNA-contacting sites (z-score of up to 6) demonstrate different expression profiles of forward and reverse strands. They were isolated and mapped after crosslinking of RNA with the proteins and immunoprecipitation by antibodies to DGCR8, a double-stranded RNA-binding protein that is the microprocessor component that recognizes and directly binds different RNAs including several hundred mRNAs, small nucleolar RNAs (snoRNAs), and long non-coding RNAs, and is involved in miRNA biogenesis [40]. The sense RNAs are transcribed at different distances from the rDNA-contacting sites centers, while the reverse strands are transcribed very closely to it (Figure 3B). Potentially, these RNAs could target protein complexes at these sites and induce either silencing or activation of transcription. The data strongly suggest the RNA-mediated inter-chromosomal interactions of chromatin regions possessing rDNA clusters with specific genomic regions. Central regions of rDNA-contacting sites are highly enriched (z-score of up to 5) by binding sites of NCAPG2. This protein is a regulatory subunit of the condensin II complex that, together with the condensin I complex, plays an important role in chromosome assembly and segregation in mitosis [41].

We also detected that the central regions of rDNA-contacting sites were clearly depleted of ZNF263, RAD21, POL2, p300, CBP, and CTCF binding sites. The similar profiles revealed for the p300 and CBP binding sites support the robustness of the data on profile building, because these proteins have similar structures and interact with many transcription factors at the same sites and act to increase the expression of their target genes. The same is true for CTCF and RAD21, which possess similar profiles. These proteins are involved in the formation of chromatin loops and reveal similar distributions in chromosomes [42]. The rDNA-contacting sites were also depleted of H3K36me3 and H3K14ac marks which are associated with active genes. These data suggest that a set of rDNA-contacting genes was silenced.

The epigenetic marks detected at rDNA-contacting sites indicate that they are enriched with the binding sites of different proteins and small RNAs that play important roles in gene expression and differentiation. Taken together, these data strongly support the conclusions drawn from our gene ontology studies.

### 3.4. Heat Shock Treatment Dramatically Changes the Pattern of rDNA-Contacting Sites

To test whether the detected rDNA contacts are structurally stable or could be affected by some physiological influences upon cells, we used a heat shock treatment. The HEK293T cells were incubated for 30 min at 43 °C and, after incubation for 2.5 h at 37 °C, the 4C-rDNA procedure and RNA-Seq were performed. Heat shock treatment disassembles the nucleoli and causes substantial changes in the localization of NORs and represses rRNA synthesis [43,44,45]. If the functional state of rDNA units is affected, could it lead to the changes in their inter-chromosomal contacts?

**Figure 4:**
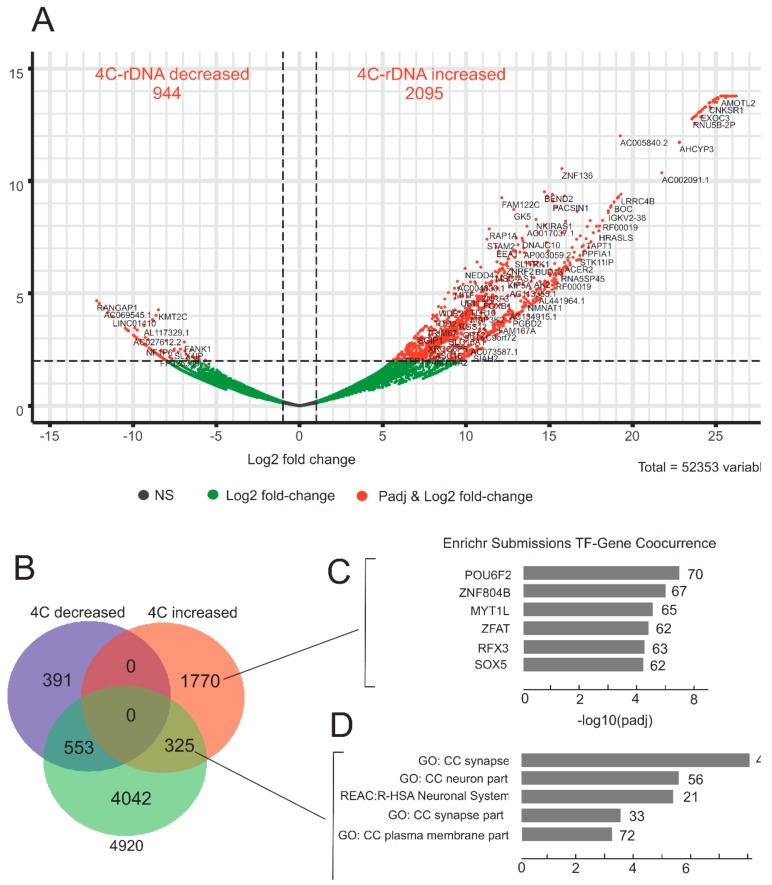
Analysis of changes in the contacts of genes with rDNA clusters after heat shock treatments. (**A**) The volcano plot presents the statistically significant log2-fold changes in contacts of genes with rDNA clusters determined in 4C-rDNA experiments. The list of 52,353 genes from gene annotation for human genome GRCh37/hg19 build 87 was downloaded from Ensembl FTP server [49]. Of these, 944 genes demonstrated a decrease in the number of contacts, while 2095 genes revealed an increase in the number of contacts. The list of the corresponding genes ranked by padj is shown in Table S9. (**B**) The diagram shows the intersections of these two groups of genes with the list of selected 4920 rDNA-contacting genes detected in non-treated HEK293T cells. The list of the corresponding genes is shown in Table S10. (**C**) The 1770 genes that demonstrated an increase in the number of contacts with rDNA after heat shock treatment were searched using the Enrichr Submissions TF-Gene Co-occurrence library of Enrichr. Genes specifying TFs were identified and demonstrated co-occurrence at specific groups of genes. The top six genes that specify TFs are shown. The value to the right of the bars indicates the number of the regulated genes. The list of these genes is shown in Table S11. (**D**) The top five GO terms associated with rDNA-contacting genes detected in untreated cells that increased their contacts with rDNA after heat shock treatment are associated with the development of neurons. The values to the right of the bars indicate the number of the corresponding genes. The list of these genes is shown in Table S12.

We observed dramatic changes in the patterns of rDNA contacts upon heat shock treatment. Figure 4A and Table S9 show that after the treatment, many rDNA-contacting genes decreased the number of contacts with rDNA clusters (log2 change up to 10^−12^) or considerably increased the frequencies of the contacts (log2 change up to 10^26^). We used stringent cut-off conditions for the selection of genes that changed the number of their contacts with rDNA. There are many genes shown in black and in green dots in Figure 4A that are indicated below the adjusted p-values. It follows that deep rearrangements in the contacts of chromatin loops containing rDNA clusters occur at about 3000 chromosomal sites. In order to analyze what happens after treatment to the genes that are normally in contact with rDNA, we built a Venn diagram to observe the overlap between this set of 4920 genes and the genes that revealed statistically significant changes in the contacts with rDNA clusters. We observed that about 7% of rDNA-contacting genes (325 genes) from the list of 4920 genes in untreated cells increased the number of their contacts with rDNA. At the same time, 553 genes (about 11%) decreased the number of contacts (Figure 4B and Table S10).

To uncover the nature of the genes that changed their contacts with rDNA after heat shock treatment, we performed a search of these gene groups in different Gene Ontology resources. No statistically significant enrichments were detected for the genes that decreased their contact frequencies with rDNA. At the same time, we observed that two groups of genes that increased the number of contacts with rDNA clusters belong to specific GO items.

The first group of 1770 genes that did not have contacts with rDNA (or had contacts below the threshold of 100 4C-rDNA reads) increased the number contacts with rDNA. This group included 117 genes encoding linc RNAs and 23 zinc finger proteins. The search revealed statistically significant correspondence with more than 50 transcription factors (the top six of them are indicated in Figure 4C) that exhibited co-occurrence with a specific set of genes (approximately 60–70) which are regulated by these multiple sets of transcription factors (Table S11). It appears that heat shock treatment switches on a new type of regulation of gene expression and triggers the massive changes in contacts with rDNA clusters and, as a result, a specific set of genes specifying that transcription factors form much more frequent contacts with rDNA.

The second group of 325 genes had more than 100 4C-rDNA reads before the treatment, increased the number of contacts with rDNA, and were associated with neuron cells (the top five items are shown in Figure 4D and Table S12).

At the same time, many genes that we currently cannot characterize by gene ontology tools significantly decreased their associations with rDNA. These data suggest that during deep rearrangements of rDNA contacts with different genomic regions after heat shock treatment, rDNA clusters lose contact with hundreds of genes and, at the same time, shape stronger contacts with a specific set of genes that control the cellular components of neurons. These results are in agreement with the data suggesting additional roles of nucleoli and ribosome biogenesis in nervous system development [46,47,48].

The majority of rDNA-containing genes from the list of the selected 4920 genes retained the contacts with rDNA after heat shock treatment (Figure 4B and Table S10). This group of 4042 genes included 98 genes specifying linc RNAs, 113 zinc finger proteins, and 7 subunits of RNA polymerases I, II, and III.

### 3.5. rDNA-Contacting Genes Specifying Transcription Factors Change Their Expression after Heat Shock Treatment and Induce an Epigenetic Switch

**Figure 5:**
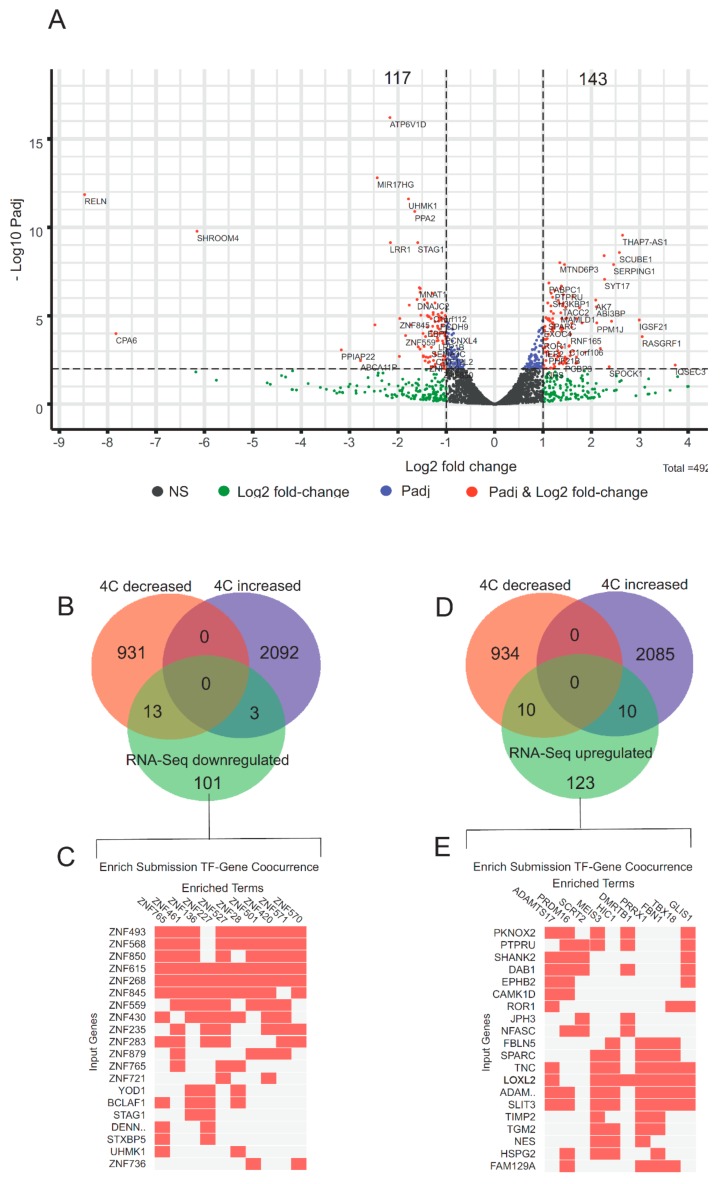
Differential expression of rDNA-contacting genes after heat shock treatment. (**A**) The volcano plot presents the statistically significant log2-fold changes in the expression of rDNA-contacting genes determined in RNA-Seq experiments (after heat shock treatment and recovery at 37 °C for 6 h). The expression of 4920 rDNA-contacting genes was analyzed. Of these, 177 genes were downregulated while 143 were upregulated. The list of the corresponding rDNA-contacting genes ranked by padj is shown in Table S9. (**B**) The diagram shows the intersections of downregulated rDNA-contacting genes with the lists of genes that revealed a decrease (944 genes) or increase (2095 genes) in rDNA contacts in Figure 4A. The list of the corresponding genes is shown in Table S15. (**C**) The top 20 downregulated genes which did not change their contacts with rDNA after heat shock treatment (indicated as “input genes”) specify TFs that reveal co-occurrence at specific groups of genes. The list of 100 of these genes (indicated as “enriched terms” in Figure 5C) is shown in Table S16. (**D**) The diagram shows the intersections of upregulated rDNA-contacting genes with the lists of genes that revealed a decrease (944 genes) or increase (2095 genes) in rDNA contacts in Figure 4A. The list of the corresponding genes is shown in Table S17. The list of the overlapping 10 upregulated genes associated with the development of neurons that increased their contacts with rDNA is shown in Table S18. (**E**) The top 20 upregulated genes which did not change their contacts with rDNA after heat shock treatment (indicated as “input genes”) specify TFs that reveal co-occurrence at specific groups of genes. The top 25 of these genes (indicated as “enriched terms” in Figure 5E) are shown in Table S19.

Surprisingly, statistically significant changes in transcription patterns detected by RNA-Seq after heat shock treatment (incubation for 30 min at 43 °C followed by further incubation for 2.5 h at 37 °C) were observed only in a set of mitochondrially encoded RNAs (Table S13). However, under these conditions, we observed significant changes in the number of rDNA contacts. The data indicate that the changes in rDNA contacts appear rapidly, while alterations in the transcription patterns are slower and probably need more time after heat shock treatment. After more prolonged incubation (6 h) at 37 °C following the heat shock treatment, we observed the expected changes in the expression of rDNA-contacting genes (Figure 5, Table S14).

Figure 5A shows that 6 h after the heat shock treatment, 117 rDNA-contacting genes were downregulated. Thirteen of them demonstrated a decrease in the number of contacts with rDNA genes, while only three genes showed an increase in the number of these contacts (Figure 5B, Table S15).

The remaining 101 downregulated genes that did not alter their contacts with rDNA after the treatment (Table S15) are associated with 100 genes, all of which correspond to *ZNF* genes (designated as “enriched terms” in Figure 5C; the complete list of these genes is shown in Table S16). These genes are jointly regulated by the downregulated rDNA-contacting genes and are involved in transcription regulation.

At the same time, 143 genes were upregulated after heat shock treatment (Figure 5A). Among them, 10 genes decreased the number of contacts with rDNA (Figure 5D, Table S17). We did not find any significant associations for them in GO resources. Ten upregulated genes that increased their contacts with rDNA clusters correspond to genes that are involved in the control of the neuronal identity of HEK293T cells (Table S18). The majority of the upregulated rDNA-contacting genes did not alter the number of their contacts with rDNA (Figure 5D). This group of 123 genes specifies TFs that demonstrate co-occurrence at specific groups of genes (Figure 5E, Table S19). These genes (designated as “enriched terms” in Figure 5E) are jointly regulated by rDNA-contacting genes and comprise an interesting set of genes that are involved in the regulation of transcription by RNA polymerase II (Table 1, Table S20).

The data suggest that after heat shock treatment, 117 rDNA-contacting genes were downregulated, and another 143 genes were upregulated (Figure 5). Both groups of genes, which did not overlap, included genes that retained their contact with rDNA and specify TFs. It appears that there was an epigenetic switch orchestrating gene expression by decreasing the expression of one set of genes while at the same time increasing the expression of another set of genes in response to the treatment. The data on the co-occurrence of TFs specified by downregulated and upregulated rDNA-contacting genes suggest that there is a cascade of regulation controlled by rDNA-contacting genes.

### 3.6. Genomic Browser Analysis Indicates Changes in rDNA Contacts after Heat Shock Treatment

**Figure 6:**
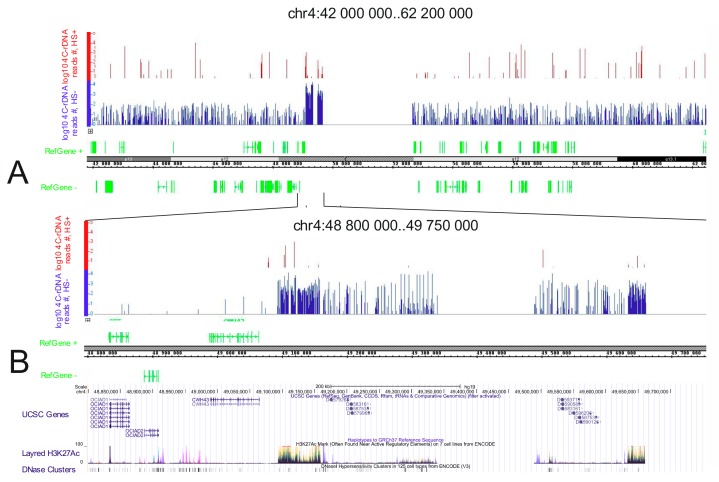
Profiles of 4C-rDNA reads in a fragment of chr4 before (shown in blue) and after (shown in red) heat shock treatment. (**A**) The position of rDNA contacts around the pericentric region of chr4. (**B**) The profiles of rDNA contacts and the distribution of H3K27ac marks in the pericentric region of chr4.

We also observed changes in the rDNA contacts upon heat shock treatment using genomic browsers. Figure 6 shows that the detected rDNA contacts are very sensitive to temperature stress. Dramatic changes were observed in the distribution of the most prominent rDNA contacts (Figure 6A,B).

Figure 6B demonstrates changes in the rDNA contacts inside of a ~1- Mb region of chr4. Normally, the region possesses two long regions (240 and 160 kb in length) that each contains multiple rDNA-contacting sites. Two regions possessing the highest density of rDNA-contacting sites precisely correspond to the regions of the same size that are decorated by the most prominent H3K27ac marks. The most intensive marks spanning about 70 (left) and 20 kb (right) may correspond to putative super-enhancers [50]. There is a clear correlation between the densities of rDNA-contacting sites and the intensities of the H3K27ac marks. These data indicate that rDNA contacts can take place within rather long chromosomal regions possessing specific epigenetic marks. These regions are important for the epigenetic regulation of gene expression. The sensitivity of the contacts in such regions to heat stress suggests that rDNA contacts could be involved in the mechanism of this regulation.

## 4. Discussion

Currently, several mechanisms of regulation in different chromosomal domains are being studied, including the coordinated expression of the genes located in the same chromosomal region. They are transcriptional territories, Pc-domains, forum domains, and TADs. Alternative mechanisms involving the concerted silencing of genes transcribed from different genomic regions by RNAi targeting of SINEs’ stretches that are incorporated in 5′or 3′ non-coding regions of the corresponding mRNAs have been suggested [51,52,53,54]. Genetically, transvection—an epigenetic mechanism operating by chromosome pairing and leading to activation or repression of genes—was discovered at the *Bithorax* complex in *Drosophila* in 1954 by Edward Lewis, who coined this term, and it was shown that transvection can be altered solely by disruption of somatic (or meiotic) pairing [55].

The data presented here suggest another mechanism in which multiple chromosomal contacts of rDNA genes are involved in the epigenetic regulation of thousands of genes located in different chromosomes and belonging to different chromosomal domains. The underlying mechanisms are not clear and remain to be investigated. Nevertheless, there is evidence strongly suggesting that these rDNA contacts could be the reason, and not the consequence, of the changes in epigenetic states of the corresponding genomic regions. It was shown that rDNA heterochromatin initiates heterochromatinization of ESC genomes and that inhibition of rDNA heterochromatin prevents ESC differentiation [7,56]. It was also shown that the formation of this repressed chromatin outside of the nucleolus activates transcription of different genes. Taken together with our findings including the results on the dynamics of rDNA contacts upon heat shock treatment, these data suggest that nucleoli could be directly involved in the epigenetic regulation of thousands of human genes. We suggest that rDNA contacts could be important for both the development and maintenance of differentiated states. We believe that short stresses do not change the differentiated state because the epigenetic memory formed by different mechanisms is preserved.

**Figure 7:**
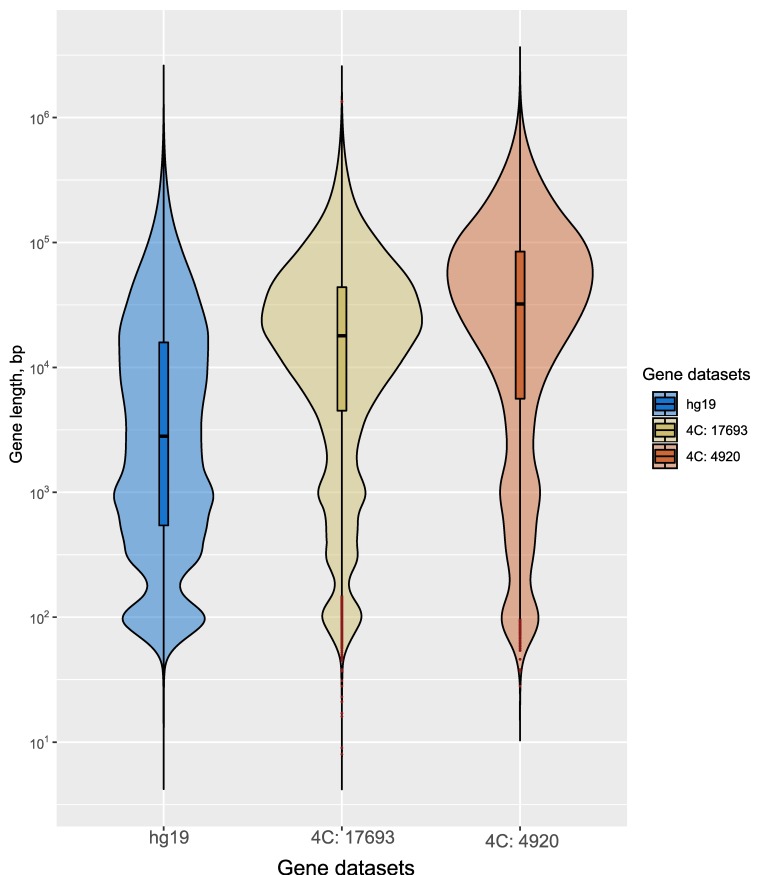
The violin plots for gene length in the genome (hg19), all rDNA-contacting genes (17,693 genes), and the selected 4920 genes. The details are described in the Supplementary Materials.

Upon heat shock treatment, the regions corresponding to putative super-enhancers almost completely lose the contacts with rDNA units (Figure 6). This fact should be considered along with the data demonstrating that heat shock treatment leads to repression of transcription of the majority of genes and activation of the transcription of genes encoding cytoprotective proteins [57]. The most abundant sites of rDNA contacts occur at both repressed pericentric regions and regions possessing 10–50 kb stretches of H3K27ac marks, which are characteristic of actively transcribed genes, and at hot spots of DSBs, which are observed in actively transcribed regions [12]. Together with the results on the effects of heat shock stress on rDNA contacts, these data strongly suggest an important role of rDNA units in both activation and repression of particular gene sets. The sensitivity of rDNA contacts to heat shock argues in favor of the functional significance of rDNA units’ contacts in the epigenetic regulation of genes. Gene ontology data strongly support this conclusion. Nevertheless, further studies will be performed to elucidate how the dynamics of rDNA-contacting sites effect the transcription of the corresponding genes. The output of heat shock stress is well studied at the protein level but an analysis of the transcription dynamics by an RNA-Seq approach is yet to be reported [45]. We did not observe any changes in expression by RNA-Seq after heat shock treatment of cells for 30 min followed by incubation at 37 °C for 2.5 h. At the same time, dramatic changes were observed following the same treatment in rDNA contacts (Figure 4). The global pattern of inter-chromosomal contacts, including rDNA contacts, should be removed before mitosis. The reproducible 4C-rDNA results suggest that these contacts are re-established in the G1 phase in each cell cycle. The gene ontology data of the rDNA-contacting genes suggest that in differentiated cells these contacts are required for the maintenance of the differentiation state. The data on the strong association of rDNA-contacting genes in HEK293T cells with the genes involved in neuron development support this conclusion (Figure 1A, Figure 2A, and Figure 4D). Figure 7 shows that the selected 4C-rDNA genes have a bias for long genes in the genome. Long genes are often highly expressed in neurons [58,59]. The activity of long genes in neural cells suggests that the genes reside in open chromatin regions and, thus, are accessible to inter-chromosomal contacts, including the contacts with rDNA clusters. It is likely that these genes are inherently long because they undergo different types of regulation.

Currently, we are studying the kinetics of restoration of the rDNA contacts and the transcription patterns after heat shock treatment as well as the rDNA contacts in different cell types. Our data shows that rDNA genes are involved in the cellular response to heat stress. We speculate that other types of stresses (e.g., DNA damage, transcription-inhibiting stress, proteotoxic stress, or inhibition of proteasome function) could result in another set of rDNA-contacting genes.

Recently, a Hi-C approach was used for the analysis of the interactions of rDNA genes in human cells [60]. The data, with about 1 Mb resolution, suggested that these contacts are enriched in the regions of repressed and late-replicating chromatin as well as CTCF binding sites. The resolution of the 4C-rDNA approach that we describe here is much higher and allows identification of the genes that are in contact with the rDNA clusters. The proximity of *DUX* genes that possess contacts with rDNA genes to nucleoli was demonstrated recently by FISH [61,62], which independently confirms our 4C data on the frequent contacts of this family of genes with nucleoli.

There are about five nucleoli in HEK293T cells. Each nucleolus usually contains up to 60 rDNA units. Previously, it was described that eight *Drosophila* nucleoli made contact with eight different regions in polytene chromosomes [2]. It is difficult to understand how five nucleoli in human cells could ensure the multiple contacts along different chromosomes upon heat shock treatment (Figure 4A). We suggest that, after the treatment, the disassembled nucleoli rapidly change the pattern of contacts with different chromosomes and that particular sites may differ in each individual cell. That is why the 4C-rDNA procedure, which uses about 15 million cells, could result in the appearance of multiple sites. The current view on cell-to-cell variability supports this possibility [63]. Further experiments will address this point. Currently, we are studying rDNA-contacting regions in the K562 and hESM01 cell lines and the preliminary data indicate that there are common rDNA-contacting sites, as well as cell-specific sites, compared with HEK293T cells (unpublished data, manuscript in preparation).

We suppose that active and repressed rDNA clusters could form specific compartments possessing protein complexes that are involved either in the activation or inhibition of transcription. Numerous genomic regions could be specifically recognized by non-coding RNAs with different characteristics and attracted to active or repressed nucleoli. As a result, these compartments could confer active or repressed states to numerous chromosomal regions. We detected that sense or antisense small RNAs are transcribed from the rDNA-contacting sites. It is of interest that about 20% of small RNAs that are recognized by DGCR8 correspond to rRNA [40]. These RNAs could target different genomic regions to particular rDNA clusters. The DGCR8-associated small RNAs also include several hundred mRNAs and long non-coding RNAs. Another possibility also takes into account the fact that the centers of the rDNA-contacting sites are highly enriched with transcription sites of DGCR8-associated non-rRNA small RNAs. These RNAs could target distinct activating or repressing complexes from the corresponding nucleoli compartments to the sites where this RNA was synthesized leading to the formation of rDNA contacts with the specific genomic regions and the induction of repression of activation of transcription at these regions.

We observed that 117 rDNA-contacting genes specifying linc RNAs, including several tens of antisense non-coding RNAs, kept their contacts with rDNA after heat shock treatment (4042 genes shown in Figure 4B and in Table S10). It was shown that the repression of rRNA synthesis induced by heat shock treatment occurs via direct binding of long antisense non-coding RNA transcribed by RNA polymerase II to rDNA, including its promoter and upstream enhancer sequences. These ncRNA target CHD4/NuRD which shifts the promoter-bound nucleosome into a position that does not allow transcription initiation [44]. We speculate that a similar reversible mechanism could be responsible for the downregulation of rDNA-contacting genes induced by heat shock stress. In any case, our data argue in favor of an RNA-mediated mechanism being involved in the epigenetic regulation of many human genes by contacts with active or repressed rDNA clusters.

We detected that rDNA-contacting genes increasing their contacts with rDNA after heat shock are enriched with both genes specifying linc RNAs and TFs that display co-occurrence at the specific set of genes (Figure 4 and Figure 5, Table 1). The data suggest that rDNA-contacting genes are involved in the concerted regulation of large groups of genes. Interestingly, in this regulation, hundreds of rDNA-contacting genes encoding TFs are involved and they often jointly regulate a group of genes that are also involved in the regulation of transcription by RNA polymerase II. It follows that this mechanism of concerted regulation employs a symphony of transcription factors downregulating one set of TFs and upregulated another set of TFs. It was reported that these diverse TFs can interact with the Mediator through the phase-separating capacity of their activation domains and that formation of condensates with the Mediator is involved in gene activation [64]. We speculate that phase separation mechanisms could be involved in trans-regulation induced by contacts of rDNA clusters with genes.

We speculate that nucleoli could be involved in the concerted regulation of a specific set of genes located in different chromosomes by the formation of physical contacts with different chromosomal regions via a phase separation mechanism. It was shown that the TF binding site numbers above sharply defined thresholds drive the formation of transcriptional condensates [65]. A high local concentration of TFs at rDNA clusters could lead to the formation of transcriptional condensates and phase separation that recruits specific chromosomal regions to the nucleoli. The supposition is corroborated by recent evidence of liquid–liquid phase separation in the formation of nucleoli and its stress responses [66].

Previously, we observed that the most prominent rDNA-contacting regions possess very long (from 5 to 50 kb) chromosomal regions possessing the H3K27ac mark which is associated with putative super-enhancers [12]. These sites also correspond to the binding sites of master transcription factors and the Mediator coactivator which drive high-level expression of genes encoding key regulators of cell identity [50]. Figure 3B shows that rDNA-contacting sites are depleted of binding of both RAD21, a core component of the cohesion complex, and CTCF. The data suggest that rDNA contacts take place not at chromatin loop anchors but in the loops themselves that possess putative super-enhancers.

During the last decade, abnormalities in the epigenetic mechanisms of gene expression have been considered by some to be more important in the etiology of cancer than mutations [67]. Disorders of epigenetic regulation can change gene function and lead to malignant cellular transformation [68]. The fact that among the rDNA-contacting genes there are many genes specifying key transcription factors, including P53 and 136 zinc finger proteins, suggests that, if this mechanism of regulation goes awry, the normal epigenetic regulation will be affected, and cancer transformation could appear. We detected a strong association between the rDNA-contacting genes and cancer cells (Figure 2). Recently, it was shown that during malignant progression, rDNA genes reorganize their rDNA-genome contacts and these changes correlate with gene expression changes at associated loci [69]. Our data suggesting that rDNA-contacting genes are associated with different cancers are in agreement with this conclusion.

Recently, three classification systems of cancer genes were suggested [68]. The genetic classification traditionally includes a number of oncogenes which could be activated by mutation and behave in a dominant fashion. Among the rDNA-contacting genes, there is a member of this family (Table S1): *ABL1*. Among the rDNA-contacting genes, there are several tumor suppressor genes: *TP53*, a well-known transcription factor that is mutated or functionally inactivated in many cancer types; *WT1*, which is involved in cell differentiation and is mutated in leukemic cells; *FHIT*, which encompasses one of the common fragile sites where damage can lead to carcinogen-induced translocations; *APC*, tumor suppressor protein that acts as an antagonist of the Wnt signaling pathway; *NF1* and *NF2*, which are associated with neurofibromatosis. The selection classification includes seven driver gene mutations or aberrant expression that are subject to selection during tumorigenesis [68] and three of which (*TP53, ABL1*, and *WT1*) correspond to rDNA-contacting genes. The epigenetic functional classification includes genes in which some of them (*TP53*, *ARID2*, *APC*, *ARID1B, ARID2*, *SMARCA4, NSD1, SETD2, EZH2*, and *BRD4*) are also involved in contacts with rDNA clusters. These data on enrichment of rDNA-contacting genes by genes associated with cancer independently support the data obtained in our gene ontology searches (Figure 2C). In this study, we concentrated on the list of 4920 genes that most frequently had contacts with rDNA. Taken together, these data lead us to conclude that rDNA-contacting genes may be associated with cancer. Our data clearly support the idea that nucleoli play a role in additional cellular functions including the response to cellular stressors, regulation of gene expression, and in carcinogenesis [70]. The detailed analysis of rDNA contacts in different normal and cancer cells and their effects on the regulation of gene expression that we are currently performing could identify new key genes involved in tumorigenesis.

## 5. Conclusions

In summary, genes that form frequent contacts with rDNA clusters are enriched with genes that are involved in the regulation of transcription, differentiation, and cancer. The contacts are very sensitive to heat shock treatment. The rDNA-contacting genes in different combinations jointly regulate large groups of genes. Our data suggest a possible role of rDNA clusters in the differentiation of human cells. We speculate that nucleoli, as membrane-less organelles, directly regulate different genes by the phase separation mechanism.

## Figures and Tables

**Table 1:** Th GO associations of genes that are jointly regulated by 123 rDNA-contacting genes shown in Figure 5D. Top 22 results of a search in g:Profiler are shown. Complete results are shown in Table S21. The data are related to Figure 5D,E.

GO.ID	Description	padj	Genes
GO:MF			
GO:0000981	DNA-binding transcription factor activity, RNA polymerase II-specific	3.0020889186981556e-16	*PRDM16,SCRT2,MEIS3,HIC1,DMRTB1,PRRX1,TBX18,GLIS1,LHX4,GLI2,HIVEP3,NFATC4,EBF4,ALX4,ELF4,HES3,ZBTB7C,PAX7,HEYL,ZNF536,GLIS2*
GO:0043565	sequence-specific DNA binding	6.766317740279593e-16	*PRDM16,SCRT2,MEIS3,HIC1,DMRTB1,PRRX1,TBX18,GLIS1,LHX4,GLI2,NFATC4,EBF4,ALX4,ELF4,HES3,PAX7,HEYL,ZNF536,GLIS2*
GO:0003700	DNA-binding transcription factor activity	1.6218692524044966e-15	*PRDM16,SCRT2,MEIS3,HIC1,DMRTB1,PRRX1,TBX18,GLIS1,LHX4,GLI2,HIVEP3,NFATC4,EBF4,ALX4,ELF4,HES3,ZBTB7, PAX7,HEYL,ZNF536,GLIS2*
GO:0140110	transcription regulator activity	5.730692260911566e-14	*PRDM16,SCRT2,MEIS3,HIC1,DMRTB1,PRRX1,TBX18,GLIS1,LHX4,GLI2,HIVEP3,NFATC4,EBF4,ALX4,ELF4,HES3,ZBTB7C,PAX7,HEYL,ZNF536,GLIS2*
GO:0003677	DNA binding	1.078772693704691e-10	*PRDM16,SCRT2,MEIS3,HIC1,DMRTB1,PRRX1,TBX18,GLIS1,LHX4,GLI2,HIVEP3,NFATC4,EBF4,ALX4,ELF4,HES3,PAX7,HEYL,ZNF536,GLIS2*
GO:0003676	nucleic acid binding	5.094046931162048e-10	*ADAMTS17,PRDM16,SCRT2,MEIS3,HIC1,DMRTB1,PRRX1,TBX18,GLIS1,LHX4,GLI2,HIVEP3,NFATC4,EBF4,ALX4,ELF4,HES3,ZBTB7C,PAX7,HEYL,ZNF536,DPF3,GLIS2*
GO:0000976	transcription regulatory region sequence-specific DNA binding	3.1980974069268857e-9	*SCRT2,PRRX1,TBX18,GLIS1,GLI2,NFATC4,EBF4,ALX4,ELF4,HES3,HEYL,ZNF536,GLIS2*
GO:1990837	sequence-specific double-stranded DNA binding	6.201300596296805e-9	*SCRT2,PRRX1,TBX18,GLIS1,GLI2,NFATC4,EBF4,ALX4,ELF4,HES3,HEYL,ZNF536,GLIS2*
GO:0044212	transcription regulatory region DNA binding	1.717308909160837e-8	*SCRT2,PRRX1,TBX18,GLIS1,GLI2,NFATC4,EBF4,ALX4,ELF4,HES3,HEYL,ZNF536,GLIS2*
GO:0001067	regulatory region nucleic acid binding	1.7651201530658695e-8	*SCRT2,PRRX1,TBX18,GLIS1,GLI2,NFATC4,EBF4,ALX4,ELF4,HES3,HEYL,ZNF536,GLIS2*
GO:0003690	double-stranded DNA binding	2.223504167721871e-8	*SCRT2,PRRX1,TBX18,GLIS1,GLI2,NFATC4,EBF4,ALX4,ELF4,HES3,HEYL,ZNF536,GLIS2*
GO:0000977	RNA polymerase II regulatory region sequence-specific DNA binding	3.2487899485312494e-8	*SCRT2,PRRX1,GLIS1,GLI2,NFATC4,EBF4,ALX4,ELF4,HES3,HEYL,ZNF536,GLIS2*
GO:0001012	RNA polymerase II regulatory region DNA binding	3.510295518605051e-8	*SCRT2,PRRX1,GLIS1,GLI2,NFATC4,EBF4,ALX4,ELF4,HES3,HEYL,ZNF536,GLIS2*
GO:1901363	heterocyclic compound binding	0.0000018894696087891545	*ADAMTS17,PRDM16,SCRT2,MEIS3,HIC1,DMRTB1,PRRX1,TBX18,GLIS1,LHX4,GLI2,HIVEP3,NFATC4,EBF4,ALX4,ELF4,HES3,ZBTB7C,PAX7,HEYL,ZNF536,DPF3,GLIS2*
GO:0097159	organic cyclic compound binding	0.0000025120194207196536	*ADAMTS17,PRDM16,SCRT2,MEIS3,HIC1,DMRTB1,PRRX1,TBX18,GLIS1,LHX4,GLI2,HIVEP3,NFATC4,EBF4,ALX4,ELF4,HES3,ZBTB7C,PAX7,HEYL,ZNF536,DPF3,GLIS2*
GO:0000978	RNA polymerase II proximal promoter sequence-specific DNA binding	0.00011929626986549908	*SCRT2,PRRX1,GLI2,NFATC4,ELF4,HES3,HEYL,ZNF536*
GO:0000987	proximal promoter sequence-specific DNA binding	0.00014630761558464278	*SCRT2,PRRX1,GLI2,NFATC4,ELF4,HES3,HEYL,ZNF536*
GO:0001227	DNA-binding transcription repressor activity, RNA polymerase II-specific	0.006154396615738856	*SCRT2,HIC1,GLIS1,NFATC4*
GO:0046872	metal ion binding	0.03598926908109106	*ADAMTS17,PRDM16,SCRT2,HIC1,DMRTB1,FBN1,GLIS1,LHX4,GLI2,HIVEP3,EBF4,ZBTB7C,ZNF536,DPF3,GLIS2*
GO:0003712	transcription coregulator activity	0.037118154816446446	*PRDM16,PRRX1,TBX18,NFATC4,HES3,HEYL*
GO:0001216	DNA-binding transcription activator activity	0.03790861793015616	*GLIS1,LHX4,EBF4,ALX4*
GO:0001228	DNA-binding transcription activator activity, RNA polymerase II-specific	0.03790861793015616	*GLIS1,LHX4,EBF4,ALX4*

## Supplementary Materials

The following are available online at https://www.mdpi.com/2073-4409/8/11/1393/s1, Table S1: The list of 4920 rDNA-contacting genes. 4C-rDNA reads were processed as described in Materials and Methods. Related to Figure 1. Table S2: rDNA-contacting genes involved in different biological processes (g:Profiler). Related to Figure 1. Table S3: Overlap of rDNA-contacting genes associated with three biological processes. See Venn Diagram in Figure 1B. Table S4: rDNA-contacting genes associated with Jensen Compartments. Related to Figure 2A. Table S5: Overlap of rDNA-contacting genes associated with three Jensen Compartments. Related to Venn Diagram in Figure 2B. Table S6: rDNA-contacting genes associated with Jensen Diseases. Related to Figure 2C. Table S7: Overlap of rDNA-contacting genes associated with three Jensen Diseases. Related to Venn Diagram in Figure 2D. Table S8: Overlap of rDNA-contacting genes associated with three Jensen Diseases. Related to Venn Diagram in Figure 2E. Table S9: Changes in contact frequencies of different rDNA-contacting genes with rDNA clusters after heat shock treatment. Excel file attached separately. Table S10: Overlap of the selected 4020 rDNA-contacting genes with genes that changed their contacts with rDNA after heat shock treatment. Related to Venn diagram in Figure 4B. Table S11: Genes that increased their contacts with rDNA after heat shock treatment specify TFs that exhibit co-occurrence at a set of 60–70 genes. Related to Venn diagram in Figure 4C. Table S12: Genes that retained their contacts with rDNA after heat shock control the neuronal cell development. Related to Venn diagram in Figure 4D. Table S13: The results of RNA-Seq before (hs-) and after (hs+) heat shock treatment of HEK293T cells (recovery time: 2.5 h). baseMean – mean of the counts divided by the size factors for the counts for both conditions. log2FoldChange – the log2 of the fold change. lfcSE gives the standard error of the log2FoldChange. Stat is the Wald statistic: the log2 fold change divided by lfcSE, which is compared to a standard normal distribution to generate a two-tailed *p*-value. padj—the adjusted *p*-values. Excel file attached separately. Table S14: The results of RNA-Seq before (hs-) and after (hs+) heat shock treatment of HEK293T cells (recovery time: 6 h) for the selected 4920 rDNA-contacting genes. Excel file attached separately). Table S15: Overlapping of rDNA-contacting genes that decrease or increase the number of their contacts with rDNA clusters and change RNA expression. Related to Venn diagram in Figure 5B, downregulated rDNA-contacting genes. Table S16: GO associations (Enrichr Submissions TF-Gene Cooccurrence) of 101 rDNA-contacting genes that were downregulated after heat shock treatment and kept their contacts with rDNA. Related to Venn diagram in Figure 5B, 101 genes, and Figure 5C, enriched terms. Table S17: Overlap of rDNA-contacting genes that decrease or increase the number of their contacts with rDNA clusters and change RNA expression. Related to Venn diagram in Figure 5D, upregulated rDNA-contacting genes. Table S18: GO associations of rDNA-contacting genes that were upregulated after heat shock treatment and increase their contacts with rDNA. Related to Venn diagram in Figure 5D, 10 genes. Table S19: GO associations (Enrichr Submissions TF-Gene Cooccurrence) of 123 rDNA-contacting genes that were upregulated after heat shock treatment and kept their contacts with rDNA. Related to Venn diagram in Figure 5D, 123 genes, and Figure 5E, enriched terms. Table S20: GO associations of genes that are co-regulated by 123 rDNA-contacting genes shown in Figure 5E, enriched terms. Figure S1: The distribution of 4920 selected rDNA-contacting genes on human chromosomes.

## References

[1] Moss T., Langlois F., Gagnon-Kugler T., Stefanovsky V. (2017). A housekeeper with power of attorney: The rRNA genes in ribosome biogenesis. Cell. Mol. Life Sci..

[2] Ananiev E.V., Barsky V.E., Ilyin Y.V., Churikov N.A. (1981). Localization of nucleoli in Drosophila melanogaster polytene chromosomes. Chromosoma.

[3] Worton R.G., Sutherland J., Sylvester J.E., Willard H.F., Bodrug S., Dube I., Duff C., Kean V., Ray P.N., Schmickel R.D. (1988). Human ribosomal RNA genes: Orientation of the tandem array and conservation of the 5′end. Science.

[4] Santoro R., Grummt I. (2005). Epigenetic mechanism of rRNA gene silencing: Temporal order of NoRC-mediated histone modification, chromatin remodeling, and DNA methylation. Mol. Cell. Biol..

[5] Guetg C., Lienemann P., Sirri V., Grummt I., Hernandez-Verdun D., Hottiger M.O., Fussenegger M., Santoro R. (2010). The NoRC complex mediates the heterochromatin formation and stability of silent rRNA genes and centromeric repeats. EMBO J..

[6] Guetg C., Scheifele F., Rosenthal F., Hottiger M.O., Santoro R. (2012). Inheritance of silent rDNA chromatin is mediated by PARP1 via noncoding RNA. Mol. Cell.

[7] Savić N., Bär D., Leone S., Frommel S.C., Weber F.A., Vollenweider E., Ferrari E., Ziegler U., Kaech A., Shakhova O. (2014). lncRNA maturation to initiate heterochromatin formation in the nucleolus is required for exit from pluripotency in ESCs. Cell Stem Cell.

[8] Van Koningsbruggen S., Gierliński M., Schofield P., Martin D., Barton G.J., Ariyurek Y., den Dunnen J.T., Lamond A.I. (2010). High-resolution whole genome sequencing reveals that specific chromatin domains from most human chromosomes associate with nucleoli. Mol. Biol. Cell..

[9] Németh A., Conesa A., Santoyo-Lopez J., Medina I., Montaner D., Péterfia B., Solovei I., Cremer T., Dopazo J., Längst G. (2010). Initial genomics of the human nucleolus. PLoS Genet..

[10] Pontvianne F., Carpentier M.C., Durut N., Pavlištová V., Jaške K., Schořová Š., Parrinello H., Rohmer M., Pikaard C.S., Fojtová M. (2016). Identification of Nucleolus-Associated Chromatin Domains Reveals a Role for the Nucleolus in 3D Organization of the A. thaliana Genome. Cell Rep..

[11] Dillinger S., Straub T., Németh A. (2017). Nucleolus association of chromosomal domains is largely maintained in cellular senescence despite massive nuclear reorganisation. PLoS ONE.

[12] Tchurikov N.A., Fedoseeva D.M., Sosin D.V., Snezhkina A.V., Melnikova N.V., Kudryavtseva A.V., Kravatsky Y.V., Kretova O.V. (2015). Hot spots of DNA double-strand breaks and genomic contacts of human rDNA units are involved in epigenetic regulation. J. Mol. Cell. Biol..

[13] Tchurikov N.A., Kretova O.V., Fedoseeva D.M., Sosin D.V., Grachev S.A., Serebraykova M.V., Romanenko S.A., Vorobieva N.V., Kravatsky Y.V. (2013). DNA double strand breaks coupled with PARP1 and HNRNPA2B1 binding sites flank coordinately expressed domains in human chromosomes. PLoS Genet..

[14] Dekker J., Rippe K., Dekker M., Kleckner N. (2002). Capturing chromosome conformation. Science.

[15] Osborne C.S., Chakalova L., Brown K.E., Carter D., Horton A., Debrand E., Goyenechea B., Mitchell J.A., Lopes S., Reik W. (2004). Active genes dynamically colocalize to shared sites of ongoing transcription. Nat. Genet..

[16] FastQC. https://www.bioinformatics.babraham.ac.uk/projects/fastqc.

[17] Cutadapt. https://cutadapt.readthedocs.io.

[18] Langmead B., Salzberg S.L. (2012). Fast gapped-read alignment with Bowtie 2. Nat. Methods.

[19] Li H., Durbin R. (2010). Fast and accurate long-read alignment with Burrows-Wheeler transform. Bioinformatics.

[20] BWA 0.7.12 method mem. http://bio-bwa.sourceforge.net.

[21] Li H., Handsaker B., Wysoker A., Fennell T., Ruan J., Homer N. (2009). 1000 Genome Project Data Processing Subgroup. The Sequence Alignment/Map format and SAMtools. Bioinformatics.

[22] Samtools 1.6. http://www.htslib.org.

[23] Ramírez F., Ryan D.P., Grüning B., Bhardwaj V., Kilpert F., Richter A.S., Heyne S., Friederike Dündar F., Manke T. (2016). DeepTools2: A next generation web server for deep-sequencing data analysis. Nucleic Acids Res..

[24] Quinlan A.R. (2014). BEDTools: The Swiss-Army Tool for Genome Feature Analysis. Curr. Protoc. Bioinformatics.

[25] Klein F.A., Pakozdi T., Anders S., Ghavi-Helm Y., Furlong E.E., Huber W. (2015). FourCSeq: Analysis of 4C sequencing data. Bioinformatics.

[26] Liao Y., Smyth G.K., Shi W. (2014). FeatureCounts: An efficient general purpose program for assigning sequence reads to genomic features. Bioinformatics.

[27] Love M.I., Huber W., Anders S. (2014). Moderated estimation of fold change and dispersion for RNA-seq data with DESeq2. Genome Biol..

[28] EnhancedVolcano R package. https://github.com/kevinblighe.

[29] Bolger A.M., Lohse M., Usadel B. (2014). Trimmomatic: A flexible trimmer for Illumina sequence data. Bioinformatics.

[30] Zhang Y., Liu T., Meyer C.A., Eeckhoute J., Johnson D.S., Bernstein B.E., Nusbaum C., Myers R.M., Brown M., Li W. (2008). Model-based analysis of ChIP-Seq (MACS). Genome Biol..

[31] Stempor P., Ahringer J. (2016). SeqPlots-Interactive software for exploratory data analyses: Pattern discovery and visualization in genomics. Wellcome Open Res..

[32] Bioinformatics. http://bioinformatics.psb.ugent.be/webtools/Venn.

[33] Dobin A., Davis C.A., Schlesinger F., Drenkow J., Zaleski C., Jha S., Batut P., Chaisson M., Gingeras T.R. (2013). STAR: Ultrafast universal RNA-seq aligner. Bioinformatics.

[34] Reimand J., Kull M., Peterson H., Hansen J., Vilog J.G. (2007). Profiler—A web-based toolset for functional profiling of gene lists from large-scale experiments. Nucleic Acids Res..

[35] Shaw G., Morse S., Ararat M., Graham F.L. (2002). Preferential transformation of human neuronal cells by human adenoviruses and the origin of HEK 293 cells. FASEB J..

[36] Wu Q., Maniatis T. (1999). A striking organization of a large family of human neural cadherin-like cell adhesion genes. Cell.

[37] Kuleshov M.V., Jones M.R., Rouillard A.D., Fernandez N.F., Duan Q., Wang Z., Koplev S., Jenkins S.L., Jagodnik K.M., Lachmann A. (2016). Enrichr: A comprehensive gene set enrichment analysis web server 2016 update. Nucleic Acids Res..

[38] Graham F.L., Smiley J., Russell W.C., Nairn R. (1977). Characteristics of a human cell line transformed by DNA from human adenovirus type 5. J. Gen. Virol..

[39] Karmodiya K., Krebs A.R., Oulad-Abdelghani M., Kimura H., Tora L. (2012). H3K9 and H3K14 acetylation co-occur at many gene regulatory elements, while H3K14ac marks a subset of inactive inducible promoters in mouse embryonic stem cells. BMC Genom..

[40] Macias S., Plass M., Stajuda A., Michlewski G., Eyras E., Cáceres J.F. (2012). DGCR8 HITS-CLIP reveals novel functions for the Microprocessor. Nat. Struct. Mol. Biol..

[41] Ono T., Losada A., Hirano M., Myers M.P., Neuwald A.F., Hirano T. (2003). Differential contributions of condensin I and condensin II to mitotic chromosome architecture in vertebrate cells. Cell.

[42] Rao S.S., Huang S.C., St Hilaire B.G., Engreitz J.M., Perez E.M., Kieffer-Kwon K.R., Sanborn A.L., Johnstone S.E., Bascom G.D., Bochkov I.D. (2017). Cohesin Loss Eliminates All Loop Domains. Cell.

[43] Zhao Z., Dammert M.A., Hoppe S., Bierhoff H., Grummt I. (2016). Heat shock represses rRNA synthesis by inactivation of TIF-IA and lncRNA-dependent changes in nucleosome positioning. Nucleic Acids Res..

[44] Zhao Z., Sentürk N., Song C., Grummt I. (2018). lncRNA PAPAS tethered to the rDNA enhancer recruits hypophosphorylated CHD4/NuRD to repress rRNA synthesis at elevated temperatures. Genes Dev..

[45] Humburg P., Maugeri N., Lee W., Mohr B., Knight J.C. (2016). Characterisation of the global transcriptional response to heat shock and the impact of individual genetic variation. Genome Med..

[46] Kraushar M.L., Viljetic B., Wijeratne H.R., Thompson K., Jiao X., Pike J.W., Medvedeva V., Groszer M., Kiledjian M., Hart R.P. (2015). Thalamic WNT3 Secretion Spatiotemporally Regulates the Neocortical Ribosome Signature and mRNA Translation to Specify Neocortical Cell Subtypes. J. Neurosci..

[47] Kraushar M.L., Popovitchenko T., Volk N.L., Rasin M.R. (2016). The frontier of RNA metamorphosis and ribosome signature in neocortical development. Int. J. Dev. Neurosci..

[48] Chau K.F., Shannon M.L., Fame R.M., Fonseca E., Mullan H., Johnson M.B., Sendamarai A.K., Springel M.W., Laurent B., Lehtinen M.K. (2018). Downregulation of ribosome biogenesis during early forebrain development. ELife.

[49] Ensembl FTP server. http://ftp.ensembl.org/pub/grch37/current/gtf.

[50] Hnisz D., Abraham B.J., Lee T.I., Lau A., Saint-André V., Sigova A.A., Hoke H.A., Young R.A. (2013). Super-enhancers in the control of cell identity and disease. Cell.

[51] Spellman P.T., Rubin G.M. (2002). Evidence for large domains of similarly expressed genes in the Drosophila genome. J. Biol..

[52] Tolhuis B., Muijrers I., de Wit E., Teunissen H., Talhout W., van Steensel B., van Lohuizen M. (2006). Genome-wide profiling of PRC1 and PRC2 Polycomb chromatin binding in Drosophila melanogaster. Nat. Genet..

[53] Le Dily F., Baù D., Pohl A., Vicent G.P., Serra F., Soronellas D., Castellano G., Wright R.H., Ballare C., Filion G. (2014). Distinct structural transitions of chromatin topological domains correlate with coordinated hormone-induced gene regulation. Genes Dev..

[54] Tchurikov N.A. (2005). Molecular mechanisms of epigenetics. Biochemistry.

[55] Lewis E.B. (1954). The theory and application of a new method of detecting chromosomal rearrangements in Drosophila melanogaster. Am. Nat..

[56] Feinberg A.P. (2014). The Nucleolus Gets the Silent Treatment. Cell Stem Cell.

[57] Richter K., Haslbeck M., Buchner J. (2010). The heat shock response: Life on the verge of death. Mol. Cell.

[58] King I.F., Yandava C.N., Mabb A.M., Hsiao J.S., Huang H.S., Pearson B.L., Calabrese J.M., Starmer J., Parker J.S., Magnuson T. (2013). Topoisomerases facilitate transcription of long genes linked to autism. Nature.

[59] Sugino K., Hempel C.M., Okaty B.W., Arnson H.A., Kato S., Dani V.S., Nelson S.B. (2014). Cell-type-specific repression by methyl-CpG-binding protein 2 is biased toward long genes. J. Neurosci..

[60] Yu S., Lemos B. (2018). The long-range interaction map of ribosomal DNA arrays. PLoS Genet..

[61] Percharde M., Lin C.J., Yin Y., Guan J., Peixoto G.A., Bulut-Karslioglu A., Biechele S., Huang B., Shen X., Ramalho-Santos M. (2018). A LINE1-nucleolin partnership regulates early development and ESC identity. Cell.

[62] Kretova O.V., Fedoseeva D.M., Kravatsky Y.V., Alembekov I.R., Slovohotov I.Y., Tchurikov N.A. (2019). Homeotic DUX4 Genes that Control Human Embryonic Development at the Two-Cell Stage Are Surrounded by Regions Contacting with rDNA Gene Clusters. Mol. Biol..

[63] Pelkmans L. (2012). Cell Biology. Using cell-to-cell variability–A new era in molecular biology. Science.

[64] Boija A., Klein I.A., Sabari B.R., Dall’Agnese A., Coffey E.L., Zamudio A.V., Li C.H., Shrinivas K., Manteiga J.C., Hannett N.M. (2018). Transcription Factors Activate Genes through the Phase-Separation Capacity of Their Activation Domains. Cell.

[65] Sabari B.R., Dall’Agnese A., Boija A., Klein I.A., Coffey E.L., Shrinivas K., Abraham B.J., Hannett N.M., Zamudio A.V., Manteiga J.C. (2018). Coactivator condensation at super-enhancers links phase separation and gene control. Science.

[66] Latonen L. (2019). Phase-to-Phase with Nucleoli-Stress Responses, Protein Aggregation and Novel Roles of RNA. Front. Cell Neurosci..

[67] Feinberg A.P., Tycko B. (2004). The history of cancer epigenetics. Nat. Rev. Cancer.

[68] Feinberg A.P., Koldobskiy M.A., Göndör A. (2016). Epigenetic modulators, modifiers and mediators in cancer aetiology and progression.Nat Rev Genet. Nat. Rev. Genet..

[69] Diesch J., Bywater M.J., Sanij E., Cameron D.P., Schierding W., Brajanovski N., Son J., Sornkom J., Hein N., Evers M. (2019). Changes in long-range rDNA-genomic interactions associate with altered RNA polymerase II gene programs during malignant transformation. Commun. Biol..

[70] Weeks S.E., Metge B.J., Samant R.S. (2019). The nucleolus: A central response hub for the stressors that drive cancer progression. Cell. Mol. Life Sci..

