# Supplementary material for: rDNA Clusters Make Contact with Genes that Are Involved in Differentiation and Cancer and Change Contacts after Heat Shock Treatment
Source: Cells. 2019 Nov 5;8(11):1393. doi: 10.3390/cells8111393 (PMC6912461; doi:10.3390/cells8111393)
Supplement: Supplementary file 1 [file cells-08-01393-s001.zip › cells-610496-SI/cells-610496-proof-supplementary.pdf]

Supplementary Materials for

rDNA clusters make contact with genes that are involved in differentiation and cancer, and change the contacts after heat shock treatment

Nickolai A. Tchurikov*, Daria M. Fedoseeva, Elena S. Klushevskaya, Ivan Y. Slovohotov, Ildar R. Alembekov, Vladimir R. Chechetkin, Yuri V. Kravatsky, Olga V. Kretova

Department of Epigenetic Mechanisms of Gene Expression Regulation, Engelhardt Institute of Molecular Biology Russian Academy of Sciences, Moscow, 119334, Russia

*To whom correspondence should be addressed. Tel: 7-499-1359753; Fax: 7-499-1351405; Email: tchurikov@eimb.ru

This file includes

Supplementary Text
Supplementary Methods
Supplementary References
Supplementary Tables S1-S20
Supplementary Figure S1

Supplementary Text

To determine the real bias of the selected rDNA-contacting genes within the genome, the following procedure was performed. The list of the *H. sapiens* genes for the hg19/GRCh37 build genome was obtained from UCSC by the following command: mysql --user=genome -N --host=genome-mysql.cse.ucsc.edu -A -D hg19 -e "select ensGene.name, name2, chrom, strand, txStart, txEnd, value from ensGene, ensemblToGeneName where ensGene.name = ensemblToGeneName.name" > genes.hg19.txt and then converted to a common table by an *in hoc* Perl script.

The next procedure applied was deduplication, i.e., after this procedure only one encounter of each gene in the list was kept and the gene length is the mean length of all gene copies. This procedure diminishes the influence of multiple gene copies to the gene length statistics because, in the GO analysis, all the software packages use only one gene encounter in the list. We calculated the basic statistics for all the 4C-rDNA-contacting genes and for the selected top 4920 4C-rDNA-contacting genes subset.

The simple analysis demonstrates that the full "official" genes list is, at least, questionable because it has "genes" with a length of three nucleotides and additional filtering and/or curation should probably be applied. For that list, we see that the median gene length of 4C-rDNA-contacting genes is approximately twice that of genes in the full list (17,863 bp vs. 9538 bp) while 95% of all genes in the genome and in the list of 4C-rDNA-contacting genes have similar distribution limits (genome: [249,

144103], 4C rDNA: [127, 140634]). The top 4C-rDNA-contacting genes (4920 genes) have a median length that is approximately 3.4 times longer than the genome genes (32,146 bp vs. 9538 bp) and a twofold longer upper distribution tail (275,213 bp vs. 144,103 bp). Finally, we visualize all the distributions using a ggplot2 violin + boxplot graph (Figure 7).

Supplementary Methods

4C procedure

DNA samples were prepared as described previously (Dekker et al., 2002; Osborne et al., 2004; http://www.protocol-online.org/cgi-bin/prot/view_cache.cgi?ID=3978). To a suspension containing about 20×10^6 HEK293T cells in 40 mL of DMEM, formaldehyde solution was added to final concentration 1.5%. After mixing, incubation of cell suspension was performed for 10 min at room temperature with mixing. Quenching with 2.75 mL of 2 M glycine (final concentration 0.125 M) was performed. After incubation at room temperature for 5 min the suspension was cooled for 15 min in an ice bath and then cells were collected by centrifugation for 15 min at 3500 rpm at 2°C. The pellet of cells was resuspended at 0 °C in 1 mL of buffer containing 10 mM Tris-HCl buffer, pH 8, 10 mM NaCl, 0.2% NP-40, and freshly added protease inhibitors (0.1 mM PMSF and 1:500 protease inhibitor cocktail (Sigma)). After incubation for 15 min, cells were homogenized by passing through a syringe about 50 times into an Eppendorf. Then nuclei were spun down by centrifugation for 5 min at 5000 rpm in an Eppendorf centrifuge 5415 R at 2°C.

The nuclei pellet was resuspended in 756 µL of solution containing 40 mM Tris-HCl buffer, pH 7.4, 50 mM NaCl, 10 mM MgCl2 and 10 mM 2-mercaptoethanol. Then 20% SDS was added to final concentration 0.3% and incubation with shaking was performed for 1 h at 37 °C. To sequester SDS, 180 µL of 10% Triton X-100 was added and the solution was incubated for 1 h at 37 °C. Before digestion, 1 µL of BSA (5 mg/mL) was added with mixing. Then 50 µL of EcoRI (10 u/µL) was added and after mixing digestion was performed overnight at 37 °C.

To inactivate the restriction enzyme, 35 µL of 20% SDS was added (final concentration 0.7%) and the probe was heated to 65 °C for 30 min. Then the mixture was transferred into a 15 mL Nunc tube and consequently 375 µL of 20% Triton X-100 (to final concentration 1%), 750 µL of 10× ligase buffer, 7.5 µL of BSA (5 mg/mL), 80 µL of 100 mM ATP, and 5241 µL of milliQ water were added and the final 7.5 mL solution was well mixed. Then 10 µL of T4 DNA ligase (200 u/µL) was added and after mixing incubation was performed for 5 h at 16 °C and then for 30 min at room temperature. During ligation, DNA concentration was equal to 2 ng/µL.

For isolation of DNA, 50 µL of proteinase K (10 mg/mL) was added (final concentration 50 µg/mL) and, after mixing, incubation was performed at 55 °C overnight. For RNA digestion, 40 µL of RNase A (10 mg/mL) was added (final concentration 0.5 µg/mL) and, after mixing, incubation was performed for 30 min at 37 °C. After extensive extraction with phenol-chloroform extraction (three

times with 7 mL each) DNA was precipitated by 2.5 vol. of ethanol after addition of 40 μL 10 mg/ glycogen and 175 μL 4 M NaCl. The final DNA pellet was washed twice with 70% ethanol and then dissolved in 0.1×TE.

About 15 μg of DNA was digested in 250 μL solution with 75 u of FaeI overnight at 37 °C. Then the enzyme was inactivated by heating at 65 °C for 30 min. DNA was isolated after phenol-chloroform extraction, precipitated by ethanol, and dissolved in 100 μL of 0.1×TE.

For circularization, 15 μg of DNA was incubated in 8 mL of T4 DNA ligase buffer containing 400 u of T4 DNA ligase for 5 h at 16 °C. DNA was isolated after phenol-chloroform extraction, precipitated by ethanol, and dissolved in 50 μL of 0.1×TE.

One or two rounds of PCR were used as described below. There are about 300-400 copies of rDNA, which is why a single round of PCR (with up to 35 cycles) was found to be sufficient. DNA concentration was titrated and finally about 30 ng of DNA was used for PCR with primers 5’ TCTTTGAAAAAAATCCCAGAAGTGGT 3’ and 5’ AAGTCCAGAAATCAACTCGCCAGT 3’ (for PCR-1), and 5’ GCCTAAGCCTGCTGAGAACTTTC 3’ and 5’ CAGCATTCTGTAGGGAGATCAAATC 3’ (for PCR-2). After separation in 2% agarose gels, two DNA fractions (200-400 bp and higher than 400 bp) were eluted using a QIAquick gel extraction kit (Qiagen). The libraries were prepared using TruSeq RNA Sample Preparation Kit v. 2 (Illumina) using adapter AR006 for 200-400 bp DNA fraction and adapter R007 for DNA fractions higher than 400 bp. The samples were sequenced using MiSeq (Illumina).

Statistical threshold for 4C reads

The number of covering reads associated with 4C contacts should exceed a statistical threshold related to the instrumental errors in reads to be genetically meaningful. Here, we present some estimates for this problem. Each sequenced read is characterized by the quality parameter Q (assessed by software supplied by the developers of the instrumental technique for deep sequencing). The probability of a reading error per nucleotide is related to the quality parameter as:

$$p_e = 10^{-Q/10} \qquad \text{(S.1)}$$

Furthermore, each read nucleotide is covered additionally by n_c fragments for the cross-check. Then, at a particular site, the consensus is determined corresponding to covering. If more than half of the nucleotides in the corresponding site of the covering fragments is read incorrectly, the consensus would also be read incorrectly. The probability of such an event is given by:

$$p_e(n_c) = \sum_{n=n_c/2}^{n_c} \binom{n}{n_c} p_e^n (1-p_e)^{n_c-n};$$
$$\binom{n}{n_c} = \frac{n_c!}{n!(n_c-n)!} \qquad \text{(S.2)}$$

The probability of at least one error in a read fragment of length *L* is expressed by (S.2) as:

$$p_e(n_c, L) = 1 - (1 - p_e(n_c))^L \approx L\, p_e(n_c) \qquad \text{(S.4)}$$

whereas the probability of at least one error in a set of N_r fragments of length *L* can be similarly assessed as:

$$p_e(n_c, L; N_r) \approx N_r L\, p_e(n_c) \qquad \text{(S.5)}$$

The resulting probabilities in r. h. s. of Eqs. (S.4) and (S.5) correspond to the Bonferroni approximation of the extreme value statistics and should be small by their applicability. The standard statistical thresholds for the probability $p_e(n_c, L; N_r)$ are $p = 0.05$ and 0.01. Thus, Eq. (S.5) provides the estimate for the threshold number of covering reads n_c depending on the quality parameter *Q*, the length of reads *L*, and the total number of reads in the data set N_r. The genetically meaningful data should satisfy this restriction. In particular, for $N_r = 10^7$; $L = 100$; $p_e(n_c, L; N_r) = 0.01$, the threshold coverage n_c should exceed 11 for the quality parameter $Q = 20$ and 8 for $Q = 30$. These thresholds were exceeded several times in our experiments ($n_c > 40$).

Supplementary References

Dekker, J., Rippe, K., Dekker, M., et al. (2002). Capturing chromosome conformation. Science 95, 1306–1311.

Osborne, C.S., Chakalova, L., Brown, K.E., et al. (2004). Active genes dynamically colocalize to shared sites of ongoing transcription. Nat. Genet. 36, 1065–1071.

Supplementary Tables S1-S20

Table S1. The list of 4920 rDNA-contacting genes. 4C-rDNA reads were processed as described in Materials and Methods. Related to Figure 1.

Gene name
CDC27P2 LINC00273 PCMTD1P2 RNA5-8SP2 IGHV1OR21-1 ROCK1P1 DUX4L16 DUX4L17 DUX4L18 DUX4L19 PABPC1P5 SLC9B1P1 CTBP2P1 VN1R7P ANKRD20A17P CDK2AP2P3 LINC00537 MIR4477A PTGER4P3 RNA5SP284 TEKT4P2 FRG2C LINC00960 DHX32 FANK1 RNU2-42P MIR3648 MIR3687 RNA5SP519 KMT2C CLUHP4 DUX4 DUX4L2 DUX4L3 DUX4L4 DUX4L5

DUX4L6 DUX4L7 DUX4L8 ISM1 TASP1 CLUHP5 DUX4L10 DUX4L11 DUX4L12 DUX4L13 DUX4L14 DUX4L15 RPL23AP60
BAGE2 CYCSP41 TPTE MIR3118-3 RNA5-8SP6 CAST OR11H13P ADCY2 FRG1B MLLT10P1 RNA5SP283 SRGAP2B RANGAP1
MIR3118-2 ELP4 MACROD2 MACROD2-IT1 RN7SL864P AGGF1P3 RARRES2P1 BNIP3P2 EIF3FP1 CDH4 ANKRD30BL CDC27P1
MIR663B RNA5-8SP5 RNU6-1132P COL24A1 MIR1299 ANKRD20A14P PIEZO2 MIR4273 RN7SL92P ZNF717 SNX18P25
SLC25A15P4 CMSS1 FILIP1L MIR1324 ZNF595 ZHX3 DUX4L9 FRG2 ANKRD20A12P MIR3118-1 SNX18P15 ZPLD1 CDK2AP2P1
THSD4 BMS1P9 AGGF1P2 FRG2B RARRES2P2 BMS1P11 PLCB1 PLCB1-IT1 DRD5P1 HFM1 NKAIN1P1 ANKRD36BP2 NMD3P1
ANKRD36 PCDH9 RNU7-87P SLX4IP DHX35 hsa-mir-6723 MTATP6P1 MTATP8P1 MTND1P23 MTND2P28 OR4F16 KHDRBS2
CDK2AP2P2 PTGER4P2 AGGF1P1 RARRES2P4 CDKN2B-AS1 MTAP C2orf27A C2orf27B CMKLR1 RNA5SP518 TSPY5P C22orf34
MIR3667 NCOR1P3 DAB1 OMA1 RPS20P5 CDC42EP3 SLC9B1P2 Y_RNA SLC16A12 TRIM48 GTF2I GTF2IRD2 STAG3L2 GTF2IP1
RNU6-576P PCMTD1P1 ACTR3BP6 SLC9B1P4 STXBP5L FAM19A5 MIR4535 LDB3 DSCAM DSCAM-IT1 MIR4760 AGBL1 FMNL2
ABCD1P4 CHEK2P4 GAB4 SLC25A15P5 TPTEP1 BNIP3P3 GABRB3 ABCC10 WDR47 SNORA70 GABRG3 ANKRD30BP2 RNU6-
614P FGF7P2 ACTR3BP5 SLC9B1P3 ANKRD36C MAPK14 TMEM101 U3 DRD5P2 ABCG8 ZSCAN10 ACTR3BP2 ANKRD30BP1
MIR3156-3 POLR3K ANKRD20A4 RNU6-1193P NCOR1P2 RNA5SP359 DTNA ITGAM ASNSP5 PON1 PON3 YME1L1P1
ANKRD20A7P MED15P7 NF1P6 ZNF91 DUXAP8 NBEAP3 TRIM51CP TBC1D2 ARID1B GTF2IP3 PHF2P2 USP24P1 VN1R91P
TMEM108 GPHN MTND1P12 DUXAP10 RNU6-458P CYCSP32 TPTE2 MIR663A NF1P4 BICD1 ACTR3BP3 PGM5P2 REXO1L10P
REXO1L11P REXO1L12P REXO1L2P REXO1L9P CKS1BP5 KCTD16 ARHGAP42P4 TRAPPC8 CEP44 FRAS1 CHODL ANKRD36B
ARHGAP42P3 MLLT10P2 RN7SL52P IGKV1OR22-5 ENTHD1 C21orf91 C21orf91-OT1 ANKRD20A5P RNU6-316P NCOR1P1
HAPLN3 CTBP2P4 ZNF355P PCSK2 SGCZ MIR4461 MTND4P12 MTND5P11 MTND6P4 PCBD2 ZPBP POLR3H IGHV1OR15-6
IGHV3-63 IGHV3-64 IGHV3-65 IGHVII-62-1 IGHVII-65-1 FAM182A ANKRD20A9P ANKRD26P1 HNRNPA1L2 MRPS31P4
LINC00349 MSANTD2P1 FAM182B EZH2P1 FKSG68 RPL34P3 RPS20P1 RUNX1 SORBS2 FOXD4L5 ARHGAP42P5 GADL1
ZRANB1 EREG CNN2P12 GXYLT1P1 ZNF965P BNIP3P7 ZNF962P ASIC2 NCAM2 RNA5SP438 TLK2P1 SH3RF3 SNRPGP9 POTEH
ORC3 CAMTA1 HERC2P3 GRAMD4P5 MIR3118-6 POTEB2 RNU6-749P ANKRD20A8P RNU6-1320P RNU6-721P OR4H6P OR4M2
OR4Q1P snoU13 DLX6-AS1 7SK POTEC RNU6-1021P PGM5P1 MYO18B FAM207CP IL21-AS1 POTED RNU6-286P IGKV2OR22-3
IGKV2OR22-4 IGKV3OR22-2 CCNB1IP1P2 WFDC8 IGHV1OR15-9 KRTAP19-10P PEAK1 MIR548X IGHV1OR15-3 IGHV1OR15-4
IGHV4OR15-8 SUZ12 CTBP2 ANXA8L1 LINC00842 NPY4R SAMSN1-AS1 RNU6-410P SERPINA1 SERPINA2P MTND1P17 DET1
BMS1P18 MIR3118-4 POTEB RNU6-631P LINC00898 TSHZ2 LINC00930 CCNG2 HERC2P8 PPIAP22 MIR548U PRIM2 KCNJ6
SYNDIG1 BMS1P10 BMS1P17 PTPRT ANKRD20A1 RNU6-368P RNU1-51P BMS1P14 NEK2P2 SCAMP1 NR3C2 HERC2P4 ELOVL7
DSCR10 DSCR8 KCNJ15 hsa-mir-3171 RNU6-978P PPIAP1 CROCC PWRN1 ANKRD20A2 FAM95B1 PAK7 RNU6-1269P SNX18P8
ANKRD20A3 RNU6-538P STXBP6 KCNA3 TMPRSS15 BLZF2P RAB27A HUNK CACNA1E RN7SKP147 RNU6-772P ZNF402P
OR11H12 ALK RN7SL516P IGHV3-30-2 IGHV3-32 IGHV4-31 IGHVII-30-1 IGHVII-31-1 PDE6G SHISA9 IGHV1OR15-2 SLC20A1P3
HSPE1P19 HSF2BP IGLV2-8 IGLV3-7 MIR650 LINC00911 CYCSP34 TPTE2P5 STARD13 LYPD6 TPTE2P1 PSPC1P2 TPTE2P6
ANO2 NBEA NTRK3 PHF21B MIR548F1 PCDH15 MYO5BP2 HERC4 HS3ST4 MIR1185-1 MIR1185-2 MIR134 MIR154 MIR300
MIR323B MIR376A1 MIR376C MIR381 MIR381HG MIR382 MIR485 MIR487A MIR487B MIR539 MIR544A MIR548W MIR654
MIR655 MIR889 RNA5SP405 PAH SYT16 CTBP2P5 OFCC1 UNC5D CYP4F29P ZNF114P1 RNA5SP497 SYN3 TIMP3 MIR4533
PWAR6 SNHG14 SNORD109A SNORD115 SNORD116-16 SNORD116-17 SNORD116-18 SNORD116-19 SNORD116-26 SNORD116-
27 SNRPN SNURF ANK3 SDK2 HCN1 ANKRD30B IGSF5 IGKV1OR2-2 DTD2 HEATR5A NOL4 KCTD1 CCDC144NL RBFOX1
snoMe28S-Am2634 LRFN5 POTEA ABCC13 CA10 DCC FSTL4 hsa-mir-4528 SLC24A3 ADAM2 RNU6-498P BCL2L13 DSCR4
DSCR4-IT1 SVEP1 MIR17HG TXNDC16 RPL8P2 CAPN3 GANC DLC1 FEM1AP1 OR4Q2 C21orf49 C21orf62 LARGE LARGE-IT1
MIR4764 SNORA76 CST9L DEC1 FGFR3P5 RN7SL609P PTPN11 VAV2 PPP2R4 RNU4-45P TBC1D22A SCARA5 C2CD2 SLC1A2
SNORD112 APBA2 PPP1R26P5 TERF1P1 CDH13 INTS4L1 MIR3182 STRADB TRAK2 ZNF112 ZNF229 ZNF285 RYR3 MIR155HG
TTC28 CECR2 TMC4 POTEG RNU6-1239P LINC00365 CDR2 OR4A42P ZNF397 ZSCAN30 RIMS2 DUSP22 FAM3B TACR3 ARNT2
C12orf40 OR10Z1 AKAP13 C12orf42 RNU6-1280P CNTLN ERG FBLN1 SNX31 SNX19P1 FAM201B LINC00162 VPS13D ZBED4
GRIK1 GRIK1-AS1 LOXHD1 MGAT4C OR4A9P CRNN HMGN3P1 ZNF292 LMCD1-AS1 POTEM GPR139 RNA5SP478 ZNF863P
DKKL1P1 ACAD11 FAF1 NPHP3 CTNNA2 OR4A5 RGS6 SPON1 ABCA11P ZNF721 ITGA2 TMPRSS3 UBASH3A MIR4307 THSD7B
CALN1 TTLL2 GABBR2 ANKRD20A11P RNU6-954P MDM2 OCA2 ABCG1 RNA5SP492 ST6GALNAC3 TMPRSS2 INPP5F MAS1LP1
TTLL8 ADAMTSL3 CLIC6 CHRFAM7A CCR1 CCR3 KCNQ1 KCNQ1OT1 RHOJ DNAL4 DPF3 SUN2 TMEM45B NUBPL
ANKRD62P1 ANKRD62P1-PARP4P3 CBFA2T2 PARP4P3 PPP1R26P2 VWFP1 OR4K15 DNM1P50 PRSS23 ULK4P2 EGFLAM
MYO5BP1 OR4H12P OR4M1 OR4N2 ATF7IP DNM1P32 GOLGA8K ULK4P1 PRDM11 EPHA6 IGHV3-47 LINC00221 TBC1D30
INADL MIR3116-2 IGHV3-41 IGHVII-40-1 TMEM117 C1orf173 GGT3P ARHGAP20 ENPP7P10 C22orf39 HIRA ZBTB20 SHC3
TRPM2 VRK2 FCRL2 GRIN3A WBSCR17 ZNF578 OR5J2 C10ORF68 CCDC7 IL4R CNTN5 GPC5 GPC5-IT1 LCE2A LCE4A
MIR548AS NRXN1 CACNG3 MEG9 MIR369 MIR410 MIR656 OR4C12 C14orf177 M1AP CTNND2 RIMS1 SYT6 CTBP2P8 DNM1P28
GNG12-AS1 GOLGA8J NRXN3 RN7SL673P WLS ISX FPGT FPGT-TNNI3K IGKV1OR-1 INO80 LINC00856 LRRC53 S100A7L2
TNNI3K TPK1 GABRA3 MIR105-1 MIR105-2 MIR767 FAM230B DOK5 SGSM1 C14orf144 MX1 CNN2P7 GXYLT1P2 EDDM3B BCL9
SCGB2B2 CES5A INTS4L2 MDGA2 NLRP13 RPL13AP2 RPS15AP3 SCAPER FTCDNL1 GOLGA1 RNU4-82P SCAI KCNE1 RAB5A
ISCA1P3 LINC00504 SNORA51 CPPED1 NKAIN2 RN7SL23P SDHAF2 ZMYM2 COL23A1 CYP4F31P FAR2P4 PLEKHB2 POTEE
RN7SL646P RNU6-127P SLC9B1 SNORD56 VASH2 ADIPOR2 RNU6-230P SLC12A8 ZNF148 GGT8P MUC16 SPRR1A DOCK2
FAM196B B3GALT5 LINS EYS SAMD12 TNC FAM19A1 ABHD17AP5 PCNT GTF2IP2 LINC00923 IGSF3 ZNF688 MS4A1 OR6N1
OR8L1P OR8U1 PAX7 ANKH LINC00871 TCAIM TNR TNR-IT1 BCKDHB KRTAP19-7 LDB2 LINC00924 MTPN NOVA1-AS1
SHANK2 ABHD4 AGBL3 C7orf49 ARL2BPP8 COMT DGCR6L GPX1P2 GRIA1 RN7SL177P GLIS3 NF1P8 PARK2 BMPR1A
HIGD1AP2 AGBL4 AGBL4-IT1 B3GAT2 BEND5 NRG3 ZNF732 ACSS1 AKR1C1 AKR1C2 CCDC178 PSG6 PSG7 SMYD2 DIO2
DIO2-AS1 ANKRD26P3 BMS1P12 FAM230A MRPL3P1 SNX19P2 CELF2 C7orf69 CLEC16A HUS1 PKD1L1 SLC13A3 ZNF793 DLG2
AK5 NF1P1 BCL2L1 FRMD3 CCL8 MKLN1 TTC6 FGD6 TANGO2 DNAJA1P5 LINC00418 OLFM3 ZRANB2-AS2 LRRC4C NEU3
TGFBR3 CASC4P1 MRPS31P2 ANO4 ZDHHC15 LINC00159 KRTAP20-3 ZNF420 LINC00664 TRAV24 TRDV1 ZNF429 ZNF493
GRIN2A SNORD115-10 SNORD115-11 SNORD115-12 SNORD115-19 SNORD115-20 SNORD115-21 SNORD115-23 SNORD115-24
SNORD115-25 SNORD115-34 SNORD115-35 SNORD115-36 SNORD115-38 SNORD115-39 SNORD115-40 SNORD115-41 TRAV8-4
TRAV8-5 RNF152 TYW1B ANK2 CEP89 CSMD1 RTN1 GOPC ROS1 C14orf64 PIGUP1 TRPM3 KCND3 AKR1CL1 PARVB PNPLA3
SAMM50 SCN8A ANGPT4 DNAJC9 FAM149B1 FAM230C GRAMD1C NAP1L4P3 NES SORCS3 VPS26AP1 NXN POTEH-AS1
KIR2DL1 KIR2DL3 KIR2DL4 KIR2DP1 KIR2DS4 KIR3DL1 KIR3DL2 KIR3DL3 KIR3DP1 CSNK2A1 FAM83B LRP1B RNU6-352P
VN2R17P ZSCAN5A ADAM20 ADAM20P1 KCNQ4 RUNX1T1 SYNE1 ARHGAP8 FAM155A FAM155A-IT1 KIAA1199 MIR1267 PRR5
PRR5-ARHGAP8 SNORA1 DPYD DPYD-IT1 ZNF840 CNTNAP4 FAM65B MEG8 RN7SKP233 SNORD113-1 SNORD113-2 PDE4DIP

*RN7SL143P RPS27P27 SH2D1A STAG2 SYPL2 EFCAB6 GPC6 LINC00639 TF TFP1 LINC00276 TTLL11 TTLL11-IT1 PRIMA1
SRRM4 CTXN2 CYP2C8 SLC12A1 ZNF678 FSIP1 DPP10 LRFN2 OR5D3P RNU6-250P PIK3C2B SLC35F3 LINC01090 LSM12
ATP5O CRYZL1 ITSN1 PLGRKT GRIA4 MAP2K3 MYH1 CECR7 ERCC4 CEACAMP6 DNAH14 PSG10P PSG8 HLCS HLCS-IT1
PPP4R4 RNASE11 RNASE12 LONP2 BCAN VSIG10 AFF3 LINC00470 LINC01020 MIR4677 PRR12 PSMC6 SAMD4A SDCCAG8
MYBL2 LAMA2 RNU6-400P ZNF486 CEP41 CFTRP1 LINC00478 MIR99A MIRLET7C TAOK3 YRDCP3 C17orf51 CAPN14 CC2D2A
FAM189A1 KLHDC7A NDNL2 IGLVIVOR22-1 ANKHD1 ANKHD1-EIF4EBP3 ARHGAP32 EIF4EBP3 RGL1 SELO TUBGCP6
CACNA1B CHEK2 KIAA1407 MMP16 QTRTD1 NEK5 MIPOL1 NALCN-AS1 SLCO2B1 DDAH1 HBD OR4K11P RNF215 SEC14L2
GADD45A PRKD1 ZNF790 MX2 ZNF720 ADAMTSL1 MIR3152 PIP5K1P2 APBA1 LINC00861 SOD1P3 EVA1C EXOSC3P1
RPL23AP12 RPS27P16 RPS3AP1 SLIT3 LINC00674 PRDM9 RN7SKP99 SLC35F4 ZMYM5 ACIN1 C14orf119 ITPRIPL2 SYT17 PCP4
CHODL-AS1 IGHV3-43 SLC22A2 STXBP4 ACTBP8 LINC00906 RYK ZNF227 ZNF331 CELSR1 MDN1 PRB1 PRB2 FAM189A2
PLXNA4 SLC22A10 FRMD6-AS2 KCNG3 PPP1R26P3 TUBGCP5 CIZ1 KRTAP10-1 KRTAP10-5 KRTAP12-2 KRTAP12-4 NT5DC1
TSPEAR FLRT2 HOMER2P2 IGHV3-25 IGHVIII-25-1 LINC00226 NLN TOX3 GPC3 NHSL2 SLC7A8 FTLP15 RPS15AP34 SARS
TTC4P1 ADARB1 CHAF1A SLC35A5 FBLN5 GALNTL6 GRAMD4 NRG1 NRG1-IT1 NRG1-IT2 RIMBP2 CPXM2 ETS2 KBTBD11
PSMB2 SHISA6 AOX1 PDE1C RAD51D RAD51L3-RFFL RFFL SLCO3A1 NPAS3 PDE1A PRR9 TAS2R1 TRAV6 CYYR1 FRMD6
HYDIN KIAA1644 RNA5SP385 RNU6-1291P ZNF337 ATP5J GABPA PCBP3 PTPRN2 TRPC4 ALDH1A2 ANKRD26P4 B3GALTL
CDK6 CHCHD6 RNU6-10P ANKRD20A18P CES1P1 EWSR1 LIPI RNA5SP488 WDR64 CCDC59 WDR52 CERS3 CLVS2 SPIDR
GZMH LINC00408 LINC00839 LINC01146 ROBO2 ZNF385D ZNF72P ANKRD32 BPESC1 GGT1 LINC00320 MCTP1 MRPS22 NPR3
SNRPD3 TMEM56 TMEM56-RWDD3 UNC79 ABHD17C CCDC34 LARP1B RNU6-185P ST8SIA1 THEMIS GALC ICT1 LCORL
RAD23BLP TMEM178B ARFIP1 IPPK MKRN3 PRMT8 STK38L TIGD4 ASTN2 KCNMB3P1 MARK2P9 PLCE1 REV1 RPL10P3
SH3RF2 SNORA70C TMEM132D FBXL7 NOS2P3 USP32P3 DYRK4 GAB1 GALNT8 KCNA6 KYNU NDUFA9 OR4C7P GRIA3
HS1BP3 HS1BP3-IT1 KSR2 SPOCK1 GLP2R OR10T1P C14orf164 RN7SKP139 SEMA6D DRP2 MPST RALYL ARHGEF26-AS1
ATP8A2 BACH1 BACH1-IT2 BACH1-IT3 GRIK1-AS2 LINC00189 RNU6-78P AKR1B15 CFTR LINC00382 BBS9 LUC7L MYO9A
RBFOX2 DNAJC2 DTX4 EGFEM1P MIR551B PMPCB SYNM ZNF257 DNAH6 LINC01035 MED13L MIR620 OR2BH1P PA2G4P3
PAPD7 RBM22P1 SCFD2 SH3TC2 SLC15A5 MAP4K3 PCTP SNORA67 BAZ1B COL8A1 MC2R TIAM1 BTNL9 ERICH1-AS1
LRRC37A7P OR4A43P PM20D1 PPM1J RHOC VWC2 COL22A1 FAM135B KL LIPE-AS1 RNU4-60P WBP11P1 CYCSP17 LDLRAD4
MIR5190 TRIM9 CTBP2P7 IGHV1-3 IGHVIII-2-1 PSTPIP2 ABCA9 KIF16B SLC44A5 STEAP2-AS1 PHC1 SEZ6L ADC DNAJC15
MSL2 RGS7BP TANC2 ATF2 DNAH9 KIAA1217 MIR603 RASGRF1 DPYSL2 GRID1 INSL6 PCDH11X RN7SKP238 ZNF98 DEPDC5
FAR2 MYO3A REEP3 SLC6A2 ATP9B GRIN2B HPSE2 IL1RAPL2 KCTD9P2 LINC00200 RPL18AP14 RTCA TET3 BPTF NME7
SLC17A3 SMTN C16orf3 GAS8 NKD1 PCDH7 C21orf2 CDH11 FAM174B MRPS6 OTUD4 SAMD5 SGCD SLC5A3 TACC2 FNDC1
GNB5 KRTAP29-1 ADAM28 BTN2A1 FGD4 LINC01053 PDZD2 PLCB4 RNU6-494P TPT1P5 DMD FAM27C FAM27E4 LINC00376
RNU1-33P GLT1D1 GLTSCR1 PTPRE LRRC49 PIK3C3 UTRN ZMYM4 NCF4 BACE2 BACE2-IT1 LINC00473 LINC00559 PDE10A
PLCXD3 RFPL4AP7 RNU4ATAC8P SDIM1 SERPINA9 SNTA1 SPEF2 VPS13B ZNF536 GIMAP4 MTHFD2L ZNF90P3 CPA6
MIR4742 SAMSN1 SLC44A1 WDR26 CTNNA3 ERC1 KRT43P LRRTM3 PAMR1 SGMS2 TAF4 IGKC IGKJ4 IGKJ5 IGLV2-18 IGLV3-
19 MYOM3 NELL1 TMEM173 FBXO31 IGHV4-4 MPPED1 DICER1-AS1 ERCC6 FREM2 GAPDHP69 KLHL32 KLRF2 KRT18P2
MARK2P8 MTUS2 NIPA1 RAB7A RNA5SP20 RNU6-56P ROCK2 SIRPG ZZZ3 CNTN4 FIP1L1 IGKV2OR2-1 IGKV2OR2-2 LNX1-AS1
LNX1-AS2 LYPD5 PDGFRA RGL2 SLC24A4 TAPBP ZNF283 ANKS1B ARID5B CST13P DOT1L GRHL2 IL1RAPL1 PHACTR1 REST
RNA5SP366 RNU1-11P S100B ZNF679 ACTG1P1 CNKSR2 FRMD4A GBP4 PRKACB SNAP25-AS1 SPOCK3 TEX41 ZNF366 C2CD3
COL15A1 CSGALNACT1 EPHA3 GPATCH2 IL1RN INVS APC BNIP3P1 CACNG2 CEP170 DCLK1 DCLK2 FAM8A2P IZUMO3
OR8K2P PXDN RPS3AP46 SRP19 XBP1P1 FAM111B HDAC9 LRRC71 SAMD3 TAB3 ZCCHC11 AJAP1 ATG4C BRD9 CDK8 LEMD3
OR8K3 ZDHHC11 ZDHHC11B AGT C15orf32 C6orf183 ERC2 OR5D15P PHACTR3 RCAN1 REXO1L1P REXO1L3P REXO1L8P
TMEM132C UBE2R2 ATXN3L C10orf11 CST2 FHL2 FSIP2 MAGEB3 SNORA38 ATAT1 AVEN CHRM5 FBXO32 MRPS18B VTI1A
WDR11 ATP6V0D1 CTDSPL PCCA PLEKHA5 PNLIPRP3 TENM2 TPH2 TRHDE KRT126P LMO7 UCHL3 VAT1L ZFYVE1 CDH2
COL16A1 COX10 KLHL25 MIR1276 OR4K13 PRDM15 PRMT3 ASXL3 BRE CDH9 DZANK1 GCNT1P1 LINC00851 MRPL33 NLGN1
PGAP3 PSPC1 RASSF3 AGGF1P4 BANP EFCAB11 ENTPD4 FAM228A FAM228B FOXN3 FRS2 LGALS14 LOXL2 SCEL DLGAP1
EEF1A1P1 KANK1 LINC00393 RNU6-1327P BMS1P15 CDC5L CEP112 DIAPH2 DIAPH2-AS1 FAM160A1 FRG1 FRMPD4 PARVG
RN7SL659P SOAT1 TRIM51 ABCD1P5 AQP4-AS1 GNPTAB GREB1L HMCN1 IGHV3-75 IGHV3-76 SNORD23 BCL2A1 BPIFB1
CORO2B TFDP2 AGMO MIR600HG PRKCE PTPRM RIPK4 RN7SL163P RPL39P40 STRBP ZNF804A CYBB FTH1P27 GDI2P2
IL1R2 LANCL3 MCTP2 NEGR1 NEGR1-IT1 OR52N5 OR52U1P OTC PRRG1 PSPH RNU6-49P SGOL2 SLC39A11 SYTL5 TM4SF2
TRIM22 TRIM5 TSPAN7 XK BRF1 DIP2C GNB4 NOVA1 RPSAP55 RYR2 SEMA3D SNORA32 UBE2CP2 CES1P2 COL25A1 DACH1
EFCAB6-AS1 KCNIP4 KIAA0825 MTND6P3 NTN1 RNU1-139P RNU6-690P RPL4P2 SPAG16 TP53I11 ZNF677 C14orf37 CYP46A1
IPO8P1 KCNH1 PDXDC1 SLC22A23 TNFSF15 USP25 ZBTB8A ZBTB8B DMXL2 ENOX1 RNU1-142P SPATA7 TLN2 ZNF10 ZNF268
CD8B CPSF1P1 DCUN1D4 GPR55 OR6K4P SLC5A4 TNRC18P3 TXK CP CT49 DENND2A LRRTM4 MYCT1 OR52B4 RNF185
RNU6-1063P USP40 USP6 WSCD2 ZNF232 ANKFN1 C4orf29 CXorf22 FLNC LPIN3 MGMT TRAPPC9 ACSM2B ATRN CYCSP51
DBH DNAJA1P4 FBXO27 hsa-mir-490 IGHVIII-26-1 MIR4290 PATE4 RPS4XP22 SARDH SMPD4P2 TNS3 TSPAN8 TUBA3C ZNF845
DUSP27 GMEB2 KCNMA1 KRTAP8-3P OR5BL1P OR9I3P OR9Q1 PCDH17 PRKCH SETD3 STT3B TLK1 ZNRF3 ZNRF3-IT1
C1orf168 C5orf64 CASP8 COL9A1 CXorf30 DPP8 FAM107B FMNL3 GTF2E1 KIF19 OR7E25P PLD5 RAPGEF5 RIN3 SDK1
TRAPPC10 TRIO ATE1 CYB5R2 CYP39A1 FTO HDAC2 HTRA1 LAMA4 LINC00907 POLR1A SLIT2 TFAP2D ZBTB34 ATP10A
C3orf67 ESRRG GAREM HNRNPA1P7 MARCH10 OTX2-AS1 PWP2 RNA5SP453 SYCP2 ADORA3 CAMK1D CDH8 DYNLL2
MIR4480 NTM PDE9A RAP1A RN7SKP197 RN7SKP76 SNORD45 TMEM233 APOOP1 CACNB2 KLHL14 MARCH3 SNX30 SPTLC3
SRSF4 TIMD4 TNS1 AHRR CENPU DDX24 DISC1 DISC1-IT1 FBXL13 LCMT1 PDCD6 RN7SKP140 RNU5A-5P TM4SF1 TSNAX
TSNAX-DISC1 ZSCAN5C BTF3P14 CEP152 DEFB122 DGCR2 DNAJC6 EVA1A LGR5 LST3 MECP2 OR2L13 OTUD7A RNASET2
RPS6KA2 RPS6KA2-IT1 SLCO1B1 SLCO1B3 SLCO1B7 CEP97 JAM2 KSR1P1 LINC00698 LPHN3 MAGOH2 OR5AK4P OR5AO1P
RBPMSLP REG4 TMEM220 C19orf18 CHRNA7 CNBD1 FAM27B FAM27E3 IGF1R IL17RA KRT25 PALMD SMAD1 ZNF606 ACTA2-
AS1 BARD1 C5orf38 DSC3 E2F3 NUTF2 RNU6-141P STAMBPL1 SYT9 UVRAG C3orf33 RNU6-46P SH3BGR TTC40 ERICH2
MYRFL OR11G2 RAB3IP TMEM241 TMPRSS6 TTC3 ZNF567 ZNF850 CADM2 CCDC91 CTNNBL1 FAM110B ITPR2 NPSR1 NPSR1-
AS1 OR7E83P SOD3 VCAN VCAN-AS1 ADAMTS6 FAM9B GABRG1 HADHA KCNK17 LINC00113 PGM2 PTPRG PXK SLC14A2
ABCA13 CPQ DCDC2 EYA1 OR4K1 OR5J7P OR8K5 SACS SHROOM3 SLC18B1 TRPC5 VPS37B WT1 ACSM1 ACSM3 C9orf41
CA5AP1 GBE1 KCTD8 LINC00032 MAGEC3 MBD3L1 RNASE9 RNU6-723P SPATA6 SRRM1 STK33 SYT1 BASP1 BRINP2
CACNA2D3 CLDN14 KRT2 PHF3 PRKCB RNU6-1003P SIPA1L3 SLC2A9 VCL ATF7IP2 IGLVIV-64 IGLVIV-65 KIAA2022 KRT8P25
LTBR NOX5 POLR2F PPIAP14 RN7SL99P SCNN1A SPESP1 TEAD1 TPP2 UBL3 ABT1 ATP10B ATXN10 CSMD3 DSCAM-AS1*

FGF14 FGF14-IT1 FSHR GSG1L RFX4 RNU6-1241P WDPCP CCDC171 CD72 GML GRM1 IGLV3-25 LINC00617 MACC1 MACC1-AS1 MIAT PPARGC1A PPL RNA5SP222 ST3GAL3 ZNF675 ACO1 ADCK1 ADH5P3 ADORA1 AEBP2 AP3B1 COPG2 FMN2 HIVEP2 HNRNPA2B1 LINC01162 MAEL MAMLD1 MIR4798 MORF4L1 NCKAP5 NFE2L3 NRIP1 PRKAR2A RGS16 RPS26P30 SKAP1 SLFN12L SORCS2 TSGA13 ZFP30 ZNF607 ZNF781 ASTN1 DPP6 HEXB HIP1 INPP5A LINC00347 LINC00922 MBOAT7 PTGFR SCNN1B STK24 WBP1L ZNF527 ASNSP1 GART KCNC1 KIAA1671 LINC00707 LINC00879 OPCML PLAC1 CILP2 GBP6 JAKMIP1 LINC00457 LINC01122 MRPS11 OR4C5 PWRN2 SMOC1 AKR1B1P1 CNIH3 DCDC1 FAM27A FAM27E2 FOXD4L2 NR2F2-AS1 PDZRN3 RNA5SP50 RNU6-278P RNU7-119P SEL1L2 SUCLA2 ZNF782 BDH2P1 FRMPD2 GALNT14 MAN2B1 SPAG17 USP46 WDR83 WDR83OS WWOX ADRBK2 ANKRD29 ATRX CBX3P4 CCDC30 CLIC5 CPHL1P LINC00353 LINC01058 OR10N1P OR4A41P OR9L1P RAD51B ACTR5 FAM221A FRMD5 PARP16 WFDC11 ABCC9 AKAP7 C15orf27 CNTN6 FSBP IPO13 RAD54B RNLS SDR42E1 SNX18P9 WDR82 AMD1 COX6A1P3 DPRXP5 GGT2 RNU6-156P USP16 XRCC4 ACSM5 AMPD3 ARHGEF10 CD93 CTAGE5 CUZD1 DEPDC1 FAM24B KIF3B MIA2 NTNG1 OR2M5 PAN3 RNU6-725P RNU6ATAC33P RTCB SLC25A1P2 STARP1 TENM4 TMEM51 TOP3A UNC13C ADAM32 ASL ATF6 CATSPER2 CKMT1B CRCP DHRS7C DNAH2 GFRA2 GRIP1 HSPB8 LINC00472 LINC00507 LINC00508 LRRK2 MAPK10 MIR4452 MIR495 MIR543 MYH4 OSBPL9P4 PTK2 RN7SKP96 RNU6-554P RORB SH3PXD2B SLC1A3 SLCO1C1 STRC VRK1 B4GALT4 B4GALT4-AS1 DNAJC3-AS1 GRAMD3 IGFBP7 KCNH5 MEF2C MSNP1 NEK7 NXPH1 OR4K17 PIR PTPLAD2 SYCP1 ZC3H13 ANKRD34C ANKRD6 BOLL C8A KIF26B KXD1 LRTM1 NUSAP1 RORA ABCD1P3 HNRNPA1P61 IER2 ILDR1 IQCJ IQCJ-SCHIP1 LUZP2 MARCH11 MIR605 PRICKLE2 PRKG1 SCHIP1 SLC4A4 TMEM74 ZNF826P ANKS1A CHD7 CMA1 CRLS1 KCNB2 KIAA1210 LINC00645 MRPS31P5 MYO5BP3 OVGP1 PDZD7 PIWIL3 PTPDC1 RNA5SP465 RNF165 SFXN3 SLC39A8 SLC7A7 TPTE2P2 VPS13A WDR16 ZNF26 ZNF433 ZNF878 DPY30 FCF1P10 FOXD4L4 KCNK13 MEMO1 NSF TPRG1L WRAP73 WSB2 ZHX2 ARHGEF7 CDCA2 GLIS1 KCTD9 PDXP SH3BP1 SOX6 TECRL CDC42BPA DMBT1P1 MTMR2 RNA5SP280 SEMA4D TEK ATP1A4 FAM194A GPM6B GSK3B LINGO2 NAPEPLD NDST3 PREX2 RAB3C RNU6-1293P RPL19P12 SLC8A1 TCAM1P YTHDF2 ZNF432 ZNF841 BFSP1 CDC14A CYP4B1 IKZF2 LINC00595 MAGI3 MTND1P31 MTND2P25 PLCH2 SERPINA4 SERPINA5 SNRK WFDC9 ZNF430 AK4 C4orf22 DGKI G2E3 GPR158 IL18RAP KRT223P KRTAP10-10 LRBA OR4C9P ANKRD13A FLVCR1 MEGF11 NANOGNBP2 OGT RNF144A TGFB2 WDFY4 WDR4 CFL1P6 CLSTN2 EMB FMN1 GLB1L3 HNRNPA1P71 MFSD9 NCK2 PRAMEF12 SNTG2 VAV3 CDH10 DYNC1I1 EPHB1 IGHV4-55 IGHV7-56 LCE2B LCE2C MROH7 MROH7-TTC4 OR4C14P PLXDC2 SCMH1 SPATA13 TTC4 USP36 DACH2 FCF1P9 FUT4 GEMIN8P3 HORMAD2 MAST2 MUM1L1 NUAK2 OSGIN2 PIWIL4 PLCD3 PTP4A1P1 RIPPLY3 RNU6-436P S100A11 SEMA5A UPP2 UPP2-IT1 ACSBG1 AKAP6 BDNF CACNA2D4 KRT74 LLGL2 LSAMP MIR4713 PABPC1 PITPNC1 PRKAR1B RBMS3 RNU6-58P SAA2 SAA2-SAA4 SAA4 SAMD13 SDAD1P4 SGCG SNTB1 TATDN2P3 UOX ZDHHC17 CHST15 CPAMD8 KRTAP13-5P KRTAP15-1 LPPR1 MEDAG MOB3B MYO1D POLR3C SART3 TEX26-AS1 TMOD2 BTF3P10 CORIN DNAAF2 DNAH3 DNAJB4 EML1 GIPC2 GUSBP6 ITFG1 KIAA0040 MGAT2 MTND1P2 MTND2P4 PLEKHM2 SNX24 TOR1AIP1 ULBP1 ABI3BP ADAMTS17 EDIL3 GAGE2B HPS3 KRTAP20-2 SLC19A3 TOX2 WASF1 WDR72 ADARB2 CSMD2 DHX57 FOXK2 HEPHL1 KDM4C LRRC9 PCMTD1P3 POLE RPS17P15 SLC24A2 SNAP25 STK31 TRAP1 ZDHHC14 A2ML1-AS1 A2ML1-AS2 ATR CHST9 EVC2 EXT2 INIP LAMA3 MYO16 PNPT1P1 RAG1 RAG2 SIK2 SUMF1 ABCB5 DNM3 DNM3-IT1 FGGY FIGN MIPEP MREG MTCO3P2 NR2F2 OR10R2 PECR RNU6-157P RPS6KA5 TBXAS1 TUBB1 ZNF141 CD163 CD163L1 CST2P1 ERMP1 HSH2D KHDC1 MYH8 NR5A2 PAQR8 PML RNF180 STOML1 SYBU TRUB2 ZNF627 ARHGAP12 C1QTNF6 CKAP4 CORO2A ENTPD3-AS1 IGLV2-23 IGLVVI-22-1 KCNH7 MRPS27 NIFK-AS1 RN7SL153P SCN11A SLX1B SLX1B-SULT1A4 SNX29P2 STK39 SULT1A4 TMEM106B ARHGAP44 BAI3 CNTNAP2 DIO3OS DTWD2 HK3 LINC00669 RBCK1 RBM19 RN7SL456P RNU6-1184P RPL21P41 ZNF510 ABCA8 BCL2 C5orf17 CASC15 EDARADD FBN1 MCFD2 MED15 MIR548Q TTC7A ALDOAP2 BIRC6 GRIK3 HSPG2 IGHV3-16 IL20RA LCA5L LY86 LY86-AS1 MIR4789 NAALADL2 NLRP2 NLRP7 PLXNA2 SNAP23 TEX36 TMCC3 TRAF6 TRDC TRDV3 VPS53 ZNF610 ADAT3 BZW1 CHMP4C CTPS2 CYCSP6 GRM8 KIAA1328 KREMEN1 LINC00442 LINC00866 MXI1 NRG3-AS1 PLAUR PRLH RNU6-810P RXFP2 SCAMP4 ACVR1C ARHGAP24 ATP5A1 HECW2 KCNJ3 KRT222 MOV10L1 MTA3 SMARCE1 TMOD1 ZBED5 CDH12 CRYBB2P1 HMGB3P20 IL12RB2 KLHL33 LINC00486 RN7SL683P SIPA1L1 SKAP2 SLC30A5 TTC22 ASUN BTF3L4P1 CXADR DISP1 FAM90A13P FAM90A15P FAM90A20P FAM90A3P FAM90A4P FAM90A5P GULP1 HSF5 IGF2R KLF12 KPNB1 LINC00351 LRP2 NIPA2P2 NKAIN3 RN7SKP100 SIGLEC30P TMCO4 TMEM100 GPCPD1 LRRC4 MIR4768 NCOR1 NFIA NHS ROR1 SDHA SLC35F1 SND1 SNORA40 TMEM211 VANGL2 CACHD1 CDH6 COL27A1 DEPTOR GDAP1 HS6ST3 LINC00587 LINC01047 NR4A2 SLC30A10 TMEM132B UBE2E2 ZNF208 CD86 CDCA7L CYB561A3 EHBP1 EPHA7 GTF3C6 HIGD1AP13 IFNWP4 KIAA0195 LINC01070 LINC01094 MICAL3 MIR648 NF2 OBSCN PALLD PSD3 STK32B VWA8 ZBTB7C ATP9A DKK2 DPT GAGE2C IGHV7-34-1 ITGB1 KCNIP1 KRTAP4-11 KRTAP4-12 NF1P2 NPM1P13 OFD1P13Y RAC1P5 RPGR SLC9A9 ST13P15 SULT1C2P1 ADHFE1 C12orf55 C8orf46 CGNL1 CIRH1A DIS3L2 EFCAB2 FYN GNRHR KCNE2 LINC00693 LRRC69 MYL12A NPM1P31 PARN RAP1GDS1 RLBP1 RNASEH1 SLC26A7 SNTB2 TSPAN3 UBA6-AS1 VPS4A ADAMTS18 CENPBD1P1 CHN1 COX10-AS1 GAS7 LIN7A LRRC7 NR4A1 RBAK RBAKDN RBAK-RBAKDN RNF216P1 RPL23AP82 SMARCA4 SULT4A1 C1orf94 C2orf88 CD44 EIF4G3 HECW1 KIF6 MAST4 MAST4-AS1 NAP1L6 PMP22 RCVRN RHBDD1 RNF213 RNU7-35P RPS15AP6 THBS2 TMEM212 C14orf182 C14orf183 CASP6 CCDC18 FOXO1 HSD17B12 IGHV3-29 IGHV4-28 IGHVII-28-1 IL16 LINC00446 MIR670 MIR670HG PDE4D PYY RBBP8 RNF144B RNF219-AS1 RPS3AP41 SRGNP1 STARD5 ZBTB8OS CD1C CPNE4 ENPP2 MAP4K5 MIR5704 RN7SL766P SOGA1 SRRM1P2 SWT1 ABCB10 ACTN2 ALG6 ARHGEF3 DLEC1 DLGAP1-AS4 GABRB1 GRK5 IL2RA KCND2 KLF13 LMX1A MIR4681 MME PACRG PDE4B PDS5B RNF216 RNF217 SUGCT TNMD TRMT61B UBE2CP4 WDR27 CSNK1G3 CUL5 EXOC4 FBXW8 FOCAD KCNS3 LINC01013 MAT1A MIR491 MMP27 NAV2 RFC3 RPTOR SETD7 SH2D7 SNRPD1 TEX11 WRB ABCC1 ACSL4 BRI3BP CMTR2 DHRS4-AS1 DHRS4L2 EFHC2 EML5 GRIK2 HHAT IGSF11 IGSF11-AS1 ITGA9 KRT39 LAMB4 PPP2R5A SETD5-AS1 SNORA16B SRGAP3 STXBP5-AS1 ZNF516 CELF4 EFHB FAM208B GLCCI1 LNP1 MRPL42P4 NIPAL2 RNA5SP470 RNF150 RPA3-AS1 SERPINB7 SLC5A1 SQSTM1 TPO UNC5C WDFY2 APBB2 CADPS CDHR4 DNM1P51 DOPEY2 EIF3E FEZ2 KALRN LIN28B LINC01121 MYLK3 NAMPT PLEKHH2 PLK1S1 RALBP1 RASSF8-AS1 RGS7 RNU2-27P RPS15AP1 RPS17P14 SLC1A1 SPIRE2 UBE2Q2P11 UGCG ZFPM2 ZNF451 CCDC12 DEFB116 LINC00517 MAN1C1 PHF20 PHLDB2 PLCXD2 RARB RERG RNF128 RNU4-40P RPL31P3 STRIP1 AGAP1 CCDC122 DOCK3 ETV6 FAM193A GNG4 HNRNPA1P58 NLRP12 RN7SL204P RPL12L3 RPL26P9 SLC22A25 SYNPR TBC1D4 VN1R4 ZMAT4 AUTS2 CCNE1 CLYBL DPYD-AS1 GRAP2 IPO7P2 LINC00378 MFSD12 MTOR NDFIP2 OR10J6P OR4S2 PGPEP1 RMI2 UBE2L6 ARHGAP29 ASXL1 ENAH FAM49A GUCY2F KIRREL LINC00840 MOV10 NF1P3 OFD1P12Y PPP6R2P1 RPL39P33 SAP18 SNORD27 THYN1 TMPRSS4 TTC26 VPS26B BBS2 CHRM1 CR1 CRB1 CTDSPL2 DDX21 FAT3 GABRA5 KRT18P31 MEGF9 N4BP2 NIPA2 NIPBL OGFOD1 PKP2 RANBP17 RN7SL373P SATB2 SYT10 UBXN2B ZBTB41 ZNF767 BACH1-AS1 BMPER CCDC64 CCDC88B ERMN FAAH2 FAM184B HMGB1 KRTAP9-3 KRTAP9-8 NPM1P41 OR52X1P PLCL2 PTPRD RASGEF1B RBM22P2 RWDD2B SCAF8 TBX15 TIAM2 ZNF521 ZNF621 AMPH ARHGAP6 CCDC13 HBE1 HBG2 HIGD1A HNRNPA1P53 MAP3K7CL MATN3 MCM3 MIR548AX MTFMT OR51AB1P OR51B2 OR51B8P OR51I1 OR52H2P OR52T1P

PGK1 PLCL1 RN7SKP126 RN7SL83P ZFP64 ZNF483 ARL15 ARL6IP5 ATP1A1 CACNA1C CACNA1C-IT1 CACNA1C-IT2 CACNA1C-IT3 CDKAL1 CEP85L COL11A1 EPM2A FAM227B FAM90A21P FAM90A22P FAM90A23P FAM90A6P FAM90A7P LINC00971 MTMR8 PLN RALGPS1 RN7SL801P ZNF233 ZNF235 ZNRF1 ADAMTS16 AXDND1 CEP128 CHCHD3 GBA3 KIAA1715 MAPK9 MICAL2 MNS1 NOL11 OR8J3 OR8K4P STX12 TEX9 ZMAT3 ZNF569 BANF2 CHD9 CTNNBIP1 EPB41L4B EVI5 EXOC6B GAS2 KLHL1 MANEAL PHGDH PSMC1P13 RNU4-59P SOX8 ZMYND11 ZNF239 ARHGEF15 CAP2 CHTF8 CREBRF CUL2 DIDO1 DIRAS2 DSG4 GRPEL2P1 HAS3 IFT88 KIAA0753 LIN9 MIXL1 MLST8 MZF1 NET1 NLGN4X NSD1 TSPAN9 ATP6V1D CDH18 DPRXP4 EIF2S1 GTF2A1L HELLS HYAL4 KMT2A KY MYRIP RNF135 RNU4-56P RNU6-1066P STON1 STON1-GTF2A1L VDR ZNF667 ADCY8 CYP4Z1 FAM19A4 FHL5 FOXO3 GDAP2 GNG5P5 IGF2BP3 LRRTM1 MITF PAAF1 PXN RAB31 RPH3A SLC47A1 TRAPPC12 ZNF582-AS1 ZNF583 CDK14 CLDN12 CPB1 DPH6 ITLN1 LINC00448 MIR5095 MNAT1 PARD3B PIGK PJA1 PTN RBMS2 RNA5SP495 RPL21P6 SCP2 SLC27A6 SLC38A6 TBPL2 TTLL12 ZNF585A ZNF585B APTX CCDC88C CLVS1 CREB5 DYNLRB2 EDNRB KCNJ12 MSI2 MSR1 OASL RBKS RN7SL449P RNA5SP353 ROBO1 RXFP1 SEC24B SH2D4B SYNE2 TDRD3 TRAT1 BNIP3L FAP FLT1 LRRFIP1 NASP NOX4 PI4KA PRCP PRUNE2 RAB30 RBM44 SDAD1P1 SGIP1 SPRED1 ACSL5 ATAD2 ATIC CBLN4 CDH17 CENPV DNAJC25 DNAJC25-GNG10 INO80D NPLOC4 PIGL PKIB SPANXN4 TCF4 TSC22D3 TUSC3 UHMK1 ZNF573 BCO2 C10orf113 C6orf3 CATSPERB COL2A1 KCNC4 LINC00885 LRRIQ3 MALL MAP3K7 MAP7 NBAS NEBL NR3C1 NSUN3 NUMA1 RPS12P21 SDHD SIDT1 SIDT1-AS1 SPTA1 TC2N TMEM261 TRAF3IP2 ZNF277 A1CF C15orf60 DIRC1 EYA4 GABRA2 GCNT2 GTDC1 ICA1L KRT8P15 OR4C15 PLEKHA6 PTPRS SCARNA15 TMC1 TMEM138 TUFT1 XKR4 ASIP ATP6V1E1 C10orf120 CCDC170 DEFA1B DEFA3 DEFT1P2 LPP MDM1 MIR4634 PKHD1 RCHY1 RN7SKP85 SHOC2 SNORD11 STK3 SUPT3H TGM2 TMEM179 UXS1 BBOX1 BDKRB1 BDKRB2 C9orf135 CHP1 CNR2 EXD1 KDR MATN2 NSUN6 NUMB PPHLN1 RHBDL3 RN7SKP101 RNU6-249P RPL30 STAM VAPB ZNF207 5S_rRNA ADGB ARHGAP15 C18orf63 CASK CD109 CD36 CHL1 DLEU7 EDA ETFA GNAT3 KRTAP27-1 LINC00894 MAP2K4 MSRB3 NHSL1 PCOLCE2 PHKB RNF182 SNX29 ABCA12 APP CALCRL COL18A1 DGKB GREB1 KCNK2 LINC00158 METTL8 NRP1 PDZRN4 PQLC1 RN7SL674P TSNARE1 UACA ARHGEF18 C1orf87 CLIP1 CSF1 CUBNP1 ELMO1 LGR4 LINC01019 METTL15 MIR1200 NDUFAF2 OR4K4P PDSS2 RAB22A RNU1-131P RPS24P12 SEC14L6 SERPING1 A2M ADTRP CD58 CDK12 IMMP2L KAT5 LCE6A LINC00271 LINC01143 MYO3B NAP1L4P1 NSG2 OR11K2P PRELID2 RANBP10 RNASEH2C RNU7-66P RRP15 SHISA5 ST7 ST7-OT4 THRB TMEM11 TTC12 ZNF626 ZNF737 BCRP7 COMMD1 CPEB2 FAM46A FUBP1 NPEPPS SCN9A SEMA3C SLC38A7 SNTG1 TRAF3IP2-AS1 UBE2K ZNF209P ADAM17 C1orf106 C5orf66 CAMK4 CYLC2 CYP3A54P FRY H2AFY LINC00536 MAP3K5 PCAT2 PKP4 RN7SL662P SMC1B SMG6 TUBAL3 ABCD3 CDC42BPG DIAPH3 HPCAL1 IGHVII-22-1 IGHVIII-22-2 ISPD KRTAP13-6P LINC00458 OR4N3P OS9 RN7SL714P RNU6-768P SLC5A8 SRIP1 TFPI TRABD2B ZER1 CCDC3 ELP3 EPHA1-AS1 ETS1 GC GPR78 GRK6 JAK2 KLHL13 MIR4300 MKKS MTND4P14 MTND5P14 NR2C2 PAPPA PAPPA-AS1 PEX5L PIK3R5 RALGPS2 RNA5SP300 TAS2R41 TPTE2P3 ANKRD30BP3 ARHGAP11B CUBNP3 DNAH5 EFEMP1 GALK2 hsa-mir-6080 MIR759 MT1HL1 OIT3 PFKFB2 PLEKHM1P PRTG RPL9P28 SHMT1 TGFB1I1 TM9SF4 U8 YOD1 CDK17 CNTN3 COMMD6 CPNE5 CXorf21 ELAVL4 GPR176 IGDCC4 PPIAP6 PSIP1 PTBP3 RNU3P3 RNU4-24P RPS23P5 SORCS1 SPATA16 TCF7L2 TEP1 TLK2 TNFRSF10B ZNF443 ZNF490 ZNF564 ZNF709 ZNF799 ACSM2A AFF2 CCT6B CD38 CYTIP EBPL ENPP7P5 H2AFZP1 IGHV3-48 IGHVIII-47-1 ITGA11 LINC00937 MYH13 NINL PARD3 RALGAPA2 SCPEP1 SKA1 SSPN STAG1 SUSD1 THOC2 TRAF3 VDAC2P1 ZNF830 ABAT ATRNL1 DOCK9 DSCAML1 ESR1 FDXR IL18R1 IL1RL1 LINC00520 MGC4294 MT2P1 OR6C68 PACRGL PXT1 RIMS3 RN7SL743P RPL7L1P12 SGK2 SLC2A12 STARD4 TMEM186 APCDD1L-AS1 ATP2B1 DSCR3 DSCR9 FAM19A2 FLG-AS1 FOXP2 HRNR KIFAP3 MIR3666 OPHN1 POTEKP RN7SL678P RNASE3 RNU6-1225P RNU6-617P RNVU1-18 RPS3P6 S100Z ADAMTS9-AS2 BCLAF1 C9orf171 CNOT1 CPB2 CPB2-AS1 DGKZP1 FBXO47 HSPA4L IMPG1 KCNMB2 LAPTM4B LARS2 LARS2-AS1 LINC00284 LRRC20 RGS3 RNU7-177P SAMD9 SLC35F2 SNORA80 URB1 FAM102B LINC00343 LINC00395 NFAM1 PEBP4 PYGL RAPGEF1 RMRPP4 SGMS1 TRPS1 ZNF568 BAZ2B BZRAP1-AS1 CASP5 CCDC169 CCDC169-SOHLH2 CCDC88A CNOT10 COPS8P3 DENND5A HNRNPA1P68 IPP KHDRBS3 LINC00544 MIR183 MIR96 NARS2 NBPF10 NOTCH2NL RIN2 RNF43 RNU6-311P SLC30A7 SOHLH2 TNKS TUB ADORA2A-AS1 ALCAM AMY1C C15orf54 C17orf75 C5orf51 C6 CHRDL1 CNN3 CNTNAP5 GATAD2B KSR1 LGALS9 MYO5B NCF2 NPL OOSP1P2 OR5G1P RNF126 SETBP1 SMG7 STK36 TBC1D3B UPB1 ZNF248 ZNF33BP1 ZNF608 ABTB2 AK7 BMS1P13 CCDC144CP DDC8 DNAH8 HDAC4 KCTD9P1 LHFPL1 LINC01043 RN7SL17P RNU6-909P SMIM2-AS1 SNORD74 SOX5 SPECC1 TIMP2 U6 AP4B1-AS1 BCL2L15 BRD4 CHCHD2P9 CWF19L2 DCK DNMT1 GHR GLRA2 KIAA0355 MAPRE2 MOB1B MS4A14 MS4A6E MS4A7 NTRK2 RNA5SP219 RNU6-552P SETD2 SRP54 TAC4 ASB17 C8orf44 C8orf44-SGK3 CACNA1I CHN2 CPNE8 EFTUD1P1 FAM171A1 FAM83G KCNK1 LATS2 LATS2-AS1 MGST1 MPZL1 NANOGP4 PAWR PCNXL4 SGK3 SLC5A10 SSXP1 CAMSAP2 CD53 DDX51 DPP9 GNG2 ITGB7 MBL1P NELL2 NOC4L POTEF RNU6-1049P SULT1B1 ADORA2A BCRP1 CDC40 CNTN4-AS2 DLEU1 EXOC6 HNRNPA1P40 LRRK1 PGM2L1 PKNOX2 POM121L9P PPFIBP1 PRAMEF26 RASGRP1 RNF220 RNU6-21P RNU6-321P SEPT7P9 SMCHD1 SPECC1L SPECC1L-ADORA2A STAU2 TTC27 UBE2W USP18 ZNF299P ARR3 ATG10 BPIFB4 C14orf39 C9orf40 CYP2AC1P FAM212B JPH3 KCTD7 OR4K6P OR5E1P PDIA6 RAB11FIP4 RABGEF1 RNU7-176P RRP7A SALL4P7 SERHL SPIN3 SPRED2 TMEM55A ZCWPW2 AMY1A CCDC144A GLI3 HNRNPKP3 KAZN LINC01101 LPPR5 MAP2K5 NCK1 OR5L2 RBPJP7 RNU6-405P RPS10P7 SEPP1 SMOC2 TDRP USP32P1 ADAM23 ARNT ATP5BP1 CASC16 CHAF1B CLDN1 COG7 DRG1 GRXCR1 KIAA1009 LYN MORC2 MORC3 MSRA OR11L1 PPA2 RN7SL568P RNU6-1266P SFI1 SPRR2B SPRR2C SPRR2E STXBP5 VWA3B DHRS3 DOCK4 LAMP5 LINC01049 NANP PRELID1P2 PXDNL SAFB2 SCGN SYNPR-AS1 TGFBR1 TMEM120B TSEN15 TTC32 USP53 ZNF337-AS1 ZNF558 ATP6V0D2 C1QTNF7 CCDC176 CYSLTR1 FCHSD2 GMDS KIAA0586 KRTAP5-8 MGLL OR7K1P PPP4R1L PRDX4 SNHG17 SOX2-OT SPG11 SVOPL TMEM41B ADAM12 ATXN1 C7orf66 DYM EPHA5 GACAT1 HTR2C LCP2 MGAM MIR1911 NFX1 OR9A4 PPFIBP2 SLC13A4 SPICE1 TAS2R38 TOP1 TRAV8-7 ACBD6 ASCC3 CHMP1B2P COBL CUX1 GLRA1 KRTAP20-1 KRTAP6-1 LINC00317 NCOA5 NFASC PSMC4 RNA5SP490 SCARNA21 SLC25A21 SLC6A17 SNX19P3 TENM3 THUMPD1 ULK4 ABLIM1 AMY1B B4GALT3 CASP12 CHCHD2P4 CHST11 CPE FAM225B HMG20A INPP4B ITGB3BP KRR1P1 MAGEA11 PPOX PTTG1IP ZBBX ZNF729 ANKUB1 C10orf90 CD96 CHRNA3 CHRNA5 DICER1 EP300 GALNT2 GLDC GZMAP1 IQSEC3 LINC00687 MBD5 METTL9 MIR1281 MIR3173 NXPH2 PCDH9-AS2 PCDHA1 PCDHA10 PCDHA11 PCDHA12 PCDHA13 PCDHA2 PCDHA3 PCDHA4 PCDHA5 PCDHA6 PCDHA7 PCDHA8 PCDHA9 PCDHAC1 PCDHAC2 PFN2 RAB2A RNU6-1005P RNU6-196P SEMA4B SLC16A7 SPARC ZNF384 ADAT2 ANKRD18A C10orf25 CEP164P1 CSRP2BP CYP4F33P ELAVL2 ERCC8 LINC00884 NUP62CL PET117 PEX3 PHF8 RNA5SP221 RSU1P2 SCARNA17 SPINT4 TBX3 TTC39B USP17L24 USP17L25 USP17L26 USP17L27 USP17L28 USP17L29 USP17L30 USP17L5 USP17L6P USP17L9P WIF1 ZNF22 ZNF474 ARMCX2 C18orf64 CACNA2D1 CASC18 CCDC73 CNTNAP3 CYP7B1 DIAPH1 DUSP23 FAM108A10P FAM209A FLT3 GCNT7 GUSBP1 HNRNPUP1 HS3ST2 IGKV1-37 IGKV2-36 IGLV4-69 IGLVI-68 MAMDC2 MIR1254-2 NOL12 PABPC1L PLAC4 PRAMENP REEP1 RNU2-5P RTFDC1 SIGLEC26P SPRR4 STX8 TCL1B TCL6 TNFRSF19 TOMM34 TRIOBP ZNF618 ANKRD31 EIF3A FPR3 GAB2 HDHD1 IQCK KIAA1244 LINC01107 PCSK5 SCFD1 SIAH3 SV2B TP73 UPRT APOL6 CAB39 COL4A6 FAM13C GRID2 IFNGR2 MIR320B2 MIR514A1 NPC1 NVL PCED1B

PIBF1 RASGRF2 RNU6-898P RRM1 TANGO6 TMEM50B TRPM7 ZNF728 ARHGEF33 BCL7C CA3 ELMO1-AS1 IGHVII-67-1 IGHVIII-67-2 IGHVIII-67-3 LINC00940 MAPK1IP1L MIR4519 MORN2 NUS1P2 OR4C10P PHYKPL PLOD2 PSMA1 SLC20A1P1 SUZ12P TERF1 TUBAP VPS39 WFDC10B ACSS3 BRD7 FHIT FLNB FLNB-AS1 FSD2 KRTAP4-3 LRRC16A ONECUT3 OR5H8P OR5K4 PAGE1 PPIAP27 PPP1R12B STK32C TTI1 UBFD1P1 ZNF345 ZNF829 C1orf167 C20orf196 C5orf47 CABIN1 CAMK2G CHRM3 CXCL13 DDX10 DYNC1I2 EFNA5 EMCN EMCN-IT2 EMCN-IT3 FAM20A FAM73A HSPE1P25 MERTK MOXD1 NF1 PKHD1L1 PPFIA2 RBMX2P1 RBMX2P3 SPATS2 SPRR2G TMEM52B UBE3C WARS2 BRD1 DAPP1 FAM129A FAM9A GPR161 GUCY2C HERC2 HLA-DQB2 LRRC16B MAGI2 MAGI2-IT1 MANSC4 OAS1 PPP6R2 RNU6-530P RNU6-917P RNU7-144P RPL39P36 SIRPB1 SIRPD TFF1 TMED8 TPT1P2 ZNF280D ZNF525 ZNF765 AKAP2 ALDH4A1 C16orf95 CCDC92 DNAH10 DNAH10OS DNAJC5 GOT2 GPR125 HNRNPA3P14 IFFO2 IGKV1-5 KCNQ5 KCNQ5-IT1 LPHN2 MGA MIR1290 MIR4695 MIR941-1 NUF2 PALM2 PALM2-AKAP2 PHKA2 PHKA2-AS1 PLD1 PTPRU RGS5 RMRPP5 SLC4A1AP SORL1 STRN3 TAS1R2 TDRD12 ZNF615 ASB13 BICC1 DLG5 ELK3 GPR126 GUSBP11 IFT80 IGSF21 LOH12CR1 MIR649 MND1 NCALD P2RX6P RPA2P1 TEAD4 TRIM59 TTC8 UBE2G2 AIFM3 ANTXR2 BID BTBD11 BTBD3 CARM1P1 CD2BP2 CNNM2 DMRT1 DSG2 ELOVL2-AS1 HPYR1 HTR4 HTR4-IT1 IFI27L2 IGF2BP1 LHFP LZTR1 MIR3198-1 PABPC1P12 PCAT1 PQLC3 RHPN2 TMEM260 WDR25 ZNF718 ATP8B4 CIT CRISP3 EIF4E EML4 FAM214A FKBP5 FRYL KANK3 LINC00575 LINC01036 MICU1 POR RELN RN7SKP86 SLC9C1 SNX20 SRD5A3-AS1 SUV420H1 TNFRSF11B TSHZ1 USP3 C1orf112 CCNYL2 CSRNP3 GRM5 HAPLN1 KIAA1324 KIRREL3 MIR517A MIR519D MIR521-2 MIR524 MROH2B NECAB1 PTPN9 PWP1 RNU6-16P RNU6-803P SELE SNUPN ST6GALNAC5 CNTN1 COL12A1 DTD1 DYNC1H1 FCRL3 FNDC3A GPATCH2L LINC00298 LINC00299 MGAT5 MRPL39 PAK1 PLS1 PLSCR4 PTK2B PTPN20B PWRN3 STAC TMPRSS4-AS1 ACYP2 ATP5F1P6 ATP8B5P C15orf26 C18orf8 DDC EFTUD1 IGHV3-60 KCNIP3 MXRA5 NDRG2 OR4C2P PSMG2 RAF1 RN7SL552P RNASE13 RNU1-77P RNU6-469P RP2 SP2 TMEM131 TMEM161A TMEM38B TNFAIP8 WDFY3 ALMS1 CCDC26 CCDC57 COLEC12 FAM108A8P FAM210A IGHV3OR16-12 LINC00604 LINC00702 MAP3K13 NLK NPHP4 RALA RN7SL435P RNU1-150P RNU6-953P YWHAQP9 ZNF215 ADCYAP1R1 AMZ2 C9orf3 DPY19L2P2 EGLN3 IL1RAP LINC01029 LRRC37A5P MROH1 MRPS21 NAV3 PTGR1 SLFN5 SPCS2P4 SPTSSA ARF4 COL6A5 EDA2R FBXL17 FBXL5 FNDC3B HERC2P5 JAG1 LINC00466 LRIT3 MAML3 MAN1A1 MEOX2 NHLRC2 NMNAT1P1 OXCT1 PGM1 PPP3CA QSER1 RN7SKP253 RN7SL130P RN7SL141P RRH TBC1D12 TMEM163 TMEM178A TRIM60P13 TSHR AKNAD1 ARMC10P1 BANK1 C3orf22 CHST13 DCBLD2 EXOC5 GPR133 GPSM2 HGSNAT JRK KIAA1598 KLF3 KLF7 MIR3118-5 MTND5P5 MYT1L NDEL1 NDUFA13 NEB RGS12 RNU6-26P RXRG SEPT9 SH3GL2 SLC7A1 SNX25 SUCLG1 TANC1 TMEM67 UBR3 YJEFN3 CAPZB CENPK CLTA DGCR10 DGCR5 DGCR9 EFCAB1 EZH2 FMO9P FSTL5 GABRR2 GRM7 HMGB3P24 KIAA0100 KLHDC8A LINC00374 LIPA MAS1 MSANTD3 MSANTD3-TMEFF1 NPFFR1 OR8A2P PER1 PRKCQ-AS1 RNA5SP487 RNF144A-AS1 SATB1 SDF2 SNORA75 STPG2 TAF1B TBC1D5 TMEFF1 TSPAN33 WWTR1 WWTR1-IT1 ZNF177 ZNF330 ZNF559 ZNF559-ZNF177 CBLB CCSER2 CDC20B DOCK10 ESR2 GSTA2 MAGEB2 MIR4439 OR8S1 PLSCR1 RERGL RN7SL194P RNF17 RPL5P35 SEC63 SLC25A48 AAGAB BMS1P16 C16orf80 C7orf76 CDRT1 CTNNA1 DGUOK DGUOK-AS1 DOK6 GPC4 IGHVII-44-2 IGHVIV-44-1 PDXK RN7SKP162 RPS24P16 SHFM1 STARD9 SUN3 TNIP1 TRAV33 TRIM16 WDR59 ZFRP1 ZNF736 AHSA1 ENPP7 FAM126B KAT7 L3MBTL3 LINC00380 MAPK4 MPRIP SNORA46 SSBP2 STIM2 TMEM135 TMEM194B WFDC10A YIPF6 ZNF540 ZNF571 ZNF571-AS1 AGGF1 ANXA4 BORA CHD6 CLDN11 DENND1A FHOD3 FREM1 ITGAE KIF13B LHFPL3 LINC00656 LUZP4 MARK2P5 MZT1 N4BP2L1 N4BP2L2 NLGN1-AS1 P2RX6 PCA3 PIGFP2 RNU6-280P RPS20 SLC7A14 SNORA16 SNORD54 TCF12 THAP7 THAP7-AS1 TRAV22 TUBA3FP ZNF734P AKIRIN2 ARHGAP5 ASAP1 BRMS1L CHST8 CTTNBP2 DCLRE1C GABRB2 GBP2 GBP7 GNE H2BFM LINC00440 SH3KBP1 SLC22A3 SLC6A10P SSUH2 ST7L VIPR2 ZNF562 AK8 CALB1 CCDC60 CLDN8 DMBT1 ENOX2 EPHB2 FAM118A GAGE12I GAGE2D HBG1 OR11H1 OSBPL5 PHACTR2 RAD51AP1 SDR39U1 TTLL9 WSCD1 ATP8A1 DEFB127 DNAJA1P1 FOXRED2 FSD1L GPR64 MAPK8IP2 NABP1 RALGAPA1P RUSC1 RUSC1-AS1 TMPRSS11E VEPH1 VSTM2B ACOX3 AGPAT4 CACNA1D CACNB4 CD300C COL21A1 DDX3X DPY19L3 EDDM3A EGFL6 LRPPRC NMNAT1P4 NSG1 RBL2 RNU1-104P SERPINB12 ARAP2 CADM3 CCZ1 CNOT7 DARC DGKH FAM46D FEM1AP4 FGFR1OP2P1 HS6ST2 OMG OR7D1P PDE11A RPL31P40 RPL7P55 SKOR1 SNORD109B SNORD115-45 SNORD115-47 SNORD115-48 VPS37A EHF FYB GJB7 KIAA1462 NSUN7 PARP8 PDE7A POLQ PTPN20A RN7SKP168 RN7SKP218 RN7SKP6 SCUBE1 SHROOM4 TPCN2 ZNF622 ARHGAP39 C1orf177 DCAF7 DNMT3B EGLN3-AS1 F7 KIAA1432 LINC00970 LINC01088 MCF2L NAA11 PNLIPRP1 RN7SKP199 RNU6-288P SHOX2 SMEK2 SOBP SPNS2 STIM1 ADK AHI1 C11orf65 CDH26 GRIK4 KCNT2 KIF9-AS1 LINC01010 MMP26 MYBPC2 OR51A8P OR51F1 OR51H2P RPA1 SMAD3 TPH1 VPS41 ABHD2 BAIAP2L1 C7orf60 CD300A CNN2P4 FXR1 IPPKP1 KIAA1257 KIDINS220 MCOLN2 NDST4 NOS1 POU5F1P6 PTGFRN RPN1 TCEB1P32 TDRD10 TMC7 TMTC1 TTC29 ZNF160 ZNF347 ZNF415 ADAMTS2 AKR1C3 BSN-AS2 BTBD9 COX5BP6 CUBN DHX15 EFCAB4B ENPP7P1 FAM85B FBXW12 FOXP1 HDX KIAA1211 LMBR1 LRCH1 MLIP MLIP-AS1 MTUS2-AS2 MYOM1 PLXNB1 PPM1D RDH10 RN7SL16P RN7SL321P RNY4P7 SLFN12 TFF3 TMPRSS12 TMTC4 TRANK1 URGCP URGCP-MRPS24 WDR33 AATF ADCY9 ANHX BCAR3 CCDC149 CDK1 CEP135 CYCSP27 FAHD2A HDAC7 HIVEP3 HTR7 ITPK1 LDLR LTB MAGT1 MCM3AP MCM3AP-AS1 MIR548AL MRGPRG NBN PRRC2C RNA5SP186 RNVU1-17 SEPT14 SLC39A10 SV2C TAGLN3 TNF VASH1 ARHGAP22 ARHGAP22-IT1 ARMC8 BDH2 C9orf131 CDRT4 FLJ00273 FLVCR2 GPN3 HRH1 IFT43 LINC00534 LINC00972 NME9 OVCH1 OVCH1-AS1 OVOL2 PANX1 SLC9B2 SNORD17 SNX5 TTLL5 TVP23C TVP23C-CDRT4 VDAC1P1 ZFPM1 ZNF454 AKT3 ATXN8OS CHKB CHKB-CPT1B CPT1B CSPP1 CYP4Z2P GAK ILF3 NGLY1 PIGB POMC PSG1 RAPGEF4-AS1 RNU6-132P SOS1 TMC2 UCHL1-AS1 WDR93 ADAM9 ADNP2 BFSP2 BLOC1S6 BMP15 DENND1B DHRS11 GMFB LINC00521 LINC01141 MRM1 NUDT4 PCDHGA1 PCDHGA10 PCDHGA11 PCDHGA12 PCDHGA2 PCDHGA3 PCDHGA4 PCDHGA5 PCDHGA6 PCDHGA7 PCDHGA8 PCDHGA9 PCDHGB1 PCDHGB2 PCDHGB3 PCDHGB4 PCDHGB6 PCDHGB7 PCDHGB8P PHEX POU6F2 RBM11 RNU6-164P RNU6-540P SCAF4 SQRDL TRIM23 AFAP1 ANAPC1P1 C2orf48 CEA CEACAM5 CEACAM6 CLOCK CRYL1 DPY19L1 DPY19L2 DYRK1A GLRX3 IGLC1 IGLC2 IGLJ1 IGLJ2 IGLL5 LGR6 MAGI1 MAGI1-IT1 MAP6 MAPKAPK5P1 MIR4327 MIR4499 MS4A5 OR10AK1P OR11A1 OR5V1 RGPD1 RNA5SP125 RXFP4 SIM2 VN1R87P WRN ARID2 C17orf80 FAM104A FAM63B GYG1P1 H3F3C HS3ST5 LINC00161 LINC01057 MARK3 MIR4529 MMP20 OR1AA1P OR1E3 PAXBP1 PGBD4P7 PLEKHG1 RCAN2 RSRC1 SERTM1 SHC2 TMC5 TNRC6B ZSWIM3 BEND7 BRINP1 C21orf90 CLSTN1 COG3 CPEB1 CYP4X1 F11-AS1 FAM220A GPR39 HHLA1 KATNAL1 KCNK10 LINC00632 LRR1 LRRC37A12P MED4 OC90 OR4F14P PLCH1 PRG3 RHOH RHOQP1 RIMKLB RN7SKP60 RPL36AL SLC7A13 TSPEAR-AS1 UBQLN4 WDR95P YBX1P7 ARF1 BCL11A CALD1 CLEC4A DKFZP761J1410 DPY19L2P1 ELAVL1 ENPP7P2 EPN2 EPN2-AS1 FAM222B HMGA2 KIAA1731 MTHFS MTURN MYO6 NEK4 POU5F1P3 SDC2 SIPA1L2 SNORA25 SNORA8 SNORD6 ST20 ST20-MTHFS TAF1D UBE2E3 WDYHV1 YAF2 ZNF41 ZNF705A ZNF880 ABL1 AGO3 ANKRD30A ANP32C ATP8A2P1 BCRP6 BRSK2 CRADD DISC1FP1 DOCK1 FAM213A KIF18A LINC00993 MARCH1 MIR4490 MLTK MUC5AC PAPOLA PCDH19 PCNXL2 PCSK6 PIP5K1A PRDX2P3 PRR14L PTPRJ RPL21P11 RUFY1 SLC27A2 TMEM161BP1 TSPAN13 VN1R53P ZNF2 ZSWIM7 ANO1 CACNG6 CCDC146 CERS6 DTX2P1 DTX2P1-UPK3BP1-PMS2P11 EFCAB5 FAM101A GCLC H3F3AP4 HIAT1 ISM1-AS1 LENG8 LINC00523 MCM9 OXSR1 PDIA5

PMEPA1 PMS2P11 PMS2P9 QKI RAB23 RN7SL587P SEC22A SNORA71 SPTLC2 TMEM189 TMEM189-UBE2V1 TRDN UBE2V1 UPK3B WIPF1 ZNF664 ZNF879

Table S2. rDNA-contacting genes involved in different biological processes (g:Profiler). Related to Figure 1.

GO item	# of genes	Names of genes
homophilic cell adhesion via plasma membrane adhesion molecules	79	*CDH4,PCDH9,DSCAM,PTPRT,PCDH15,SDK2,CDH13,CELSR1,ROBO2,PCDH11X,PCDH7,CDH11,CNTN4,CDH2,CDH9,HMCN1,PTPRM,PCDH17,SDK1,CDH8,DSC3,CNTN6,CLSTN2,EMB,CDH10,CDH12,CDH6,PALLD,ITGB1,CDHR4,FAT3,DSG4,CDH18,ROBO1,CDH17,DSCAML1,TENM3,PCDHA1,PCDHA10,PCDHA11,PCDHA12,PCDHA13,PCDHA2,PCDHA3,PCDHA4,PCDHA5,PCDHA6,PCDHA7,PCDHA8,PCDHA9,PCDHAC1,PCDHAC2,IGSF21,DSG2,KIRREL3,CADM3,CDH26,PCDHGA1,PCDHGA10,PCDHGA11,PCDHGA12,PCDHGA2,PCDHGA3,PCDHGA4,PCDHGA5,PCDHGA6,PCDHGA7,PCDHGA8,PCDHGA9,PCDHGB1,PCDHGB2,PCDHGB3,PCDHGB4,PCDHGB6,PCDHGB7,CEACAM5,CEACAM6,CLSTN1,PCDH19*
nervous system development	500	*SRGAP2B,MACROD2,CDH4,PLCB1,DAB1,DSCAM,GABRB3,ITGAM,ARID1B,TMEM108,CHODL,C21ORF91,HAPLN3,PCSK2,RUNX1,ASIC2,NCAM2,SYNDIG1,ALK,NTRK3,PCDH15,UNC5D,ANK3,SDK2,HCN1,RBFOX1,CA10,DCC,FSTL4,DLC1,PTPN11,SLC1A2,APBA2,STRADB,TRAK2,CECR2,RIMS2,ARNT2,GRIK1,NPHP3,CTNNA2,MDM2,INPP5F,DPF3,SUN2,CBFA2T2,SHC3,GRIN3A,NRXN1,CTNND2,RIMS1,NRXN3,WLS,DOK5,MDGA2,POTEE,ZNF148,TNC,PAX7,TNR,MTPN,SHANK2,PARK2,BMPR1A,NRG3,OLFM3,LRRC4C,GRIN2A,ANK2,SCN8A,NES,SYPL2,SRRM4,BCAN,SDCCAG8,LAMA2,CC2D2A,PRKD1,APBA1,SLIT3,SYT17,PCP4,RYK,CELSR1,PLXNA4,FLRT2,ADARB1,NRG1,HYDIN,ATP5J,TRPC4,ALDH1A2,CDK6,ROBO2,GAB1,SPOCK1,SEMA6D,DRP2,MPST,ATP8A2,RBFOX2,SH3TC2,TIAM1,VWC2,SEZ6L,RASGRF1,DPYSL2,GRIN2B,IL1RAPL2,BPTF,GAS8,NKD1,CDH11,SLC5A3,TACC2,DMD,ZNF536,LRRTM3,NELL1,FBXO31,CNTN4,GRHL2,IL1RAPL1,REST,S100B,PRKACB,C2CD3,CSGALNACT1,EPHA3,DCLK1,DCLK2,HDAC9,AGT,RCAN1,ATAT1,ATP6V0D1,TENM2,CDH2,CDH9,NLGN1,FRS2,KANK1,BCL2A1,PTPRM,ZNF804A,NEGR1,XK,GNB4,SEMA3D,COL25A1,NTN1,CYP46A1,TRAPPC9,ATRN,PCDH17,PRKCH,RAPGEF5,SDK1,HDAC2,SLIT2,TFAP2D,CAMK1D,NTM,RAP1A,DISC1,MECP2,CHRNA7,SMAD1,VCAN,PTPRG,DCDC2,EYA1,SHROOM3,TRPC5,SYT1,BASP1,BRINP2,VCL,ABT1,ATXN10,CSMD3,FGF14,FSHR,RFX4,WDPCP,PPARGC1A,ADORA1,ASTN1,HEXB,MBOAT7,STK24,GART,KCNC1,OPCML,ATRX,CNTN6,ARHGEF10,NTNG1,TENM4,GFRA2,GRIP1,LRRK2,PTK2,RORB,SLC1A3,STRC,MEF2C,LRTM1,RORA,IER2,PRKG1,ANKS1A,CHD7,CMA1,PDZD7,RNF165,VPS13A,ZHX2,ARHGEF7,SOX6,MTMR2,SEMA4D,GPM6B,GSK3B,LINGO2,PREX2,SLC8A1,ZNF430,AK4,TGFB2,CLSTN2,EMB,NCK2,SNTG2,EPHB1,SEMA5A,ACSBG1,BDNF,LSAMP,MYO1D,TMOD2,EML1,WASF1,KDM4C,SNAP25,MYO16,DNM3,NR2F2,RPS6KA5,SYBU,TMEM106B,ARHGAP44,CNTNAP2,BCL2,HSPG2,PLXNA2,TRAF6,PRLH,HECW2,SMARCE1,SIPA1L1,LRP2,NFIA,ROR1,SDHA,VANGL2,NR4A2,EPHA7,NF2,PALLD,ITGB1,FYN,CHN1,GAS7,LRRC7,SMARCA4,HECW1,PMP22,THBS2,ENPP2,GABRB1,LMX1A,FBXW8,NAV2,ACSL4,UNC5C,APBB2,FEZ2,KALRN,RARB,ETV6,AUTS2,MTOR,ENAH,BBS2,CHRM1,CRB1,GABRA5,NIPBL,SATB2,HMGB1,PTPRD,ZNF521,EPM2A,MAPK9,KLHL1,PHGDH,SOX8,ARHGEF15,IFT88,NLGN4X,FOXO3,LRRTM1,MNAT1,PTN,EDNRB,ROBO1,SEC24B,SYNE2,ATIC,TCF4,UHMK1,COL2A1,MALL,SPTA1,PTPRS,TMC1,STK3,MATN2,NUMB,CHL1,APP,NRP1,CSF1,IMMP2L,SEMA3C,FRY,ISPD,ELP3,ETS1,JAK2,NR2C2,DNAH5,PRTG,CNTN3,CPNE5,ELAVL4,AFF2,CD38,PARD3,THOC2,ABAT,DSCAML1,ATP2B1,FOXP2,OPHN1,RAPGEF1,CASP5,CCDC88A,DENND5A,NOTCH2NL,ALCAM,CHRDL1,STK36,HDAC4,SOX5,TIMP2,NTRK2,SETD2,CAMSAP2,ADORA2A,GLI3,NCK1,ADAM23,CLDN1,GRXCR1,LYN,TGFBR1,SPG11,TMEM41B,ATXN1,EPHA5,COBL,CUX1,NFASC,SLC6A17,TENM3,ULK4,HMG20A,CHRNA3,DICER1,EP300,MBD5,PCDHA1,PCDHA10,PCDHA11,PCDHA2,PCDHA3,PCDHA4,PCDHA5,PCDHA6,PCDHA7,PCDHA8,PCDHAC1,PCDHAC2,PHF8,TBX3,TRIOBP,GAB2,TP73,GRID2,RRM1,CAMK2G,CHRM3,EFNA5,NF1,GPR161,MAGI2,SORL1,DLG5,IGSF21,TTC8,BTBD3,IGF2BP1,CIT,EIF4E,FRYL,RELN,HAPLN1,KIRREL3,PTPN9,CNTN1,PAK1,PTK2B,NDRG2,MAP3K13,NPHP4,RALA,IL1RAP,NAV3,ARF4,JAG1,OXCT1,PPP3CA,TSHR,KLF7,MYT1L,NDEL1,SH3G*

		L2,EZH2,MAS1,DOCK10,CTNNA1,DGUOK,DOK6,GPC4,KIF13B,TCF12,ASAP1,CHST8,CTTNBP2,GABRB2,SH3KBP1,AK8,EPHB2,MAPK8IP2,OMG,SKOR1,SHROOM4,DNMT3B,SHOX2,AHI1,KIDINS220,NOS1,PLXNB1,CDK1,ITPK1,LDLR,TAGLN3,TNF,OVOL2,AKT3,GAK,SOS1,ADNP2,BLOC1S6,GMFB,POU6F2,DYRK1A,MAP6,SIM2,WRN,BRINP1,CLSTN1,ARF1,BCL11A,SDC2,ABL1,BRSK2,PCDH19
neuron development	262	*CDH4,DAB1,DSCAM,GABRB3,ARID1B,TMEM108,CHODL,C21ORF91,RUNX1,NCAM2,ALK,NTRK3,PCDH15,UNC5D,ANK3,HCN1,DCC,FSTL4,PTPN11,STRADB,TRAK2,CECR2,RIMS2,CTNNA2,MDM2,INPP5F,CBFA2T2,SHC3,GRIN3A,NRXN1,CTNND2,RIMS1,NRXN3,DOK5,TNC,TNR,SHANK2,PARK2,OLFM3,LRRC4C,LAMA2,PRKD1,SLIT3,SYT17,RYK,PLXNA4,FLRT2,ADARB1,ROBO2,GAB1,SPOCK1,SEMA6D,ATP8A2,RBFOX2,TIAM1,RASGRF1,DPYSL2,CDH11,DMD,FBXO31,CNTN4,IL1RAPL1,S100B,EPHA3,DCLK1,DCLK2,AGT,ATAT1,TENM2,CDH2,NLGN1,FRS2,KANK1,PTPRM,ZNF804A,NEGR1,XK,SEMA3D,COL25A1,NTN1,SDK1,HDAC2,SLIT2,CAMK1D,NTM,RAP1A,MECP2,CHRNA7,PTPRG,DCDC2,TRPC5,SYT1,VCL,ATXN10,CSMD3,FSHR,WDPCP,STK24,OPCML,CNTN6,NTNG1,TENM4,GFRA2,GRIP1,LRRK2,PTK2,RORB,SLC1A3,STRC,MEF2C,PRKG1,ANKS1A,PDZD7,RNF165,MTMR2,SEMA4D,GSK3B,PREX2,TGFB2,EMB,NCK2,EPHB1,SEMA5A,BDNF,WASF1,SNAP25,MYO16,DNM3,RPS6KA5,TMEM106B,ARHGAP44,CNTNAP2,BCL2,PLXNA2,HECW2,SIPA1L1,LRP2,VANGL2,NR4A2,EPHA7,PALLD,ITGB1,FYN,CHN1,GAS7,LRRC7,HECW1,PMP22,GABRB1,LMX1A,FBXW8,ACSL4,UNC5C,APBB2,FEZ2,KALRN,AUTS2,MTOR,ENAH,CRB1,GABRA5,HMGB1,PTPRD,MAPK9,KLHL1,PHGDH,IFT88,PTN,EDNRB,ROBO1,SEC24B,UHMK1,SPTA1,PTPRS,TMC1,MATN2,NUMB,CHL1,APP,NRP1,SEMA3C,FRY,ISPD,JAK2,CPNE5,ELAVL4,CD38,PARD3,THOC2,DSCAML1,OPHN1,RAPGEF1,CCDC88A,DENND5A,ALCAM,NTRK2,CAMSAP2,ADORA2A,GLI3,NCK1,GRXCR1,LYN,SPG11,EPHA5,COBL,CUX1,NFASC,TENM3,ULK4,CHRNA3,DICER1,EP300,TRIOBP,GAB2,GRID2,EFNA5,MAGI2,DLG5,TTC8,BTBD3,FRYL,RELN,KIRREL3,PTPN9,CNTN1,PAK1,PTK2B,MAP3K13,NPHP4,ARF4,PPP3CA,TSHR,KLF7,MYT1L,NDEL1,SH3GL2,EZH2,DOCK10,CTNNA1,DGUOK,DOK6,KIF13B,ASAP1,GABRB2,SH3KBP1,EPHB2,MAPK8IP2,OMG,SKOR1,SHOX2,AHI1,KIDINS220,PLXNB1,GAK,SOS1,BLOC1S6,MAP6,ARF1,BCL11A,SDC2,ABL1,BRSK2*
regulation of membrane potential	122	*PIEZO2,GABRB3,GABRG3,TMEM108,ASIC2,CACNA1E,NTRK3,ANK3,HCN1,RIMS2,CHRFAM7A,KCNQ1,NRXN1,RIMS1,GABRA3,KCNE1,SHANK2,GRIA1,PARK2,BCL2L1,GRIN2A,ANK2,KCND3,SCN8A,GRIA4,CACNA1B,GRIA3,CFTR,GRIN2B,DMD,SNTA1,CTNNA3,IL1RN,CACNG2,NLGN1,RYR2,KCNH1,KCNMA1,CACNB2,MECP2,CHRNA7,GABRG1,PXK,TRPC5,FGF14,GRM1,ADORA1,KCNC1,CNIH3,AKAP7,LRRK2,KCNH5,MEF2C,MTMR2,ATP1A4,GSK3B,SLC8A1,DGKI,AKAP6,ABCB5,HSH2D,KCNH7,SCN11A,BCL2,GRIK3,KCNJ3,CXADR,KCNE2,SLC26A7,ACTN2,GABRB1,KCND2,GRIK2,CELF4,GABRA5,PKP2,ARL6IP5,ATP1A1,CACNA1C,PLN,NLGN4X,FAM19A4,PTN,BNIP3L,BCO2,GABRA2,CNR2,KDR,APP,KCNK2,SCN9A,ABAT,RIMS3,KCNMB2,GLRA2,NTRK2,CACNA1I,KCNK1,ADORA2A,KCTD7,PPA2,USP53,GLRA1,CHRNA3,CHRNA5,CACNA2D1,GRID2,BID,DSG2,RELN,GRM5,PTK2B,PPP3CA,GABRR2,P2RX6,GABRB2,MAPK8IP2,CACNA1D,KCNT2,ABL1,GCLC,TRDN*
synaptic signaling	172	*GABRB3,GABRG3,DTNA,ARID1B,TMEM108,ASIC2,CACNA1E,SHISA9,SYN3,SLC1A2,APBA2,RIMS2,GRIK1,GABBR2,CHRFAM7A,SHC3,NRXN1,CACNG3,RIMS1,SYT6,NRXN3,GABRA3,RAB5A,TNR,SHANK2,GRIA1,PARK2,NRG3,DLG2,GRIN2A,CEP89,SORCS3,CNTNAP4,GRIA4,LAMA2,CACNA1B,APBA1,SYT17,SLC22A2,ADARB1,RIMBP2,SHISA6,PTPRN2,MCTP1,GRIA3,TRIM9,RASGRF1,GRID1,SLC6A2,GRIN2B,CNTN4,IL1RAPL1,S100B,CACNG2,AGT,CHRM5,CDH2,NLGN1,DLGAP1,PRKCE,NOVA1,DBH,PCDH17,CDH8,RAP1A,CACNB2,MECP2,CHRNA7,SYT9,GABRG1,PXK,SYT1,FGF14,GRM1,ADORA1,CNIH3,USP46,UNC13C,LRRK2,SLC1A3,MEF2C,TPRG1L,MTMR2,GSK3B,DGKI,CLSTN2,BDNF,TMOD2,SLC24A2,SNAP25,SYBU,GRIK3,SNAP23,GRM8,SIPA1L1,LIN7A,PMP22,GABRB1,KCND2,RNF216,EXOC4,GRIK2,CELF4,SQSTM1,CADPS,SLC1A1,SYNPR,MTOR,CHRM1,GABRA5,SYT10,PLCL2,PTPRD,AMPH,PGK1,PLCL1,NLGN4X,LRRTM1,PTN,KCNC4,GABRA2,CNR2,CASK,APP,JAK2,GPR176,CD38,ABAT,RIMS3,OPHN1,GLRA2,NTRK2,ADORA2A,JPH3,SCGN,FCHSD2,SPG11,HTR2C,GLRA1,CHRNA3,CHRNA5,PFN2,SV2B,GRID2,CHRM3,NF1,PPFIA2,DNAJC5,HTR4,RELN,GRM5,PTK2B,PPP3CA,GABRR2,GRM7,P2RX6,GABRB2,CALB1,EPHB2,MAPK8IP2,NSG1,GRIK4,NOS1,CUBN,HTR7,SV2C,TNF,HRH1,BLOC1S6,CLSTN1,ARF1,ABL1*
chemical synaptic transmission	169	*GABRB3,GABRG3,DTNA,ARID1B,TMEM108,ASIC2,CACNA1E,SHISA9,SYN3,SLC1A2,APBA2,RIMS2,GRIK1,GABBR2,CHRFAM7A,SHC3,NRXN1,CACNG3,RIMS1,SYT6,NRXN3,GABRA3,RAB5A,TNR,SHANK2,GRIA1,PARK2,NRG3,DLG2,GRIN2A,CEP89,SORCS3,CNTNAP4,GRIA4,LAMA2,CACNA1B,APBA1,SYT17,SLC22A2,ADARB1,RIMBP2,SHISA6,PTPRN2,MCTP1,GRIA3,TRIM9,RASGRF1,GRID1,SLC6A2,GRIN2B,CNTN4,S100B,CACNG2,AGT,CHRM5,CDH2,NLGN1,DLGAP1,PRKCE,NOVA1,DBH,PCDH17,CDH8,RAP1A,CACNB2,MEC*

		P2,CHRNA7,SYT9,GABRG1,PXK,SYT1,FGF14,GRM1,ADORA1,CNIH3,USP46,UNC13C,LRRK2,SLC1A3,MEF2C,TPRG1L,MTMR2,GSK3B,DGKI,CLSTN2,BDNF,TMOD2,SLC24A2,SNAP25,SYBU,GRIK3,SNAP23,GRM8,SIPA1L1,LIN7A,PMP22,GABRB1,KCND2,RNF216,EXOC4,GRIK2,CELF4,SQSTM1,CADPS,SLC1A1,SYNPR,MTOR,CHRM1,GABRA5,SYT10,PLCL2,AMPH,PGK1,PLCL1,NLGN4X,LRRTM1,PTN,KCNC4,GABRA2,CNR2,CASK,APP,JAK2,GPR176,CD38,ABAT,RIMS3,OPHN1,GLRA2,NTRK2,ADORA2A,JPH3,SCGN,FCHSD2,SPG11,HTR2C,GLRA1,CHRNA3,CHRNA5,PFN2,SV2B,GRID2,CHRM3,NF1,PPFIA2,DNAJC5,HTR4,RELN,GRM5,PTK2B,PPP3CA,GABRR2,GRM7,P2RX6,GABRB2,CALB1,EPHB2,MAPK8IP2,NSG1,GRIK4,CUBN,HTR7,SV2C,TNF,HRH1,BLOC1S6,CLSTN1,ARF1,ABL1
neuron projection development	233	*CDH4,DAB1,DSCAM,ARID1B,TMEM108,CHODL,C21ORF91,NCAM2,NTRK3,PCDH15,UNC5D,ANK3,DCC,FSTL4,PTPN11,STRADB,TRAK2,CECR2,RIMS2,CTNNA2,MDM2,INPP5F,CBFA2T2,SHC3,GRIN3A,NRXN1,CTNND2,RIMS1,NRXN3,DOK5,TNC,TNR,SHANK2,PARK2,LRRC4C,LAMA2,PRKD1,SLIT3,SYT17,RYK,PLXNA4,FLRT2,ADARB1,ROBO2,GAB1,SPOCK1,SEMA6D,ATP8A2,RBFOX2,TIAM1,RASGRF1,DPYSL2,CDH11,DMD,FBXO31,CNTN4,IL1RAPL1,S100B,EPHA3,DCLK1,AGT,CDH2,NLGN1,FRS2,KANK1,PTPRM,ZNF804A,NEGR1,XK,SEMA3D,COL25A1,NTN1,SDK1,HDAC2,SLIT2,CAMK1D,RAP1A,MECP2,CHRNA7,PTPRG,DCDC2,TRPC5,SYT1,VCL,ATXN10,CSMD3,FSHR,STK24,CNTN6,NTNG1,GFRA2,GRIP1,LRRK2,PTK2,STRC,MEF2C,PRKG1,PDZD7,RNF165,SEMA4D,GSK3B,PREX2,EMB,NCK2,EPHB1,SEMA5A,BDNF,WASF1,SNAP25,MYO16,DNM3,RPS6KA5,TMEM106B,ARHGAP44,CNTNAP2,BCL2,PLXNA2,HECW2,SIPA1L1,LRP2,VANGL2,NR4A2,EPHA7,PALLD,ITGB1,FYN,CHN1,GAS7,LRRC7,HECW1,PMP22,LMX1A,FBXW8,ACSL4,UNC5C,APBB2,FEZ2,KALRN,AUTS2,MTOR,ENAH,HMGB1,PTPRD,KLHL1,PHGDH,IFT88,PTN,ROBO1,SEC24B,UHMK1,SPTA1,PTPRS,MATN2,NUMB,CHL1,APP,NRP1,SEMA3C,FRY,ISPD,JAK2,CPNE5,ELAVL4,CD38,PARD3,DSCAML1,OPHN1,RAPGEF1,CCDC88A,DENND5A,ALCAM,NTRK2,CAMSAP2,ADORA2A,GLI3,NCK1,GRXCR1,LYN,SPG11,EPHA5,COBL,CUX1,NFASC,TENM3,ULK4,CHRNA3,DICER1,EP300,TRIOBP,GAB2,GRID2,EFNA5,MAGI2,DLG5,TTC8,BTBD3,FRYL,RELN,KIRREL3,PTPN9,CNTN1,PAK1,PTK2B,MAP3K13,ARF4,PPP3CA,TSHR,KLF7,NDEL1,SH3GL2,EZH2,DOCK10,CTNNA1,DGUOK,DOK6,KIF13B,ASAP1,SH3KBP1,EPHB2,MAPK8IP2,OMG,SHOX2,KIDINS220,PLXNB1,GAK,SOS1,BLOC1S6,MAP6,ARF1,BCL11A,SDC2,ABL1,BRSK2*
component morphogenesis	260	*CDH4,DAB1,CDC42EP3,LDB3,DSCAM,FMNL2,MAPK14,TMEM108,ZRANB1,PEAK1,NTRK3,PCDH15,UNC5D,ANK3,DCC,FSTL4,CAPN3,DLC1,PTPN11,STRADB,TRAK2,RIMS2,AKAP13,FBLN1,CTNNA2,RHOJ,NUBPL,SHC3,NRXN1,CTNND2,RIMS1,NRXN3,DOK5,ZMYM2,COL23A1,TNR,SHANK2,PARK2,BCL2L1,MKLN1,FGD6,LRRC4C,ANK2,PARVB,LAMA2,SLIT3,ZMYM5,SYT17,RYK,PLXNA4,FLRT2,ADARB1,NRG1,FRMD6,ROBO2,GAB1,SEMA6D,ATP8A2,RBFOX2,TIAM1,RHOC,DPYSL2,C21ORF2,CDH11,FGD4,DMD,ZMYM4,MYOM3,FBXO31,CNTN4,PDGFRA,GRHL2,IL1RAPL1,S100B,DCLK1,CDH2,NLGN1,FRS2,KANK1,PARVG,PTPRM,XK,SEMA3D,COL25A1,NTN1,FMNL3,SLIT2,ATP10A,CHRNA7,PALMD,DCDC2,SHROOM3,TRPC5,SYT1,SIPA1L3,VCL,WDPCP,MAEL,HEXB,STK24,CNTN6,NTNG1,TENM4,GFRA2,GRIP1,LRRK2,PTK2,SLC1A3,STRC,MEF2C,PDZD7,RNF165,ARHGEF7,SOX6,MTMR2,SEMA4D,TEK,GSK3B,PREX2,YTHDF2,TGFB2,EMB,EPHB1,SEMA5A,BDNF,SART3,TMOD2,WASF1,MYO16,DNM3,RPS6KA5,TMEM106B,ARHGAP44,CNTNAP2,BCL2,PLXNA2,HECW2,TMOD1,SIPA1L1,LRP2,VANGL2,NR4A2,EPHA7,OBSCN,PALLD,ITGB1,FYN,CHN1,GAS7,CD44,HECW1,PMP22,ENPP2,ACTN2,LMX1A,TNMD,FBXW8,UNC5C,APBB2,FEZ2,KALRN,MYLK3,PHLDB2,STRIP1,AUTS2,ASXL1,ENAH,ERMN,PTPRD,MATN3,GAS2,CAP2,VDR,PTN,ROBO1,SEC24B,NOX4,NEBL,SPTA1,PTPRS,KDR,MATN2,NUMB,ARHGAP15,CHL1,APP,COL18A1,NRP1,SEMA3C,FRY,ISPD,CPNE5,ELAVL4,PARD3,DSCAML1,OPHN1,ALCAM,NTRK2,ITGB7,ADORA2A,GLI3,SPG11,EPHA5,COBL,CUX1,NFASC,CHRNA3,DICER1,EP300,SPARC,DIAPH1,TRIOBP,GAB2,SCFD1,FLNB,EFNA5,MERTK,PALM2,PALM2-AKAP2,TTC8,BTBD3,DMRT1,FRYL,RELN,KIRREL3,COL12A1,PAK1,PTK2B,RP2,MAP3K13,PPP3CA,KLF7,NDEL1,NEB,SH3GL2,CAPZB,LIPA,DOCK10,DOK6,FHOD3,KIF13B,SH3KBP1,EPHB2,MAPK8IP2,COL21A1,OMG,SHOX2,MYBPC2,KIDINS220,MYOM1,PLXNB1,ZFPM1,SOS1,MAP6,RHOH,BCL11A,SDC2,ABL1,BRSK2,DOCK1*
cell morphogenesis	238	*CDH4,DAB1,CDC42EP3,DSCAM,FMNL2,MAPK14,TMEM108,ZRANB1,PEAK1,NTRK3,PCDH15,UNC5D,ANK3,DCC,FSTL4,DLC1,PTPN11,STRADB,TRAK2,RIMS2,FBLN1,CTNNA2,RHOJ,SHC3,NRXN1,CTNND2,RIMS1,NRXN3,DOK5,ZMYM2,COL23A1,TNR,SHANK2,PARK2,MKLN1,FGD6,LRRC4C,PARVB,LAMA2,SLIT3,ZMYM5,SYT17,RYK,PLXNA4,FLRT2,ADARB1,NRG1,FRMD6,ROBO2,GAB1,SEMA6D,ATP8A2,RBFOX2,TIAM1,RHOC,DPYSL2,C21ORF2,CDH11,FGD4,DMD,ZMYM4,FBXO31,CNTN4,GRHL2,IL1RAPL1,S100B,DCLK1,C*

		DH2,NLGN1,FRS2,KANK1,PARVG,PTPRM,XK,SEMA3D,COL25A1,NTN1,FMNL3,SLIT2,ATP10A,CHRNA7,PALMD,DCDC2,SHROOM3,TRPC5,SYT1,SIPA1L3,VCL,WDPCP,MAEL,HEXB,STK24,CNTN6,NTNG1,GFRA2,GRIP1,LRRK2,PTK2,SLC1A3,STRC,MEF2C,PDZD7,RNF165,ARHGEF7,SOX6,SEMA4D,TEK,GSK3B,PREX2,YTHDF2,TGFB2,EMB,EPHB1,SEMA5A,BDNF,SART3,WASF1,MYO16,DNM3,RPS6KA5,TMEM106B,ARHGAP44,CNTNAP2,BCL2,PLXNA2,HECW2,SIPA1L1,LRP2,VANGL2,NR4A2,EPHA7,PALLD,ITGB1,FYN,CHN1,GAS7,CD44,HECW1,ENPP2,LMX1A,TNMD,FBXW8,UNC5C,APBB2,FEZ2,KALRN,STRIP1,AUTS2,ASXL1,ENAH,ERMN,PTPRD,MATN3,GAS2,CAP2,VDR,PTN,ROBO1,SEC24B,NOX4,SPTA1,PTPRS,KDR,MATN2,NUMB,ARHGAP15,CHL1,APP,COL18A1,NRP1,SEMA3C,FRY,ISPD,CPNE5,ELAVL4,PARD3,DSCAML1,OPHN1,ALCAM,NTRK2,ITGB7,ADORA2A,GLI3,SPG11,EPHA5,COBL,CUX1,NFASC,CHRNA3,DICER1,EP300,SPARC,DIAPH1,TRIOBP,GAB2,SCFD1,FLNB,EFNA5,MERTK,PALM2,PALM2-AKAP2,TTC8,BTBD3,DMRT1,FRYL,RELN,KIRREL3,COL12A1,PAK1,PTK2B,RP2,MAP3K13,PPP3CA,KLF7,NDEL1,SH3GL2,CAPZB,LIPA,DOCK10,DOK6,KIF13B,SH3KBP1,EPHB2,MAPK8IP2,COL21A1,OMG,SHOX2,KIDINS220,PLXNB1,ZFPM1,SOS1,MAP6,RHOH,BCL11A,SDC2,ABL1,BRSK2,DOCK1
plasma membrane bounded cell projection organization	330	*CDH4,DAB1,CDC42EP3,DSCAM,ARID1B,TMEM108,CHODL,C21ORF91,NCAM2,CROCC,NTRK3,PCDH15,UNC5D,ANK3,DCC,FSTL4,PTPN11,VAV2,CDH13,STRADB,TRAK2,CECR2,RIMS2,NPHP3,CTNNA2,MDM2,INPP5F,TTLL8,CBFA2T2,TBC1D30,SHC3,TRPM2,GRIN3A,NRXN1,CTNND2,RIMS1,NRXN3,DOK5,RAB5A,TNC,PCNT,TNR,SHANK2,PARK2,FGD6,LRRC4C,CEP89,PARVB,SDCCAG8,LAMA2,CEP41,CC2D2A,PRKD1,SLIT3,SYT17,RYK,PLXNA4,FLRT2,ADARB1,HYDIN,ROBO2,GAB1,SPOCK1,SEMA6D,ATP8A2,BBS9,RBFOX2,TIAM1,RASGRF1,DPYSL2,GRIN2B,GAS8,C21ORF2,CDH11,FGD4,DMD,LRRC49,FBXO31,CNTN4,IL1RAPL1,S100B,C2CD3,EPHA3,APC,DCLK1,AGT,ATAT1,WDR11,ATP6V0D1,TENM2,CDH2,NLGN1,FRS2,KANK1,PTPRM,ZNF804A,NEGR1,XK,SEMA3D,COL25A1,NTN1,SPAG16,KIF19,SDK1,HDAC2,SLIT2,CAMK1D,DYNLL2,RAP1A,DISC1,CEP152,MECP2,CEP97,CHRNA7,IGF1R,RAB3IP,PTPRG,DCDC2,TRPC5,SPATA6,SYT1,VCL,ATXN10,CSMD3,FSHR,RFX4,WDPCP,STK24,SPAG17,CNTN6,KIF3B,NTNG1,GFRA2,GRIP1,LRRK2,PTK2,STRC,MEF2C,PRKG1,PDZD7,PTPDC1,RNF165,WRAP73,ARHGEF7,SH3BP1,MTMR2,SEMA4D,GSK3B,PREX2,EMB,NCK2,VAV3,EPHB1,SEMA5A,BDNF,DNAAF2,WASF1,SNAP25,MYO16,DNM3,RPS6KA5,TMEM106B,ARHGAP44,CNTNAP2,BCL2,LCA5L,PLXNA2,HECW2,SIPA1L1,LRP2,VANGL2,NR4A2,EPHA7,PALLD,ITGB1,RPGR,FYN,CHN1,GAS7,LRRC7,CD44,HECW1,PMP22,ENPP2,ACTN2,LMX1A,FBXW8,ACSL4,UNC5C,APBB2,FEZ2,KALRN,AUTS2,MTOR,ENAH,TTC26,BBS2,HMGB1,PTPRD,CCDC13,ADAMTS16,MNS1,KLHL1,PHGDH,IFT88,ATP6V1D,PTN,DYNLRB2,ROBO1,SEC24B,SYNE2,UHMK1,SPTA1,PTPRS,TMEM138,PKHD1,MATN2,NUMB,CHL1,APP,NRP1,SEMA3C,FRY,ISPD,JAK2,DNAH5,CPNE5,ELAVL4,CD38,NINL,PARD3,DSCAML1,KIFAP3,OPHN1,RAPGEF1,CCDC88A,DENND5A,ALCAM,STK36,DNAH8,HDAC4,NTRK2,CAMSAP2,ADORA2A,GLI3,NCK1,GRXCR1,LYN,SFI1,TGFBR1,KIAA0586,SPG11,EPHA5,COBL,CUX1,NFASC,TENM3,ULK4,ABLIM1,CHRNA3,DICER1,EP300,PFN2,TRIOBP,GAB2,GRID2,PIBF1,DYNC1I2,EFNA5,MAGI2,PLD1,DLG5,IFT80,TTC8,BTBD3,FRYL,RELN,KIRREL3,PTPN9,CNTN1,DYNC1H1,PAK1,PLS1,PTK2B,ALMS1,MAP3K13,NPHP4,RALA,NAV3,ARF4,PPP3CA,TSHR,KLF7,NDEL1,SEPT9,SH3GL2,TANC1,TMEM67,CAPZB,EZH2,WWTR1,DOCK10,CTNNA1,DGUOK,DOK6,KIF13B,ASAP1,SH3KBP1,EPHB2,MAPK8IP2,OMG,SHOX2,AHI1,KIDINS220,PLXNB1,CDK1,CEP135,SEPT14,IFT43,GAK,SOS1,BLOC1S6,MAP6,ARF1,BCL11A,SDC2,ABL1,BRSK2,PIP5K1A,RAB23*
dendrite development	73	*DAB1,DSCAM,ARID1B,C21ORF91,DCC,FSTL4,TRAK2,CTNNA2,GRIN3A,CTNND2,SHANK2,RBFOX2,TIAM1,FBXO31,IL1RAPL1,DCLK1,NLGN1,SDK1,HDAC2,CAMK1D,MECP2,CHRNA7,DCDC2,TRPC5,CSMD3,GRIP1,LRRK2,MEF2C,PRKG1,SEMA4D,GSK3B,PREX2,NCK2,EPHB1,DNM3,TMEM106B,ARHGAP44,HECW2,SIPA1L1,ITGB1,FYN,HECW1,FBXW8,ACSL4,KALRN,MTOR,PTPRD,KLHL1,PTPRS,MATN2,APP,NRP1,ELAVL4,CAMSAP2,COBL,CUX1,CHRNA3,DLG5,BTBD3,RELN,ARF4,PPP3CA,KLF7,EZH2,DOCK10,ASAP1,EPHB2,MAPK8IP2,KIDINS220,MAP6,ARF1,BCL11A,SDC2*
cell projection organization	336	*CDH4,DAB1,CDC42EP3,DSCAM,ARID1B,TMEM108,CHODL,C21ORF91,NCAM2,CROCC,NTRK3,PCDH15,UNC5D,ANK3,DCC,FSTL4,PTPN11,VAV2,CDH13,STRADB,TRAK2,CECR2,RIMS2,NPHP3,CTNNA2,ITGA2,MDM2,INPP5F,TTLL8,RHOJ,CBFA2T2,TBC1D30,SHC3,TRPM2,GRIN3A,NRXN1,CTNND2,RIMS1,NRXN3,DOK5,RAB5A,TNC,PCNT,TNR,SHANK2,PARK2,FGD6,LRRC4C,CEP89,PARVB,SDCCAG8,LAMA2,CEP41,CC2D2A,PRKD1,SLIT3,SYT17,RYK,PLXNA4,FLRT2,ADARB1,HYDIN,ROBO2,GAB1,SPOCK1,SEMA6D,ATP8A2,BBS9,RBFOX2,TIAM1,DNAH9,RASGRF1,DPYSL2,GRIN2B,GAS8,C21ORF2,CDH11,FGD4,DMD,LRRC49,FBXO31,CNTN4,IL1RAPL1,S100B,C2CD3,EPHA3,APC,DCLK1,AGT,ATAT1,WDR11,ATP6V0D1,TENM2,CDH2,NLGN1,FRS2,KANK1,PARVG,PTPRM,ZNF804A,NEGR1,XK,SEMA3D,COL25A1,NTN1,SPAG16,KIF19,SDK1,*

		HDAC2,SLIT2,CAMK1D,DYNLL2,RAP1A,DISC1,CEP152,MECP2,CEP97,CHRNA7,IGF1R,RAB3IP,PTPRG,DCDC2,TRPC5,SPATA6,SYT1,VCL,ATXN10,CSMD3,FSHR,RFX4,WDPCP,STK24,SPAG17,CNTN6,KIF3B,NTNG1,GFRA2,GRIP1,LRRK2,PTK2,STRC,MEF2C,PRKG1,PDZD7,PTPDC1,RNF165,WRAP73,ARHGEF7,SH3BP1,MTMR2,SEMA4D,GSK3B,PREX2,EMB,NCK2,VAV3,EPHB1,SEMA5A,BDNF,DNAAF2,WASF1,SNAP25,MYO16,DNM3,RPS6KA5,TMEM106B,ARHGAP44,CNTNAP2,BCL2,LCA5L,PLXNA2,HECW2,SIPA1L1,LRP2,VANGL2,NR4A2,EPHA7,PALLD,ITGB1,RPGR,FYN,CHN1,GAS7,LRRC7,CD44,HECW1,PMP22,ENPP2,ACTN2,LMX1A,FBXW8,ACSL4,UNC5C,APBB2,FEZ2,KALRN,AUTS2,MTOR,ENAH,TTC26,BBS2,ERMN,HMGB1,PTPRD,CCDC13,ADAMTS16,MNS1,KLHL1,PHGDH,IFT88,ATP6V1D,PTN,DYNLRB2,ROBO1,SEC24B,SYNE2,UHMK1,SPTA1,PTPRS,TMEM138,PKHD1,MATN2,NUMB,CHL1,APP,NRP1,SEMA3C,FRY,ISPD,JAK2,DNAH5,CPNE5,ELAVL4,CD38,NINL,PARD3,DSCAML1,KIFAP3,OPHN1,RAPGEF1,CCDC88A,DENND5A,ALCAM,STK36,AK7,DNAH8,HDAC4,NTRK2,CAMSAP2,ADORA2A,GLI3,NCK1,GRXCR1,LYN,SFI1,TGFBR1,KIAA0586,SPG11,EPHA5,COBL,CUX1,NFASC,TENM3,ULK4,ABLIM1,CHRNA3,DICER1,EP300,PFN2,TRIOBP,GAB2,GRID2,PIBF1,DYNC1I2,EFNA5,MAGI2,PLD1,DLG5,IFT80,TTC8,BTBD3,FRYL,RELN,KIRREL3,PTPN9,CNTN1,DYNC1H1,PAK1,PLS1,PTK2B,ALMS1,MAP3K13,NPHP4,RALA,NAV3,ARF4,PPP3CA,TSHR,KLF7,NDEL1,SEPT9,SH3GL2,TANC1,TMEM67,CAPZB,EZH2,WWTR1,DOCK10,CTNNA1,DGUOK,DOK6,KIF13B,ASAP1,SH3KBP1,EPHB2,MAPK8IP2,OMG,SHOX2,AHI1,KIDINS220,PLXNB1,CDK1,CEP135,SEPT14,IFT43,GAK,SOS1,BLOC1S6,MAP6,ARF1,BCL11A,SDC2,ABL1,BRSK2,PIP5K1A,RAB23
regulation of postsynaptic membrane potential	49	*GABRB3,GABRG3,TMEM108,RIMS2,CHRFAM7A,NRXN1,RIMS1,GABRA3,SHANK2,GRIA1,GRIN2A,GRIA4,GRIA3,GRIN2B,NLGN1,MECP2,CHRNA7,GABRG1,FGF14,GRM1,ADORA1,LRRK2,MEF2C,MTMR2,GSK3B,SLC8A1,DGKI,GABRB1,KCND2,GRIK2,CELF4,GABRA5,NLGN4X,GABRA2,APP,ABAT,GLRA2,ADORA2A,GLRA1,CHRNA3,CHRNA5,GRID2,RELN,GRM5,PTK2B,PPP3CA,GABRR2,P2RX6,MAPK8IP2*
glutamate receptor signaling pathway	37	*PLCB1,SHISA9,GRIK1,GRIN3A,NRXN1,CACNG3,SHANK2,GRIA1,GRIN2A,TRPM3,GRIA4,SHISA6,GRIA3,TIAM1,RASGRF1,GRID1,GRIN2B,CACNG2,NLGN1,GSG1L,GRM1,PPARGC1A,CNIH3,MEF2C,GRIK3,GRM8,FYN,GRIK2,APP,GRID2,RELN,GRM5,PTK2B,GRM7,EPHB2,MAPK8IP2,GRIK4*
neuron differentiation	299	*CDH4,DAB1,DSCAM,GABRB3,ARID1B,TMEM108,CHODL,C21ORF91,RUNX1,NCAM2,ALK,NTRK3,PCDH15,UNC5D,ANK3,SDK2,HCN1,DCC,FSTL4,PTPN11,STRADB,TRAK2,CECR2,RIMS2,CTNNA2,MDM2,INPP5F,CBFA2T2,SHC3,GRIN3A,NRXN1,CTNND2,RIMS1,NRXN3,DOK5,MDGA2,TNC,PAX7,TNR,MTPN,SHANK2,PARK2,OLFM3,LRRC4C,LAMA2,PRKD1,SLIT3,SYT17,PCP4,RYK,PLXNA4,FLRT2,ADARB1,NRG1,ALDH1A2,ROBO2,GAB1,SPOCK1,SEMA6D,ATP8A2,RBFOX2,TIAM1,VWC2,RASGRF1,DPYSL2,NKD1,CDH11,DMD,ZNF536,FBXO31,CNTN4,IL1RAPL1,REST,S100B,EPHA3,DCLK1,DCLK2,HDAC9,AGT,ATAT1,TENM2,CDH2,NLGN1,FRS2,KANK1,PTPRM,ZNF804A,NEGR1,XK,SEMA3D,COL25A1,NTN1,TRAPPC9,SDK1,HDAC2,SLIT2,CAMK1D,NTM,RAP1A,MECP2,CHRNA7,PTPRG,DCDC2,EYA1,TRPC5,SYT1,BRINP2,VCL,ABT1,ATXN10,CSMD3,FSHR,WDPCP,STK24,OPCML,CNTN6,NTNG1,TENM4,GFRA2,GRIP1,LRRK2,PTK2,RORB,SLC1A3,STRC,MEF2C,RORA,IER2,PRKG1,ANKS1A,PDZD7,RNF165,ZHX2,MTMR2,SEMA4D,GSK3B,PREX2,TGFB2,EMB,NCK2,EPHB1,SEMA5A,BDNF,WASF1,KDM4C,SNAP25,MYO16,DNM3,RPS6KA5,TMEM106B,ARHGAP44,CNTNAP2,BCL2,PLXNA2,HECW2,SIPA1L1,LRP2,VANGL2,NR4A2,EPHA7,PALLD,ITGB1,FYN,CHN1,GAS7,LRRC7,HECW1,PMP22,GABRB1,LMX1A,FBXW8,ACSL4,UNC5C,APBB2,FEZ2,KALRN,RARB,AUTS2,MTOR,ENAH,CRB1,GABRA5,SATB2,HMGB1,PTPRD,ZNF521,MAPK9,KLHL1,PHGDH,SOX8,IFT88,NLGN4X,FOXO3,PTN,EDNRB,ROBO1,SEC24B,TCF4,UHMK1,SPTA1,PTPRS,TMC1,MATN2,NUMB,CHL1,APP,NRP1,SEMA3C,FRY,ISPD,JAK2,CPNE5,ELAVL4,CD38,PARD3,THOC2,DSCAML1,OPHN1,RAPGEF1,CCDC88A,DENND5A,ALCAM,TIMP2,NTRK2,CAMSAP2,ADORA2A,GLI3,NCK1,GRXCR1,LYN,TGFBR1,SPG11,EPHA5,COBL,CUX1,NFASC,TENM3,ULK4,HMG20A,CHRNA3,DICER1,EP300,TRIOBP,GAB2,TP73,GRID2,EFNA5,MAGI2,DLG5,TTC8,BTBD3,EIF4E,FRYL,RELN,KIRREL3,PTPN9,CNTN1,PAK1,PTK2B,MAP3K13,NPHP4,ARF4,JAG1,PPP3CA,TSHR,KLF7,MYT1L,NDEL1,SH3GL2,EZH2,DOCK10,CTNNA1,DGUOK,DOK6,KIF13B,TCF12,ASAP1,GABRB2,SH3KBP1,EPHB2,MAPK8IP2,OMG,SKOR1,DNMT3B,SHOX2,AHI1,KIDINS220,PLXNB1,GAK,SOS1,ADNP2,BLOC1S6,MAP6,BRINP1,ARF1,BCL11A,SDC2,ABL1,BRSK2*

Table S3. Overlap of rDNA-contacting genes associated with three biological processes. See Venn Diagram in Figure 1B.

Names	total	elements
cell morphogenesis nervous system developmen_list1 synaptic signaling	39	*PTN ADARB1 LAMA2 SYT1 PTK2B PTPRD NTRK2 PPP3CA CNTN4 BDNF OPHN1 ADORA2A NLGN1 ABL1 LRRK2 CDH2 SPG11 TMEM108 SLC1A3 SHANK2 CHRNA3 NRXN3 RIMS2 SYT17 SIPA1L1 S100B RIMS1 RELN NRXN1 PARK2 MAPK8IP2 SHC3 EPHB2 IL1RAPL1 TNR GSK3B APP CHRNA7 MEF2C*
cell morphogenesis nervous system developmen_list1	142	*KANK1 STK24 APBB2 WASF1 RYK SOS1 TGFB2 PAK1 BTBD3 DNM3 CTNND2 EP300 PTPRS EFNA5 XK SEMA4D EMB DPYSL2 PLXNA2 NR4A2 FBXW8 NRP1 FRY NFASC STRADB RPS6KA5 KLF7 TRPC5 KIDINS220 ARHGEF7 PALLD CPNE5 DSCAML1 DSCAM ELAVL4 PTPN11 DAB1 SEC24B DICER1 UNC5C FBXO31 FRS2 TRAK2 PTK2 MAP3K13 TTC8 BCL2 DOCK10 LRRC4C ALCAM NTN1 DCLK1 PLXNB1 BRSK2 SHROOM3 SDC2 LRP2 EPHA5 SPTA1 SEMA6D TRIOBP CNTNAP2 SEMA3D ENAH MYO16 FRYL FLRT2 ISPD NUMB GRHL2 TIAM1 NTNG1 ARHGAP44 RBFOX2 SOX6 FSTL4 STRC KIF13B HECW2 VCL ROBO2 MATN2 CHL1 VANGL2 GRIP1 COBL GAS7 FYN SH3KBP1 PREX2 ITGB1 PTPRM UNC5D KIRREL3 EPHA7 SLIT3 DOK5 DMD ROBO1 DLC1 KALRN CUX1 ANK3 HEXB SEMA5A ATP8A2 PCDH15 BCL11A SH3GL2 SHOX2 DCC FEZ2 CTNNA2 SLIT2 DOK6 PDZD7 MAP6 ENPP2 CDH11 CHN1 GLI3 COL25A1 PARD3 NTRK3 LMX1A AUTS2 NRG1 DCDC2 GAB1 CDH4 OMG WDPCP GAB2 GFRA2 NDEL1 CNTN6 TMEM106B EPHB1 SEMA3C RNF165 HECW1 PLXNA4*
nervous system developmen_list1 synaptic signaling	41	*RAP1A CLSTN1 NLGN4X MECP2 NOS1 GRIK1 CD38 ADORA1 RASGRF1 ASIC2 NRG3 ABAT GRID2 ARF1 AGT CLSTN2 CHRM1 MTMR2 PCDH17 SYBU GABRB3 MTOR GABRB1 SNAP25 TNF APBA2 SLC1A2 GABRB2 GABRA5 GRIN2B JAK2 NF1 CHRM3 BLOC1S6 TMOD2 PMP22 LRRTM1 GRIN2A ARID1B FGF14 APBA1*
nervous system developmen_list1	278	*ANKS1A PAX7 ITGAM INPP5F ATRX TACC2 CHST8 CNTN3 PRTG IER2 ITPK1 GPC4 CHRDL1 MNAT1 BBS2 ATP6V0D1 IL1RAPL2 SYNDIG1 PCDHA10 EPHA3 DPF3 SETD2 FOXP2 DENND5A EIF4E NPHP4 TNC HCN1 SYNE2 CC2D2A TRAF6 SDCCAG8 AFF2 PCDHA5 RORA SORL1 NFIA MDGA2 ATXN10 PCDHAC1 BCAN LYN HDAC4 CTNNA1 STK36 KLHL1 BASP1 SH3TC2 BMPR1A PLCB1 BPTF CHD7 TENM4 SLC8A1 DGUOK ZNF430 SOX5 PRKG1 CECR2 EML1 CDH9 SRRM4 AK8 AK4 MDM2 KCNC1 NKD1 ZNF148 C2CD3 PCDHA4 NCAM2 SKOR1 MAGI2 GPR161 THOC2 NELL1 ALK PCDH19 EZH2 ACSBG1 KDM4C CSGALNACT1 SDK1 SMAD1 ARHGEF15 CDK1 MYT1L SRGAP2B ULK4 TRAPPC9 CSF1 ZHX2 REST SMARCA4 TAGLN3 RBFOX1 CNTN1 POU6F2 RALA TENM3 OPCML LINGO2 ARF4 HMG20A COL2A1 CA10 DNMT3B NIPBL ADAM23 PRKD1 ATP5J SOX8 SYPL2 CAMSAP2 PTPRG MBD5 CELSR1 TBX3 HDAC2 POTEE THBS2 LRRC7 ETS1 LRRTM3 TCF12 TENM2 EPM2A JAG1 RARB TP73 CBFA2T2 IFT88 RUNX1 GMFB TCF4 NR2C2 GNB4 ALDH1A2 CAMK1D VWC2 MAPK9 WRN GRXCR1 HDAC9 AHI1 PCDHA3 MTPN PCDHA2 UHMK1 BRINP2 RRM1 PCDHA11 PHF8 OXCT1 DISC1 NOTCH2NL LRTM1 PPARGC1A SEZ6L CASP5 VCAN ATAT1 EYA1 SHROOM4 RORB SATB2 CDK6 NAV3 SNTG2 NDRG2 RAPGEF5 NTM RAPGEF1 ASAP1 CMA1 CIT IMMP2L ANK2 DYRK1A NCK1 LDLR PRLH PCDHA1 ATXN1 CSMD3 RCAN1 DCLK2 SIM2 EDNRB PCP4 HAPLN3 PRKCH PCDHA8 TFAP2D PHGDH SCN8A NES DLG5 NEGR1 GPM6B ASTN1 NAV2 TMC1 CLDN1 STK3 TGFBR1 TIMP2 FSHR GAS8 NCK2 MBOAT7 RFX4 PCDHAC2 GAK C21ORF91 SDK2 TRPC4 IL1RAP OVOL2 PRKACB ZNF536 FOXO3 DRP2 ZNF804A OLFM3 MAS1 CYP46A1 GART ADNP2 ETV6 MACROD2 SLC5A3 ELP3 VPS13A SUN2 TSHR BRINP1 ATIC NF2 MALL ROR1 PCDHA7 MYO1D PTPN9 HYDIN ARHGEF10 PCSK2 ABT1 HSPG2 CHODL AKT3 CRB1 NPHP3 TMEM41B SMARCE1 DNAH5 ZNF521 CAMK2G ACSL4 SDHA ATP2B1 LSAMP IGF2BP1 PCDHA6 NR2F2 BCL2A1 CTTNBP2 SLC6A17 ATRN HAPLN1 CCDC88A HMGB1 ARNT2 IGSF21 GRIN3A WLS SPOCK1 MPST*
cell morphogenesis	57	*VDR CD44 DOCK1 DMRT1 PARVG KDR TEK RHOC FMNL3 PALMD MAEL COL18A1 DIAPH1 CDC42EP3 COL21A1 RHOH FGD4 PALM2 MERTK YTHDF2 ARHGAP15 GAS2 PALM2-AKAP2 RHOJ ATP10A ITGB7 ZMYM4 STRIP1 PEAK1 MAPK14 CAP2 FBLN1 COL23A1 FRMD6 ZMYM2 ZMYM5 ZFPM1 ERMN ZRANB1 SART3 TNMD ASXL1 MATN3 MKLN1 SIPA1L3 LIPA NOX4 FLNB SPARC SCFD1 PARVB FMNL2 C21ORF2 RP2 FGD6 CAPZB COL12A1*
synaptic signaling	92	*PRKCE SLC6A2 CUBN AMPH TPRG1L EXOC4 DLG2 HTR4 CHRM5 CNR2 CALB1 SHISA6 SYN3 SLC22A2 PTPRN2 GLRA1 GRM8 RNF216 SQSTM1 SYT6 DGKI USP46 CHRNA5 HTR2C PPFIA2 CACNA1E HTR7 SYT10 SNAP23 DNAJC5 PLCL1 GLRA2 SLC1A1 SORCS3 GRM5 PLCL2 CACNG3 GABRR2 GRIK2 SHISA9 CHRFAM7A HRH1 DBH CACNA1B GRIK4 CDH8 GABRG1 GRIK3 CACNB2 PFN2 KCNC4 SYNPR SV2B GRM7 P2RX6 RIMS3 RIMBP2 CADPS DLGAP1 UNC13C RAB5A SCGN LIN7A GRIA1 NOVA1 GPR176 CNTNAP4 CACNG2 NSG1 GABBR2 KCND2 GABRG3 SV2C GABRA2 GRIA4 CNIH3 GRM1 TRIM9 FCHSD2 SLC24A2 GRIA3 SYT9 JPH3 GABRA3 CASK PXK CEP89 DTNA CELF4 GRID1 MCTP1 PGK1*

Table S4. rDNA-contacting genes associated with Jensen Compartments. Related to Figure 2A.

Index	Name	# of genes	Genes
1	Synapse	243	*APP;FRMPD4;ITSN1;ARR3;SYNPR;OPHN1;RUSC1;DPYSL2;ANKS1B;PRIMA1;UNC13C;EPHA7;MEF2C;CXADR;SLC6A17;ACTN2;UNC5C;ANK2;ANK3;SYTL5;MAPK8IP2;GABRG3;GABRG1;TIAM1;TLN2;ATP6V0D1;PFN2;KCTD16;MTMR2;CTBP2;SLC22A2;PCDH15;TPRG1L;KALRN;FXR1;GRIN2A;BLOC1S6;FLRT2;VTI1A;SYNDIG1;GSG1L;STXBP5;LYN;PTPRN2;DTNA;CADM2;IQSEC3;GRIN2B;DCLK1;CPEB1;GRIN3A;SSPN;CPEB2;GABRB3;GABRB2;ARF4;ARF1;GABRB1;TENM2;HIP1;SH3KBP1;DENND1A;KCNC4;GRIK3;LRRC4;GRIK4;GRIK1;GRIK2;MECP2;PPP3CA;GABRR2;TRIM9;SIPA1L1;DLGAP1;EMB;NDEL1;MME;KCND2;SYN3;SORCS3;ZDHHC17;ENAH;CNKSR2;PLCB4;LRRC7;PDZRN3;DSCAML1;RAB7A;PTN;TMEM163;DRP2;GLRA1;GLRA2;EGFLAM;APBB2;DMD;CTNNA2;DISC1;CAP2;GABRA2;GABBR2;GABRA5;GABRA3;SAMD4A;RIMBP2;VWC2;GOPC;APBA1;APBA2;CHRM3;RAB3C;CHRM1;CLSTN2;CHRM5;CLSTN1;COMT;CDH8;PARK2;SLC8A1;RPH3A;RIMS2;ARHGAP44;RIMS1;GRIP1;SCGN;RIMS3;CDH2;ADORA1;SH3GL2*

			;KCNH1;PPFIA2;NSF;DSCAM;MAGI2;CASK;SYPL2;SHISA9;SHISA6;KCTD8;TANC1;CACNB4;OLFM3;PKP4;AMPH;UTRN;DGKI;GRIA1;CHRNA3;CHRNA5;CTTNBP2;CHRNA7;CACNA1B;LIN7A;CACNA1C;CNN3;NPHP4;LRRC4C;ASIC2;GRIA3;GRIA4;MPST;CEP112;SYT1;NSG1;SYT9;SYT6;SDK1;ARHGAP32;LRFN2;DAB1;DLG2;DMXL2;MDM2;NF1;CPE;CNIH3;RAB5A;SDK2;BCL2L1;ITGB1;SNAP25;GSK3B;LRRK2;SNAP23;GPHN;GRM1;SYNE1;GRM5;CALB1;GRM7;LRRTM3;LRRTM4;LRRTM1;BAI3;GRM8;PSD3;NCK2;PHACTR1;NOS1;ERC1;ERC2;SPG11;SNTA1;GRID2;GRID1;NRG1;MYRIP;DNM3;FCHSD2;DNAJC6;DNAJC5;IL1RAPL1;SOS1;SHANK2;CNTNAP4;HDAC4;NLGN1;DDC;LAMA2;NRXN1;NRXN3;ASAP1;ATP1A1;ZNRF1;SV2C;PALMD;SV2B;IGF2BP1;SPOCK1;PTK2B;FYN;WASF1;CAMK2G;NTRK2;NLGN4X;CADPS;ATP2B1;SYT17;SYT10;RGS12;CBLN4;KCNK1;SNTB1
2	Synapse_part	192	CHRM3;RAB3C;APP;CHRM1;CLSTN2;FRMPD4;CHRM5;CLSTN1;COMT;CDH8;PARK2;SLC8A1;RPH3A;RIMS2;ARHGAP44;RIMS1;GRIP1;SYNPR;RIMS3;OPHN1;RUSC1;DPYSL2;ADORA1;KCNH1;ANKS1B;PPFIA2;NSF;UNC13C;EPHA7;MEF2C;SLC6A17;ACTN2;MAGI2;CASK;ANK2;SYPL2;ANK3;SYTL5;MAPK8IP2;SHISA9;GABRG3;GABRG1;KCTD8;TANC1;TIAM1;PKP4;AMPH;ATP6V0D1;UTRN;DGKI;PFN2;KCTD16;GRIA1;MTMR2;CHRNA3;CHRNA5;CTTNBP2;CHRNA7;SLC22A2;CACNA1B;TPRG1L;LIN7A;CACNA1C;KALRN;CNN3;FXR1;GRIN2A;BLOC1S6;VTI1A;SYNDIG1;STXBP5;LRRC4C;ASIC2;GRIA3;GRIA4;LYN;PTPRN2;SYT1;IQSEC3;NSG1;SYT9;GRIN2B;DCLK1;SYT6;ARHGAP32;LRFN2;CPEB1;GRIN3A;DAB1;DLG2;DMXL2;SSPN;NF1;CPE;CNIH3;RAB5A;BCL2L1;GABRB3;ITGB1;SNAP25;GABRB2;ARF4;GSK3B;ARF1;GABRB1;TENM2;HIP1;DENND1A;LRRK2;SNAP23;GRIK3;LRRC4;GRIK4;GRIK1;GRIK2;GPHN;GRM1;SYNE1;MECP2;PPP3CA;GABRR2;TRIM9;GRM5;SIPA1L1;CALB1;GRM7;LRRTM3;LRRTM4;LRRTM1;BAI3;GRM8;PSD3;DLGAP1;NOS1;ERC1;ERC2;NDEL1;GRID2;MME;KCND2;GRID1;SYN3;SORCS3;ZDHHC17;DNM3;CNKSR2;PLCB4;LRRC7;DNAJC6;DNAJC5;IL1RAPL1;SOS1;SHANK2;CNTNAP4;RAB7A;NLGN1;DDC;LAMA2;NRXN1;NRXN3;ASAP1;ATP1A1;TMEM163;DRP2;ZNRF1;GLRA1;GLRA2;SV2C;PALMD;SV2B;IGF2BP1;SPOCK1;PTK2B;DMD;FYN;CTNNA2;DISC1;CAMK2G;CAP2;GABRA2;GABBR2;NTRK2;NLGN4X;GABRA5;GABRA3;CADPS;ATP2B1;SYT17;SYT10;GOPC;APBA1;APBA2
3	Postsynapse	130	CHRM3;APP;CHRM1;CLSTN2;FRMPD4;CHRM5;CLSTN1;COMT;SLC8A1;ARHGAP44;GRIP1;OPHN1;RUSC1;ADORA1;ANKS1B;NSF;EPHA7;MEF2C;ACTN2;MAGI2;ANK2;ANK3;MAPK8IP2;GABRG3;GABRG1;KCTD8;TANC1;TIAM1;PKP4;UTRN;DGKI;KCTD16;GRIA1;MTMR2;CHRNA3;CHRNA5;CTTNBP2;CHRNA7;LIN7A;CACNA1C;KALRN;CNN3;FXR1;GRIN2A;SYNDIG1;LRRC4C;ASIC2;GRIA3;GRIA4;LYN;IQSEC3;NSG1;GRIN2B;DCLK1;ARHGAP32;LRFN2;CPEB1;GRIN3A;DAB1;DLG2;SSPN;CNIH3;GABRB3;ITGB1;GABRB2;ARF4;GSK3B;ARF1;GABRB1;TENM2;HIP1;LRRK2;GRIK3;LRRC4;GRIK4;GRIK1;GRIK2;GPHN;GRM1;SYNE1;MECP2;PPP3CA;GABRR2;GRM5;SIPA1L1;GRM7;LRRTM3;LRRTM4;LRRTM1;BAI3;PSD3;DLGAP1;NOS1;GRID2;KCND2;GRID1;SYN3;SORCS3;DNM3;CNKSR2;PLCB4;LRRC7;DNAJC6;IL1RAPL1;SOS1;SHANK2;NLGN1;LAMA2;ASAP1;ATP1A1;DRP2;GLRA1;GLRA2;PALMD;IGF2BP1;SPOCK1;PTK2B;DMD;FYN;CTNNA2;DISC1;CAMK2G;CAP2;GABRA2;GABBR2;NTRK2;NLGN4X;GABRA5;GABRA3;GOPC
4	Synaptic_membrane	99	CHRM3;CHRM1;CLSTN2;CHRM5;CLSTN1;COMT;RIMS2;RIMS1;GRIP1;RUSC1;ADORA1;KCNH1;ANKS1B;UNC13C;EPHA7;CASK;ANK2;ANK3;SHISA9;GABRG3;GABRG1;KCTD8;TANC1;UTRN;DGKI;KCTD16;GRIA1;MTMR2;CHRNA3;CHRNA5;CHRNA7;LIN7A;GRIN2A;SYNDIG1;LRRC4C;GRIA3;GRIA4;SYT1;IQSEC3;NSG1;GRIN2B;ARHGAP32;LRFN2;CPEB1;GRIN3A;DLG2;SSPN;CPE;CNIH3;GABRB3;ITGB1;SNAP25;GABRB2;ARF1;GABRB1;TENM2;DENND1A;GRIK3;LRRC4;GRIK4;GRIK1;GRIK2;GPHN;SYNE1;GABRR2;SIPA1L1;GRM7;LRRTM3;LRRTM4;LRRTM1;GRM8;PSD3;DLGAP1;ERC1;ERC2;GRID2;KCND2;GRID1;ZDHHC17;CNKSR2;LRRC7;IL1RAPL1;SHANK2;CNTNAP4;NLGN1;NRXN1;DRP2;GLRA1;GLRA2;DMD;DISC1;GABRA2;GABBR2;NTRK2;NLGN4X;GABRA5;GABRA3;ATP2B1;GOPC
5	Postsynaptic_membrane	78	GABRB3;GABRB2;CHRM3;ARF1;GABRB1;TENM2;CHRM1;CLSTN2;CHRM5;CLSTN1;GRIK3;LRRC4;GRIK4;GRIK1;GRIK2;COMT;GPHN;SYNE1;GABRR2;GRIP1;SIPA1L1;GRM7;LRRTM3;RUSC1;LRRTM4;PSD3;ADORA1;DLGAP1;ANKS1B;EPHA7;GRID2;KCND2;GRID1;ANK2;ANK3;GABRG3;GABRG1;KCTD8;CNKSR2;TANC1;LRRC7;IL1RAPL1;UTRN;SHANK2;KCTD16;GRIA1;NLGN1;CHRNA3;CHRNA5;CHRNA7;LIN7A;DRP2;GLRA1;GLRA2;GRIN2A;SYNDIG1;DMD;LRRC4C;DISC1;GRIA3;GRIA4;GABRA2;GABBR2;NTRK2;NLGN4X;GABRA5;IQSEC3;GABRA3;NSG1;GRIN2B;ARHGAP32;LRFN2;CPEB1;GRIN3A;DLG2;SSPN;GOPC;CNIH3

Table S5. Overlap of rDNA-contacting genes associated with three Jensen Compartments. Related to Venn Diagram in Figure 2B.

Names	total	elements
postsynapse synapse synaptic membrane	82	GLRA2 GABRB3 GPHN DISC1 GABRA2 GRIA4 CNIH3 GRM7 LRRC4C ARHGAP32 SYNDIG1 CLSTN1 GABRB1 SHANK2 DRP2 ANKS1B GABRR2 GRIP1 NLGN4X LRRTM1 DLG2 GRIN2A IQSEC3 CHRM5 ANK2 NLGN1 ITGB1 GRIA3 KCTD8 GOPC GRIK2 SSPN LRRTM4 LRRC4 CPEB1 CHRNA3 DLGAP1 GRID2 LRRC7 IL1RAPL1 ARF1 GLRA1 GABRA3 LRRTM3

		CLSTN2 EPHA7 SYNE1 GABRB2 GABRA5 NTRK2 TENM2 GRIN2B CHRM1 UTRN LIN7A TANC1 DMD CNKSR2 GRIK1 GRIA1 GRIK4 KCTD16 DGKI CHRNA5 GABRG1 COMT GRIK3 SIPA1L1 ANK3 GRID1 NSG1 CHRM3 PSD3 GABBR2 MTMR2 KCND2 CHRNA7 LRFN2 GRIN3A ADORA1 RUSC1 GABRG3
postsynapse synapse	48	*DNAJC6 OPHN1 SORCS3 CACNA1C ASIC2 GRM1 SOS1 PALMD CAP2 CTNNA2 LAMA2 GRM5 DNM3 ARF4 HIP1 DCLK1 ASAP1 FRMPD4 FYN BAI3 PTK2B LRRK2 NSF MAPK8IP2 SYN3 LYN MECP2 NOS1 GSK3B CNN3 PLCB4 ACTN2 SLC8A1 KALRN CAMK2G PKP4 PPP3CA APP DAB1 IGF2BP1 TIAM1 CTTNBP2 ARHGAP44 FXR1 MAGI2 ATP1A1 MEF2C SPOCK1*
synapse synaptic membrane	17	*ERC2 DENND1A SYT1 RIMS1 SNAP25 NRXN1 SHISA9 CASK UNC13C GRM8 RIMS2 ERC1 ATP2B1 CNTNAP4 CPE ZDHHC17 KCNH1*
synapse	96	*SYTL5 SV2C KCNC4 PFN2 SH3GL2 SAMD4A GSG1L SYNPR TMEM163 PTN APBB2 SV2B WASF1 SDK2 AMPH CADM2 ATP6V0D1 RGS12 TRIM9 ITSN1 PDZRN3 CTBP2 TPRG1L FCHSD2 OLFM3 APBA2 VTI1A NPHP4 MME SYPL2 EMB DPYSL2 SH3KBP1 RIMS3 BCL2L1 CEP112 RIMBP2 NRG1 CALB1 RAB3C SHISA6 PARK2 SYT9 CADPS MYRIP SLC22A2 CXADR CACNB4 PTPRN2 CDH2 TLN2 HDAC4 NRXN3 DTNA RAB5A SYT17 SCGN CACNA1B CPEB2 SYT6 NDEL1 DSCAML1 CDH8 STXBP5 DSCAM DDC CBLN4 ENAH DMXL2 NF1 EGFLAM MDM2 ARR3 VWC2 FLRT2 SPG11 RPH3A SNTA1 PPFIA2 SYT10 SNAP23 ZNRF1 SLC6A17 UNC5C PRIMA1 NCK2 PHACTR1 RAB7A DNAJC5 BLOC1S6 SNTB1 PCDH15 SDK1 KCNK1 MPST APBA1*

Table S6. rDNA-contacting genes associated with Jensen Diseases. Related to Figure 2C.

Index	Name	# of genes	Genes
1	Kidney_cancer	905	*ZNF292;ITSN1;SUV420H1;PREX2;PPP4R4;NDST3;GBP6;MEF2C;RBFOX1;CACNA2D1;CACNA2D3;UNC5C;DICER1;HSPG2;PHKA2;EML1;SND1;CNBD1;EML5;CLOCK;ABCB5;CEP85L;AGAP1;BAZ2B;ADAMTS16;GRIN2A;C6;RNF213;ADAMTS18;ADAMTS17;MOV10L1;JAG1;COL24A1;CADM2;LRBA;ABCA9;BTBD11;ABCA8;GRIN2B;HRNR;PCDHGA11;PPFIBP1;FAM135B;PCDHGA12;GRIN3A;FAT3;EZH2;LRRC4;EFCAB5;ARHGAP5;TRIOBP;NIPBL;ATIC;JPH3;JAK2;NINL;CHST9;ZNF483;KCND2;KCND3;TEX11;RIPK4;BAZ1B;TMC5;LRRC7;DPYD;PXDN;KCNMA1;DSCAML1;CRB1;SAMD9;COL11A1;KCNA3;KIAA2022;AXDND1;FSTL5;WRN;TUBA3C;DMD;FLNB;FLNC;CSMD3;CSMD2;CSMD1;ZSWIM3;EVC2;MBD5;ATRNL1;KCNB2;TRAPPC10;COL22A1;ATAD2;SAMD4A;STXBP5L;TAOK3;RIMBP2;LGR4;FRYL;ZNF454;ZNF451;PLOD2;FRY;SLC9C1;MYLK3;SMCHD1;HEPHL1;ZNF208;SLC16A7;MAP3K7;ROS1;ZNF443;MAP3K5;KCNH1;KCNH5;KCNH7;TNNI3K;STON1-GTF2A1L;SLC9A9;PKP2;PKP4;ROR1;ZNF677;UTRN;GRIA1;GLIS3;STK31;LOXHD1;ATXN1;STK36;ZNF429;ZBBX;CPAMD8;SLCO1B3;ZNF667;LRRC4C;MDN1;GRIA3;GRIA4;ZNF420;KL;HPS3;INADL;CORO2B;QKI;TMPRSS15;PTPRD;DMXL2;CNTNAP2;PIWIL4;FLT1;NUMA1;FLT3;RASGRF2;LRRK2;LRRK1;RASGRF1;FAM208B;BMP15;PPP6R2;HMG20A;ERC2;SPG11;CTNNBL1;CERS3;GRID2;OVCH1;GRID1;VPS13D;ATRX;VPS13A;VPS13B;NRG1;SEZ6L;ATRN;NPC1;NRG3;PCCA;IL1RAPL2;MYO3A;IL1RAPL1;BIRC6;PIK3C3;TOP1;ST6GALNAC3;DSG4;CNTNAP5;CNTNAP4;COL15A1;FOCAD;ROCK2;ATP10B;ATP10A;LRP2;ZBTB41;EHBP1;KIAA1324;FYN;ATP9B;ATP9A;LINGO2;CIZ1;SEC24B;PCDH9;NTRK3;CYP4B1;SORL1;MYO16;MYO1D;NELL2;NELL1;MCM3AP;ZNF615;MDGA2;UNC79;EIF3A;THSD7B;ULK4;DHX57;KDR;PTGFRN;ZNF721;EPHB2;ACAD11;SCAPER;EPHB1;TBC1D22A;TRIM22;EPHA5;ACTN2;ANK2;ANK3;FNDC1;KCNQ5;MXRA5;EPHA3;PCDH15;PCDH19;KALRN;PCDH17;PKHD1;HIVEP3;HIVEP2;A2M;NTNG1;BBS2;GREB1;DCLK1;PTK2;MED13L;SMARCA4;TF;RGL1;KIAA0825;FBXL7;SPAG16;SPAG17;PTPRU;PTPRS;CSRNP3;GRIK3;GRIK4;MIA2;PTPRM;GRIK1;GRIK2;PTPRG;AKAP13;KCNT2;BBS9;MME;GAB1;SORCS1;ANO4;SORCS3;ANO2;CNKSR2;ANKFN1;BANK1;FSHR;CYLC2;DEPDC5;ARHGEF7;PDZRN3;SFI1;PDZRN4;RELN;OS9;PAK7;GPC5;PCNT;GPC6;DYNC1I1;ZNF585A;GABBR2;DYNC1H1;MYO9A;SGSM1;NLRP13;NLRP12;KIF26B;TTC3;CACHD1;TACC2;MAMLD1;IL18R1;OTC;OTUD4;FHOD3;PRDM9;UBE3C;PCDH11X;KLHL32;CELSR1;IGF1R;NR3C2;DMBT1;GNPTAB;SCN9A;ENPP2;EP300;DENND5A;ARID2;RAG1;TGM2;PPFIA2;TPTE2;PDGFRA;HFM1;CD93;TANC1;ADAM17;ZNF91;ADAM12;SIDT1;WDFY3;RAF1;RTN1;CACNA1B;PLD5;SLC1A3;CACNA1D;PDS5B;CACNA1C;PLD1;CACNA1E;SPTA1;CALD1;CAMTA1;RHPN2;MGAM;TFAP2D;AUTS2;CNTN5;MUC16;CNTN6;IGF2R;ANKHD1;SDK1;PAN3;CNTN1;CNTN3;CNTN4;FBN1;GSK3B;MAST4;CPXM2;MAST2;CHD7;NGLY1;CHD6;AFF3;GPHN;GRM1;AFF2;CABIN1;SGOL2;GRM5;GRM7;MYO18B;GRM8;ZNF385D;ARHGEF10;RBM19;SHROOM3;NAV2;ETV6;VCAN;SETBP1;TTC40;ACOX3;LPHN2;LPHN3;DNAH3;DNAH2;LAMA2;DDX24;DNAH8;LAMA4;DNAH5;LAMA3;PKD1L1;ASAP1;DNAH9;SPOCK3;MAN1C1;TAS1R2;SLIT3;SLIT2;ATF7IP;CCDC178;URGCP;EYA4;LAMB4;ZNF804A;REST;CCDC170;ACIN1;TEK;ERCC6;TAF4;RBM44;CCDC171;IPO13;TRIO;WIPF1;MYT1L;COL12A1;FRMPD2;ABAT;ABCA12;SLC4A4;ABCA13;BACH1;RXFP2;SCN11A;CSPP1;PLCE1;DIP2C;ANKS1A;NCKAP5;CSRP2BP;ANKS1B;CUBN;UNC13C;PRKCB;REV1;FNDC3B;KIAA1462;AFAP1;WDR72;ARMC8;FNDC3A;GABRG3;DKK2;GABRG1;KIAA1217;ADCY9;TEP1;HECW2;PARD3;HECW1;PRKD1;ASTN2;FREM1;FREM2;ASTN1;TPH2;FLRT2;PCDHA1;CHST15;PCDHA5;PCDHA4;PCDHA3;PCDHA2;PCDHA9;PCD*

			HA8;PCDHA7;PCDHA6;DNAH10;PRRC2C;INO80;PLCH1;GALNTL6;CDK12;PHF3;GABRB3;GABRB2;MYOM1;SETD2;SETD3;SPEF2;DENND1A;FMN1;FMN2;PYGL;SMG7;ABCC10;PHEX;GLI3;ADAMTSL1;TRIM9;CHL1;ADAMTSL3;RNF150;GPR133;BRD1;FCRL3;TCF12;TACR3;CIT;WDR33;NCOR1;COL2A1;EXOC4;GPR126;RARB;EXOC6;PLCB1;GPR125;OCA2;NBAS;PRUNE2;FIGN;ADCY2;ADCY8;FAM214A;BCLAF1;SCAF8;FGD6;ERMP1;NSD1;ABI3BP;LDLR;ZNF345;GPR158;RUNX1T1;BRD4;GABRA2;ZNF583;GABRA5;KIDINS220;SCAF4;KIAA0195;MKLN1;NFIA;ALMS1;MSRB3;SEMA5A;ZNF573;COL16A1;ATP8A2;ATP8A1;CLSTN2;TUSC3;DGKB;CTNND2;CLSTN1;TNC;DACH2;CDH9;CDH8;SLC8A1;RPH3A;CDH6;RIMS2;RIMS1;CDH4;CDH2;MAEL;TNR;ZNF569;ZNF568;PCDHAC2;ZNF564;PCDHAC1;MAGI1;MAP2K4;RNF43;COL27A1;CEP135;USP6;RALGAPA2;SEMA6D;USP3;MAGI2;FRMD4A;MITF;THOC2;DISP1;ADAM2;CACNB2;CLIP1;ILF3;OBSCN;OR2L13;PHLDB2;DGKI;DGKH;DNMT1;CUL2;ITPR2;GTF2E1;TPO;PLXNA2;ABL1;KIAA1407;PLXNA4;IMPG1;TMEM132D;ZNF540;ZNF781;ATP8B4;CD163;OSBPL5;TMEM132B;ESRRG;NEB;PHKB;TRAPPC8;PXDNL;TRAPPC9;TECRL;BCL9;LRFN5;NBEA;CNOT1;ZFP64;NF1;TPP2;CPE;ZNF536;PLXNB1;PCNXL2;NF2;LZTR1;MAP3K13;NES;EIF4G3;DPP10;ALK;DIDO1;SYCP2;KIAA0100;C1ORF173;SYNE2;SYNE1;ADAMTS2;SART3;BAI3;DNMT3B;HYDIN;RNF17;CEP170;SGCG;PRKG1;TRPC5;PLEKHG1;CR1;TRPC4;COBL;DUSP27;C1ORF168;CATSPERB;POLR1A;PKHD1L1;SOS1;SLC24A2;NLGN1;PLEKHH2;SLC24A3;SEMA3D;TPTE;KIAA1211;JAKMIP1;HDAC9;NHS;ASCC3;CACNG3;NLGN4X;SEMA4D;CEP152;MACC1;PTPN11;CDC42BPA;MTOR;ASXL1;MGAT4C;NFASC;FRAS1;APC;KIAA0586;ASXL3;VPS41;TNRC6B;HPSE2;CXORF22;CBLB;PWP1;RPS6KA5;EDNRB;ZNF607;CHEK2;RPS6KA2;HMCN1;SOX6;TNS3;EYS;TNS1;SOX5;MTUS2;ACSL4;SRRM1;RUNX1;TIAM2;TIAM1;DOPEY2;WBSCR17;PAPPA;CD109;MAGEB2;WDPCP;TLN2;SIK2;ZFPM2;ATP6V0D2;OR4C15;CFTR;PTGFR;RWDD2B;IGSF3;PDE1C;TSHZ1;TSHZ2;TRANK1;TMTC1;PPL;TMTC4;FXR1;TRPM2;INPP5F;ASUN;MAP7;MAGEC3;TRPM7;TRPM3;BCL11A;CCDC88A;STAG1;STAG2;ATR;INVS;ROBO2;RYR2;ZCCHC11;TENM2;TENM3;C2CD3;TENM4;SIPA1L2;SIPA1L3;RYR3;ROBO1;RPTOR;SIPA1L1;ZMYM4;ZMYM5;SH3PXD2B;KIF13B;VAV3;LRRIQ3;STRBP;CYP7B1;PARP8;VWA8;NFX1;NME9;MATN2;GUCY2C;AKAP6;GUCY2F;NOL4;DRP2;C10ORF90;CUX1;KIAA0753;CTNNA1;CTNNA3;CTNNA2;HDX;CORIN;MAP4K3;ARNT2;DLC1;APBA2;GALNT14;KIAA1199;DOCK10;IL18RAP;SACS;TRAPPC12;SVEP1;DSCAM;LMO7;PCDHA13;PCDHA12;PCDHA11;TMEM131;PCDHA10;KIF16B;SPECC1L;COL4A6;ELMO1;AMPH;USP53;NTM;FBLN1;ARHGAP15;SEL1L2;TRHDE;MARCH10;ARHGAP12;ARHGAP22;ARHGAP20;PDZD2;DPP8;NLRP7;LARGE;SOHLH2;MYH13;CDH26;ZC3H13;BPTF;SPECC1;SMAD3;WSCD2;ARHGAP29;ESR1;LRP1B;TDRD3;ARHGAP32;DIAPH2;DLG2;DLG5;PI4KA;B4GALT3;DOCK4;PCDHGB7;DOCK3;PCDHGB6;NVL;SEPT9;HK3;CASP8;LRRTM3;LRRTM1;TRPS1;AOX1;NOS1;POLE;PCDHGA7;ABCC1;PCDHGA6;PCDHGA5;STPG2;DCC;PCDHGA3;PCDHGA1;ARAP2;ABCC9;ARID1B;TGFBR1;DNM3;TGFBR3;RBL2;MYH1;ALDH1A2;CDH10;CDH11;MYH8;CDH12;COL6A5;DOCK2;MYH4;CDH18;COLEC12;SLFN5;CD163L1;NRXN1;ATP1A4;NRXN3;THBS2;UACA;PDE11A;NCAM2;OPCML;TCF7L2;KDM4C;CADPS;PDE4DIP;OR6N1;TSHR;PDE10A;RGS12;LPIN3;HCN1
2	Liver_cancer	265	*TRIO;THSD7B;COL12A1;ABCA13;PREX2;EDNRB;KDR;PLCE1;FAM65B;HMCN1;EYS;EPHA5;ADGB;CUBN;UNC13C;EPHA6;PRKCB;STARD9;CACNA2D3;UNC5C;ANK2;ANK3;DICER1;HSPG2;SND1;KIAA1217;DOPEY2;ZNF717;PAPPA;PARD3;HECW1;TLN2;PRKD1;PRR12;ZFPM2;FREM1;MXRA5;FREM2;ABCB5;TRANK1;PCDH15;BAZ2B;KALRN;PKHD1;RNF213;FLRT2;MYO6;PCDHA5;HIVEP3;PCDHA2;PCDHA7;DNAH10;COL24A1;CADM2;DNAH14;WDR52;PRRC2C;MED13L;SMARCA4;TF;FAT3;SLC44A5;ROBO2;GABRB2;RYR2;SETD2;TENM2;PTPRS;SPEF2;TENM3;TENM4;DENND1A;GRIK4;FMN1;EFCAB5;GRIK1;FMN2;ARHGAP5;RYR3;GLI3;ROBO1;ADAMTSL1;NIPBL;JPH3;ZNF366;VAV3;MME;SLC39A10;SORCS1;ANO4;C12ORF55;WDR33;MMP16;PXDN;FAM193A;PLCB1;DEPDC5;PDZRN3;NBAS;COL11A1;PRUNE2;KIAA2022;ADCY2;ADCY8;TM9SF4;FSTL4;FSTL5;RELN;CUX1;PAK7;DMD;FLNB;CSMD3;CSMD2;CSMD1;GPR158;RUNX1T1;EVC2;DYNC1H1;COL22A1;PLEKHA5;MYO9A;SCAF4;STXBP5L;AGBL1;ALMS1;APBA2;FHOD3;CTNND2;FRY;CELSR1;CDH9;SLC9C1;CDH8;IGF1R;RIMS2;SMCHD1;SACS;TNR;SVEP1;ARID2;PCDHAC2;KCNH5;DSCAM;MAGI2;PCDHA13;TANC2;ZNF91;OBSCN;SPECC1L;KIAA1731;PKP4;WDFY3;AMPH;UTRN;GRIA1;CUL5;UBR3;CACNA1C;CACNA1E;STK31;SPTA1;CACNA1I;SNTG2;PDZD2;NLRP7;MYH13;PLXNA4;MDN1;FAM83B;GRIA4;BPTF;TMEM132D;MGAM;MUC16;CNTN6;NEB;LRP1B;ANKHD1;SDK1;LRFN2;LRFN5;DIAPH3;NBEA;CNOT1;DMXL2;NF1;CNTN1;ZNF536;NES;CNTNAP2;DOCK4;MCTP1;SYCP2;NUMA1;MAST4;MCM9;CHD6;EFCAB11;SYNE2;AFF2;SYNE1;GRM7;LRRTM1;BAI3;MYO18B;GRM8;HYDIN;ERC2;PCDHGA7;ABCC1;GRID1;DCC;PCDHGA2;VPS13D;ATRX;VPS13A;VPS13B;COBL;RBL2;VCAN;NAV3;NRG3;PEAK1;CDH11;TTC40;BIRC6;PKHD1L1;COL6A5;MCTP2;DOCK2;LPHN3;CDH18;CNTNAP5;DNAH3;DNAH2;LAMA2;DNAH8;NRXN1;DNAH5;DNAH6;NRXN3;PKD1L1;ATP10A;TPTE;DNAH9;KIAA1211;LRP2;THBS2;EHBP1;PRTG;CECR2;NTRK2;PLCL1;PCDH7;MACC1;PDE4DIP;CDC42BPA;FAM155A;FRAS1;APC;ASXL3;MDGA2;UNC79;TNRC6B*
3	Skin_cancer	212	*ABCA12;PREX2;SCN11A;NDST4;KDR;HMCN1;EPHB2;CUBN;ANK2;ANK3;KIAA1217;SPATA16;HECW2;PAPPA;KCNQ5;ASTN2;MXRA5;FREM2;CFTR;EPHA3;ASTN1;KCTD16;PDE1C;ABCB5;PCDH15;CD1C;KALRN;PKHD1;GRIN2A;C6;PCDHA1;ADAMTS18;PCDHA5;MOV10L1;PCDHA8;PCDHA7;TRPM3;MSR1;NEBL;STAC;CADM2;GRIN2B;A1CF;MED13L;ISX;FAM135B;GRIN3A;PAH;COL9A1;PLCH1;SPAG17;PTPRS;FMN2;GRIK2;EFCAB6;C8A;TRIOBP;KCNT2;CHL1;ADAMTSL3;CG*

			NL1;ANO4;SORCS3;SLC22A25;COL2A1;LRRC7;DPYD;PLCB1;CYLC2;NPSR1;CRB1;COL11A1;KIAA2022;AKAP6;ADCY8;SNX31;FSTL5;ADAM28;RELN;TUBA3C;PAK7;DMD;CTNNA3;FLNB;CSMD3;CSMD2;CORIN;ATRNL1;KCNB2;COL22A1;NLRP13;NLRP12;TACC2;CHRM3;GALNT14;ATP8A1;CLSTN2;FAM13C;TNC;KIAA1199;CDH9;CDH8;CDH6;TNR;ARID2;ROS1;PCDHAC2;RGS7;MAGI1;PDGFRA;KCNH5;KCNH7;MAGI2;THEMIS;PCDHA13;PCDHA10;COL4A6;PHLDB2;DGKI;DSC3;GRIA1;KHDRBS2;CACNA1D;STK31;TPO;LOXHD1;ATXN1;SLCO1B3;NLRP2;SRGAP3;LRRC4C;FAM83B;TMEM132D;KL;ATP8B4;CD163;TMEM132B;ARHGAP29;LRP1B;PTPRD;DMXL2;NF1;ZNF536;TCF4;CNTN4;ALK;CNTNAP2;FLT1;FLT3;RASGRF2;LRRK2;CHD6;C1ORF173;SYNE1;GRM7;BAI3;GRM8;HYDIN;RNF17;AOX1;ZNF385D;ADAMTS6;GRID2;DCC;VPS13D;VPS13A;VPS13B;ABCC9;NAV2;SEZ6L;C1ORF168;MYH1;VCAN;CATSPERB;NRG3;CDH10;MYH8;BIRC6;LPHN2;DOCK2;MYH4;DSG4;CDH18;DNAH3;COL15A1;PLEKHH2;DNAH2;CD163L1;LAMA2;DNAH8;DNAH5;PKD1L1;TPTE;DNAH9;LRP2;PTK2B;SLIT3;SLIT2;CACNG3;CADPS;PLCL2;LAMB4;ZNF804A;SORL1;TBX15;NELL1;NFASC;APC;SYT10;TEK;CCDC60;UNC79
4	Melanoma	224	ACSM3;ABCA12;HTR4;PREX2;SCN11A;LIPI;NDST4;PPP4R4;KDR;HMCN1;EPHB2;TNS1;RBFOX1;ANK2;ANK3;KIAA1217;SPATA16;MTAP;WBSCR17;SLC5A8;HECW2;PAPPA;KCNQ5;ASTN2;MXRA5;FREM2;CFTR;ASTN1;PDE1C;TSHZ2;ABCB5;PCDH15;CD1C;KALRN;PKHD1;TRPM2;GRIN2A;C6;PCDHA1;ADAMTS18;PCDHA5;MOV10L1;PCDHA9;PCDHA7;TRPM3;MSR1;DNAH10;NEBL;STAC;CADM2;GRIN2B;A1CF;MED13L;ISX;GRIN3A;BPIFB1;PLCH1;SPAG16;SPAG17;PTPRS;GRIK1;FMN2;GRIK2;EFCAB6;C8A;PCSK5;TRIOBP;SIPA1L1;KCNT2;CHL1;ADAMTSL3;CGNL1;NBPF10;ANO4;SORCS3;SLC22A25;COL2A1;DPYD;PLCB1;CYLC2;NPSR1;CRB1;COL11A1;KIAA2022;AKAP6;ADCY8;SNX31;FSTL5;ADAM28;RELN;TUBA3C;PAK7;DMD;CTNNA3;GPC5;FLNB;CSMD3;CSMD2;SAMD3;KCNB2;COL22A1;NLRP13;NLRP12;TACC2;CHRM3;GALNT14;CLSTN2;FAM13C;CTNND2;TNC;CDH9;CDH8;CDH6;TNR;RAG2;ARID2;ROS1;PCDHAC2;RGS7;MAGI1;KCNH5;KCNH7;MAGI2;THEMIS;LMO7;PCDHA13;ADAM2;COL4A6;PHLDB2;DGKI;DSC3;FTO;GRIA1;KHDRBS2;CACNA1D;STK31;TPO;LOXHD1;ATXN1;SLCO1B3;NLRP2;SRGAP3;LRRC4C;FAM83B;ATP8B4;CD163;TMEM132B;ARHGAP29;LRP1B;PTPRD;SDK1;NF1;TCF4;ALK;CNTNAP2;FLT1;RASGRF2;LRRK2;CRISP3;C1ORF173;ACSM2B;SYNE1;GHR;GRM7;BAI3;GRM8;HYDIN;AOX1;ZNF385D;EDIL3;ADAMTS6;GRID2;DCC;PCDHGA2;VPS13D;VPS13A;VPS13B;ARNT;ABCC9;SEZ6L;C1ORF168;MYH1;VCAN;CATSPERB;NRG3;CDH10;MYH8;BIRC6;LPHN2;MCTP2;DOCK2;MYH4;DSG4;CDH18;DNAH3;PLEKHH2;DNAH2;CD163L1;LAMA2;DNAH8;DNAH5;NRXN3;PKD1L1;TPTE;DNAH9;LRP2;SPOCK3;PTK2B;SLIT3;SLIT2;CACNG3;CADPS;PLCL2;LAMB4;ZNF804A;SORL1;TBX15;NELL1;NFASC;APC;SYT10;TEK;CCDC60;UNC79
5	Endometrial_cancer	131	TRIO;PRDM9;ZNF292;PCDH11X;TNC;ABCA12;RXFP1;ABCA13;PREX2;CDH4;SCN11A;SACS;EP300;POTEE;SVEP1;POTEF;HMCN1;EYS;CUBN;DSCAM;MTUS2;ANK2;THOC2;ANK3;DICER1;PCDHA12;TIAM1;FNDC1;HECW2;UTRN;MXRA5;FREM2;TRANK1;PCDH15;CACNA1D;CACNA1C;PCDH19;KALRN;CACNA1E;PKHD1;SPTA1;GRIN2A;CPAMD8;PLXNA2;PCDHA3;PCDHA9;PCDHA8;MDN1;TRPM3;DNAH10;MUC16;ABCA9;PRRC2C;NEB;LRP1B;DCLK1;HRNR;PTPRD;SDK1;FAM135B;DLG2;DMXL2;NF1;TPP2;FAT3;LZTR1;EIF4G3;CNTNAP2;RYR2;MYOM1;DOCK3;TENM3;SYCP2;TENM4;CHD7;FAM208B;FMN2;SIPA1L2;RYR3;SYNE1;SIPA1L1;GRM8;POLE;GRID2;TRPC5;KCND2;DCC;NBPF10;SORCS1;ARID1B;MYH1;LRRC7;CDH10;BIRC6;PKHD1L1;CNTNAP5;DNAH3;DNAH2;LAMA2;SEMA3D;DNAH8;COL11A1;NRXN1;DNAH5;PKD1L1;TPTE;AKAP6;DNAH9;LRP2;ADCY8;RELN;CUX1;NSD1;DMD;TRIM48;FLNC;ASCC3;CSMD3;PCNT;CSMD2;CSMD1;GPR158;ATF7IP;DYNC1H1;LAMB4;PDE4DIP;MTOR;SCAF4;FRAS1;APC;KIF26B
6	Pancreatic_cancer	73	RYR2;TENM2;TENM3;THSD7B;CHD7;LDLRAD4;ABCA12;BICD1;BACH1;RYR3;GLI3;PARK2;AFF2;SYNE1;DOCK10;PREX2;DEC1;RIMS1;PRB2;SACS;TNR;HMCN1;NCKAP5;FAM19A5;SOX5;MAP2K4;RNF43;KLF12;DSCAM;GRID1;ATRX;MTUS2;ANK2;ABCC9;SLCO1C1;NR5A2;OBSCN;PXDN;TFF1;COL6A5;DOCK2;DSCAML1;DNAH5;DNAH6;PCDH15;NRXN3;ATP10A;DNAH9;CACNA1C;ADCY8;SPTA1;DPP6;LOXHD1;PCDHA1;PCDHA5;DMD;FLNC;SLIT2;CSMD3;CSMD2;CSMD1;NDFIP2;PCDH9;MUC16;NEB;LRP1B;SMARCA4;PTPRD;MYO1D;APC;ZNF536;FAT3;MDGA2
7	Breast_cancer	173	FHOD3;PRDM9;THSD7B;CXORF22;COL12A1;PCDH11X;TNC;CELSR1;ABCA13;CDH9;PALM2-AKAP2;PREX2;RIMS2;DMBT1;CHEK2;SCN9A;ZNF208;KDR;EP300;ZNF569;SVEP1;HMCN1;ROS1;POTEC;EPHA5;CUBN;PDGFRA;IL4R;MTUS2;ANK2;ANK3;HSPG2;OBSCN;ADCY9;ADAM12;HECW1;ELMO1;WDFY3;UTRN;MXRA5;EPHA3;DGKH;FTO;NXPH2;CTTNBP2;STXBP4;TSHZ2;ABCB5;CACNA1B;PCDH15;CACNA1D;CACNA1E;FHIT;PKHD1;ADAMTS16;SPTA1;TRPM2;GRIN2A;PDZD2;CPAMD8;PLXNA2;HIVEP3;PCDHA3;PLXNA4;MDN1;IMPG1;TMEM132D;MUC16;CNTN6;BCL11A;NEB;ESR1;LRP1B;DCLK1;HRNR;SMARCA4;PTPRD;SDK1;STAG2;DIAPH3;FUBP1;CCDC88C;NF1;FAT3;LZTR1;ATR;DPP10;ROBO2;SPAG17;CNTNAP2;RYR2;SETD2;DOCK4;FLT1;DDX3X;TENM3;MAST4;TENM4;CHD6;FAM208B;SYNE2;RYR3;AFF2;SYNE1;GRM7;CHL1;BAI3;MYO18B;HYDIN;PGPEP1;CHST9;PDE4D;ATRX;NBPF10;VPS13B;C10ORF11;ABCC9;RAD51B;TOX3;MYH1;NCOR1;LRRC7;CDH10;MYO3A;MYH8;BIRC6;PKHD1L1;MYH4;SOS1;DSG4;CDH18;RBMS3;DNAH3;NBAS;DNAH2;CD163L1;LAMA2;DNAH8;DNAH5;ASB13;PKD1L1;AKAP6;DNAH9;LRP2;RELN;TUBA3C;NHS;DMD;GPC5;CSMD3;PCNT;CSMD1;ATF7IP;A

			TE1;DYNC1H1;TCF7L2;ANKRD30A;FAM46A;CADPS;LAMB4;PDE4DIP;CYP4B1;PTPN11;MTOR;STXBP5L;GALC;NLRP12;FRAS1;APC;CCDC170;TEK;LGR6;HCN1
8	Lung_cancer	157	ZNF451;ATP8A2;THSD7B;ATP8A1;ZNF292;FRMPD2;CBLB;IL1RAP;CDH9;CDH8;ETS2;CDH2;SCN11A;KDR;EP300;POTEF;KIFAP3;ARID2;ANKS1A;CMKLR1;KCNH1;PPFIA2;POTEC;EPHA5;PDGFRA;MEF2C;KCNH5;DSCAM;PRKCB;THEMIS;TNNI3K;THOC2;GABRG3;PCDHA10;KCTD8;CLIP1;ZNF91;OR4K15;KRTAP4-11;HECW1;MAGEB2;ZNF559;OR4C12;PKP4;AMPH;PRKD1;DGKI;EPHA3;GRIA1;CHRNA3;RTN1;PCDH15;ITPR2;PCDH17;PKHD1;SLCO1B1;GRIN2A;KIAA1009;RNF213;STK36;ZBBX;VTI1A;PCDHA3;PCDHA2;LRRC4C;PCDHA8;IMPG1;GRIA4;BPTF;ATP8B4;STARD13;CD163;TFAP2D;MUC16;ESRRG;PXDNL;FRG1B;LRP1B;ANKHD1;SMARCA4;PTPRD;CCDC88A;FAM135B;ADCK1;ZFP64;NF1;NES;MIPEP;FLT1;ITGAM;DOCK3;NUMA1;FLT3;PCDHGB2;C1ORF173;SIPA1L2;SLC6A2;TRIOBP;SYNE1;GRM7;LRRTM3;BAI3;GRM8;ZNF527;PHACTR3;HYDIN;TAS2R1;JAK2;NINL;PCDHGA8;PCDHGA2;FCRL3;ATRX;NBPF10;SLCO1B7;DUSP27;CNKSR2;VCAN;NRG3;SETBP1;CDH10;BIRC6;PIK3C3;SOS1;LPHN3;RBMS3;CRB1;SAMD9;COL11A1;AKAP6;ASB17;FYB;STAMBPL1;PAK7;GPC3;ZNF229;DMD;GPC5;CSMD3;ATF7IP;NTRK2;OR8J3;NLGN4X;ATRNL1;NTRK3;COL22A1;KCNIP4;ATAD2;OR5L2;PLEKHA5;SCAF4;NELL2;REST;APC;CCDC170;GOPC;HCN1

Table S7. Overlap of rDNA-contacting genes associated with three Jensen Diseases. Related to Venn Diagram in Figure 2D.

Names	total	elements
endometrial cancer melanoma skin cancer	46	*TRPM3 TPTE HECW2 CACNA1D DNAH8 LAMA2 TNC ANK2 PREX2 DNAH3 PTPRD CSMD3 GRM8 DMD LRP1B KALRN BIRC6 NF1 ANK3 AKAP6 PCDH15 CSMD2 DCC MXRA5 COL11A1 CDH10 APC ADCY8 RELN GRIN2A PKD1L1 HMCN1 DNAH9 ABCA12 GRID2 LAMB4 LRP2 SYNE1 FREM2 CNTNAP2 PKHD1 DNAH5 FMN2 MYH1 SCN11A DNAH2*
melanoma skin cancer	142	*NLRP13 DSG4 COL4A6 CATSPERB KCNB2 TACC2 SEZ6L VCAN FSTL5 GRM7 ADAMTS18 CD163 KDR COL22A1 KIAA1217 FAM13C SPAG17 ZNF385D CGNL1 KCNT2 FLT1 CDH18 MYH8 CHL1 C8A SRGAP3 PTPRS ATP8B4 UNC79 PDE1C DOCK2 BAI3 DSC3 PTK2B ASTN2 SLCO1B3 ANO4 CYLC2 PCDHA5 PCDHA1 MED13L ATXN1 SORL1 CADPS NFASC PLCH1 KIAA2022 FAM83B SLIT3 RGS7 GRIN2B PLCB1 PAK7 TEK STAC C1ORF173 GRIA1 A1CF KCNH7 MOV10L1 CCDC60 DGKI CDH9 MYH4 ASTN1 SYT10 ADAM28 PCDHA13 CHRM3 MAGI2 FLNB NLRP2 NELL1 ALK TMEM132B MSR1 NLRP12 PCDHAC2 CDH6 ABCB5 SORCS3 LRRC4C CADM2 STK31 PLCL2 SLIT2 CACNG3 COL2A1 LOXHD1 ZNF804A PLEKHH2 CD163L1 ARHGAP29 NDST4 RASGRF2 NRG3 VPS13A SLC22A25 TUBA3C GRIK2 ADAMTSL3 DPYD PCDHA7 LRRK2 EPHB2 LPHN2 HYDIN PHLDB2 KHDRBS2 TPO MAGI1 CLSTN2 ARID2 TRIOBP CTNNA3 TNR VPS13D AOX1 C1ORF168 TBX15 CRB1 PAPPA NEBL SPATA16 CDH8 KCNQ5 GALNT14 TCF4 THEMIS ADAMTS6 CFTR NPSR1 C6 ROS1 SNX31 EFCAB6 VPS13B CD1C KCNH5 GRIN3A ISX ABCC9*
endometrial cancer skin cancer	5	*CUBN PCDHA8 DMXL2 FAM135B LRRC7*
endometrial cancer melanoma	5	*PCDHA9 SIPA1L1 NBPF10 SDK1 DNAH10*
skin cancer	19	*COL9A1 PCDHA10 EPHA3 KIAA1199 PDGFRA KCTD16 NAV2 ATRNL1 CNTN4 FLT3 COL15A1 TMEM132D RNF17 KL ZNF536 ATP8A1 CHD6 CORIN PAH*
melanoma	31	*LIPI LMO7 PCDHGA2 CTNND2 EDIL3 SPOCK3 BPIFB1 HTR4 SAMD3 NRXN3 ACSM3 GPC5 SPAG16 GRIK1 PCSK5 FTO GHR TRPM2 ARNT WBSCR17 RBFOX1 TNS1 RAG2 MTAP MCTP2 PPP4R4 ADAM2 CRISP3 ACSM2B SLC5A8 TSHZ2*
endometrial cancer	75	*PDE4DIP RYR3 KIF26B MTOR EYS MTUS2 CACNA1C CNTNAP5 SORCS1 FRAS1 GPR158 LZTR1 EP300 TRIO SVEP1 NSD1 DLG2 MDN1 SACS PLXNA2 TPP2 MYOM1 ASCC3 TRPC5 TRIM48 EIF4G3 UTRN CHD7 TENM4 PRDM9 DSCAM CUX1 POTEF CACNA1E DICER1 KCND2 THOC2 PCDH19 MUC16 PRRC2C FNDC1 DOCK3 TENM3 PCDHA12 PCDH11X TRANK1 CSMD1 DCLK1 NRXN1 RXFP1 ARID1B POTEE CDH4 SCAF4 DYNC1H1 SPTA1 FLNC SIPA1L2 ZNF292 RYR2 SEMA3D PKHD1L1 NEB ATF7IP FAM208B FAT3 CPAMD8 TIAM1 PCNT PCDHA3 ABCA13 POLE ABCA9 HRNR SYCP2*

Table S8. Overlap of rDNA-contacting genes associated with three Jensen Diseases. Related to Venn Diagram in Figure 2E.

Names	total	elements
breast cancer liver cancer lung cancer	20	*ATRX GRM7 KDR BAI3 CSMD3 DMD LRP1B BIRC6 CDH9 NF1 MUC16 PCDH15 SMARCA4 APC HYDIN EPHA5 SYNE1 PKHD1 HECW1 THSD7B*
breast cancer lung cancer	18	*IMPG1 CCDC170 SOS1 RBMS3 EP300 EPHA3 FLT1 HCN1 PTPRD PDGFRA GPC5 POTEC AKAP6 NBPF10 CDH10 GRIN2A ATF7IP PCDHA3*
breast cancer liver cancer	60	*PDE4DIP RYR3 NBAS DIAPH3 CUBN DNAH8 LAMA2 FRAS1 ROBO2 SETD2 SVEP1 CDH18 SYNE2 MDN1 ANK2 AFF2 PREX2 DNAH3 STXBP5L RIMS2 UTRN TENM4 WDFY3 ANK3 CACNA1E HIVEP3 SDK1 ABCB5 OBSCN TMEM132D MXRA5 TENM3 CSMD1 DOCK4 RELN MAST4 PKD1L1 CHD6 HMCN1 DNAH9 CELSR1 MYO18B LRP2 SPTA1 DYNC1H1 HSPG2 CNTNAP2 RYR2 PKHD1L1 DNAH5 PDZD2 CNTN6 NEB COL12A1 FAT3 FHOD3 VPS13B ABCA13 DNAH2 PLXNA4*
liver cancer lung cancer	28	*VCAN AMPH PCDHGA2 COL22A1 NTRK2 PRKCB GRM8 BPTF PAK7 GRIA1 DSCAM NES PKP4 NUMA1 ANKHD1 GRIA4 COL11A1 ZNF91 PLEKHA5 PRKD1 NRG3 SCAF4 ARID2 CDH8 LPHN3 PCDHA2 KCNH5 RNF213*
breast cancer	75	*DSG4 MTOR MTUS2 CCDC88C ADAM12 CACNA1D NHS IL4R LZTR1 SPAG17 MYH8 CHL1 LGR6 C10ORF11 TNC RAD51B ZNF208 PLXNA2 TCF7L2 CADPS FHIT MYO3A STAG2 ANKRD30A TEK CXORF22 ASB13 PRDM9 PTPN11 FTO DGKH MYH4 GALC CHST9 DMBT1 TRPM2 BCL11A ESR1 NLRP12 FAM46A PDE4D ATE1 PCDH11X TOX3 CD163L1 DCLK1 ADAMTS16 TUBA3C ATR NCOR1 DDX3X ELMO1 LAMB4 LRRC7 DPP10 ADCY9 CACNA1B CHEK2 PGPEP1 ZNF569 MYH1 SCN9A TSHZ2 CYP4B1 FAM208B PALM2-AKAP2 NXPH2 ROS1 FUBP1 STXBP4 CPAMD8 CTTNBP2 PCNT HRNR ABCC9*
lung cancer	91	*ZFP64 ANKS1A ITGAM ZNF559 SLC6A2 SLCO1B7 CD163 CBLB PCDHA10 RTN1 NLGN4X VTI1A ADCK1 ATP8B4 NINL KCTD8 KIAA1009 CHRNA3 OR4K15 STK36 GPC3 JAK2 PCDHA8 PHACTR3 TFAP2D ATAD2 STAMBPL1 ZNF229 C1ORF173 FRG1B DGKI POTEF OR8J3 PPFIA2 ATRNL1 OR5L2 CLIP1 KRTAP4-11 THOC2 FLT3 ATP8A2 ZNF451 TAS2R1 OR4C12 KIFAP3 GABRG3 SAMD9 SLCO1B1 PXDNL PCDHGB2 CMKLR1 REST DOCK3 STARD13 ASB17 IL1RAP LRRC4C PIK3C3 SETBP1 FYB ATP8A1 ITPR2 GOPC FRMPD2 NTRK3 FAM135B NELL2 DUSP27 ETS2 TNNI3K FCRL3 CDH2 LRRTM3 SIPA1L2 TRIOBP PCDHGA8 ZNF292 CRB1 CNKSR2 ZBBX ZNF527 THEMIS KCNIP4 ESRRG SCN11A MAGEB2 CCDC88A MEF2C PCDH17 MIPEP KCNH1*
liver cancer	157	*PLCE1 EYS TPTE FMN1 CACNA1C MGAM PRTG FSTL5 PEAK1 CNTNAP5 SORCS1 ZFPM2 CTNND2 DNAH14 NAV3 GPR158 KIAA1217 TTC40 SNTG2 ASXL3 TRIO APBA2 PTPRS ADGB COBL SLC39A10 KIAA1211 UNC79 DOCK2 SPECC1L SACS ANO4 PCDHA5 MED13L TANC2 FRY MDGA2 KIAA2022 FAM193A C12ORF55 TLN2 FAM83B UNC13C LRFN5 ZNF717 GABRB2 EDNRB MYO9A NRXN3 TM9SF4 NLRP7 WDR33 PLCB1 ADCY2 ROBO1 STARD9 FAM65B GRIK1 CUL5 CECR2 KALRN DOPEY2 DMXL2 CUX1 PRR12 NBEA ABCC1 EFCAB5 DICER1 PCDHA13 UNC5C RUNX1T1 LRFN2 MAGI2 FLNB MYO6 SMCHD1 EVC2 PRRC2C CSMD2 PLCL1 PCDHAC2 MCM9 COL24A1 UBR3 SLC44A5 WDR52 FREM1 ALMS1 FAM155A DNAH6 DCC BAZ2B PCDHGA7 CNTN1 PCDH7 RBL2 MMP16 ERC2 STK31 CADM2 DENND1A ZNF536 EPHA6 PXDN PRUNE2 TRANK1 PDZRN3 TF CDH11 ADCY8 SLC9C1 CDC42BPA LRRTM1 GLI3 CNOT1 MME NIPBL IGF1R SND1 VPS13A NRXN1 MCTP2 PARD3 COL6A5 AGBL1 DEPDC5 PCDHA7 THBS2 JPH3 ADAMTSL1 TNR VPS13D TENM2 FREM2 VAV3 PAPPA EHBP1 MACC1 GRIK4 MYH13 FMN2 ARHGAP5 CACNA1I KIAA1731 ZNF366 FLRT2 DNAH10 EFCAB11 GRID1 ATP10A TNRC6B CACNA2D3 MCTP1 SPEF2 SYCP2 FSTL4*

Table S9. Changes in contact frequencies of different rDNA-contacting genes with rDNA clusters after heat shock treatment. Excel file attached separately.

Table S10. Overlap of the selected 4020 rDNA-contacting genes with genes that changed their contacts with rDNA after heat shock treatment. Related to Venn Diagram in Figure 4B.

Names	total	elements
4920 - 4C decreased	553	*RNU7-66P CMTR2 GLT1D1 LDB3 TMC4 EPB41L4B KLHL14 RNF185 POTED CCDC34 KIAA1210 ZNF257 GPR78 ANGPT4 VAT1L ABCD1P3 CHST15 SAMSN1 SPANXN4 OR7E25P TF FGF7P2 OR10J6P AKR1B15 COX10 CENPU TMEM11 GNB4 BDH2P1 ACTR5 HK3 LCA5L FAM225B HS1BP3 TRAV8-7 PLSCR4 STRIP1 MSNP1 RNF182 GPX1P2 VRK2 NDRG2 ZNF962P ZNF384 ZMYM5 OR11L1 RCAN1 FAM193A EDDM3B SIRPG RAB5A MIR17HG JRK SNX18P9 ANKRD20A5P IL20RA STRN3 CMKLR1 LINC00575 KRT74 SPINT4 ITGA2 RNU1-142P MBL1P RNU4ATAC8P IGHVIII-2-1 NIPA2 GBP4 MAPK1IP1L PQLC3 MKKS CR1 IGHVIII-26-1 HFM1 STK24 NUTF2 TPT1P2 EIF4E BPIFB1 LINC00320 ERCC4 FAM83B OR4K15 NCOR1P1 ENTHD1 C5orf51 MOV10L1 NLK NIPA2P2 IPPK OR5L2 TMPRSS3 RNF152 MEOX2 KCNG3 LINC00365 OR4S2 TMEM52B LINC01094 DUSP27 OR8K4P NANOGNBP2 ABCD1P4 CHEK2 SIGLEC30P RAB22A DDX21 ANKRD20A9P ZNF688 PWP1 ILDR1 GRXCR1 STXBP4 MED15 ZBED4 MT1HL1 ZER1 OR52X1P POTEM FAM182A DET1 LINC01020 ACTBP8 POTEB2 ZMYM2 TAB3 ERMN TRIM48 ADCY2 NOVA1 SH3BGR TRDV1 RPL21P41 ABCC13 IGHV1OR15-9 WRB POTEB CDC20B LINC00347 SYNM GLP2R MAP2K3 IGKV1-5 ANKRD34C KMT2C MT2P1 DIO3OS IFNWP4 IGHV3-76 UGCG ORC3 VWFP1 SLC9B1P3 CHST8 LINC00839 HSPB8 PXT1 RLBP1 KCNE1 TRMT61B ITLN1 ZSCAN5A TSPAN3 TPP2 URB1 BMPR1A RPL23AP12 ABCG1 ABHD4 FAM207CP AKIRIN2 SOD3 MPPED1 PWRN2*

		CYCSP51 ETS2 FCRL3 CTBP2P4 OVGP1 HIRA TMPRSS6 SERPINA1 RBM19 FEM1AP1 CDR2 PSMC6 ITGB7 ZSCAN10 NLRP13 MANSC4 MAPK14 DYNLL2 PRR14L ROCK1P1 PRLH CST9L CYCSP17 DMBT1P1 PWRN1 WDR4 RPS4XP22 EREG ANKRD36BP2 DUX4L9 CBX3P4 GRAMD4 PPIAP1 LINC00158 FRMPD2 NPM1P31 BCL2L13 OSGIN2 CRISP3 GABPA KIF3B ZNF429 CTBP2P5 WDR47 KRTAP13-6P OR4A5 ELOVL2-AS1 ACTR3BP2 ZDHHC11 MOV10 RPL39P40 ZNF337-AS1 MS4A1 IGHV3-16 TMEM51 LINC00674 C7orf66 BOLL DCUN1D4 LINC00544 SLC12A1 KRTAP8-3P SMC1B CA10 SLC4A1AP IGHV7-34-1 PGAP3 HELLS SHISA9 ZNF355P SNRPN OR4K17 SNHG17 LINC00458 SRD5A3-AS1 S100A7L2 OR4C15 ARHGAP44 C2CD2 IGKV2OR2-2 SLC30A5 C22orf34 RANBP10 LINC01053 TDRP CIZ1 ZNF98 RNVU1-18 PLCD3 WSB2 ADIPOR2 PABPC1 RN7SL766P EFCAB6-AS1 RNU1-131P TBC1D2 SLFN5 MORC2 LINC00702 RPL39P33 TTLL8 ACTR3BP3 MFSD9 FBXO32 SLC18B1 CLIC6 GTF2IP2 IGF2BP1 RANGAP1 NF1P6 KRTAP4-12 CYP39A1 CHCHD2P4 ARL2BPP8 BTN2A1 LINC00520 G2E3 CCDC88B MAGEB3 CXADR POLR3K CECR2 C10orf120 SERPING1 ASIP CDH10 KL OR10T1P ZNF331 POTEG PWRN3 KRTAP29-1 MYH13 MMP27 AGGF1P3 GANC ZNF583 LINC01058 OR4C9P LINC00113 PHYKPL HPYR1 LINC00960 PSMC4 LINC00408 EPHA7 OR4A9P KANK3 LINC01070 AGGF1P2 ARR3 GAS8 LCE2B TRPM2 LINC00507 S100B GABRG3 CILP2 TNFRSF19 NAP1L6 MXRA5 RNA5SP519 RPS3AP41 ZNF965P OR4C14P HUNK RPS23P5 ISCA1P3 REEP1 CHMP4C BCL2A1 DTX4 AJAP1 SNX19P2 USP40 GSG1L NCOA5 RNA5SP478 RN7SL568P SOGA1 PAGE1 STARP1 KLHDC7A NF1P4 GADD45A RCHY1 NF1P1 FAM201B TMED8 PRIMA1 POLR2F KRTAP5-8 ATP1A4 C1QTNF6 DUX4L19 SH2D7 CYTIP DPP9 GLDC SUZ12 BLZF2P ZNF729 TSPY5P PJA1 TTC22 OR5D15P OR4C2P ISX RAD51AP1 SEC14L6 CYB5R2 ANKRD20A11P IL4R TM4SF1 PNLIPRP3 ACTR3BP5 RMRPP5 WBP11P1 KRT25 GNG5P5 SPRR2G TMEM211 MARK2P9 ATP8B5P ZNF91 PATE4 PMP22 FAM111B PLEKHA5 LINC00317 TFF1 PLEKHA6 OR6K4P C14orf177 COMT SNX19P1 CD1C DUX4L16 RNU1-51P LINC00884 KLRF2 CYCSP6 ZBTB34 ZNF826P MAGEC3 FPR3 DSCR8 CYLC2 BTNL9 BASP1 GRK6 CRLS1 SQSTM1 DEFB116 KRT8P25 SLC16A12 CSF1 RNA5SP495 ANKRD30BP1 GPATCH2L CECR7 ARID5B MYBL2 ZNF527 BRF1 ACTR3BP6 WT1 OR4C5 KSR1 CFL1P6 TUFT1 FBLN1 MTFMT RAF1 GRPEL2P1 PSMB2 SNRPD1 DIO2-AS1 KBTBD11 HBD REV1 GALC OR4C12 DIRAS2 ABCB5 OR4Q2 NEK2P2 HNRNPA1P58 MANEAL PPIAP27 NSUN3 GOPC POLR3C LGALS14 CPPED1 ABHD17C ZNF292 TBX15 C21orf91-OT1 ZNF521 SMYD2 TMPRSS2 DUXAP10 ACSM5 CA5AP1 ZNF610 KDR BRD9 OR2M5 OGT SYNDIG1 DPF3 TMEM45B DUX4L17 AGBL3 SCARA5 LINC00923 AGGF1P1 LINC01146 RASGRP1 IGHV3-65 ZNF451 RN7SKP126 GNPTAB ABCC10 RARRES2P1 TMEM173 SOX8 BCL2L1 GBP6 OR5J2 IGLV3-19 MIR155HG IGHV3-43 HSH2D SLX4IP YME1L1P1 ZNF569 TMEM161A LINC00707 FAM189A2 RNU6-576P MAT1A KCNC4 GNB5 IGHV3-60 BACH1 POTEH EWSR1 PSG6 FAM221A RNU4-45P LINC00378 SART3 FDXR RAB27A HLA-DQB2 IGKV2OR22-3 TUBGCP6 CST2 OR4N2 NUMA1 ZNF330 RN7SL659P ATE1 CEP44 MYO5BP2 OR8L1P ETV6 RNU6-458P KCNJ15 ATIC FANK1 AMD1 TUBB1 ARMCX2 USP3 OSBPL5 RXFP2
4920 - 4C increased	325	*LRRC37A5P C10orf90 BBOX1 KLHL13 RTN1 BANK1 WSCD1 NFIA SYN3 PRKCB SLC8A1 OPHN1 MAP3K13 KIAA1324 COL18A1 RSRC1 THRB SLC9A9 RN7SL52P IGSF11 CDK17 DPYD QKI KCNQ5 PLCXD2 ZBTB20 HEPHL1 DLEC1 SORBS2 C1orf112 ETFA FSTL5 AHRR APBA2 ASTN2 CSMD3 PECR NEGR1 KIAA0753 WIF1 PPP6R2 SETBP1 ZBTB7C IL1RAPL1 WDPCP RERGL CD109 GRID1 CNBD1 CCSER2 LMO7 FOXP2 EXOC4 HEATR5A BBS9 CDC27P2 NTRK2 CCDC3 WDFY3 TDRD3 OVCH1-AS1 LINC00273 ZNRF3 KRTAP13-5P TFP1 BTBD11 WDR27 STPG2 MTPN PHEX SYBU PDE4DIP RGS12 UBE2E2 FTCDNL1 SH2D4B TANGO6 DLG5 TMEM135 EIF3E SH3GL2 MYLK3 ZNF734P CCDC178 SLC4A4 CRB1 COL12A1 ABLIM1 AFF3 CHRDL1 RAP1A TEX36 ST6GALNAC5 DIAPH2 DIP2C DGKH SLC16A7 TBC1D5 CNTN4 TNFRSF11B HMCN1 CDC27P1 EPHA5 SGIP1 NPSR1-AS1 ALDH1A2 MAPK9 ESRRG TRAT1 PDE9A KIAA1217 VANGL2 SLC1A2 STXBP5-AS1 LNP1 MAML3 GRIN2B ROBO1 PRRC2C KCNK1 FCHSD2 WDR64 ADCY8 ELP3 CCDC30 SHC3 MGMT SKA1 PRICKLE2 CSRNP3 CACNA2D3 SPESP1 CACNA1D IL1RAPL2 SPAG17 CHCHD3 EDIL3 KLF7 AHSA1 GDAP2 MND1 SDK1 GRM5 PAMR1 RPL31P40 DIAPH1 IGF1R JAM2 CNTNAP2 CBLN4 KCNIP4 ZFP64 KIF13B CD96 TMOD1 NHSL1 GAS7 CLEC16A ITGA9 PCSK5 CTPS2 KCNMB2 GTF3C6 DOCK3 LARS2 GPC6 MAMDC2 SUSD1 LINC00470 RAB7A RNLS SPOCK1 HPSE2 TACC2 ITPK1 PAK1 MITF STK32C ADCK1 UTRN CDC14A GPC5 ENOX2 CASC16 DNAJC5 ULK4 DLX6-AS1 BACE2 SLC25A21 CLSTN2 PLD1 RARB FAM126B PHACTR1 GUSBP1 USP17L24 VTI1A DISC1FP1 FHIT BARD1 FAM19A1 TRABD2B C1QTNF7 GALNT2 RNF150 DPY19L3 PDE4D PCDH11X RUFY1 EGFEM1P NUF2 SAMD5 SSBP2 RPH3A GREB1L EMCN GPC4 ADGB VSTM2B KLHL1 KIDINS220 PLCB4 BAZ2B MGAT4C ARFIP1 RBBP8 TTI1 NSUN6 GRID2 TTC29 ZNF415 PDE4B MAPRE2 PHF8 SGCD SHROOM4 NAV3 IMMP2L SLC35F2 NSUN7 IGF2R TTLL5 MARCH11 AUTS2 ESR2 ADARB2 COX10-AS1 MCTP1 PANX1 CCDC59 FRMD3 LGR6 GRM8 PHF3 CACHD1 NUP62CL ARNT CCDC26 CADM2 DCLK1 SERPINB7 EVI5 SYCP1 DYM RNF219-AS1 ANO1 CADPS RGS7 TNMD SULT1C2P1 GNG4 ACO1 WDFY4 STK33 UBXN2B COL25A1 VPS13A HYDIN TXNDC16 CTNNA3 SPATS2 TRAV8-4 CREB5 B3GAT2 LAMP5 DACH2 ZC3H13 KCNK2 SORCS1 DNM3 PPP1R12B PLXNA2 ZDHHC15 PLCB1 CDH9 NDUFAF2 MGA MAGI2 NLRP2 PLCL1 GALNTL6 CCDC144CP MBD3L1 SLC30A10 NECAB1 SMOC1 RYR2 KCNH5 EYS IL16 ROBO2 NCALD DYRK1A PCP4 PDSS2 CCDC92 SLC2A9 CASK LCE4A MARCH1 SEC22A LMBR1 ZPBP KIAA0825 VPS13B*
4C decreased	391	*AC137499.1 AL589182.1 HS3ST3A1 AC008667.3 RF00426 LINC01915 LINC01701 LINC01441 AC138701.1 AL031674.1 AC011193.1 LINC01789 AL021937.3 CDC14C LINC01502 ZNF37CP AC084373.1 AF064860.2 AC091826.2 AL031289.1 LINC01432 AL133173.1 AC006206.2 ZNF840P LINC01194 LINC01907 FRG1-DT AC092423.1 AC124944.2 AL132708.1 AC068446.2 AP001605.1 AC119751.4 LINC02311 NEURL1-AS1 AC140658.7 AC026434.2 AC009271.1 AC021439.1 AL121999.1 TMEM231P1 AC106871.1 AF064858.1 ZFAND1 TUNAR LINC02305 AC079062.1 AP000534.2 BNIP3P6 AP003351.1 AC025884.1 LINC01495 AL139348.1 AC097493.3 AC073325.1 AC020595.1 AP000233.2 AC008608.1 AC009567.1 LINC01684 AC118282.3 AF121897.1 AC005185.1 AL158090.1 AL358292.1 INTS13 LINC01648 AC087883.1 LINC01687 LRRC1 CR383658.1 SDR16C6P ARHGAP27P1-BPTFP1-KPNA2P3 AL589743.2 LINC02322 AC069545.1 AC145543.1 RF00156 AC097532.1 Z82249.1 AC013460.1 AC009169.1 MRPL58 FRG1BP IATPR AC098826.1 AL137009.1 AC097532.2 AC023078.2 AP000470.1 AL773545.1 AL020994.2 AC126603.1 AC245028.2 AC140847.2 AC005863.1 AL355516.1 AL035250.1 LINC00308 FP325330.1 AL049875.1 AC007333.1 AC006273.1 AC068760.1 AL158042.1 OR7E104P AL359081.1 LINC01262 LINC01203 LINC01603 GRK3 AF241725.1 AC090136.2 LINC01809 AL157756.1 AC105148.1 CR383656.10 AL110503.1 AC119751.1 D21S2088E AC023824.4 AC009139.2 LINC02200 AP000542.1 LINC01938 AC245748.2 AL137792.1 AL392023.2 DUX4L50 MS4A15 CSE1L ZNF630 AC117569.1 AL022324.3 PTPA MIR193BHG AL161636.1 BNIP3P41 RPL21P89 AC174048.1 CR383656.4 LINC01692 TPTE2P4 AC007333.2 AL157359.2 CARMIL3 AC018618.1 AL139246.3 AC010880.1 AC010374.2 PYHIN5P AL357500.1 AC022816.1 AC025277.1 WDR35 GUCY1B1 AC097374.1 AL445383.1 AC006455.1 PIP4P2 AL606495.2 AL117329.1 AC069061.2 AC116353.2 PRAMEF25 FO393415.1 AC111198.1 RPL23AP87 AL731574.1 AL160237.2 AC087463.1 AC010425.1 AL603840.1 AF165147.1 AC023934.1 AC023157.2 AC024651.2 AL132996.1 AC097480.1 LINC01426 Z82198.3 AC027612.2 AL136018.1 TESC RPL21P91 LINC01908 LINC02126 AC092115.3 AL031601.1 AP005901.1 SP140L AC004656.1 LINC01491 LRRC37A4P LINC02125 AC133065.1 AL356800.1 GTF2IP4 LINC01410 AP003108.2 PKIA-AS1 LINC02499 AC010615.4 AC024475.4 AP000547.1 AL049812.2 LINC02250 AP001341.1 RF00421 MIR548XHG LINC02284 AP005212.1 AL136968.2 LINC02309 AC044784.2 AC012322.1 AP006748.1 AC026741.1 AC089987.1 AL355607.2 AC037471.2 AC016930.1 AL162632.2 AL356095.1*

		AL137845.2 AP000705.1 BMS1 AL121718.1 AL352984.1 AC016737.1 AC062021.1 AP001574.1 AC068643.1 LINC02409 AC099654.1 AC005580.1 AC093074.1 AC092681.1 AC114501.2 AL669831.5 AL109618.1 AC018730.2 AP000547.2 AL356276.2 CR383656.6 AC063952.1 MYEOV LINC02277 AC130464.1 CEMIP AK6P2 LINC01268 AC099793.1 AL139393.1 AC008794.1 AC091193.1 AL049651.1 AC092966.1 AL022332.1 AC007848.1 AC027612.4 AC060788.1 AC016044.1 AL365232.1 LCP1 LINC01227 AL121821.1 LINC02089 LINC02101 AL663058.1 LINC02347 LINC02082 HACD4 LINC01683 CIB4 AC068722.1 AC025857.1 LINC01644 AC018904.1 AL353633.1 AC009081.1 RF01182 AC106785.2 AL512310.1 RGMB AP000265.1 AL117190.1 AC090618.1 AC015771.1 AC009652.1 AL049775.2 RPL12P12 AC097501.2 LINC01920 AC108047.1 AL353148.1 CFAP46 SOCS6 LINC00929 AC005476.2 AL121852.1 AC092175.1 FO393418.1 AC087341.1 PAXIP1-AS2 AC005307.1 RPL26P28 AC016573.1 RF00012 AL772155.1 LINC01229 AC026495.1 AF212831.1 AC138123.1 AC006548.2 AP001116.1 AC142384.1 EDN3 AP000235.1 CR786580.1 LINC02485 AC132825.3 RPL12P27 AL607077.1 LINC01706 LINC01734 ATP5F1A AC091198.1 HRASLS5 AC091730.1 C1orf185 AL590640.1 LINC02312 BHLHB9 AC087516.2 LINC01425 AL109935.1 INTS4P1 AC008164.1 AL121821.2 PPIAP8 AP003973.2 AC119751.5 AC027458.1 AC118282.2 AC011507.1 LINC01310 AC122710.1 LINC02290 AC122134.1 AC006987.2 AC026787.1 AL161722.3 GPR137C AC116553.1 AC022695.2 AL592494.1 AL021877.1 AL627422.1 AL590623.1 LINC01533 AP001803.2 AP000472.1 AC011503.1 AC244502.1 MIR4290HG FAM242A AC090993.1 AC002511.1 AL110505.1 AL359955.1 AC020914.3 AC090525.1 LINC01689 AC008992.1 FAM227A SNRPCP1 AC018767.1 AL355493.2 AC073314.1 AC009299.1 LINC01224 AL033381.1 AC012414.5 AP001979.2 AC100770.1 AP000959.1 LINC02307 AL360270.3 AC073133.2 AC073530.1
4C increased	1770	*AL590652.1 ELMO2 FARP1 AC084116.3 LINC01201 XDH AEN PTPRR ACOT12 SLC10A7 PXYLP1 ANGPTL5 CNGB1 RNVU1-14 COL4A5 AC010280.2 ZEB1 MMS22L AP003469.4 OOSP4B TMEM132E NAT10 RN7SL35P TMEM184A LHFPL2 NMNAT1 LINC01655 C9orf43 CYP2F1 CNBD2 AL391358.1 GRPEL1 USP6NL LRGUK TERF2IP CD160 PDCD5 LINC02487 CEP350 CIDECP ARHGEF9 AL360175.1 EXOC3 EEF1DP6 LINC02438 MIR513A2 PRY PPIAP33 GCC2 AC087283.1 RARRES3 TMEM255A PREP HS2ST1 CD82 AC022201.1 GCLM SNX19 C12orf75 CHFR PIK3CB RNU6ATAC4P NRIR LINC02015 AC006238.1 ASS1 AC092957.1 AC112487.1 SRP72 PTPN5 ARSH PRR29-AS1 DDHD2 LINC01270 PPBP LINC01760 ITPRID1 USP32 KDM6A AP001528.1 MED27 PDP1 AHCYL2 AL137027.1 TPT1P14 AC011487.1 Z68871.1 CHRNB2 UBN2 GLG1 AC090809.1 L2HGDH AGPS TARID AC098487.1 NBPF3 CEP85 NOP58 C19orf67 NADSYN1 AC005261.1 LINC01243 CAMSAP1 RBM6 WAPL NFE2L2 TTC41P AC025263.1 SPINK5 KRCC1 TFEC PPM1H DCX GPBP1L1 RIC3 OTOA AL022324.1 EIF4E2 KLHL6 TTC13 CRABP1 LINC02107 CUL4B NEPNP ATP6AP1L DCTN1 CADPS2 LINC02077 FTH1P8 SLC2A10 TRIM45 PTCRA ARHGEF26 AC008080.1 CRAT37 DDI2 RF00393 FAM120A GPR135 LINC00348 AC100781.1 ARHGAP25 AC009262.1 AC092646.2 USH2A LINC01378 AC092078.2 UBE2J1 FAM157A AC083949.1 CLIP2 HNRNPA1P51 SLC35B1 GK5 SIGLEC1 ADD1 ZNF136 ZSCAN12 AC096773.1 ANP32A DNAJC1 TAC1 ASZ1 KIAA0232 FBXO3 AC093730.1 AC104170.1 LINC02117 AC079779.2 AP006296.1 HERC3 ZBTB47 OR1L3 PCNPP2 MORC1 MIR548A1 AL158825.1 EFCAB12 RAB9AP5 ARSA PIH1D3 GAS1RR AC022387.1 AC093459.1 AC090001.1 PIANP AL390729.1 NLRP9 AC007923.4 GAL GNGT1 MBD2 AMOTL2 LINC01111 PLA2G10 BLK FARS2 HRASLS LINC02180 RPP30 GTPBP2 CEACAMP11 EFR3B AVL9 AC142381.4 AC021074.3 ZFYVE9 FER1L6 LINC01790 TBC1D16 AC122138.1 SYN2 SEPT7 LINC02299 LINC01365 URB2 AL031687.1 AC005798.1 SYNPO2L CFAP70 CCDC162P IGHMBP2 TRAV19 AL139806.1 MSC-AS1 ZNF84 AC132938.1 LINC01554 STARD8 GRAMD1B NKAIN1 SIAH2 POLR3A ADGRE5 TCHP IMPG2 ZNF385C MMP3 SLC35F5 HIGD1C AC106772.1 XPO6 C6orf89 AC002091.1 ANKMY1 C9orf72 FAHD2B HACL1 MFGE8 AC092106.1 SH3BP2 TLR10 AC239859.1 WDR92 SAMHD1 RPS4XP3 TRIM4 SH3YL1 FMO3 LINC00630 KLHL15 AC010931.3 ZSCAN5B FARP2 LINC01995 MAPKAPK3 PACS1 AC010320.2 INTS6-AS1 RORA-AS1 AL512361.1 AL139383.1 AC124657.1 LINC01795 TRHR LINC00992 CENPW AP005433.1 AL121949.2 MINPP1 OR7E111P SH3D21 IPCEF1 CYP2AB1P AC097486.1 D2HGDH C1RL-AS1 MPP6 TBL1XR1 DBNDD1 SZT2 MUC17 TMEM126A AL731661.1 PDE5A PPIAP5 ZNF541 OR5G5P SCLT1 MOCOS UBE2H TMPRSS7 CALCR AC037450.1 MBTD1 USP33 UNC13A RPS8P4 CCT3 AC105362.1 PSMG3-AS1 DSTNP5 GCN1 MED21 SPTBN2 TOMM70 KLHL31 ATP2B4 KANSL3 HHLA3 AL807761.4 AC079380.1 CCDC85C PDHX TBC1D32 C1orf105 HEATR6 RUNX2 KRT18P49 ATP5F1C FGF10 POLR2A MBNL3 CROCCP2 LINC01331 RSPH10B L3HYPDH ZNF204P RASGRP4 SCN7A PLG TYR TP53TG1 IL36RN TMEM64 MED12L ZDHHC21 LEKR1 LINC02580 AC082650.1 TM9SF3 SLC9C2 RBM41 CDH7 PRRG3 CHIC1 SULF1 PRDM5 PUDPP1 KIF3A AC009387.1 C13orf42 PUDP TRIML2 AC010148.1 FAM168A RASA1 QRFPR NONO FAT1 KLHL42 MBP LINC01798 AC024940.1 STAM2 PIK3R1 HELZ HMGN1P11 C17orf112 ADAT1 CENPI ANKRD33B KCNQ3 AC016205.1 ABCB11 AC010493.1 CFDP1 PLA2G16 RPL21P39 AC099673.1 AC090825.1 PROX2 GPR63 ZHX1 LINC02522 CLCA4-AS1 KDM2B MARCO BRD7P1 MIR4500HG LINC02177 OR8B5P ST6GAL1 IL9 RAB28 RNU6-320P CCDC144NL-AS1 NSFP1 TLE4 AC010327.4 ST3GAL5 MRPL23 DHRS1 DEK VPS25P1 AGBL2 AL022722.2 DCN AP001628.1 NRBF2 RSF1 PCNX3 AL133163.1 PTCHD1 TMLHE STX6 TPR AP003059.2 BPI BNC2 KRBA1 AC107029.1 STAB2 CCT7P2 FBXL4 EIPR1 AC106900.2 PTDSS2 NFU1 UGT8 ALG1 SFXN5 SESTD1 SMAP2 ZBTB46 MAP7D1 TWIST1 JRKL MAPK6P1 EEA1 AL078581.2 EHHADH AC026826.2 SI AL449403.1 KCNE4 RPSAP27 BAAT SLC38A11 CH25H TMEM206 CHST3 THORLNC TFAP2E AP002414.5 RBMS1 COLQ FRMPD2B GBX1 RF00096 LINC01639 CDIP1 NPIPB1P DARS2 ERBB4 DNASE1 OR2T33 TNIK AC093827.1 WWC2 GSTA1 AC004987.3 MPPED2 AC092447.7 MEF2C-AS1 LINC01257 ADD3 TAF4B LINC02226 TUBGCP3 NBEAL1 CLGN ENPP4 SMARCC1 CLHC1 FHL3 SLC37A3 LINC00355 LINC02465 USP20 L3MBTL2 TBC1D31 CHSY1 AL513329.1 CRY2 PNPLA4 MAML2 NFATC1 CD55 LYPLA1 SSX2 AC023796.1 LNPEP RPL22P22 CCDC39 LINC02532 NEDD4 ZNF43 OR5AU1 ZRANB3 RBBP9 RBM33 SPATA17 FMO4 AC010198.1 GLRX2 AC017037.1 HSPBAP1 PRKCI PRTFDC1 AL022310.1 GSTA9P ALKAL1 PRY2 ARFGEF3 ZNF175 MAP3K14 PHC3 PPP1R8 GOLPH3 C12orf50 C6orf203 PRKG2 AC090679.1 FANCL URI1 SOS2 CDK2AP2 LINC02320 USP10 ZMYND8 CCDC186 C9orf163 OR52I1 LINC01695 FAM135A STRIP2 RN7SKP206 LINC01412 FAM156B AL929601.2 AP003715.1 Z93930.2 OSER1-DT AC073264.1 CICP22 ATP13A4 RNU5B-2P TENM3-AS1 AC055758.2 RCC2 AC090115.1 SHCBP1L LINC00535 THSD1 OTX1 TEKT3 STOX2 DNAH1 ABCC4 VGLL1 LLGL1 LINC01942 HUWE1 POLA1 RRAS2 SEC14L3 AL135938.1 MYPN TBC1D2B KRTAP9-6 AC105415.1 WWC1 SENP6 GAPDHP32 VEGFC XRCC6P1 ZBTB16 AC012213.1 AL590867.1 NGB RSPO4 FAM167A FMO8P ZNF518A LINC01892 PHC2 LINC01142 ST3GAL6 CA5BP1 ARSB AL133371.1 AMPD1 AL731556.1 ANKRD11 ITPR1 AC018521.1 EIF5A2 EMX2OS ARMCX5-GPRASP2 RABGAP1 LINC01924 OXR1 AC010132.1 AL355615.1 AC140658.3 ENPP7P8 GRHPR SEMA7A AC008565.1 DAPK2 DDX18P3 AC005562.1 MINDY3 HHIPL2 ATP5MC2 ALDH7A1 EDEM2 OR7E162P SLC26A11 RCAN3 ATG13 GPR149 STAT6 NEXN-AS1 ZNRF2 THOP1 ZNF787 UTP23 AGXT2 MIGA1 C8orf34 AC114316.2 MLLT3 ABLIM2 BTG4 JAZF1 ELMOD2 CCDC130 RSPH10B2 MTUS1 C1orf140 AC092894.1 AC018866.1 LINC02513 LINC02583 KPNA5 VPS35L ZNF184 AP001836.1 PPP1R3A FBXL19 VDAC1P9 AC006296.2 TLR3 DSG1-AS1 AC090888.3 CDC7 MCCC2 HEATR1 AC107021.2 TMEM154 AC006372.2 MRGPRX1 NUP210 AC093106.2 AC012574.1 AL355674.1 XPR1 PKN2 AL139231.1 RGS2 GFRA3 GOLGA8M PSG9 LINC01060 PRKAA2 AL390718.1 SGPP2 ARMC3 AC092818.1 TBL1X IGKV2-38 SCYL2 LINC02531 VPS37C AL592291.1 SAP130 LINC01120 PCDH1 MRPL32 TRIM67 OCIAD2 AL353072.1 MAOB LINC02430 AC113386.1 MIR4445 PCDH11Y LINC00358 TRAV26-1 AC092673.1 AL445647.1 BEND2 SNPH AC084064.1 TAF13 RPS3AP25 LPA OR3A3 RNU4-73P LINC00383 ZNF876P PPME1 TNPO3 AC010476.1 AL133346.1 AC008060.3 AC004943.2 GRIK5 AL392086.1 ERBB3 RN7SL605P FREM3 PSAT1 RPS3AP35 PUS7 AC007861.1 AC123905.1 AC099508.2 OPRD1 COMMD10 SACM1L ADGRL3*

		CX3CL1 EFEMP2 PCM1 TSG101 LINC00942 PAQR5 SMIM36 ITGA9-AS1 AC025947.1 NCAPD2 OTUD6A PPM1L ABCB4 FAM185A FRK AC117440.1 RNVU1-15 RTP4 DZIP3 CD48 RNU6-1032P RBMXP2 LINC02516 FGD5 AFMID AC093599.1 NOS1AP BAZ1A AC003035.1 TMC3 OTOGL ZFAND3 AC118757.1 TCERG1L IFNA4 LINC02235 LINC01505 ZNF517 HMGCLL1 XKR6 ACVR1B CCDC15 AC087241.3 FAM83D COQ8B RBMX MPP3 AC093802.1 DTHD1 AL122019.1 RAB11A PPP2R2B JAK1 AC078785.1 GPD2 ZNF800 SCUBE2 GALNT11 ACSL6 RF00416 EI24P2 KCNB1 LYRM4 LINC02099 AC025260.1 AC093426.1 RP1L1 GPRIN3 GNPAT MYPOP RAP2C-AS1 PGBD2 AC113208.2 AL357079.1 GUSBP2 PLB1 ENC1 KYAT3 HPS5 ST13 PTP4A1 MARS VOPP1 RPL12P41 OR7E109P PSMA8 AC073333.1 MIOS TDO2 AC096669.1 RNF19A AC104071.1 AC242426.1 SPATC1L RABL2A RN7SL761P GTF3C1 CES4A SEPHS2 PRAME TEPP U91319.1 WNK1 ADGRV1 SPRYD3 SCN2B PDIA4 CYP4F11 NOTCH3 TRIM15 ANP32B PPP1R26-AS1 NUDCD1 TRBV5-1 AC009093.2 RNU6-641P SNAP91 DAPK1 MAP3K19 ALDH9A1 LRP11 RNA5SP443 PDLIM1P2 NPTXR FO393414.2 GATA4 AC241644.2 ELMOD1 SLC25A43 SMAD5 GARS TCP10L2 IGLV3-4 PFDN2 AC125603.2 RF01210 RPS4XP18 BIRC3 RNU6-830P ZNHIT6 RNGTT TNIP3 PIGX ZEB2P1 CMTM8 AC021086.1 OR2B6 DAZAP1 FNBP1L TRIM33 AC005062.1 NDUFAF4 MSH3 SLC16A14 UNC5A PIKFYVE FMO10P AC007389.1 TSC22D1 AL133445.1 ELOVL5 AC011498.1 SSX6P ACSL3 LINC01515 SDAD1 KPNA3 ANLN ZNF32-AS3 PRR16 IGKV2D-38 DYSF BMT2 AC106806.2 OR1D4 MTHFD2P1 PPIL4 LINC01269 AC025741.1 RN7SL571P AP000253.1 IP6K1 DKFZp779M0652 AC110079.1 AC092944.1 SNX18P14 AC091173.1 AC007834.1 SORT1 USP7 SLC35D2 PPP1R9A SAA3P LINC02068 CCDC185 C14orf132 PRPS2 P2RY10BP GCK CELF1 AP003306.1 LRRC52-AS1 SERF1A ZZEF1 FOXB1 AC008448.1 SENP7 AL359643.1 AF279873.3 AL139158.2 WNT3 LIN52 POU6F1 GDAP1L1 NDUFC1 KIF2A AC034199.1 ZNF79 ARL6IP6 UBXN7 AC006148.1 DRAM2 RF00019 HLF AC004830.1 RNU7-182P TMED9 COLEC11 APOBEC3B OR1D3P AF064858.2 AC073370.1 COP1 LINC01794 PBX1 FN1 AC008869.1 AL031733.1 ZNF275 RPS6KA6 CDHR1 LINC01445 AC112176.1 FAM71F1 PROX1 HPF1 AC023245.1 BAALC-AS1 DEF8 MALINC1 AC009005.1 RNU6-477P KCTD10 SYTL4 TRBV5-6 ADGRG4 AL137145.2 DDX39A DIXDC1 FAM114A2 LRRC2 HAGH RBM26 BRINP3 AC134698.4 SGK1 LEF1-AS1 LINC01237 AL034397.1 CHST10 MIR4495 TRAPPC2 NFKBIZ AC104695.2 SPAG9 SLC39A9 LINC01861 NKIRAS1 TCEANC RPL7AP57 AC156455.1 USP17L18 AF038458.2 SVIL-AS1 HNRNPA1P34 LRP5 AL772202.1 CTNNB1 ELK1P1 USP17L19 ANKRD17 B3GNT2P1 AC134915.1 VEZT PRDM6 SOCS5P2 RN7SKP49 AC078980.1 LAMC3 UBE2Q2 AC062028.1 PGGT1B KCNN3 LINC01339 LINC02031 SH2D3C IBA57 LINC02147 AL163642.1 GAP43 LINC01799 POFUT2 EDNRB-AS1 NTPCR C10orf143 HIPK3 AL133523.1 LINC01251 HNRNPCL1 KIF9 PITPNM2 ZNF780B ENOPH1 RN7SL290P PGLYRP4 ADRA1D RNU6-144P LINC02463 ORC5 BIRC2 NLGN3 ZC3H12C PLS3 AC005244.2 CKS1BP6 RCC1 PRNT OR52B3P ASAH2B ZFPM2-AS1 PDK4 LINC02220 AC074254.1 SLC44A3-AS1 GOLGA2P7 SLCO6A1 PRDM1 SMAP1 BBX ARMC2 SMPD4P1 AC006272.2 CACNA2D2 PTPRC KIAA1109 BEST3 LINC01681 CCND3P2 AC136489.1 AC007100.1 HDDC2 Z93242.2 OR4C1P MIR4453HG ZNF767P AL353651.1 COL4A2 CCT4P1 C1S BRD7P5 ME2 AC104009.1 AC093716.1 EXT1 CLEC4D AC073316.1 FOXJ3 OR1D5 BCAR1P2 LINC02432 RSPH14 PARP12 SYNE3 SSBP3 MAP3K21 CYP4F22 LINC01182 FAM86C2P AC099520.1 AC004540.1 HSD17B3 MSH4 NARF AC026765.2 LINC00476 UGGT2 DHX8 CT55 ICMT PIP4K2A AL355075.4 LINC02313 UBAC2 SLC25A17 TTC39A UGT2B25P CRYZL2P-SEC16B LINC02283 EML6 CPSF6 PRSS55 LINC00858 EYA3 XIST PPIAP85 LINC00877 HSPA12B HELB MGST2 CTPS1 NCKAP1L MED13 NRGN SMIM31 AP005436.2 AC099398.1 AC025283.2 ZNF423 RNU6-1113P GUSB COPS3 EHMT1 RAX ZDHHC13 PLPPR1 FGF5 C4orf51 OR11Q1P REPS1 FBXW7 AC090791.1 SUCLG2-AS1 OR2B7P GSTA6P GOLGA6L4 KIF13A KRT7 KRT18P59 DLGAP5 AC048387.1 WAC-AS1 DPEP1 TMEM87A RNU6-374P CD209 TIPRL AC009107.1 LINC01115 VPS54 STS TMEM123 LINC01476 CLSPN RAPGEF2 PLEKHD1 PRIMPOL BUD13 SUCO IL32 EPHA5-AS1 KDM1B AC026336.1 RN7SL468P EEF1GP2 CPS1 ALG5 NCOA2 DCAKD LINC02270 TRAK1 USP12 EIF4H AC118942.1 KATNAL2 BOC XRCC5 RAB1A NFXL1 C2orf69P3 USO1 RUFY3 NFKB1 MANBA RBMXP4 RNF8 ZMPSTE24 DEDD2 UBE3A ORC4 AC135506.1 FAM117B RSL24D1P9 PLPPR4 GRK7 HTR2A EPAS1 R3HDM1 KRT12 MKRN1 POU2F1 CLNS1AP1 LINC02444 AC131011.1 GALNT1 ANKMY2 SPINK8 LINC00364 RTN3P1 BCAR1P1 RNF123 AL035413.1 LINC02384 ACOT8 LINC01847 DNAI2 CCDC148 SELENOT ENPP7P6 BCAS3 AC010105.1 TTTY10 SLC16A1 SLC17A5 RNU6-535P TEX15 VGLL4 AC114781.4 C1orf43 PPIP5K1 GOLPH3L SYAP1 DCAF17 LCEP1 PDE3B UCK2 TET1P1 SLC30A9 RBMY2JP MEF2D MICU3 RNF25 AC092364.1 JPT1 KMT2E PTEN ARSF AFF1 SUSD5 ATP1A3 ERI1 XRCC6P5 UGGT1 RAC1P8 NOSIP SYNPO2 NUP210L LMNTD1 LYRM2 FBXO8 XAGE5 SLC25A26 TCAF1 RNF141 LIN54 AC004869.1 AL078590.3 TCP10 BIN3 ATF3 AC005840.2 LINC01934 NPHP1 FBN2 AC234771.2 RTN4R AC111152.2 TPM3P4 KLHL23 GPR173 PCMT1 LINC01556 LINC02534 TMEM222 MARK1 AC114550.1 CABP5 ABCA1 SLC8A3 AHCYP3 MBOAT1 ULK1 MALRD1 RPL9P15 USP24 LINC02477 AC122683.1 AC017002.3 RPS24P8 AC122685.1 KCNH2 CAMK2D IFNG-AS1 SDHAF3 MROH8 AC096554.1 WASH8P DHX30 LINC00649 PTPRB AL390760.1 CASC3 LINC01525 AC012468.1 CATSPERG ITSN2 NECAP2 PPARG AL353678.1 AL357315.2 MMAB ISX-AS1 CACNG4 RASGEF1C MTTP NYX WIPF3 PARP14 MAGEA9 TMEM165 AL663109.1 AGAP9 AP000282.1 TAMM41 AL357060.1 IP6K3 FAM169A DYTN HSFY4P MAP2K6 ZC3HAV1 FAM149A TENM1 NBPF20 HTR3B DDX42 TXNRD2 LINC01337 AC003958.2 ODAM AL512303.1 RP1 SUB1 DEFB103A SMIM12 AP000442.2 SNX4 MYO1H SELENOF AC020718.1 AC105430.1 GOLGA8A AC019186.1 CCDC158 LINC01228 LINC01477 REV3L GAPDHP64 NYNRIN EPRS LINC01470 PAXBP1P1 NFYAP1 CCDC134 C6orf99 S1PR4 MYLK4 SPATA22 CWC27 MTCYBP14 RN7SL865P RBMY2NP PRKX AL137224.1 KIF5A LINC00278 SERF1B CNNM4 AC010754.1 AC018450.1 AC107057.1 CAMLG LHX8 CIDEC AC104457.1 CD300LG LINC02455 CTH IL15 MEI4 PAPPA2 ANKRD18DP PFKFB3 SIN3A OSTM1 PHBP21 CRYBG3 GALNT7 AC107220.1 AC010203.1 COQ3 CSNK1D AC008591.1 AP005357.1 AL157400.3 CXXC5 AC073587.1 TP53BP1 OCIAD1 AK2 AC099518.1 AC009961.1 TES RAB9AP2 SH3TC1 LINC02005 Z83844.1 AC020704.1 CUX2 AFG1L AC005229.1 CCDC151 MCEE DDX4 LINC01249 AKAP4 RAPGEF4 HEG1 AL357055.1 OR5F1 LAMC1 CLTC AL441964.1 PBX3 ZNF761 MTCYBP4 PDXDC2P-NPIPB14P ANGPT1 PSMD14 AL133372.2 LINC01048 LRRC47 AC004522.2 SLC4A10 ADGRG6 ACSS2 BAG6 AC144568.2 ITGA4 SEC23A FERMT2 TSSC2 PHLDB1 TPD52 AL390860.1 PTPRK STK11IP ZNF45 GAPVD1 TBC1D3P4 KIAA2012 LINC01555 AC005614.2 CNNM1 SS18 AC138035.1 ITGB1BP1 ZC3H7B STARD6 AC087672.2 TRBV7-6 CLCA4 AC064862.6 SMURF1 DEFA7P AC111152.1 BTNL8 OR2A1-AS1 BCL2L11 FOXL2NB TRBV4-1 METAP1D LINC00899 UBE2Q2P8 BRIP1 GRID2IP SLC7A5 CYP20A1 SORBS1 AC113355.1 PACSIN1 LRCH2 AC113398.1 AC097510.1 ZC3H12D AC092436.2 SRSF10 MAP3K2 STRA8 CYP4F62P RF00411 ARRDC3 TBPL1 SLC16A13 ZNF844 NIN SLC1A4 GRIN1 CALU OR1A1 LINC02488 TNFSF13B DENND3 AC004840.2 XPA AC073172.2 MMAA INTS9 IRF5 HNRNPM ARL14EP AC040963.1 ELOVL6 AL359837.1 AC016708.1 DLX3 UBE2E1 LINC02149 COL14A1 LRRC4B PPDPFL RPL21P12 SLC20A2 AC007879.1 AC019197.1 NPIPB10P SLC25A33 PARL TOGARAM1 AC018742.1 AL049697.1 RNU6-1047P SLFN13 LINC02615 ZFYVE26 TBX20 AC011287.1 DST AC131211.1 LNX2 ERP44 AC022166.1 JMJD1C EIF4E3 MAGEA1 RUSC2 RBMY2KP ZCCHC8 INPP5D KLHL29 CFAP161 OPRM1 ACER2 LINC02475 CALML3-AS1 CNKSR1 MPDZ SDHC UBTFL10 LINC02382 DOCK11P1 AC011890.1 OR6C66P OXNAD1 AC090833.1 FAM122C PDCD11 LINC00430 FLT1P1 NT5C2 AC110995.1 OAF SLITRK1 AC092809.4 MTSS1 PPFIA1 PROX1-AS1 HBS1L DENND4C CDK19 PITPNM3 SDR42E2 CCND2 CUL4A AC137579.1 HSP90AB3P PTPN3 FAM217B AC093801.1 LINC02386 TJP2 AC003958.1 PAX3 TXNRD3 LINC01510 AL391361.2 AL356218.1 MYL6P3 SSX2IP LINC01748 TTC17 TAPT1 POMT2 AC092978.1 TOPAZ1 BMPR1B CFHR3 AL139344.1 AC245291.1 NLGN4Y DEFB103B AL359317.1 AC096644.2 NAA25 LINC01234 CLUAP1 RNU6-65P AL591074.1 AC010884.1 GMPS LINC00886 GPT2

		AC034231.1 NPS RNU6-364P USP17L17 AC142381.3 NDUFA10 ETNK1 B3GALNT2 CLNS1A NUP43 CYP4F30P BCAT1 KBTBD12 GGTA1P ARL17A ZNF398 CAPN7 ZSCAN22 AC024909.1 AC073311.1 LINC00845 AC079950.1 LINC02379 ZKSCAN7-AS1 KDM3A TULP3 APBB1IP AL390962.1 ADGRG7 SOCS5P1 AC009313.1 RNA5SP45 TAOK1 UNC45A RNU6-111P RPL3P7 RASSF6 FLNA AL359853.2 AC119396.1 AC138915.2 AC011474.1 RFTN2 AL161727.1 AF121898.1 AJ239322.1 TTC1 AC092620.1 SIN3B OR51P1P EIF4A1P7 TP63 RETREG1 FAM49B DNAJC13 CNST LINC02208 FRMD4B AP001351.1 CYP2R1 CRISP1 DNAJC10 RNF169 LINC01478
4920	4042	*DDC8 USP17L28 CD44 SAMD4A TRAF3IP2-AS1 DPY30 KCNMA1 MIR4677 PAX7 PKNOX2 RALYL ZHX3 C15orf27 APBB2 C10orf25 REXO1L12P VPS4A ZNF799 FRG2 GGT3P NCF4 CST2P1 MTHFD2L RNU6-498P LCORL MIR4461 ALDOAP2 BSN-AS2 FGFR1OP2P1 DLEU7 SCAF8 EP300 NIFK-AS1 RDH10 GPSM2 CPXM2 OR51A8P LRRC71 KCNA6 MIR3156-3 C4orf22 BCRP6 CDK14 ERG PARN CHD9 UBE2CP4 EPN2 MSRB3 XK PDE1C SEMA4D MREG AK7 INIP NINL SLC12A8 TNS3 NR4A2 PIR IGSF3 RRP7A ZFYVE1 MED13L CXCL13 KIAA1199 SORL1 TEAD1 CTAGE5 SNORA46 SLC22A2 LINC00698 RPS6KA5 CNTN4-AS2 SUGCT ATXN10 RNA5SP359 DHRS11 BMS1P11 TAOK3 ZNF510 GADL1 STX8 EIF4G3 ACSM3 A2M BAZ1B SLCO2B1 ROCK2 RNU1-77P TEK OR5D3P PHF20 FOXK2 CPNE4 GRK5 DGUOK FGGY RNU6-749P SRRM4 FAM73A ARHGEF26-AS1 OR8J3 ANKRD6 PYY KCNC1 STARD5 GPR55 GBP7 DNAJC15 SLFN12 RSU1P2 STAU2 OR9L1P PCDHA13 SIAH3 LINC01121 C2CD3 CD38 GML CCNE1 LINC00159 IGHV3-25 RPL26P9 GSTA2 ISM1 RNU6-132P ANKRD20A3 CORO2A OR7K1P ARHGAP42P4 MIR4499 FBXO31 FRS2 C12orf40 AMY1B AP4B1-AS1 CDK8 HDAC7 FAM19A4 PIWIL3 FPGT CDK1 NLGN1-AS1 N4BP2L2 ZNF232 A2ML1-AS1 TRAPPC9 RPS15AP6 BANF2 FAM90A6P FAM182B KIAA0100 C8orf44-SGK3 FNDC1 CLUHP4 LCMT1 LINC00924 SSXP1 SAA2-SAA4 BCL2 MMP16 CAMTA1 MIR654 ZNF239 LINC01047 OR4M2 PLCL2 BDKRB1 C4orf29 WDFY2 FAM90A3P PXDN GXYLT1P1 RNU6-436P TOMM34 MIR1200 RAC1P5 ADAM32 TOX3 CHST11 PWP2 DNAL4 ANHX ZSCAN5C IPP CDC42BPA F7 CNOT1 SLC13A4 ADAM23 B3GALT5 RAG1 SAMM50 CDC42EP3 TSPAN7 MIR1185-1 RHOH ATR IGLV3-25 ADAM20P1 PAQR8 FSIP1 HERC2P8 MIR4768 GRIK2 SNX25 RNA5SP284 GNRHR SUZ12P SDC2 MAPK10 SARS GAGE2B LAMB4 MAN2B1 OTX2-AS1 PRDX4 AGT LRRTM3 DAPP1 CHRM1 HMGB3P24 FUT4 OR4A42P CD53 DBH FAM46D OR5G1P LINC00162 GPATCH2 MCOLN2 ZNF227 RPL7P55 CDH8 LINC00851 KRT222 UBE2L6 IPO13 SUV420H1 GMFB ARHGAP15 LTBR CCR3 HBG2 PRR5 PCSK6 SPRR2B IGLV2-23 SALL4P7 RNY4P7 PALM2-AKAP2 AHI1 LINC00486 UBE2W SCN11A LPIN3 ACTG1P1 LRRC53 ATP10A RTCB C7orf60 ACAD11 PCMTD1P3 NEGR1-IT1 SKAP2 SCHIP1 RNU7-177P IGLV3-7 SYTL5 MOB1B PIGL GDI2P2 HUS1 KIF19 DYRK4 GOT2 ZNF667 CYCSP34 GRIK1-AS1 MIR4789 PPARGC1A KCNJ6 KCNK10 PCAT1 CASP5 ZNF709 BRD4 UPP2 NDUFA13 MIA2 ZDHHC11B LINC00517 MLIP-AS1 PCDHGB4 S100Z ANKRD26P3 GPR158 DNAJC9 ZNF568 ITSN1 OAS1 LIN28B TMEM241 GRIP1 TTC3 DUX4L3 SIDT1-AS1 P2RX6P ELMO1-AS1 RN7SL16P C3orf22 PLK1S1 CMA1 MIR3118-2 PAWR TBC1D3B TLK1 UPK3B PREX2 LDLR URGCP-MRPS24 SNORD11 ELAVL2 RPN1 FAM9B TANC2 WDR16 PGM1 ZNF863P DEFA1B DLGAP1 DLGAP1-AS4 DUX4L15 DNAJB4 TMEM189 MIR519D PCDHGA10 C15orf60 GTF2IP1 SVOPL EIF2S1 JAK2 LGR5 POLR1A TMPRSS4 TSHZ1 TM9SF4 BICD1 LRRC16B AKAP2 LRP1B ARMC10P1 LRRC37A12P ZBTB8OS ABCG8 H2AFZP1 PHGDH ICA1L RNA5SP470 OTUD7A TPTE2 EMCN-IT2 MIR491 KALRN CCDC60 OR8K3 MKLN1 DOPEY2 RAB11FIP4 SDHD SUMF1 NF1 RPL39P36 RPS17P15 PPFIBP1 USP24P1 FAM49A MAP4K3 SNTA1 PCDHGA5 CACNG2 MIR4439 IGLL5 BTBD9 SNORA75 OR9I3P FPGT-TNNI3K SAFB2 NCK2 FMO9P DNAJC25 CD93 GEMIN8P3 CCDC144A OR52T1P FKBP5 ERCC8 TAF1D OR11H12 IGHVIII-47-1 TMOD2 PGBD4P7 SCNN1B SLC44A5 PLCB1-IT1 MEGF11 SDK2 DICER1-AS1 C18orf64 CCT6B CLIC5 PCDHA12 FBXL5 RNU6-1241P RNF43 NR2F2-AS1 TRHDE SNX18P25 ZNF677 ZNF536 MAP2K4 CSRP2BP HS3ST5 CD72 KAT5 MAP6 SDR39U1 MAS1 OR2L13 PARVB CDH11 ZBTB41 CDK2AP2P1 MME FAM213A GRIN2A MAST4 WDR26 NRXN1 RNU6-723P SNORD116-27 FAM230C YJEFN3 DSCAM-IT1 SPTLC3 DTWD2 ULBP1 AGBL1 MALL PARK2 LINC00226 MAPK8IP2 NELL2 ARID1B DEFT1P2 FLJ00273 DPY19L2P2 C21orf2 MAGI1 LAMA3 RNU6-554P SLC14A2 BZRAP1-AS1 RNU7-87P GSK3B PDXP KIF9-AS1 VPS37B KIR2DL1 PCDHGA4 FOXO1 ADAMTS17 PCDH9-AS2 C9orf135 WDR82 PKP2 MTND6P3 SPATA16 AMPD3 NPHP3 ADCYAP1R1 SFXN3 SNORA71 ST6GALNAC3 PDE6G LRRFIP1 GCLC TUBAP SERPINA4 MTCO3P2 ADAMTS6 TBPL2 IL18RAP RNU6-164P TRIM16 CDC40 TNR-IT1 DPP6 SETD3 MIR539 MIR4713 PRELID2 STK39 ACOX3 GOLGA8J RPS6KA2 SLC22A3 LINC00161 SNORD116-17 C2orf48 ARHGAP8 ZNF571-AS1 SLCO1C1 PLXNA4 PTGER4P2 GRIN3A RN7SL678P DEPDC1 GRAMD1C LINC00504 ANAPC1P1 SMOC2 TNRC18P3 ZDHHC17 KCNH1 HORMAD2 HLCS MCF2L ACSS3 LINC00604 ABCD3 DCDC1 RHOQP1 VPS26B PARVG CACNA1C FEM1AP4 PDCD6 AMPH BRI3BP BACH1-AS1 RNU6-768P CDRT4 KRTAP10-5 MAN1A1 U8 PCED1B MIR524 NLGN4X SNORD109A RNU6-1021P IGHV4-28 FSD2 LINC01162 ATP5A1 SPRR2C HTR4 PPP1R26P3 URGCP EMB TRAF6 AMY1C CKMT1B PNPLA3 C14orf183 FAM19A5 ANO4 C12orf55 RNU6-1003P KCNQ4 GIPC2 PIK3C2B C5orf64 PA2G4P3 FAM184B EXD1 ST7L CTNNA1 LINC00595 C15orf26 RNASEH2C COMMD1 SCAMP4 MYO9A FOXN3 PFKFB2 HNRNPA1P61 SH3TC2 RNU6-917P WDR33 RN7SKP233 OR5K4 PCMTD1P2 RN7SL92P PAK7 CHD7 RNF126 RXFP4 ANKH C19orf18 BIRC6 ADHFE1 SH3PXD2B PTBP3 IGKC ANKRD20A14P AK8 PCAT2 MDM2 BCRP7 MIR544A NDST3 NBEA POTEC FAM19A2 CDHR4 GBE1 ANKRD30BP2 ZNF285 SNX5 RASGEF1B AFAP1 NCAM2 OC90 DPY19L2P1 CYSLTR1 PAPD7 MIR154 SNORD115-35 GPR161 TIAM2 OR51B2 SLC20A1P3 NR4A1 ZNF10 TIGD4 SLX1B-SULT1A4 TRAK2 CEACAM5 C14orf37 MYT1L ZNF878 SRGAP2B STAG1 UPB1 GIMAP4 CLDN14 KCNA3 IQCJ-SCHIP1 SCARNA15 CARM1P1 ZHX2 KIR2DP1 LRRC49 C1orf177 SMARCA4 SOHLH2 HERC2P3 TAGLN3 FAHD2A CNTN1 NFE2L3 TTLL9 PALMD EML4 STK31 ENPP7 TTC39B TMC5 DUX4L12 KIAA1257 HIGD1AP13 PLEKHB2 GLIS3 OR4K6P TMEM50B SNORA1 ANKRD36 MTATP8P1 ARHGAP29 HBE1 GOLGA8K RTCA NRG3 APOOP1 CAMSAP2 LINC00970 RNU6-141P ABL1 PTPRG NCOR1 ZNF433 RPL7L1P12 ABCA11P PLAC1 PWAR6 MC2R TBX3 CHRFAM7A MIR620 ELMO1 DIO2 RPL13AP2 PAAF1 SLC24A4 SLC5A10 CABIN1 TRIOBP SYNE1 A2ML1-AS2 TUBA3FP HRH1 MRPS31P5 FBXO47 CYP4F33P OIT3 ZBBX FBXL17 PKHD1L1 SLC9B1P4 HTRA1 PDZD2 ZNF585A IFT88 GUSBP11 ARHGAP5 HSPE1P19 ZNF160 KIAA1731 DNAH10 GAS2 GRIK3 CACNB2 PDE10A NUMB TNIP1 PAN3 FSIP2 TOX2 PSG10P ZNF720 NABP1 LIN9 RHOJ CLUHP5 MRPS31P4 BRE FAM9A ZNF474 SEC14L2 OGFOD1 ZBTB8B BACH1-IT2 SOX6 CIRH1A CCZ1 POLE TSPEAR LINC01035 TBC1D22A FILIP1L TTC4P1 COL4A6 CATSPERB ZNF559 ZNF559-ZNF177 TRPM3 ZMYM4 STK32B SLC7A7 TACR3 VCAN BRMS1L DNAH8 NHS IGLC2 CNTNAP5 SLC1A3 MIR3173 RPS3P6 PCDHGA11 C14orf119 DNAJA1P1 MIR670HG RNA5SP438 ASNSP5 SHANK2 LRCH1 CHP1 SAA4 RAPGEF5 MIR3666 TCL1B RN7SKP218 RPA2P1 SLC39A10 CDCA2 DTD2 IGHVIV-44-1 PDIA6 PTGFRN RNU7-119P SNORA76 NCK1 KCTD8 SDIM1 CHCHD6 RNU6-631P SPIN3 CPB2-AS1 UNC5D CD8B HS3ST2 MRPS21 ZNF830 EVA1A MAGEA11 ZNF567 SH3BP1 MIR605 SIM2 CHCHD2P9 GABRA5 CCNB1IP1P2 KIRREL GPC3 PDGFRA DOK5 TLK2P1 PIGUP1 PTPRJ DMD NANOGP4 C22orf39 RNU4-56P STAMBPL1 ZRANB2-AS2 ZNF737 STAG3L2 CRYL1 OTC AGMO MRPS6 HADHA IGHV3-64 PER1 RNU6-46P ANKRD20A4 MIR3118-3 FNDC3B MYH4 WFDC10B ADAMTS9-AS2 ASTN1 MIR1324 AIFM3 GNG12-AS1 PCDHGB3 NF1P8 CENPK ATRNL1 RN7SL714P KIR3DL3 DTX2P1 SUN3 SLC27A2 DUX4L2 PRDX2P3 CHRM3 WARS2 RPL23AP60 CPE C16orf3 RNA5SP385 TUBGCP5 LINC00587 UBE2V1 RFFL CALD1 MBOAT7 IGLJ2 POMC KIAA1462 NLRP12 MIR4681 DNM1P32 TLK2 ZNF2 COL24A1 SNX18P8 MIR759 GAK ATP5F1P6 C10orf113 HSF5 PSPC1 ARHGAP24 ALMS1 TERF1 TMEM132D SNORA16B COL11A1 VN1R91P SLC13A3 SPRED2 N4BP2L1 C11orf65 LINC00907 PITPNC1 SLIT2 TPTE2P6 ITGAE ANKRD18A C2orf88 CACNG6 CEP128 MCM3AP-AS1 LRIT3*

		ZSWIM3 ZNF804A IGHVII-67-1 WDR83 COMMD6 SNORA67 MIR1254-2 NARS2 OR4N3P TTC4 C15orf54 XKR4 OR10AK1P LPPR5 PTGER4P3 PCOLCE2 IGLVI-68 LINC01143 SLC26A7 GPC5-IT1 C9orf3 KIAA1432 ROR1 MYO1D RAPGEF4-AS1 MAPK4 OMG ZNF486 KAZN RN7SL83P ADAMTSL1 KRR1P1 ZNF675 EZH2P1 CSNK2A1 CWF19L2 NRG3-AS1 VN2R17P EXOSC3P1 OR4F16 RNA5SP353 MRPS18B AOX1 USP17L27 DTNA SERPINA5 PMS2P9 AKT3 PHKB ADAM2 NDEL1 RBAK LAPTM4B LINC00472 GULP1 TRIM5 KCNS3 ARHGAP11B RPL23AP82 PCDHGA3 THEMIS VWA3B SLC5A8 FLVCR2 SDHA CYP4B1 STEAP2-AS1 MEDAG MIR4529 RNU6-810P MTND2P25 ZNF280D GAGE2C FAM230B RHBDD1 OR8K2P TTC26 ZNF22 PSD3 ABCA13 CHRNA7 HOMER2P2 HECW1 HMGB1 MEF2C FAM174B MAST4-AS1 PSPH RAP1GDS1 COG7 IGHVII-31-1 IGHVII-22-1 KLF3 BAIAP2L1 EFTUD1P1 RN7SL130P SDAD1P4 IGKJ5 RPTOR LINC01013 KANK1 KIF26B GPHN SLC6A2 C1orf106 PTN LPP ATRX DMRT1 TVP23C ELP4 RYK BID IER2 SNORD115-48 TGFB2 TRAF3IP2 LAMA2 MNAT1 ATP6V0D1 CD300C NPM1P41 LZTR1 TRIO ZNF385D IQCK C8A CAPN3 SLC15A5 TFDP2 CTNNBL1 CCDC12 IGLVIV-64 REXO1L10P PSMC1P13 RAD51B DGCR9 ZNF248 PTPRE RNA5SP487 PTK2B FAM20A LINC00645 RNU6-321P FBXW8 RALGAPA1P SH2D1A MYO3B DUSP22 CALB1 LINC00866 SLX1B KRT223P FAM95B1 SH3RF3 C18orf8 ZNF347 GTF2E1 STRADB KIAA2022 MYOM1 KCNIP3 PSG8 EXT2 RGS16 IL21-AS1 ZSCAN30 TUBAL3 RNU6-58P CRYZL1 MECP2 PSMA1 ZNF454 MAP3K5 BCO2 NOS1 CLDN11 TIMP3 MIR4742 RNA5SP50 STAG2 RGS3 SPAG16 ZNF299P DSCAML1 DUXAP8 PLN DUX4L8 PRDM9 FBXO27 YOD1 EML1 SMAD3 CTDSPL2 C14orf182 TBC1D12 PABPC1P12 CDK12 USP17L6P RERG HTR2C RNU6-196P NEK4 DCBLD2 PRKAR2A FAM90A13P CACNA1E SYT10 THYN1 KATNAL1 NF1P2 MAGI2-IT1 MYO6 EZH2 RBM22P2 MUC16 MRPL33 VCAN-AS1 HIGD1AP2 KIFAP3 GLRA2 PHKA2-AS1 PON1 BORA PI4KA VWA8 DDAH1 ALDH4A1 TMC2 SAMD13 RNF17 DHRS7C OR51B8P PCDHGA7 RNU2-5P RNU1-139P TMEM161BP1 PRAMEF26 FAM102B CD36 MIR3116-2 PIBF1 ARF4 LINC00448 SNORD115-20 MIR3667 TMPRSS11E SLCO3A1 TNFSF15 RN7SKP162 NCF2 GABRR2 PIK3C3 BNIP3P3 CAPN14 RNA5SP453 FAM90A5P USP16 SLC9C1 KRTAP20-2 SNORA40 GPCPD1 BCL9 UBE2CP2 IGHV4OR15-8 C9orf41 TRAF3 RIPPLY3 PLXNB1 C9orf171 SND1 ERICH1-AS1 SNAP25-AS1 MIRLET7C CHD6 DPYD-AS1 FGD4 LY86 PDXK MTND1P12 LRRC4 SPICE1 N4BP2 IFFO2 IGFBP7 NDNL2 POTEE NMNAT1P1 ERMP1 FAM24B MACROD2-IT1 FAM27B TCF12 ZNF721 VN1R7P B4GALT4-AS1 MERTK ARMC8 RN7SKP253 RN7SL801P SNORD115-36 SLC47A1 AEBP2 CORO2B OR5BL1P RNF144A USP17L30 SLC7A14 C18orf63 RN7SL864P TP73 C7orf76 GRIK4 RBMS2 ANTXR2 LINC00376 RN7SKP76 TMEM74 TAPBP RUNX1 IL1RL1 ENAH ZNF765 KIR3DL2 GPR64 C3orf33 TAC4 GABRG1 MYO16 CYP4F31P KRTAP10-1 TSHZ2 TRAV22 ZNF366 MIR548U MTND4P12 ZNF540 LOH12CR1 ISPD CD58 PTGFR SNX31 GRHL2 IGHVII-30-1 SELE IGHV1OR21-1 PCDHA2 FAR2 FAM171A1 MAGT1 MIR517A PCDHA11 CHKB SCUBE1 ARHGAP42P5 ARHGAP22-IT1 CNN2P12 ZNF595 CDH17 GNAT3 OXCT1 RPL5P35 SV2B HECW2 FAM230A MIR663A NOTCH2NL ABCA8 LINC00894 ZNF679 SLCO1B7 PEAK1 EYA1 KCNQ1OT1 FBXW12 LINC00669 SATB2 RNF216P1 ABTB2 MORC3 MAGOH2 TMEM132C MIR4477A GPR126 ANKS1B OR4F14P PPHLN1 CDH18 RNU6-185P SNORD115-21 WFDC10A CHL1 RAPGEF1 P2RX6 KRTAP19-7 HSF2BP SPOCK3 BNIP3P1 PLS1 FRMD6 UNC79 IGLVVI-22-1 INTS4L2 ANK2 SPON1 RAB23 REXO1L8P FAM8A2P ZNF516 INVS KRTAP12-2 ANO2 PCDHA9 hsa-mir-6080 HPS3 LINS KCNK17 ARHGEF3 SNORD115 RHBDL3 SUPT3H KIAA1244 ZNF840 GABRB2 EDNRB SLIT3 MAN1C1 CFTRP1 BAGE2 RCVRN NLRP7 LIN7A CYCSP27 TPCN2 CNN2P4 MPZL1 ZNF578 LRRIQ3 ANKRD30BL ADAM9 PRR5-ARHGAP8 TPH1 SNORA51 MARK2P5 RNU6-1266P GPR176 MATN3 IFNGR2 PKP4 SEPT9 RNASE9 EGFLAM FAM155A-IT1 UOX LUZP4 PLD5 SIPA1L1 DGKB RNU6-538P TGFBR1 GBP2 DUX4L13 RPL19P12 TTLL2 UACA LRPPRC ACVR1C HEXB RPL34P3 DPH6 ZNF209P TNKS ABI3BP DNAJC25-GNG10 TSNAX TAS2R1 YBX1P7 IGHV1OR15-6 TUB KLF12 ARHGEF18 PCDHAC2 AXDND1 DCAF7 LNX1-AS2 LINC00473 WBSCR17 CDH6 PCDHGB2 NDFIP2 RBPJP7 GABRA2 RIMKLB MIR4327 KRT126P TRAV8-5 TNS1 MDM1 OVOL2 SSUH2 RBL2 LENG8 RN7SL516P USP53 IGHV3-30-2 AATF CHEK2P4 ACSL5 RNU6-352P LATS2-AS1 C1orf87 PRUNE2 DOK6 RN7SL449P SGMS1 GART hsa-mir-4528 LINC01122 HERC2P4 PCDHGA12 TTC7A RN7SL99P PSG1 RNU6-978P GJB7 ITPR2 SUN2 SNRPGP9 BRINP1 HIAT1 SEPT7P9 RNU6-552P OMA1 RPL30 IGSF5 SULT4A1 DRG1 MIR1299 SRP19 SIRPD TRAPPC10 KLHL25 LEMD3 KHDRBS2 GAGE2D CPQ IGLVIVOR22-1 POLQ CNOT10 DYNLRB2 SOAT1 IGHV1-3 ABHD17AP5 MAP4K5 CHODL TCAIM FOXD4L4 OASL KCTD9 MSANTD3-TMEFF1 TM4SF2 EHBP1 ACTN2 DARC SLC9B2 SHMT1 RPL36AL NEB SRSF4 SCGB2B2 POU5F1P3 CAMK2G C6orf3 HNRNPA1P7 FGF14 BRD1 MIR1290 RNU6-1184P KLHL32 CEP164P1 BCL7C RNU6-278P TMEM100 PSTPIP2 LINC00861 SNORD115-12 MIR4490 RNF165 FXR1 DNAH2 ANP32C CASC4P1 ITGB3BP UBASH3A FRG2C HDHD1 CUBNP1 DRD5P1 RNF213 LYPD6 PDIA5 SUCLG1 TMEM67 OR51I1 VDR MIR4452 CLDN12 SLC25A1P2 PRKCE DTD1 BTF3L4P1 ITGAM MIR4695 CCDC149 DUX4L7 SH3RF2 SIGLEC26P CNTN3 RPS3AP46 MGAM DUX4L10 GLIS1 RN7SKP168 PRDM11 CD163 DPT RGS5 BTBD3 RBM22P1 ACSS1 BBS2 PACRG ULK4P1 CLSTN1 PCDHA10 LANCL3 IGHVIII-22-2 EPHA3 GAPDHP69 RN7SL587P SLC20A1P1 LATS2 MIXL1 RPS6KA2-IT1 SKAP1 CC2D2A DSC3 WDR11 MIR487B RNU6-909P CNR2 KLHL33 SNX29P2 GLRX3 ST13P15 LRRTM4 CCR1 UVRAG CACNB4 PTPRN2 MTND1P2 SDAD1P1 LINC00395 LYN USP6 INPP4B ZNF732 MIR655 MYO3A MIR941-1 DKKL1P1 AKR1B1P1 MGAT2 LARGE-IT1 HIVEP2 UBE2R2 RN7SL673P PYGL FAM65B TXK GRIK1 CUL5 OR9A4 MTA3 NOL12 CHTF8 SYT6 FOXD4L2 LINC00446 NUBPL SOX5 PRAMENP KIF6 ADH5P3 GXYLT1P2 DSCAM DGKI IL12RB2 RIN3 RN7SL23P REG4 USP17L29 OR52H2P UBL3 RNASE3 NLN RNU6-49P PABPC1L TBC1D30 MTND1P23 ANKFN1 NME7 GTF2A1L PABPC1P5 MIR4519 DENND1B ANKRD30BP3 SPNS2 SKOR1 HERC2P5 RNU6ATAC33P IL1R2 RPS27P27 NBPF10 EFHB TRAPPC8 RUSC1 KDM4C FGF14-IT1 ACSM2A SLC9B1P2 CYCSP41 C5orf66 SLC1A1 ST20 DHX57 ACSM1 RNU6-1063P MIR1267 TDRD12 RNA5SP366 SNORA8 CCL8 PPP6R2P1 ENTPD4 GTF2IRD2 THAP7 MAPKAPK5P1 TMCO4 CTBP2P7 RNF144B FAM27C ZNF841 PEX3 BMS1P12 ATG4C RBM11 EPHA6 NTN1 ABCB10 LHFPL1 LINC01107 KRTAP20-3 GAGE12I OS9 NGLY1 U6 IGLC1 ARAP2 ENTPD3-AS1 LINC00937 NR5A2 LDB2 MYRFL CYP4Z2P TATDN2P3 WDR72 PPP1R26P5 SNX30 SNORD112 PKD1L1 C21orf62 NLGN1 TSNARE1 RNU6-803P STRBP DNAH9 TMEM260 OR11H1 BTF3P14 ARHGAP42P3 OR9Q1 SHROOM3 CYCSP32 MSI2 CORIN LRRC7 IGHV4-4 RNA5SP125 TEX41 LINC00221 PRSS23 RBPMSLP CPHL1P ANKRD29 EPM2A DEFB127 PDZRN4 SGMS2 TNFRSF10B SNORD45 LINC00930 SPECC1L-ADORA2A SHFM1 MIR600HG QTRTD1 LCE6A KIF18A LARP1B RN7SL743P LIPE-AS1 AQP4-AS1 C21orf91 NPM1P13 SOX2-OT CFTR CAMK1D MIR96 RNU2-27P DYNC1I1 KRT18P2 FLRT2 WRN SDF2 NALCN-AS1 KMT2A RN7SL435P ZNF90P3 CCDC144NL XBP1P1 FAM210A TCEB1P32 RAD51L3-RFFL EFCAB6 ZNF402P BRINP2 RNU6-56P ERCC6 TEX26-AS1 PRMT3 EFTUD1 LINC00840 RN7SKP140 DSCR3 HSPE1P25 USP17L5 RPS20P5 ZDHHC14 DPP8 NIPA1 MIR4764 FAM194A MSRA EMCN-IT3 SYNPR DDX51 PCDH9 ZNF626 MKRN3 LINC00349 ITFG1 LINC01029 IMPG1 ARHGEF33 RN7SKP99 DSCR4 GLB1L3 NKAIN2 CYP2C8 FAM90A4P CAP2 RBMS3 DGCR6L CST13P SNORD56 FAR2P4 OFCC1 CSNK1G3 MATN2 REXO1L1P SMG6 SNORD115-41 SRGNP1 ITGA11 WWTR1 COBL SNORD17 NFX1 MDN1 CIT ZNF607 HERC4 ATP6V0D2 HTR4-IT1 ARHGAP6 MCM3AP KCTD7 ST3GAL3 MIR4307 PCDHA1 TTC6 TCF7L2 APCDD1L-AS1 TYW1B SETD7 hsa-mir-3171 CHAF1B QSER1 RN7SKP96 KIAA1328 Y_RNA RNU6-286P OFD1P13Y PCDHA8 GBA3 LINC00298 RN7SKP238 RPGR CES5A OVCH1 CATSPER2 TMEFF1 GPR125 CLDN8 STARD9 HSD17B12 NDUFA9 LINC00537 NES EGLN3 HS6ST3 FKSG68 CUX1 RNU3P3 MUC5AC GPM6B CLEC4A LINC01010 RN7SL456P PRR12 ANK3 SNORD74 TIMP2 CDH12 ZMAT4 LINC00911 TECRL SNX24 OR11A1 UPP2-IT1 TMEM132B FRMD6-AS2 CPSF1P1 SLC25A15P5 PTPLAD2 ENPP7P10 SAMD9 FLG-AS1 SV2C HNRNPKP3 ZNF490 AKR1C3 FGFR3P5 GMDS TPTE2P3 CACNA2D4 LHFPL3 SPARC RGPD1 C14orf144 WDR52 CNIH3 PLEKHM2 ANKRD62P1-PARP4P3 ASB17 RNU1-33P

		FEZ2 THSD4 CDC5L SLC9B1 EFCAB1 RPS24P16 KPNB1 LRRC37A7P TAS2R38 FOXO3 TRIM59 NF1P3 HIP1 RELN MRPS31P2 LINC00284 RASGRF2 ST7 TRPS1 HS3ST4 RPS17P14 RNU6-1193P PCNXL2 MTUS2-AS2 TMEM101 NF2 ADAMTSL3 EVA1C SNHG14 PCDHGA6 NXPH1 OFD1P12Y DDX3X PHF2P2 LINC00871 MYO18B IGLV4-69 CDH4 ARHGEF10 RNU6-410P SERPINB12 CASP12 GABRA3 E2F3 FRG2B FGD6 TFF3 MIR603 TNR LINC00687 VDAC1P1 ADCY9 DPP10 OCA2 CELF4 PPP4R4 PRKCQ-AS1 CDKAL1 VAV3 RMI2 ARHGAP22 MROH1 ULK4P2 NPFFR1 NPC1 PCDHGB1 KCTD9P1 INPP5A RPL12L3 C17orf51 SMARCE1 VRK1 MIR649 ZNF678 CNTN6 MLLT10P1 CXorf30 TMEM106B TRAP1 ACSM2B SMEK2 SCN9A ZNF420 CDK2AP2P2 SCARNA17 APP ATP2B1 ATF7IP2 MS4A14 BNIP3P2 C6 KCTD9P2 ZNRF1 SERHL MAMLD1 ATRN FBLN5 HAPLN1 MIR3152 CCDC88A MIR551B CHKB-CPT1B C10orf11 ARNT2 KCTD1 MIR663B C5orf17 SPEF2 MIR3118-1 CXorf22 H2AFY APBA1 ANKRD20A7P RAB2A SNORD115-11 PLCE1 CEACAMP6 IGHV1OR15-4 GRAP2 MUM1L1 ATP5BP1 WASF1 HNRNPA1P71 ANKRD36B CUBN ADAM12 RNU6-1280P FAM214A DNAJA1P5 NT5DC1 CUBNP3 RNU4-82P AMZ2 FRAS1 SIDT1 ATP9B TPRG1L PEBP4 ASNSP1 IGF2BP3 CACNA2D1 DHX35 DEC1 PON3 RPS15AP34 TMCC3 RN7SL143P HCN1 TCL6 SAMSN1-AS1 CD2BP2 PAPOLA TOP3A RNU6-10P RAB3IP CEACAM6 CHRM5 SPECC1L ZNF208 TET3 CASP6 LINC00693 KIAA1009 GCNT1P1 ZNF582-AS1 NOC4L PIGFP2 FRY MNS1 MSANTD2P1 ZNF718 PTPN20B DKK2 FAM209A PCDHAC1 TANGO2 CCDC64 HERC2 GGT2 LRFN5 NASP PIK3R5 CTBP2P8 PALLD TRAV24 TENM4 AGBL4 CPNE5 GZMAP1 CLTA PARP16 DUX4L18 A1CF ASB13 SLC6A10P hsa-mir-490 IPO7P2 RHOC PPP3CA CHRNA5 RNU6-1291P FAM63B TSPAN13 GHR SPTSSA RNU6-311P SPIRE2 DUX4 FTH1P27 BCLAF1 MIR3118-6 LINC00374 NKD1 ARHGAP39 AKR1CL1 HTR7 KIAA1715 ZNF345 RIPK4 MYL12A WDR59 CCDC57 TRUB2 LINC00656 CEP170 RPS15AP3 RASGRF1 LINC00440 RIN2 FRMD5 MAGI1-IT1 MIR99A PTK2 RNU6-1132P RNF217 UBR3 RNU6-1225P OTUD4 LLGL2 RBFOX1 KIR2DL4 STARD13 TMEM120B PCDH7 SCP2 RNA5-8SP2 LRRC4C USP17L25 ASUN ABHD2 DENND1A ALCAM MZT1 INTS4L1 PDZRN3 C1orf168 ZNF41 AVEN TMEM117 TPTE2P2 DNMT3B GUCY2C ADAMTS16 TASP1 NIPBL PPP2R5A LINC01090 FYB SLC17A3 MICU1 EBPL IGHVII-62-1 BDH2 HNRNPA3P14 ZNF114P1 CNN2P7 DEPDC5 DUX4L6 RGL1 MIR3198-1 ATG10 MBD5 LRRK2 NKAIN3 KIAA0355 MIR548AX CPEB1 INO80D PCDHGA1 ZNF728 DYNC1H1 PALM2 LSM12 EPHA1-AS1 RNU6-316P CD300A VASH1 SLC35A5 PKHD1 MTND2P28 MEG8 MIR1281 TTLL11 NEBL DIDO1 CCDC18 COG3 DIRC1 CBFA2T2 MARK3 DHX15 AGGF1 PRRG1 DUX4L11 LINC00993 ITPRIPL2 ADAMTS2 PKIB TCF4 NR2C2 MYH1 MGST1 NAALADL2 RBM44 FRYL ZNF432 SPG11 VN1R4 AP3B1 C6orf183 RARRES2P4 FUBP1 KIAA0586 STON1 CPAMD8 FOCAD TIAM1 PCA3 OR7E83P MMP20 PLEKHM1P PRG3 KRT43P MIR4533 MLIP DDX24 OR5H8P ATP1A1 MIR487A EML5 C5orf47 SORCS2 PRIM2 PGM2L1 RNU4-59P STRC BCL2L15 RN7SL646P LIPI OR4H6P YIPF6 DISC1 DZANK1 RN7SL141P ALG6 PPL LRTM1 MAP3K7CL FMN1 ANKRD20A1 RALGPS1 DNM1P51 MIR485 TMEM212 PMS2P11 CHAF1A ANKRD62P1 PHF21B TRAV6 ZFPM2 SEC63 TDRD10 GC PIEZO2 SLC35F1 RPL9P28 TOR1AIP1 ADC SNTG2 PXN SVEP1 TNF C21orf49 NTM ASAP1 RNU4-40P PCBP3 BFSP2 LINC00971 FRMPD4 ATP8B4 LMCD1-AS1 COL23A1 NSD1 TMEM179 LINC00639 DSCR10 FYN FAM3B KCND3 ITGB1 KIR3DP1 RIMBP2 TTC12 PRMT8 CSPP1 IFT43 CCDC169-SOHLH2 XRCC4 RABGEF1 LRBA MEMO1 DCLK2 GAB4 PRELID1P2 RNU6-617P MAP7 PPOX CRCP C7orf49 TEX9 RAD54B MIR1911 NSG2 LRR1 MIR3687 OR8A2P MSANTD3 LINC00842 ZNF177 DGCR5 MCFD2 IGKV2-36 GRIA1 POM121L9P ZNF627 RNU2-42P KRT8P15 RN7SL204P RN7SL194P SPIDR C17orf80 UBA6-AS1 ANKHD1-EIF4EBP3 SNORD115-23 NAV2 MIR183 EFCAB5 STK3 CHN2 ZNF781 VN1R53P CNOT7 FSHR POTEKP NBEAP3 MARCH3 BMP15 RUNX1T1 SGK2 RNVU1-17 MSR1 SNORD115-40 LINC00380 PSIP1 NOL11 SPRR4 USP18 SLC30A7 SMCHD1 MED15P7 NRG1-IT1 NET1 PCDH15 ESR1 GCNT2 ARHGAP12 ZNF767 SLCO1B1 KSR1P1 ZNF790 SGCZ CCDC176 ICT1 EDA2R SEL1L2 PLCXD3 MIR889 FAM107B LUZP2 DENND2A SIK2 APTX GRM1 SCFD1 TRPC4 MTND1P31 CNTN5 ERC2 PRKACB ZNF705A GNG2 RN7SL662P PCBD2 TRANK1 RIMS1 UBE2K CPB1 MIR4798 RRH BCAR3 DRP2 RNU7-176P PDZD7 POR ENPP2 FHL5 TRIM60P13 RPS27P16 DOCK4 KCNMB3P1 VPS53 SLC5A3 TRIM51 LINC00382 TMPRSS4-AS1 IGHV3-41 OR4K4P ATP6V1E1 FRMD4A MCTP2 SNORD115-38 CERS3 LMX1A PIGK SLC25A15P4 PCDHA7 WWOX PTPN9 ABCA12 PLOD2 TTC27 FIGN PCSK2 KCNIP1 TMEM178A ABT1 ARID2 CD86 MIR670 MARK2P8 RNA5SP300 NMD3P1 ZNF33BP1 B4GALT4 FAM228B BMS1P16 RNU6-78P LINC01043 KRTAP19-10P CNKSR2 DNM3-IT1 GFRA2 FAM104A HMGB3P20 SNX29 SNURF REXO1L11P BEND5 COL8A1 ATXN3L EPHB1 BMS1P18 FAM85B COLEC12 LPHN3 LINC00478 LSAMP FAM90A22P MIR548F1 WFDC9 KCNQ1 NR2F2 CTTNBP2 C2orf27B C14orf164 FHOD3 PSG7 IGHVIII-67-3 LINC01088 PARP8 ZNF215 TEAD4 RNU6-725P EFEMP1 TNRC6B RNA5SP186 PIGB SRRM1 NME9 RN7SL321P ZNF608 IGSF21 RNU6-250P SLC38A6 SLC9B1P1 GLCCI1 LNX1-AS1 RASSF8-AS1 DUSP23 MIPEP HRNR IGKJ4 ABCC9 UCHL3 KLHDC8A IPO8P1 BPIFB4 MRPL42P4 RWDD2B ARHGAP20 INSL6 DOCK1 SERPINA2P NR3C2 MIR134 INPP5F SNORD115-47 CRADD SHC2 MTUS2 VASH2 WWTR1-IT1 DIAPH3 OR8U1 TPTE2P1 PTTG1IP COL9A1 ADARB1 GGT1 ZNF664 TMEM178B HAS3 SOD1P3 OR10Z1 TTC40 ARHGAP32 SLC27A6 SLC7A13 TRPM7 CGNL1 FLT1 PPP2R4 REXO1L9P LINC00276 CDRT1 ANKUB1 RN7SKP6 LINC00032 EFNA5 NOL4 LINC00536 MIR4634 GTF2IP3 ISM1-AS1 NXN ENPP7P5 CNTLN ASL C1orf173 NEK5 AMY1A PCDHA5 PM20D1 BMS1P10 CMSS1 SLC22A23 PHC1 LINC01049 DSCAM-AS1 PLCH1 CACNA1C-IT3 NAMPT PCNXL4 PCDHGB7 C14orf39 TLN2 ZNF493 BPESC1 HDAC4 RNASE13 GLRA1 TRAV33 MIR376A1 STK36 RNU4-24P FAAH2 ZNF717 CTNNBIP1 TRPC5 TEP1 SRRM1P2 FAM90A20P YAF2 WDR95P KIAA1644 SMPD4P2 CT49 HNRNPUP1 MRM1 ATP9A F11-AS1 HNRNPA2B1 RNF216 NHLRC2 DNM1P50 ZNF430 ZNF793 FTO MGC4294 USP46 DMXL2 STIM2 POTEF SNORD23 RNU6-1327P SNORD54 ADORA2A-AS1 C8orf46 RNA5SP492 EIF3A C10ORF68 HS1BP3-IT1 SEC24B CASC15 PPFIA2 FRG1 ADAM28 IGSF11-AS1 SDHAF2 ZNF268 ENOX1 KRTAP4-11 RN7SKP60 UNC5C DNM1P28 AKAP6 MIR648 ACSBG1 ANKRD36C NKAIN1P1 ANKRD26P4 CSGALNACT1 IZUMO3 CSMD2 RNU6-1005P PTPN20A KRTAP20-1 SMAD1 ARHGEF15 OR1E3 ZNRF3-IT1 LST3 SORCS3 LINC00523 RPL21P6 ANKHD1 VPS37A KRTAP12-4 LINC00189 LINC00299 LARS2-AS1 POU6F2 TTC8 BRD7 FCF1P10 SNORD6 TENM3 RNU6-280P LINGO2 OPCML KRTAP27-1 DIAPH2-AS1 SFI1 GNE OR52N5 RBMX2P1 TSPEAR-AS1 HMG20A CEP85L ATF2 ARL6IP5 TUSC3 COPG2 RNU4-60P PLSCR1 PAPPA-AS1 RNU6-156P PHACTR2 CD163L1 ZNF72P ZNF880 NOX5 C1orf167 EEF1A1P1 RNA5SP283 KCNJ12 OR51AB1P ATP5J SRP54 STARD4 DNAAF2 TAS1R2 MTAP ADORA3 COL6A5 HYAL4 BACE2-IT1 SP2 TMEM38B CCDC91 PDE7A MMP26 CALN1 LRP2 CYBB UXS1 SPTA1 ZNF483 SEMA6D KCNJ3 ZNF573 PCDHGA8 RALGPS2 RGL2 SNRK FAM27E4 PMPCB DHRS4L2 MACC1 OR4C10P CXorf21 IL17RA YTHDF2 MIR548AS LHFP CAMK4 SERPINA9 RN7SL177P GALNT14 NPLOC4 TMEM189-UBE2V1 IGLVIV-65 RNU6-721P DKFZP761J1410 LINC00466 MZF1 VWC2 GUCY2F ANKRD20A17P IGHVII-40-1 COL16A1 KRTAP6-1 CELF2 ATP8A2P1 SMIM2-AS1 TP53I11 CACNA1C-IT1 SHOC2 PDXDC1 NTNG1 HHLA1 ZNF606 ADTRP RBFOX2 DDX10 MIR5095 IGLV2-8 SNORD116-18 FBXL7 IL1RN ZNF26 PCDH17 TFPI USP17L9P MIR323B ABCA9 SMTN TMEM108 CA3 PQLC1 GABRB3 TPTE BFSP1 CKS1BP5 LRRC20 DSCR9 MIR3182 LPPR1 LCP2 RMRPP4 SEZ6L NAP1L4P3 GRM7 SLC39A8 PET117 RNU7-144P IGHV3-29 SMG7 MX1 ZNF564 PAXBP1 ANKRD13A PLGRKT ZCCHC11 COPS8P3 RNU6-288P RNA5SP219 KIAA1211 IQSEC3 ZNF782 TGFBR3 RNU6-772P PPA2 MAP3K7 UPRT AKR1C1 ZNF615 MIPOL1 GTF2I DNAH3 C16orf80 C15orf32 SLC7A1 PTPRM ATXN1 SSPN KIRREL3 ADAM17 USP36 CHRNA3 OR6C68 TMEM186 MIR548W AK5 ELAVL1 BNIP3L IGKV1OR-1 PRKCH HAPLN3 GALNT8 PCDHGB6 NRXN3 RFPL4AP7 OR7D1P CYP2AC1P OR6N1 RHPN2 ANKRD30A FSBP DLC1 HMGN3P1 C9orf40 ASXL1 KCNH7 NUAK2 KRTAP15-1 LCE2C NUDT4 SNORA70C RNU1-104P CLDN1 PCTP TSNAX-DISC1 ABCC1 FOXD4L5 WFDC8 IGHV7-56 C14orf64 NSG1 PLEKHG1 RBAK-RBAKDN ANKRD20A8P GABBR2 WDR83OS BCRP1 KCND2 WIPF1 C21orf90 PTPRU LY86-AS1

		ATP8A2 NHSL2 SNTB1 SLC24A3 TGFB1I1 FAM228A SOBP TSGA13 SHOX2 NPY4R RNU6-530P MIR381 EGLN3-AS1 BMS1P15 ZNF207 MPRIP KLF13 RN7SL153P RNASE12 RN7SKP100 NMNAT1P4 IGHVIII-67-2 GRIA4 ANKRD32 CERS6 SCMH1 VPS26AP1 TMPRSS12 USP32P1 TPH2 WDYHV1 SNORD115-10 KIAA1598 MORF4L1 CNNM2 APC LOXHD1 FAM108A8P ELK3 ZNF233 OLFM3 INADL RRP15 GPR139 LINC00508 COX5BP6 ADNP2 OR5AK4P MACROD2 IL18R1 SGK3 BNIP3P7 FAM90A21P INO80 NAA11 TMTC1 TSHR TIMD4 CCDC7 OR4A43P RGS7BP FSD1L EPHB2 LPHN2 STON1-GTF2A1L MTND6P4 COL27A1 PHLDB2 PLAUR BMS1P17 RNU6-1293P TMEM56 TEX11 SCAF4 SYT16 PRR9 MTND5P14 RALBP1 IGHV3-47 TMC7 RNU6-16P ZCWPW2 FBXL13 KAT7 HNRNPA1L2 IFI27L2 MIR376C MYBPC2 ZNF235 ACSL4 CAPZB SATB1 ERC1 IGHV4-31 PPM1D OR10N1P ZNF850 ST20-MTHFS NXPH2 ROS1 MACC1-AS1 PCDHA6 EFCAB11 SLC5A4 NRG1-IT2 DGCR10 AGO3 SLC6A17 STT3B RNA5SP405 ZBED5 MAGEB2 CKAP4 SLFN12L CCDC13 MOB3B SNORA16 PGK1 RNASEH1 WLS MIR656 ANKS1A RPL10P3 RYR3 PRCP RNF135 NBAS MTND1P17 RN7SKP86 ZPLD1 NBN PRTG RNA5-8SP6 C12orf42 ADAMTS18 SEPT14 SOS1 MIR767 EHF RNA5SP20 CTNND2 DNAH14 MAST2 COL22A1 RPS15AP1 RNU6-1320P TC2N SETD2 KCNQ5-IT1 RASSF3 KRTAP9-3 RNU6-494P SNORD116-19 DCK SYNE2 ENPP7P2 RNU6-954P SQRDL BAI3 DOCK2 TRIM23 DPYSL2 SDCCAG8 EXOC5 MIR381HG RN7SL17P OR4Q1P SLCO1B3 FLVCR1 PTPDC1 RAB3C PPM1J CEP135 NRP1 TRIM22 CDH13 UBE2E3 MDGA2 SAMD3 METTL8 EPN2-AS1 VEPH1 RFC3 FAM129A OOSP1P2 BCAN AKAP7 RNU6-953P CROCC ASCC3 OR4C7P TGM2 STAM MIR4535 ACYP2 MARCH10 ARHGEF7 MIR382 PHACTR3 C7orf69 ZNF277 7SK ANKRD26P1 ZNF879 SAMD12 DACH1 PTPN11 PHKA2 TRDN RNU6-1066P KRTAP4-3 APOL6 KRT39 ZNF397 MIR5704 MIR1276 DAB1 DPRXP4 LINC00442 DICER1 ZNF148 TEKT4P2 ZNF141 PCDHA4 CHODL-AS1 SNTG1 SULT1A4 GGT8P C5orf38 THOC2 UBQLN4 TAF1B LINC00898 LINC00906 COL15A1 ALK IGHV3-63 EXOC6B PPP1R26P2 IGKV1OR2-2 EVC2 LINC01019 GREB1 FAM27E3 ADAT2 REEP3 TCAM1P BDNF MIR320B2 LDLRAD4 CHMP1B2P EIF4EBP3 OR51F1 ACBD6 REST DNAH6 REXO1L3P DSCR4-IT1 MGAT5 TSPAN8 IGHVII-44-2 MRPS11 C2orf27A MYO5B STOML1 MAEL SDR42E1 LINC01057 RNU6-127P RNU6-469P SNORD109B RNA5SP465 TMEM233 BDKRB2 RNF180 TAS2R41 H3F3AP4 DUX4L5 RNF215 NDST4 PACRGL MIR543 ADORA2A MAGI3 FAM90A23P CDKN2B-AS1 STIM1 TSEN15 MIR521-2 CCDC122 SYPL2 COL21A1 TUBA3C FAM135B BACH1-IT3 TSC22D3 KIF16B SWT1 CASP8 NRIP1 IGLJ1 CDH2 MROH7 RNU6-26P ARF1 FLNC DPYD-IT1 SIPA1L2 PLCH2 CCNG2 RCAN2 LRRC69 CASC18 TENM2 TMEM261 MTMR8 RPL31P3 DSG2 PPIAP22 CEA TANC1 JAG1 CNN3 MIR105-2 CACNA1B PAPPA AKNAD1 FAM27A IGHVII-65-1 VPS41 IFT80 BMS1P13 PSMG2 LINC00879 AKR1C2 MIR3118-4 RN7SL373P HSPA4L RPL21P11 POU5F1P6 FAM208B HDAC9 METTL15 OR5J7P CUZD1 CPB2 UHMK1 ELOVL7 LINC00351 CHST13 GOLGA1 ANKRD20A18P VDAC2P1 NR3C1 PPIAP6 NEK7 MIR300 GPR133 FSTL4 FAM108A10P PFN2 CDCA7L MTOR FAM222B MCM3 KCNB2 SLC19A3 C17orf75 ZNF585B RNU6-690P CYP3A54P CCDC170 STK38L RN7SKP197 HNRNPA1P40 RALGAPA2 CBLB RORB FAF1 KIAA1407 CDK6 LARGE VPS39 VAPB GABRB1 SNAP25 LUC7L ABCD1P5 SGSM1 TPTEP1 ST8SIA1 JAKMIP1 SH3KBP1 PCMTD1P1 RIMS3 SIRPB1 RTFDC1 MIR4290 MIR410 RPL4P2 FHL2 CDC42BPG HIGD1A LTB LINC01101 OR4A41P NEU3 ZFPM1 BZW1 PIP5K1A GDAP1 OR51H2P RPL8P2 RNA5SP222 ZRANB1 TPTE2P5 DISP1 PRB2 HS6ST2 MYCT1 CPT1B SCGN KYNU MS4A5 GPN3 RNU6-21P TFAP2D ZFP30 MTND5P5 RN7SKP101 SPATA6 BEND7 CLVS2 CYP7B1 RN7SL609P STXBP5 FAM212B TBXAS1 KXD1 OSBPL9P4 USP25 OR2BH1P RN7SL674P BMS1P14 RN7SKP139 SLC44A1 FCRL2 FAM196B H3F3C SPRED1 BMS1P9 NPR3 SIPA1L3 MROH2B LIPA FAM220A RNU6-1269P TMC1 CNTNAP4 TTLL11-IT1 MRPS22 TSPAN33 TTC32 LINC00393 CDH26 AGPAT4 NOX4 IGKV1-37 PDS5B CES1P2 LRFN2 FLNB RNU6-1049P UBE2Q2P11 RNU6-157P SCAI MEGF9 MIR5190 SNORA32 RNU1-11P PGM5P2 SNUPN NCOR1P3 KRT18P31 POTEA PCDHGA9 NAPEPLD MIR3648 GCNT7 OBSCN SPECC1 FAM155A FCF1P9 DPY19L2 GACAT1 FTLP15 C20orf196 NCOR1P2 CYYR1 IGKV2OR2-1 IL1RAP LINC00559 KIR3DL1 PTPRT MRPL3P1 FAM118A TAF4 SETD5-AS1 TRIM9 KY IGHV3-48 WRAP73 CSMD1 SNORA38 PLEKHH2 RPS3AP1 CENPBD1P1 SLC24A2 SNORD115-24 GLI3 LRRTM1 VAV2 MTND5P11 FAM90A7P SPRR1A LRRC9 SCEL SNX20 GRIA3 RNA5SP488 ILF3 RNU6-400P RP2 NTRK3 ZFRP1 RXFP1 OR5E1P LINC00457 DPRXP5 MIAT FBN1 SGCG SLC35F3 DCDC2 NANP ZNF621 ANKRD20A12P SEMA4B DYNC1I2 NIPAL2 LONP2 RAB31 AGGF1P4 DHRS3 LINC00418 GAB2 CEP89 VPS13D YRDCP3 DIS3L2 FREM2 ZNF829 BMPER hsa-mir-6723 NOS2P3 SNTB2 LINC00922 SLC7A8 ANKRD31 TMEM41B MYO5BP3 FAM149B1 S100A11 SPATA7 EFHC2 DOT1L RNU7-35P ZBTB8A PDE1A SNORD113-2 NFAM1 OR10R2 MS4A6E TMEM194B LINC01036 ONECUT3 ZMAT3 IPPKP1 ATF7IP OR4H12P VIPR2 GMEB2 HMGA2 B3GALTL MIR1185-2 HHAT MTATP6P1 TMPRSS15 LINC00632 ZNF558 MLLT10P2 MX2 RPA3-AS1 FAM227B THSD7B EFCAB4B PSPC1P2 UBE2G2 KIAA0040 RN7SL683P HLCS-IT1 KRT2 ZNF618 RBKS DNAJC6 CLOCK DSG4 CPA6 MIR105-1 U3 CPNE8 DEFA3 PGM2 LINC00521 M1AP AGBL4-IT1 NUS1P2 WBP1L RXRG DNAH10OS KIR2DS4 H2BFM MIR3118-5 PCDHGA2 TBC1D4 SYT1 OR4K1 MSL2 MOXD1 FAM13C OR5AO1P ASXL3 HNRNPA1P68 KCNT2 DHX32 SCAPER GZMH DENND5A CRYBB2P1 CDK2AP2P3 RNU5A-5P OR4K13 MED4 RNF220 PTPRS NPHP4 PNPT1P1 IKZF2 TNC DOCK9 LINC00856 IGHV3-32 PML DLG2 FAM101A SACS TTLL12 RBCK1 AFF2 MIR650 IGHV3-75 NCKAP5 THAP7-AS1 RN7SL552P MGLL FAM27E2 FAM83G SCFD2 PTPRD RORA RNA5-8SP5 CYP4X1 IL2RA TRAPPC12 STXBP5L REXO1L2P SHISA6 FAP NSF ANKRD20A2 NFASC OR52U1P TPT1P5 LINC01141 ERICH2 HDX SCPEP1 IGKV2OR22-4 RN7SKP85 METTL9 ATP5O snoMe28S-Am2634 RBAKDN LINC00200 MAP2K5 PCDHGB8P RPS20 SEPP1 CPEB2 LINC00885 LOXL2 IGHV1OR15-3 TMEM56-RWDD3 ATAD2 BPTF ZNF229 RPS10P7 LRRK1 NPEPPS SLC2A12 GRIK1-AS2 RNA5SP221 DNMT1 PIWIL4 MRGPRG ZNF571 PRKG1 UBFD1P1 ZNF283 PLXDC2 CTBP2P1 ELAVL4 SNORD116-16 CADM3 PIP5K1P2 KRTAP9-8 KIR2DL3 FOXRED2 IGLV2-18 GLTSCR1 AK4 DGUOK-AS1 KREMEN1 SNORD116-26 FMNL3 SNAP23 STXBP6 ANXA8L1 WDR25 CYP4Z1 CHST9 DMBT1 LINC00664 MXI1 SPTLC2 USP17L26 TTC28 MTURN FLT3 GALK2 NELL1 GRAMD4P5 GYG1P1 CEP97 SNX18P15 ANKRD30B PCDH19 SRIP1 ADORA1 MIR514A1 STX12 VN1R87P MRPL39 OR11H13P MEG9 FAM46A SNORD27 RNU6-368P HPCAL1 MCM9 PTGR1 RNU6-405P KIAA1671 PXDNL ST7-OT4 EDA SAP18 ASIC2 FIP1L1 RALA DOCK10 SPCS2P4 IGDCC4 PRB1 EDDM3A CTXN2 RPS26P30 OR11K2P CACNG3 COL2A1 RPA1 CNTNAP3 PPP4R1L PPIAP14 FNDC3A MIR4760 NAP1L4P1 LINC00617 KHDRBS3 DNAJC2 MYO5BP1 TPK1 MICAL2 PRKD1 ATP8A1 TNFAIP8 KIAA0195 MTHFS ATXN8OS CALCRL PTP4A1P1 RNU1-150P ABAT RPS24P12 SLC22A25 EGFL6 BRSK2 LGALS9 SCARNA21 MIR495 DRD5P2 MICAL3 ADK CELSR1 TNNI3K HDAC2 THBS2 LINC00271 RANBP17 TPO ETS1 MRPS27 MLST8 TVP23C-CDRT4 TMEM138 ADAT3 C9orf131 TMEM131 GTDC1 RNU6-230P TRIM51CP OR8K5 RNA5SP497 RNASE11 MIR4480 BICC1 CRNN DISC1-IT1 SCAMP1 KCNK13 SEMA3D BANP MTND2P4 CCDC169 DUX4L4 DDC MIR548AL RNU6-614P WFDC11 LYPD5 RN7SKP147 HNRNPA1P53 COX6A1P3 FLNB-AS1 FAT3 OR1AA1P NPSR1 CAB39 ENPP7P1 SNORA25 POTEH-AS1 OR11G2 RAD23BLP WSCD2 SERTM1 PCDHA3 SNORA70 MTMR2 AAGAB YWHAQP9 RRM1 MFSD12 OR52B4 CTDSPL PDE11A POLR3H TSPAN9 THUMPD1 SYCP2 CLYBL ACIN1 USP32P3 PCCA RAB30 TMEM163 CEP41 MS4A7 CLVS1 ATF6 PRKAR1B LINC00972 FAM189A1 VCL IQCJ ZSWIM7 DEPTOR CCDC88C B4GALT3 NVL RNA5SP490 ATAT1 TMEM220 PPFIBP2 EDARADD CENPV CREBRF DTX2P1-UPK3BP1-PMS2P11 RNU6-1239P CTBP2 ZMYND11 MYH8 SNORD113-1 RGS6 SRGAP3 ZNF525 RNF144A-AS1 EFCAB2 PGM5P1 SNX19P3 AKAP13 CACNA1C-IT2 MIR548X ZNF337 OXSR1 RAD51D MIR369 SARDH CUL2 RNASET2 CCDC146 MIR4273 MYRIP SLC39A11 LINC00343 SCNN1A IGKV1OR22-5 KCNE2 UNC13C ZNF443 DUX4L14 OR5V1 RIMS2 KRTAP10-10 TMEM55A IGHVII-28-1 SYT17 RNU6-898P CCDC171 ACTA2-AS1 FOXP1 MAS1LP1 SLC5A1 SNORA80 C16orf95 DNAJC3-AS1 SNRPD3 STAC RBMX2P3 OR4M1 SCN8A IGHVIII-25-1 FRG1B MIR4300 KCTD16 PNLIPRP1 EYA4 DEFB122 PARP4P3 KSR2

		TMTC4 L3MBTL3 NOVA1-AS1 MROH7-TTC4 ARL15 SGOL2 IGHV3OR16-12 HIVEP3 SPATA13 ZNF736 CEP152 CLIP1 RNA5SP518 RARRES2P2 RUSC1-AS1 SEMA5A GUSBP6 MORN2 5S_rRNA GAREM PRDM15 PLAC4 BLOC1S6 MTND4P14 BCL11A RFX4 RPL18AP14 EIF3FP1 ADAM20 LRRC16A SNORD115-45 AGAP1 FREM1 ATP6V1D SAA2 LAMA4 SNORD115-19 PMEPA1 C1orf94 DCC IGHV4-55 ZZZ3 ZNF112 RN7SL163P MIR548Q UBE3C CTNNA2 RPSAP55 PEX5L CYP46A1 TERF1P1 CYB561A3 IGHV1OR15-2 PARD3B CHN1 FAM90A15P UCHL1-AS1 PRAMEF12 RNU6-249P HGSNAT RAG2 LCE2A GATAD2B FMNL2 RNU6-540P ANXA4 IGKV3OR22-2 PARD3 CEP112 snoU13 MYOM3 CCDC73 OR8S1 SYNPR-AS1 NPL FAM110B NRG1 CAST SNORD115-34 CES1P1 LGR4 GAB1 SYT9 SULT1B1 ZNF845 DGCR2 DHRS4-AS1 LINC00534 VSIG10 TRDC RPS20P1 DLEU1 JPH3 SLC38A7 CYP4F29P GPR39 SLC22A10 RNF128 PXK HSPG2 SLC25A48 CP FAM160A1 SUCLA2 BTF3P10 ATP10B SLC35F4 NPAS3 ZNF562 SPRR2E DNAH5 KHDC1 DNAJA1P4 PGPEP1 MLTK CCNYL2 FMN2 SEMA3C GRAMD3 WDR93 CACNA1I DPY19L1 RPS12P21 C8orf44 DGKZP1 RNA5SP280 NUSAP1 PCNT BCKDHB PAH OR4K11P LINC00940 DCLRE1C HBG1 ADRBK2 C3orf67 LINC00353 ZNF622 SELO SNORD115-39 TOP1 TRDV3 EXOC6 SNORD115-25 RN7SKP199 SHISA5 MPST

Table S11. Genes that increased their contacts with rDNA after heat shock treatment specify TF, that exhibit co-occurrence at a set of 60–70 genes. Related to Venn Diagram in Figure 4C.

Term	Overlap	Adjusted P-value	Genes
POU6F2	70/299	0.00000011546621302894669071466906022302434386972436186624690890312194824218 75	ROBO2;CNTNAP2;RYR2;TENM1;CSRNP3;THRB;CLSTN2;ZBTB20;PRDM1;AFF3;ADARB2;SLC8A1;IGF1R;ROBO1;GRM8;PHACTR1;SH3GL2;EPHA5;CADPS2;GRID2;ADGRV1;GRID1;PDE4D;MAGI2;CACNA2D3;SORCS1;PROX1;SYN3;FOXP2;KIAA1217;GAP43;TCERG1L;NAV3;SETBP1;RARB;KCNQ5;PLCB1;CREB5;NCALD;FSTL5;CUX2;ERBB4;PLXNA2;SPOCK1;CTNNA3;GPC5;CSMD3;GPC6;NTRK2;NEGR1;AUTS2;CADM2;ZBTB16;KCNIP4;CADPS;ESRRG;SORBS2;BTBD11;SULF1;GRIN2B;PBX1;DCLK1;MGAT4C;IGSF11;SDK1;NFIA;PPP2R2B;SMOC1;CNTN4;ADGRL3
FOXP2	66/299	0.000000178779033569847485027695405818137786013721779454499483108520507 8125	ROBO2;CNTNAP2;TENM1;CSRNP3;THRB;BNC2;COL14A1;COL12A1;ZBTB20;PRDM1;AFF3;SLC4A4;SLC8A1;IGF1R;ROBO1;CCND2;HMCN1;EDIL3;EPHA5;CADPS2;DST;PDE4D;MTUS1;CACNA2D3;MITF;LMO7;PROX1;DCN;RUNX2;KIAA1217;PLCB4;ZEB1;SETBP1;MPPED2;RARB;PLCB1;ATF3;SAMD5;FBN2;HLF;EPAS1;PIK3R1;NCALD;STOX2;ERBB4;PLXNA2;GPC6;ABCA1;NTRK2;NEGR1;AUTS2;CADM2;ZBTB16;FN1;PBX3;ESRRG;SORBS2;SULF1;FMO4;PBX1;DCLK1;SDK1;NFIA;CNTN4;BMPR1B;ADGRL3
ZNF804B	67/299	0.000000105702698500669214722361664848815365758127882145345211029052734 375	ROBO2;CNTNAP2;RYR2;TENM1;PTPRR;DOCK3;THRB;CLSTN2;COL14A1;AFF3;SLC8A1;ROBO1;GRM5;GRM8;PDE4B;FAM19A1;PHACTR1;HMCN1;EDIL3;SH3GL2;EYS;RGS7;EPHA5;CADPS2;GRID2;KCNH5;ADGRV1;GRID1;PDE4D;MAGI2;CACNA2D3;SORCS1;FOXP2;KIAA1217;NAV3;SETBP1;DPYD;KCNQ3;KCNQ5;PLCB1;ASTN2;RAPGEF4;ADCY8;FHIT;FSTL5;ERBB4;CTNNA3;GPC5;CSMD3;GPC6;BRINP3;NEGR1;AUTS2;CADM2;KCNIP4;CADPS;ESRRG;SORBS2;SULF1;USH2A;GRIN2B;OXR1;MGAT4C;SDK1;CNTN4;GALNTL6;ADGRL3
ESRRG	65/299	0.000000266920901064186131065739462919772506666049594059586524963378906 25	ROBO2;CNTNAP2;RYR2;THRB;ZBTB20;AFF3;SLC4A4;SLC8A1;IGF1R;ROBO1;PDE4B;PDK4;SH3GL2;CADPS2;ADGRV1;PDE4D;MAGI2;MTUS1;CACNA2D3;LMO7;SORCS1;PROX1;FOXP2;DNM3;KIAA1217;PLCB4;MPPED2;RARB;KCNQ5;PLCB1;SGK1;ATF3;RAPGEF4;CREB5;GAS7;HLF;RTN1;SLC1A2;ITPR1;PIK3R1;NCALD;ERBB4;PLXNA2;CTNNA3;GPC6;NTRK2;NEGR1;AUTS2;ANGPT1;CADM2;ZBTB16;CADPS;FN1;SORBS2;BTBD11;SULF1;FMO4;PBX1;DCLK1;SDK1;NFIA;CNTN4;BMPR1B;RETREG1;ADGRL3
MYT1L	65/299	0.000000266920901064186131065739462919772506666049594059586524963378906 25	ROBO2;CNTNAP2;RYR2;TENM1;CSRNP3;CLSTN2;PTEN;ZBTB20;AFF3;ADARB2;SLC8A1;ROBO1;RPH3A;GRM5;KIF5A;ENC1;PHACTR1;DIP2C;SH3GL2;RGS7;EPHA5;CADPS2;UNC13A;PRKCB;SGIP1;PDE4D;DYRK1A;CACNA2D3;SYN2;FOXP2;GAP43;NAV3;TBL1XR1;DCX;KCNQ3;WDFY3;PLCB1;RAPGEF4;GAS7;KMT2E;RTN1;ANKRD11;SLC1A2;ITPR1;CACNA1D;NCALD;PLXNA2;SPOCK1;CSMD3;NTRK2;NEGR1;AUTS2;KCNB1;CADM2;CADPS;ESRRG;SORBS2;GRIN2B;PBX1;DCLK1;SNAP91;GRIN1;SDK1;NFIA;PPP2R2B
ZFAT	62/299	0.00000140581072351939826277312725610890709049272118136286735534667968 75	ZFAND3;ZMYND8;WWC1;ZBTB20;PTPRK;PRDM1;AFF3;SLC8A1;IGF1R;ROBO1;PDE4B;DIP2C;TLE4;NCOA2;CADPS2;BCAS3;DST;DAPK1;PDE4D;MAGI2;ARRDC3;DYRK1A;LMO7;MTSS1;RUNX2;KIAA1217;SETBP1;TBL1XR1;TBC1D5;KCNQ5;TNIK;MAML3;PLCB1;UTRN;SGK1;ATF3;VGLL4;CREB5;GAS7;CAMK2D;PFKFB3;DHX8;ITPR1;PPM1H;PIK3R1;CXXC5;NCALD;ABLIM1;PLXNA2;MEF2D;JAZF1;AUTS2;ZBTB16;ATP2B4;PDE4DIP;OXR1;PBX1;EXT1;SDK1;NFIA;FAT1;ADGRL3
SCAPER	62/299	0.00000140581072351939826277312725610890709049272118136286735534667968 75	PHF3;MAML2;ZMYND8;JMJD1C;ZBTB20;RSF1;PTPRK;EPRS;FNBP1L;PPP1R9A;SLC8A1;IGF1R;ROBO1;EEA1;SENP7;PCM1;REV3L;BBX;DIP2C;CADPS2;DST;PDE4D;ARRDC3;MTUS1;VPS13A;VPS13B;LMO7;MTSS1;KIAA1217;FCHSD2;ZEB1;NAV3;SETBP1;WDFY3;TNIK;PLCB1;UTRN;KMT2E;COLEC11;CAMK2D;ITPR1;BAZ2B;PIK3R1;MLLT3;ADD3;ABCA1;SPAG9;AUTS2;KIDINS220;MGA;NBEAL1;PDE4DIP;SORBS2;HELZ;SORBS1;OXR1;PBX1;KLF7;NFIA;SESTD1;WNK1;SSBP2
RFX3	63/299	0.00000129866728052223072154827340773941557472426211461424827575683593 75	ROBO2;THRB;MAML2;ZMYND8;JMJD1C;ZBTB20;PPP1R9A;SLC4A4;SLC8A1;IGF1R;ROBO1;ENC1;REV3L;BBX;TLE4;NCOA2;CADPS2;ST6GAL1;DST;ITGA4;TSC22D1;PDE4D;ARRDC3;VPS13A;LMO7;PROX1;MTSS1;PLCB4;ZEB1;SETBP1;TBL1XR1;RAPGEF2;PDE5A;TNIK;MAML3;PLCB1;UTRN;ATF3;COLEC11;ITPR1;BAZ2B;PIK3R1;MLLT3;ADD3;NCALD;ABLIM1;UBN2;PLXNA2;ABCA1;NTRK2;POU2F1;NEGR1;AUTS2;ATP2B4;ESRRG;SORBS2;LNPEP;PBX1;KLF7;NFIA;ETNK1;SSBP2;ADGRL

			3
SOX5	62/299	0.000014058107235193982627731272561089070904927211813628673553466796875	ROBO2;CNTNAP2;THRB;BNC2;ZBTB20;PTPRK;PRDM1;AFF3;SLC4A4;SLC8A1;IGF1R;ROBO1;CCND2;ENC1;PDE4B;PHACTR1;EDIL3;TLE4;CADPS2;DST;PDE4D;MAGI2;MITF;PROX1;FOXP2;KIAA1217;PLCB4;ZEB1;NAV3;SETBP1;RARB;TNIK;PLCB1;UTRN;SGK1;CREB5;GAS7;HLF;EPAS1;ITPR1;PIK3R1;NCALD;ABLIM1;ERBB4;PLXNA2;GPC6;ABCA1;NTRK2;NEGR1;AUTS2;ANGPT1;ZBTB16;FN1;ESRRG;SORBS2;SORBS1;SULF1;PBX1;DCLK1;SDK1;NFIA;ADGRL3
PLXNA4	62/299	0.000014058107235193982627731272561089070904927211813628673553466796875	ROBO2;CNTNAP2;COL18A1;RYR2;CLSTN2;COL12A1;ZBTB20;AFF3;SLC4A4;SLC8A1;IGF1R;ROBO1;CCND2;ENC1;PDE4B;PHACTR1;HMCN1;RNF150;EDIL3;CADPS2;DST;PRKCB;PDE4D;CACNA2D3;LMO7;PROX1;KIAA1217;COL4A2;NAV3;SETBP1;TNIK;PLCB1;CREB5;ITGA9;GAS7;FBN2;GRAMD1B;EPAS1;PRICKLE2;ITPR1;NCALD;ABLIM1;ERBB4;PLXNA2;SPOCK1;GPC6;ABCA1;NTRK2;NEGR1;AUTS2;ZBTB16;CADPS;FN1;ATP2B4;SORBS2;BTBD11;SORBS1;SULF1;DCLK1;SDK1;NFIA;ADGRL3

Table S12. Genes that retained their contacts with rDNA after heat shock control the neuronal cell development. Related to Venn Diagram in Figure 4D.

GO.ID	Description	# of genes	padj	Genes
GO:0045202	synapse	45	3.7806456304 88192e-8	*SYN3,PRKCB,SLC8A1,OPHN1,APBA2,IL1RAPL1,GRID1,EXOC4,NTRK2,RGS12,DLG5,SH3GL2,RAP1A,SLC1A2,GRIN2B,KCNK1,FCHSD2,IL1RAPL2,SDK1,GRM5,CBLN4,GPC6,SPOCK1,UTRN,DNAJC5,CLSTN2,PLD1,PHACTR1,VTI1A,RPH3A,GPC4,PLCB4,GRID2,PDE4B,MCTP1,GRM8,CADM2,DCLK1,CADPS,LAMP5,KCNK2,DNM3,CDH9,MAGI2,CASK*
GO:0097458	neuron part	56	0.00000252	*SYN3,PRKCB,SLC8A1,OPHN1,APBA2,ASTN2,IL1RAPL1,EXOC4,NTRK2,WDFY3,MTPN,SYBU,RGS12,DLG5,SH3GL2,CRB1,RAP1A,CNTN4,EPHA5,MAPK9,PDE9A,SLC1A2,GRIN2B,ROBO1,KCNK1,GRM5,IGF1R,CNTNAP2,KIF13B,SPOCK1,PAK1,UTRN,CDC14A,DNAJC5,CLSTN2,VTI1A,RPH3A,GPC4,KLHL1,PLCB4,GRID2,PDE4B,AUTS2,MCTP1,GRM8,CADM2,DCLK1,CADPS,LAMP5,KCNK2,DNM3,CDH9,MAGI2,ROBO2,DYRK1A,CASK*
GO:0044456	synapse part	33	0.0002522	*SYN3,PRKCB,SLC8A1,OPHN1,APBA2,IL1RAPL1,GRID1,NTRK2,DLG5,SH3GL2,SLC1A2,GRIN2B,GRM5,SPOCK1,UTRN,DNAJC5,CLSTN2,VTI1A,RPH3A,GPC4,PLCB4,GRID2,PDE4B,MCTP1,GRM8,DCLK1,CADPS,LAMP5,KCNK2,DNM3,CDH9,MAGI2,CASK*
GO:0044459	plasma membrane part	72	0.00101	*SLC8A1,KIAA1324,KCNQ5,SORBS2,IL1RAPL1,WDPCP,GRID1,LMO7,EXOC4,BBS9,NTRK2,ZNRF3,PHEX,DLG5,SLC4A4,CRB1,SLC16A7,TBC1D5,HMCN1,EPHA5,SGIP1,TRAT1,PDE9A,VANGL2,SLC1A2,GRIN2B,ROBO1,KCNK1,ADCY8,CACNA2D3,CACNA1D,GRM5,DIAPH1,IGF1R,JAM2,CNTNAP2,KCNIP4,CD96,ITGA9,KCNMB2,GPC6,ITPK1,PAK1,UTRN,GPC5,ENOX2,CLSTN2,PLD1,TRABD2B,PDE4D,PCDH11X,GPC4,GRID2,PDE4B,SGCD,SHROOM4,IGF2R,LGR6,GRM8,DCLK1,ANO1,GNG4,COL25A1,LAMP5,KCNK2,DNM3,PLXNA2,CDH9,KCNH5,ROBO2,SLC2A9,CASK*
REAC:R-HSA-112316	Neuronal System	21	0.0000090	*SYN3,PRKCB,KCNQ5,APBA2,IL1RAPL1,SLC1A2,GRIN2B,KCNK1,ADCY8,CACNA2D3,IL1RAPL2,GRM5,KCNMB2,DNAJC5,PANX1,GNG4,KCNK2,PLCB1,KCNH5,NCALD,CASK*

Table S13. The results of RNA-Seq before (hs-) and after (hs+) heat shock treatment of HEK293T cells (recovery time: 2.5 h). baseMean – mean of the counts divided by the size factors for the counts for both conditions. log2FoldChange – the log2 of the fold change. lfcSE gives the standard error of the log2FoldChange. Stat is the Wald statistic: the log2 Fold Change divided by lfcSE, which is compared to a standard Normal distribution to generate a two-tailed p-value. padj - the adjusted p-values. Excel file attached separately.

Table S14. The results of RNA-Seq before (hs-) and after (hs+) heat shock treatment of HEK293T cells (recovery time: 6 h) for the selected 4920 rDNA-contacting genes. Excel file attached separately).

Table S15. Overlapping of rDNA-contacting genes that decrease or increase the number of their contacts with rDNA clusters and change RNA expression. Related to Venn Diagram in Figure 5B – downregulated rDNA-contacting genes.

Names	total	elements
4C increased RNA-Seq downregulated	3	*C1orf112 HEATR5A SHROOM4*
4C decreased RNA-Seq downregulated	13	*POLR3K EPHA7 MIR17HG C5orf51 ZNF91 ZSCAN5A CRLS1 HIRA PSMC6 REV1 SNHG17 SRD5A3-AS1 ZNF569*
4C increased	2092	*AL590652.1 ELMO2 HPSE2 FARP1 LRRC37A5P TNIP3 PIGX AC084116.3 C10orf90 LINC01201 BBOX1 ZEB2P1 KLHL13 XDH TACC2 AEN CMTM8 PTPRR ACOT12 SLC10A7 PXYLP1 ITPK1 PAK1 AC021086.1 ANGPTL5 OR2B6 CNGB1 DAZAP1 MITF RTN1 RNVU1-14 STK32C FNBP1L COL4A5 AC010280.2 ZEB1 ADCK1 TRIM33 AC005062.1 NDUFAF4 MSH3 SLC16A14 MMS22L AP003469.4 BANK1 WSCD1 UNC5A PIKFYVE OOSP4B TMEM132E FMO10P NFIA NAT10 AC007389.1 RN7SL35P TMEM184A SYN3 TSC22D1 LHFPL2 AL133445.1 NMNAT1 LINC01655 C9orf43 ELOVL5 CYP2F1 PRKCB AC011498.1 CNBD2 SSX6P ACSL3 LINC01515 CDC14A UTRN AL391358.1 GRPEL1 USP6NL SDAD1 GPC5 ENOX2 LRGUK TERF2IP CD160 PDCD5 SLC8A1 LINC02487 KPNA3 ANLN CASC16 CEP350 PRR16 ZNF32-AS3 CIDECP IGKV2D-38 ARHGEF9 AC106806.2 BMT2 DYSF OR1D4 MTHFD2P1 AL360175.1 EXOC3 PPIL4 LINC01269 AC025741.1 EEF1DP6 RN7SL571P AP000253.1 DNAJC5 LINC02438 IP6K1 MIR513A2 DKFZp779M0652 AC110079.1 PRY AC092944.1 ULK4 OPHN1 DLX6-AS1 AC091173.1 SNX18P14 AC007834.1 PPIAP33 GCC2 AC087283.1 RARRES3 SORT1 TMEM255A PREP USP7 HS2ST1 CD82 MAP3K13 AC022201.1 GCLM SLC35D2 KIAA1324 SNX19 C12orf75 CHFR COL18A1 PIK3CB RNU6ATAC4P NRIR PPP1R9A SAA3P CCDC185 LINC02068 LINC02015 AC006238.1 C14orf132 RSRC1 GCK P2RY10BP PRPS2 THRB CELF1 ASS1 SLC9A9 AP003306.1 LRRC52-AS1 AC092957.1 SERF1A AC112487.1 SRP72 ZZEF1 PTPN5 RN7SL52P FOXB1 ARSH PRR29-AS1 DDHD2 LINC01270 IGSF11 CDK17 DPYD SENP7 AC008448.1 PPBP BACE2 AL359643.1 AF279873.3 AL139158.2 LINC01760 ITPRID1 USP32 QKI KDM6A AP001528.1 MED27 WNT3 LIN52 SLC25A21 PDP1 CLSTN2 AHCYL2 AL137027.1 TPT1P14 AC011487.1 POU6F1 Z68871.1 GDAP1L1 PLD1 CHRNB2 UBN2 GLG1 NDUFC1 AC034199.1 KIF2A AC090809.1 ZNF79 ARL6IP6 L2HGDH UBXN7 RARB AC006148.1 KCNQ5 AGPS PLCXD2 ZBTB20 TARID DRAM2 AC098487.1 RF00019 HLF RNU7-182P AC004830.1 TMED9 HEPHL1 COLEC11 APOBEC3B OR1D3P NBPF3 CEP85 NOP58 AF064858.2 C19orf67 AC073370.1 COP1 NADSYN1 LINC01794 PBX1 FN1 FAM126B AC008869.1 AL031733.1 AC005261.1 DLEC1 LINC01243 CAMSAP1 PHACTR1 RBM6 SORBS2 WAPL NFE2L2 TTC41P AC025263.1 ZNF275 SPINK5 KRCC1 RPS6KA6 LINC01445 CDHR1 AC112176.1 TFEC PROX1 FAM71F1 HPF1 PPM1H DCX GPBP1L1 RIC3 AC023245.1 ETFA BAALC-AS1 OTOA FSTL5 AHRR AL022324.1 DEF8 EIF4E2 KLHL6 MALINC1 GUSBP1 TTC13 CRABP1 AC009005.1 LINC02107 RNU6-477P CUL4B KCTD10 NEPNP SYTL4 TRBV5-6 ATP6AP1L ADGRG4 DCTN1 AL137145.2 USP17L24 APBA2 DDX39A DIXDC1 VTI1A CADPS2 LINC02077 FAM114A2 LRRC2 FTH1P8 HAGH TRIM45 SLC2A10 PTCRA ARHGEF26 AC008080.1 CRAT37 ASTN2 RBM26 BRINP3 SGK1 AC134698.4 DDI2 LEF1-AS1 LINC01237 RF00393 AL034397.1 CSMD3 CHST10 MIR4495 PECR FAM120A TRAPPC2 DISC1FP1 FHIT AC104695.2 NFKBIZ SPAG9 GPR135 SLC39A9 LINC00348 LINC01861 NKIRAS1 RPL7AP57 TCEANC AC156455.1 AC100781.1 BARD1 USP17L18 ARHGAP25 FAM19A1 AF038458.2 TRABD2B AC009262.1 SVIL-AS1 HNRNPA1P34 AC092646.2 USH2A C1QTNF7 LRP5 LINC01378 AL772202.1 NEGR1 GALNT2 CTNNB1 ELK1P1 AC092078.2 KIAA0753 ANKRD17 USP17L19 B3GNT2P1 UBE2J1 FAM157A AC083949.1 AC134915.1 CLIP2 PRDM6 RNF150 VEZT HNRNPA1P51 SLC35B1 GK5 SIGLEC1 RN7SKP49 SOCS5P2 AC078980.1 ADD1 ZNF136 ZSCAN12 AC096773.1 LAMC3 ANP32A UBE2Q2 DNAJC1 TAC1 PGGT1B AC062028.1 ASZ1 KCNN3 DPY19L3 KIAA0232 WIF1 FBXO3 AC093730.1 LINC01339 AC104170.1 LINC02117 LINC02031 AC079779.2 AP006296.1 HERC3 SH2D3C ZBTB47 IBA57 PDE4D LINC02147 AL163642.1 GAP43 POFUT2 LINC01799 PCDH11X EDNRB-AS1 OR1L3 NTPCR C10orf143 HIPK3 PCNPP2 AL133523.1 LINC01251 HNRNPCL1 MORC1 PPP6R2 KIF9 SETBP1 MIR548A1 PITPNM2 ZBTB7C AL158825.1 ZNF780B ENOPH1 EFCAB12 RN7SL290P RUFY1 PGLYRP4 RAB9AP5 ADRA1D RNU6-144P LINC02463 ORC5 ARSA PIH1D3 BIRC2 GAS1RR ZC3H12C NLGN3 AC022387.1 PLS3 EGFEM1P AC093459.1 AC090001.1 AC005244.2 PIANP AL390729.1 CKS1BP6 NLRP9 IL1RAPL1 WDPCP AC007923.4 NUF2 GAL RCC1 PRNT OR52B3P ASAH2B GNGT1 MBD2 AMOTL2 PLA2G10 LINC01111 ZFPM2-AS1 PDK4 BLK LINC02220 FARS2 HRASLS SAMD5 AC074254.1 LINC02180 SSBP2 SLC44A3-AS1 RERGL RPP30 GTPBP2 RPH3A GOLGA2P7 CD109 CEACAMP11 GRID1 GREB1L PRDM1 SLCO6A1 SMAP1 EFR3B BBX AVL9 AC142381.4 ARMC2 AC021074.3 ZFYVE9 FER1L6 LINC01790 SMPD4P1 TBC1D16 AC122138.1 AC006272.2 CACNA2D2 SYN2 SEPT7 KIAA1109 PTPRC LINC02299 BEST3 LINC01681 LINC01365 URB2 AL031687.1 AC005798.1 CCND3P2 AC136489.1 CNBD1 SYNPO2L CFAP70 AC007100.1 HDDC2 Z93242.2 EMCN OR4C1P CCDC162P IGHMBP2 MIR4453HG TRAV19 ZNF767P CCSER2 AL139806.1 GPC4 LMO7 AL353651.1 COL4A2 MSC-AS1 C1S CCT4P1 ZNF84 AC132938.1 BRD7P5 ME2 LINC01554 AC104009.1 STARD8 GRAMD1B FOXP2 NKAIN1 AC093716.1 SIAH2 POLR3A ADGB EXT1 CLEC4D EXOC4 VSTM2B ADGRE5 TCHP IMPG2 ZNF385C AC073316.1 MMP3 FOXJ3 SLC35F5 OR1D5 HIGD1C BCAR1P2 AC106772.1 LINC02432 RSPH14 PARP12 SYNE3 XPO6 C6orf89 AC002091.1 ANKMY1 BBS9 C9orf72 SSBP3 CYP4F22 MAP3K21 FAHD2B HACL1 LINC01182 FAM86C2P CDC27P2 AC099520.1 KLHL1 AC004540.1 NTRK2 CCDC3 MFGE8 AC092106.1 KIDINS220 SH3BP2 PLCB4 HSD17B3 TLR10 WDFY3 MSH4 NARF AC239859.1 WDR92 AC026765.2 LINC00476 SAMHD1 RPS4XP3 UGGT2 DHX8 TRIM4 SH3YL1 CT55 FMO3 LINC00630 TDRD3 OVCH1-AS1 KLHL15 ICMT AC010931.3 ZSCAN5B PIP4K2A FARP2 LINC01995 MAPKAPK3 PACS1 AC010320.2 AL355075.4 INTS6-AS1 UBAC2 LINC02313 SLC25A17 TTC39A RORA-AS1 UGT2B25P AL512361.1 AL139383.1 AC124657.1 CRYZL2P-SEC16B LINC01795 LINC02283 LINC00273 BAZ2B TRHR EML6 CPSF6 CENPW LINC00992 ZNRF3 AP005433.1 AL121949.2 PRSS55 LINC00858 MINPP1 EYA3 XIST PPIAP85 OR7E111P MGAT4C SH3D21 IPCEF1 CYP2AB1P AC097486.1 D2HGDH C1RL-AS1 ARFIP1 LINC00877 KRTAP13-5P HSPA12B MPP6 TBL1XR1 DBNDD1 HELB SZT2 MUC17 MGST2 CTPS1 TMEM126A AL731661.1 PDE5A NCKAP1L PPIAP5 ZNF541 RBBP8 OR5G5P TFP1 NRGN MED13 SCLT1 SMIM31 TTI1 NSUN6 AP005436.2 MOCOS UBE2H TMPRSS7 AC099398.1 CALCR AC037450.1 BTBD11 MBTD1 AC025283.2 GRID2 ZNF423 USP33 UNC13A RPS8P4 CCT3 AC105362.1 PSMG3-AS1 RNU6-1113P GUSB COPS3 DSTNP5 EHMT1 TTC29 RAX GCN1 WDR27 MED21 ZNF415 ZDHHC13 PLPPR1 SPTBN2 TOMM70 KLHL31 ATP2B4 KANSL3 HHLA3 AL807761.4 FGF5 C4orf51 OR11Q1P AC079380.1 STPG2 REPS1 CCDC85C FBXW7 PDHX*

		OR2B7P SUCLG2-AS1 AC090791.1 TBC1D32 MTPN GSTA6P C1orf105 GOLGA6L4 HEATR6 RUNX2 PDE4B KRT18P49 ATP5F1C MAPRE2 FGF10 POLR2A KRT7 KIF13A CROCCP2 MBNL3 KRT18P59 LINC01331 DLGAP5 AC048387.1 PHEX PHF8 WAC-AS1 DPEP1 SYBU SGCD TMEM87A PDE4DIP RSPH10B L3HYPDH ZNF204P RASGRP4 RNU6-374P SCN7A CD209 TIPRL AC009107.1 PLG TYR TP53TG1 IL36RN TMEM64 LINC01115 MED12L ZDHHC21 LEKR1 VPS54 LINC02580 STS AC082650.1 TM9SF3 TMEM123 LINC01476 SLC9C2 RBM41 CLSPN RGS12 RAPGEF2 NAV3 PLEKHD1 CDH7 PRRG3 PRIMPOL CHIC1 SULF1 PRDM5 PUDPP1 BUD13 UBE2E2 KIF3A SUCO AC009387.1 C13orf42 IMMP2L IL32 FTCDNL1 EPHA5-AS1 PUDP SH2D4B TRIML2 KDM1B AC010148.1 AC026336.1 FAM168A RASA1 RN7SL468P QRFPR NONO EEF1GP2 FAT1 KLHL42 MBP CPS1 ALG5 LINC01798 NCOA2 TANGO6 SLC35F2 AC024940.1 DCAKD STAM2 PIK3R1 LINC02270 HELZ TRAK1 USP12 HMGN1P11 C17orf112 ADAT1 CENPI EIF4H AC118942.1 ANKRD33B KCNQ3 AC016205.1 ABCB11 KATNAL2 AC010493.1 BOC XRCC5 CFDP1 DLG5 PLA2G16 RAB1A RPL21P39 AC099673.1 NFXL1 AC090825.1 C2orf69P3 USO1 PROX2 RUFY3 GPR63 ZHX1 NFKB1 MANBA TMEM135 LINC02522 CLCA4-AS1 RBMXP4 KDM2B MARCO NSUN7 BRD7P1 RNF8 IGF2R MIR4500HG LINC02177 OR8B5P ST6GAL1 EIF3E IL9 ZMPSTE24 RAB28 DEDD2 UBE3A RNU6-320P CCDC144NL-AS1 NSFP1 TLE4 AC010327.4 SH3GL2 ST3GAL5 MRPL23 DHRS1 ORC4 AC135506.1 DEK FAM117B VPS25P1 AGBL2 AL022722.2 DCN AP001628.1 RSL24D1P9 PLPPR4 HTR2A GRK7 EPAS1 R3HDM1 NRBF2 RSF1 KRT12 MYLK3 MKRN1 PCNX3 POU2F1 AL133163.1 CLNS1AP1 LINC02444 GALNT1 PTCHD1 AC131011.1 ANKMY2 TMLHE STX6 AP003059.2 TPR BPI TTLL5 SPINK8 ZNF734P BNC2 MARCH11 KRBA1 AC107029.1 LINC00364 STAB2 CCT7P2 RTN3P1 AUTS2 FBXL4 BCAR1P1 AC106900.2 EIPR1 PTDSS2 NFU1 UGT8 ALG1 RNF123 SFXN5 AL035413.1 CCDC178 SESTD1 SLC4A4 LINC02384 ESR2 ACOT8 LINC01847 DNAI2 SMAP2 ZBTB46 MAP7D1 CCDC148 TWIST1 SELENOT ENPP7P6 JRKL MAPK6P1 EEA1 CRB1 BCAS3 AC010105.1 ADARB2 TTTY10 SLC16A1 AL078581.2 EHHADH SLC17A5 AC026826.2 SI AL449403.1 RNU6-535P KCNE4 RPSAP27 TEX15 BAAT SLC38A11 COX10-AS1 CH25H COL12A1 TMEM206 VGLL4 CHST3 AC114781.4 ABLIM1 THORLNC TFAP2E AP002414.5 C1orf43 COLQ RBMS1 FRMPD2B PPIP5K1 GOLPH3L SYAP1 GBX1 RF00096 DCAF17 LCEP1 PDE3B MCTP1 UCK2 LINC01639 CDIP1 NPIPB1P AFF3 TET1P1 DARS2 ERBB4 SLC30A9 DNASE1 OR2T33 RBMY2JP TNIK MEF2D AC093827.1 PANX1 MICU3 WWC2 GSTA1 RNF25 AC092364.1 AC004987.3 AC092447.7 MPPED2 JPT1 CCDC59 LINC01257 KMT2E CHRDL1 MEF2C-AS1 ADD3 PTEN ARSF TAF4B FRMD3 AFF1 SUSD5 RAP1A LINC02226 ATP1A3 ERI1 XRCC6P5 TUBGCP3 UGGT1 NBEAL1 LGR6 TEX36 RAC1P8 CLGN ENPP4 SMARCC1 CLHC1 FHL3 SLC37A3 NOSIP SYNPO2 NUP210L LINC00355 LINC02465 USP20 LMNTD1 L3MBTL2 TBC1D31 LYRM2 ST6GALNAC5 CHSY1 FBXO8 XAGE5 AL513329.1 SLC25A26 DIAPH2 CRY2 PNPLA4 DIP2C TCAF1 GRM8 RNF141 LIN54 AC004869.1 AL078590.3 MAML2 TCP10 NFATC1 BIN3 CD55 LYPLA1 SSX2 LNPEP AC023796.1 ATF3 DGKH AC005840.2 RPL22P22 LINC01934 CCDC39 LINC02532 NPHP1 FBN2 SLC16A7 AC234771.2 RTN4R PHF3 NEDD4 ZNF43 OR5AU1 TPM3P4 AC111152.2 ZRANB3 RBBP9 RBM33 KLHL23 SPATA17 FMO4 AC010198.1 CNTN4 TBC1D5 GPR173 GLRX2 CACHD1 PCMT1 LINC01556 AC017037.1 LINC02534 TMEM222 NUP62CL ARNT HSPBAP1 AC114550.1 MARK1 PRKCI PRTFDC1 CABP5 ABCA1 CCDC26 AL022310.1 GSTA9P SLC8A3 AHCYP3 MBOAT1 ALKAL1 PRY2 ARFGEF3 ULK1 ZNF175 MAP3K14 PHC3 PPP1R8 TNFRSF11B GOLPH3 C12orf50 CADM2 MALRD1 RPL9P15 USP24 C6orf203 PRKG2 AC090679.1 LINC02477 FANCL AC122683.1 AC017002.3 URI1 SOS2 RPS24P8 DCLK1 AC122685.1 KCNH2 CDK2AP2 LINC02320 CAMK2D USP10 IFNG-AS1 SDHAF3 MROH8 HMCN1 AC096554.1 ZMYND8 CCDC186 CDC27P1 C9orf163 WASH8P OR52I1 DHX30 LINC01695 LINC00649 FAM135A PTPRB STRIP2 AL390760.1 RN7SKP206 LINC01412 CASC3 EPHA5 LINC01525 FAM156B AL929601.2 AP003715.1 AC012468.1 CATSPERG ITSN2 SGIP1 NECAP2 PPARG AL353678.1 AL357315.2 MMAB OSER1-DT Z93930.2 SERPINB7 EVI5 AC073264.1 CICP22 SYCP1 ISX-AS1 ATP13A4 NPSR1-AS1 CACNG4 RNU5B-2P TENM3-AS1 RASGEF1C AC055758.2 RCC2 AC090115.1 ALDH1A2 LINC00535 SHCBP1L MTTP MAPK9 THSD1 ESRRG OTX1 NYX WIPF3 PARP14 DYM TMEM165 MAGEA9 TEKT3 AL663109.1 STOX2 DNAH1 AGAP9 ABCC4 VGLL1 RNF219-AS1 TRAT1 AP000282.1 TAMM41 LLGL1 AL357060.1 LINC01942 IP6K3 FAM169A DYTN HSFY4P MAP2K6 HUWE1 POLA1 ZC3HAV1 RRAS2 FAM149A PDE9A TENM1 NBPF20 SEC14L3 DDX42 HTR3B TXNRD2 AL135938.1 MYPN KIAA1217 LINC01337 AC003958.2 ODAM AL512303.1 RP1 VANGL2 TBC1D2B KRTAP9-6 AC105415.1 SUB1 ANO1 DEFB103A WWC1 SMIM12 AP000442.2 SNX4 MYO1H SELENOF AC020718.1 SENP6 GAPDHP32 VEGFC SLC1A2 XRCC6P1 ZBTB16 AC105430.1 CADPS GOLGA8A STXBP5-AS1 LNP1 AC019186.1 CCDC158 AC012213.1 LINC01228 AL590867.1 LINC01477 NGB REV3L GAPDHP64 MAML3 RSPO4 RGS7 FAM167A NYNRIN EPRS LINC01470 PAXBP1P1 GRIN2B FMO8P NFYAP1 ZNF518A LINC01892 CCDC134 C6orf99 S1PR4 PHC2 LINC01142 CA5BP1 ROBO1 ST3GAL6 TNMD ARSB MYLK4 SPATA22 AL133371.1 AMPD1 AL731556.1 CWC27 MTCYBP14 RN7SL865P RBMY2NP ANKRD11 PRKX ITPR1 AC018521.1 AL137224.1 KIF5A EIF5A2 EMX2OS SULT1C2P1 LINC00278 SERF1B CNNM4 AC010754.1 ARMCX5-GPRASP2 AC018450.1 GNG4 RABGAP1 LINC01924 AC107057.1 OXR1 CAMLG AC010132.1 LHX8 AL355615.1 CIDEC AC140658.3 AC104457.1 CD300LG ENPP7P8 ACO1 WDFY4 LINC02455 CTH GRHPR SEMA7A IL15 MEI4 PRRC2C PAPPA2 STK33 ANKRD18DP KCNK1 AC008565.1 DAPK2 PFKFB3 SIN3A OSTM1 DDX18P3 PHBP21 AC005562.1 MINDY3 CRYBG3 GALNT7 AC107220.1 AC010203.1 HHIPL2 COQ3 ATP5MC2 UBXN2B ALDH7A1 CSNK1D AC008591.1 EDEM2 AP005357.1 OR7E162P RCAN3 SLC26A11 ATG13 AL157400.3 GPR149 FCHSD2 STAT6 WDR64 ZNRF2 NEXN-AS1 THOP1 ADCY8 CXXC5 AC073587.1 TP53BP1 COL25A1 OCIAD1 ELP3 AK2 ZNF787 AC099518.1 VPS13A AC009961.1 UTP23 RAB9AP2 TES AGXT2 SH3TC1 MIGA1 C8orf34 LINC02005 AC114316.2 MLLT3 ABLIM2 JAZF1 Z83844.1 BTG4 CCDC30 ELMOD2 SHC3 CCDC130 AC020704.1 RSPH10B2 CUX2 MTUS1 AFG1L HYDIN C1orf140 AC005229.1 AC018866.1 CCDC151 AC092894.1 MGMT MCEE LINC02583 LINC02513 DDX4 TXNDC16 LINC01249 CTNNA3 SPATS2 KPNA5 AKAP4 VPS35L ZNF184 AP001836.1 RAPGEF4 HEG1 AL357055.1 PPP1R3A FBXL19 SKA1 OR5F1 VDAC1P9 LAMC1 CLTC AL441964.1 AC006296.2 PBX3 TLR3 ZNF761 MTCYBP4 PDXDC2P-NPIPB14P AC090888.3 PRICKLE2 DSG1-AS1 CSRNP3 CDC7 TRAV8-4 ANGPT1 MCCC2 HEATR1 PSMD14 AC107021.2 TMEM154 AC006372.2 MRGPRX1 AL133372.2 LINC01048 NUP210 CREB5 LRRC47 AC004522.2 AC093106.2 B3GAT2 CACNA2D3 LAMP5 SLC4A10 ADGRG6 ACSS2 BAG6 AC012574.1 AC144568.2 AL355674.1 PKN2 XPR1 ITGA4 SEC23A FERMT2 TSSC2 PHLDB1 AL139231.1 TPD52 SPESP1 DACH2 AL390860.1 RGS2 PTPRK STK11IP ZC3H13 ZNF45 GFRA3 GAPVD1 TBC1D3P4 GOLGA8M LINC01060 PSG9 CACNA1D AL390718.1 PRKAA2 SGPP2 KCNK2 SORCS1 ARMC3 KIAA2012 DNM3 AC092818.1 IL1RAPL2 LINC01555 TBL1X SPAG17 AC005614.2 CHCHD3 EDIL3 CNNM1 IGKV2-38 SS18 AC138035.1 SCYL2 ITGB1BP1 LINC02531 ZC3H7B PPP1R12B VPS37C AL592291.1 SAP130 STARD6 LINC01120 PCDH1 PLXNA2 ZDHHC15 MRPL32 TRIM67 AC087672.2 OCIAD2 TRBV7-6 AL353072.1 CLCA4 MAOB AC064862.6 LINC02430 SMURF1 DEFA7P AC111152.1 AC113386.1 BTNL8 OR2A1-AS1 BCL2L11 FOXL2NB MIR4445 PCDH11Y KLF7 AHSA1 LINC00358 GDAP2 TRBV4-1 TRAV26-1 METAP1D AC092673.1 PLCB1 AL445647.1 LINC00899 BEND2 SNPH AC084064.1 UBE2Q2P8 TAF13 BRIP1 GRID2IP CDH9 SLC7A5 CYP20A1 RPS3AP25 SORBS1 LPA NDUFAF2 AC113355.1 OR3A3 PACSIN1 LRCH2 AC113398.1 AC097510.1 RNU4-73P MND1 LINC00383 ZC3H12D MGA AC092436.2 ZNF876P SRSF10 MAGI2 PPME1 NLRP2 TNPO3 MAP3K2 STRA8 AL133346.1 AC010476.1 AC008060.3 CYP4F62P RF00411 AC004943.2 GRIK5 AL392086.1 ARRDC3 TBPL1 ERBB3 SDK1 SLC16A13 ZNF844 NIN PLCL1 RN7SL605P SLC1A4 FREM3 GALNTL6 PSAT1 GRIN1 CALU OR1A1 LINC02488 CCDC144CP RPS3AP35 TNFSF13B DENND3 PUS7 AC007861.1 AC004840.2 XPA AC073172.2 MMAA INTS9 AC123905.1 GRM5 AC099508.2 PAMR1 IRF5

		HNRNPM ARL14EP RPL31P40 DIAPH1 OPRD1 AC040963.1 ELOVL6 AL359837.1 COMMD10 AC016708.1 SACM1L MBD3L1 CX3CL1 ADGRL3 DLX3 UBE2E1 SLC30A10 NECAB1 LINC02149 IGF1R COL14A1 LRRC4B EFEMP2 PPDPFL RPL21P12 PCM1 TSG101 LINC00942 SLC20A2 AC007879.1 PAQR5 AC019197.1 JAM2 NPIPB10P ITGA9-AS1 SMIM36 AC025947.1 NCAPD2 OTUD6A PPM1L SMOC1 ABCB4 SLC25A33 FAM185A CNTNAP2 FRK AC117440.1 PARL TOGARAM1 AC018742.1 RYR2 RNVU1-15 AL049697.1 RNU6-1047P SLFN13 LINC02615 CBLN4 ZFYVE26 KCNIP4 RTP4 TBX20 AC011287.1 DST DZIP3 AC131211.1 CD48 RNU6-1032P RBMXP2 LNX2 ERP44 LINC02516 FGD5 AFMID AC022166.1 AC093599.1 JMJD1C EIF4E3 KCNH5 MAGEA1 NOS1AP RUSC2 AC003035.1 BAZ1A RBMY2KP TMC3 ZCCHC8 INPP5D KLHL29 OTOGL CFAP161 ZFAND3 AC118757.1 TCERG1L OPRM1 ACER2 LINC02475 ZFP64 IFNA4 CALML3-AS1 KIF13B CNKSR1 MPDZ SDHC EYS IL16 LINC02235 LINC01505 UBTFL10 ZNF517 HMGCLL1 XKR6 LINC02382 DOCK11P1 ACVR1B CD96 AC011890.1 CCDC15 AC087241.3 OR6C66P ROBO2 FAM83D OXNAD1 AC090833.1 FAM122C TMOD1 PDCD11 COQ8B NHSL1 RBMX MPP3 LINC00430 DTHD1 AC093802.1 FLT1P1 NT5C2 AC110995.1 NCALD OAF GAS7 AL122019.1 SLITRK1 AC092809.4 CLEC16A MTSS1 DYRK1A PPFIA1 RAB11A PROX1-AS1 HBS1L PPP2R2B DENND4C JAK1 AC078785.1 GPD2 CDK19 ITGA9 ZNF800 GALNT11 SCUBE2 ACSL6 RF00416 PITPNM3 EI24P2 SDR42E2 KCNB1 LYRM4 CCND2 CUL4A AC137579.1 LINC02099 HSP90AB3P PCP4 PTPN3 AC025260.1 FAM217B AC093426.1 RP1L1 GPRIN3 GNPAT AC093801.1 MYPOP RAP2C-AS1 PCSK5 LINC02386 CTPS2 TJP2 PGBD2 AC113208.2 AC003958.1 AL357079.1 GUSBP2 PAX3 TXNRD3 LINC01510 AL391361.2 AL356218.1 MYL6P3 SSX2IP LINC01748 PLB1 TTC17 TAPT1 ENC1 KYAT3 KCNMB2 POMT2 HPS5 AC092978.1 ST13 PTP4A1 MARS TOPAZ1 CFHR3 BMPR1B GTF3C6 VOPP1 RPL12P41 AL139344.1 OR7E109P AC245291.1 PSMA8 NLGN4Y DEFB103B AC073333.1 AL359317.1 MIOS AC096644.2 TDO2 AC096669.1 NAA25 LINC01234 CLUAP1 RNF19A RNU6-65P AC104071.1 AL591074.1 AC242426.1 SPATC1L AC010884.1 GMPS LINC00886 GPT2 AC034231.1 RABL2A NPS RN7SL761P GTF3C1 RNU6-364P DOCK3 CES4A USP17L17 AC142381.3 NDUFA10 PDSS2 SEPHS2 CCDC92 ETNK1 PRAME TEPP LARS2 U91319.1 B3GALNT2 CLNS1A NUP43 GPC6 CYP4F30P BCAT1 KBTBD12 GGTA1P WNK1 SLC2A9 ARL17A ADGRV1 SPRYD3 SCN2B ZNF398 MAMDC2 PDIA4 CAPN7 ZSCAN22 AC024909.1 AC073311.1 LINC00845 LINC02379 AC079950.1 CYP4F11 ZKSCAN7-AS1 KDM3A NOTCH3 TULP3 APBB1IP AL390962.1 ADGRG7 TRIM15 SOCS5P1 AC009313.1 RNA5SP45 ANP32B TAOK1 UNC45A RNU6-111P RPL3P7 PPP1R26-AS1 CASK NUDCD1 RASSF6 FLNA AL359853.2 TRBV5-1 AC119396.1 AC009093.2 AC138915.2 RNU6-641P SNAP91 DAPK1 MAP3K19 AC011474.1 RFTN2 AL161727.1 AF121898.1 AJ239322.1 LCE4A ALDH9A1 TTC1 SUSD1 AC092620.1 RNA5SP443 LRP11 PDLIM1P2 SIN3B OR51P1P MARCH1 EIF4A1P7 NPTXR FO393414.2 TP63 SEC22A GATA4 LMBR1 AC241644.2 ELMOD1 ZPBP SLC25A43 RETREG1 KIAA0825 SMAD5 GARS FAM49B DNAJC13 LINC00470 CNST LINC02208 IGLV3-4 TCP10L2 VPS13B FRMD4B PFDN2 AP001351.1 AC125603.2 CYP2R1 CRISP1 RF01210 RPS4XP18 RAB7A BIRC3 RNLS ZNHIT6 RNU6-830P SPOCK1 DNAJC10 RNGTT RNF169 LINC01478
4C decreased	931	AC092115.3 RNU7-66P CMTR2 AL031601.1 KRTAP4-12 AC137499.1 GLT1D1 AL589182.1 CYP39A1 LDB3 TMC4 EPB41L4B AP005901.1 CHCHD2P4 HS3ST3A1 KLHL14 ARL2BPP8 BTN2A1 LINC00520 SP140L G2E3 CCDC88B AC008667.3 AC004656.1 LINC01491 LRRC37A4P RNF185 MAGEB3 CXADR POTED LINC02125 CCDC34 KIAA1210 RF00426 CECR2 LINC01915 LINC01701 LINC01441 AC133065.1 AL356800.1 ZNF257 C10orf120 GPR78 AC138701.1 GTF2IP4 ANGPT4 VAT1L AL031674.1 LINC01410 AC011193.1 AP003108.2 ABCD1P3 CHST15 PKIA-AS1 LINC02499 AC010615.4 SERPING1 AC024475.4 AP000547.1 ASIP CDH10 KL LINC01789 SAMSN1 SPANXN4 OR7E25P AL049812.2 LINC02250 TF FGF7P2 OR10T1P AP001341.1 ZNF331 POTEG AL021937.3 OR10J6P AKR1B15 RF00421 MIR548XHG PWRN3 CDC14C COX10 CENPU LINC01502 TMEM11 KRTAP29-1 MYH13 GNB4 LINC02284 AP005212.1 MMP27 BDH2P1 ACTR5 AGGF1P3 HK3 LCA5L GANC ZNF37CP FAM225B ZNF583 LINC01058 AC084373.1 OR4C9P AL136968.2 HS1BP3 AF064860.2 TRAV8-7 LINC02309 AC044784.2 PLSCR4 STRIP1 MSNP1 AC091826.2 RNF182 AC012322.1 AL031289.1 GPX1P2 VRK2 AP006748.1 NDRG2 LINC00113 PHYKPL HPYR1 LINC01432 AL133173.1 LINC00960 ZNF962P ZNF384 AC026741.1 PSMC4 ZMYM5 AC089987.1 OR11L1 RCAN1 FAM193A LINC00408 OR4A9P EDDM3B SIRPG RAB5A AL355607.2 KANK3 AC006206.2 LINC01070 JRK AGGF1P2 SNX18P9 ZNF840P ANKRD20A5P AC037471.2 ARR3 AC016930.1 IL20RA LINC01194 GAS8 LCE2B LINC01907 AL162632.2 TRPM2 FRG1-DT LINC00507 S100B GABRG3 AL356095.1 AL137845.2 STRN3 CILP2 CMKLR1 LINC00575 TNFRSF19 NAP1L6 AC092423.1 AP000705.1 BMS1 MXRA5 AC124944.2 KRT74 SPINT4 AL121718.1 AL352984.1 RNA5SP519 AC016737.1 AL132708.1 ITGA2 RNU1-142P RPS3AP41 ZNF965P MBL1P AC068446.2 RNU4ATAC8P AP001605.1 IGHVIII-2-1 AC062021.1 NIPA2 OR4C14P AC119751.4 HUNK AP001574.1 LINC02311 GBP4 AC068643.1 MAPK1IP1L NEURL1-AS1 RPS23P5 ISCA1P3 REEP1 PQLC3 MKKS CHMP4C BCL2A1 AC140658.7 CR1 AC026434.2 AC009271.1 LINC02409 DTX4 AJAP1 USP40 SNX19P2 AC021439.1 IGHVIII-26-1 HFM1 AL121999.1 GSG1L STK24 TMEM231P1 NCOA5 RNA5SP478 AC099654.1 AC106871.1 AC005580.1 RN7SL568P SOGA1 NUTF2 PAGE1 TPT1P2 AC093074.1 AC092681.1 AC114501.2 EIF4E AF064858.1 AL669831.5 BPIFB1 STARP1 ZFAND1 LINC00320 AL109618.1 KLHDC7A TUNAR ERCC4 FAM83B OR4K15 NCOR1P1 NF1P4 ENTHD1 LINC02305 GADD45A AC018730.2 MOV10L1 RCHY1 AP000547.2 AL356276.2 CR383656.6 NF1P1 FAM201B NLK AC079062.1 NIPA2P2 IPPK AC063952.1 AP000534.2 MYEOV TMED8 OR5L2 BNIP3P6 PRIMA1 LINC02277 POLR2F KRTAP5-8 TMPRSS3 ATP1A4 AP003351.1 AC130464.1 RNF152 C1QTNF6 MEOX2 CEMIP DUX4L19 KCNG3 LINC00365 AC025884.1 OR4S2 LINC01495 AK6P2 AL139348.1 TMEM52B SH2D7 LINC01268 LINC01094 DUSP27 AC097493.3 OR8K4P NANOGNBP2 CYTIP AC099793.1 AC073325.1 AC020595.1 AL139393.1 AP000233.2 DPP9 AC008608.1 ABCD1P4 AC009567.1 GLDC CHEK2 SUZ12 LINC01684 AC118282.3 BLZF2P AF121897.1 ZNF729 SIGLEC30P TSPY5P AC005185.1 RAB22A AL158090.1 PJA1 DDX21 ANKRD20A9P ZNF688 PWP1 AL358292.1 ILDR1 GRXCR1 TTC22 STXBP4 OR5D15P AC008794.1 MED15 ZBED4 MT1HL1 INTS13 ZER1 LINC01648 OR4C2P OR52X1P AC091193.1 AC087883.1 AL049651.1 ISX RAD51AP1 FAM182A POTEM AC092966.1 LINC01687 LRRC1 CR383658.1 SEC14L6 CYB5R2 DET1 AL022332.1 ANKRD20A11P LINC01020 ACTBP8 AC007848.1 POTEB2 AC027612.4 IL4R SDR16C6P TM4SF1 ARHGAP27P1-BPTFP1-KPNA2P3 AL589743.2 AC060788.1 LINC02322 PNLIPRP3 ZMYM2 TAB3 AC069545.1 AC145543.1 RF00156 AC016044.1 ACTR3BP5 AC097532.1 AL365232.1 ERMN TRIM48 LCP1 AC013460.1 Z82249.1 RMRPP5 WBP11P1 ADCY2 KRT25 NOVA1 AC009169.1 MRPL58 GNG5P5 LINC01227 FRG1BP IATPR SH3BGR AC098826.1 SPRR2G TMEM211 AL121821.1 LINC02089 LINC02101 TRDV1 RPL21P41 ABCC13 AL663058.1 LINC02347 MARK2P9 ATP8B5P LINC02082 IGHV1OR15-9 PATE4 HACD4 PMP22 FAM111B AL137009.1 LINC01683 AC097532.2 PLEKHA5 AC023078.2 WRB CIB4 AP000470.1 LINC00317 POTEB CDC20B LINC00347 AL773545.1 AC068722.1 TFF1 SYNM AC025857.1 PLEKHA6 GLP2R MAP2K3 AL020994.2 IGKV1-5 AC126603.1 ANKRD34C AC245028.2 KMT2C MT2P1 DIO3OS IFNWP4 AC140847.2 LINC01644 OR6K4P C14orf177 AC005863.1 COMT AC018904.1 SNX19P1 IGHV3-76 AL353633.1 AC009081.1 CD1C AL355516.1 DUX4L16 RNU1-51P RF01182 AC106785.2 UGCG ORC3 AL035250.1 VWFP1 CHST8 SLC9B1P3 AL512310.1 RGMB LINC00839 AP000265.1 AL117190.1 HSPB8 LINC00884 AC090618.1 PXT1 RLBP1 KLRF2 LINC00308 CYCSP6 FP325330.1 AL049875.1 ZBTB34 ZNF826P KCNE1 ITLN1 TRMT61B AC015771.1 MAGEC3 FPR3 AC009652.1 TSPAN3 DSCR8 CYLC2 AL049775.2 TPP2 AC007333.1 RPL12P12 AC097501.2 BTNL9 LINC01920 URB1 AC108047.1 AC006273.1 BASP1 GRK6 BMPR1A RPL23AP12 ABCG1 SQSTM1 FAM207CP ABHD4 AL353148.1 AC068760.1 AL158042.1 DEFB116 CFAP46 OR7E104P KRT8P25 AL359081.1 LINC01262 LINC01203 SLC16A12 SOCS6 AKIRIN2 LINC00929 SOD3 CSF1 AC005476.2

		LINC01603 AL121852.1 GRK3 RNA5SP495 AC092175.1 ANKRD30BP1 MPPED1 FO393418.1 AC090136.2 AF241725.1 LINC01809 PWRN2 CYCSP51 GPATCH2L AC087341.1 PAXIP1-AS2 AC005307.1 ETS2 FCRL3 CECR7 CTBP2P4 OVGP1 ARID5B RPL26P28 AL157756.1 AC105148.1 AC016573.1 RF00012 AL772155.1 CR383656.10 TMPRSS6 SERPINA1 MYBL2 RBM19 ZNF527 FEM1AP1 AL110503.1 CDR2 LINC01229 AC119751.1 AC026495.1 ITGB7 D21S2088E ZSCAN10 BRF1 NLRP13 ACTR3BP6 MANSC4 AF212831.1 WT1 OR4C5 MAPK14 AC023824.4 DYNLL2 PRR14L KSR1 AC138123.1 CFL1P6 TUFT1 FBLN1 AC006548.2 MTFMT RAF1 AP001116.1 AC009139.2 AC142384.1 EDN3 ROCK1P1 GRPEL2P1 PRLH AP000235.1 CR786580.1 PSMB2 SNRPD1 CST9L LINC02200 LINC02485 AP000542.1 AC132825.3 LINC01938 CYCSP17 RPL12P27 DMBT1P1 DIO2-AS1 HBD KBTBD11 GALC AL607077.1 AC245748.2 PWRN1 WDR4 AL137792.1 LINC01706 RPS4XP22 LINC01734 OR4C12 ATP5F1A EREG DIRAS2 AL392023.2 ABCB5 AC091198.1 HRASLS5 DUX4L50 ANKRD36BP2 OR4Q2 MS4A15 CSE1L NEK2P2 DUX4L9 CBX3P4 ZNF630 HNRNPA1P58 AC117569.1 MANEAL AL022324.3 AC091730.1 GRAMD4 PTPA PPIAP1 PPIAP27 C1orf185 AL590640.1 LINC02312 NSUN3 LINC00158 BHLHB9 GOPC FRMPD2 AC087516.2 LINC01425 NPM1P31 BCL2L13 POLR3C AL109935.1 MIR193BHG LGALS14 CPPED1 INTS4P1 AL161636.1 RPL21P89 BNIP3P41 AC008164.1 ABHD17C ZNF292 AL121821.2 TBX15 C21orf91-OT1 AC174048.1 OSGIN2 CRISP3 ZNF521 CR383656.4 GABPA SMYD2 TMPRSS2 LINC01692 TPTE2P4 AC007333.2 AL157359.2 PPIAP8 KIF3B CARMIL3 DUXAP10 AP003973.2 AC018618.1 AC119751.5 ZNF429 CTBP2P5 ACSM5 AC027458.1 AC118282.2 AL139246.3 WDR47 CA5AP1 AC010880.1 AC011507.1 AC010374.2 KRTAP13-6P OR4A5 ZNF610 LINC01310 ELOVL2-AS1 KDR BRD9 ACTR3BP2 OR2M5 OGT PYHIN5P AC122710.1 SYNDIG1 ZDHHC11 DPF3 MOV10 TMEM45B RPL39P40 ZNF337-AS1 MS4A1 DUX4L17 IGHV3-16 TMEM51 LINC02290 AGBL3 AL357500.1 SCARA5 AC022816.1 LINC00674 AC025277.1 AC122134.1 LINC00923 AC006987.2 AC026787.1 GPR137C AL161722.3 AC022695.2 AC116553.1 AL592494.1 LINC01146 AGGF1P1 AL021877.1 C7orf66 AL627422.1 RASGRP1 IGHV3-65 AL590623.1 LINC01533 BOLL ZNF451 DCUN1D4 AP001803.2 LINC00544 AP000472.1 AC011503.1 SLC12A1 KRTAP8-3P WDR35 SMC1B RN7SKP126 GNPTAB ABCC10 GUCY1B1 RARRES2P1 TMEM173 CA10 SLC4A1AP AC244502.1 IGHV7-34-1 AL445383.1 AC097374.1 SOX8 PGAP3 AC006455.1 BCL2L1 PIP4P2 HELLS GBP6 SHISA9 OR5J2 ZNF355P SNRPN AL606495.2 AL117329.1 OR4K17 IGLV3-19 LINC00458 MIR4290HG MIR155HG AC069061.2 IGHV3-43 S100A7L2 HSH2D AC116353.2 SLX4IP YME1L1P1 OR4C15 TMEM161A FAM242A PRAMEF25 ARHGAP44 FO393415.1 LINC00707 AC111198.1 RPL23AP87 C2CD2 FAM189A2 AC090993.1 RNU6-576P IGKV2OR2-2 MAT1A AC002511.1 KCNC4 AL731574.1 AL110505.1 GNB5 AL359955.1 SLC30A5 C22orf34 AL160237.2 AC087463.1 AC010425.1 IGHV3-60 RANBP10 BACH1 LINC01053 POTEH EWSR1 AL603840.1 AF165147.1 TDRP CIZ1 AC020914.3 AC090525.1 LINC01689 AC008992.1 ZNF98 RNVU1-18 PLCD3 WSB2 PSG6 FAM227A FAM221A RNU4-45P AC023934.1 LINC00378 ADIPOR2 SART3 PABPC1 RN7SL766P EFCAB6-AS1 RNU1-131P FDXR RAB27A TBC1D2 SNRPCP1 AC023157.2 HLA-DQB2 IGKV2OR22-3 SLFN5 AC024651.2 AC018767.1 TUBGCP6 MORC2 AL132996.1 AL355493.2 CST2 AC097480.1 OR4N2 NUMA1 ZNF330 LINC01426 RN7SL659P LINC00702 RPL39P33 Z82198.3 ATE1 CEP44 AC027612.2 MYO5BP2 TTLL8 AC073314.1 OR8L1P ETV6 AL136018.1 ACTR3BP3 TESC RNU6-458P MFSD9 AC009299.1 KCNJ15 RPL21P91 ATIC LINC01224 FBXO32 FANK1 AL033381.1 AC012414.5 LINC01908 AP001979.2 AC100770.1 AMD1 SLC18B1 AP000959.1 CLIC6 TUBB1 ARMCX2 LINC02307 GTF2IP2 AL360270.3 IGF2BP1 USP3 RANGAP1 OSBPL5 LINC02126 AC073133.2 AC073530.1 RXFP2 NF1P6
RNA-Seq downregulated	101	*TRAF3IP2-AS1 BCLAF1 MAGI1-IT1 CDK1 SCP2 C1orf168 AVEN PPP2R5A EBPL GMFB UBE2W HUS1 ALG6 ZFPM2 ZNF568 LRP1B LRR1 SCFD1 GRIN3A UCHL3 ABCD3 HAS3 PM20D1 PCNXL4 ZNF493 ZNF430 MDM2 SDHAF2 ZNF268 RASGEF1B STAG1 STARD4 ABCA11P ZNF483 SHOC2 ZNF720 FILIP1L ZNF559 BRMS1L ZNF782 PPA2 ZNF615 MRPS6 CENPK ITGAE OMG CWF19L2 ZNF235 ZNF850 NBN MNAT1 TRIM23 RALGAPA1P FLVCR1 METTL8 ASCC3 ZNF879 YOD1 DICER1 USP16 LRRC4 ZNF721 LRRC69 PPIAP22 ZNF765 PTGFR UHMK1 MIR663A RAB23 BZW1 GPN3 STXBP5 MIR3648 GCNT7 SSUH2 SRP19 NEB B3GALTL CPA6 ATP5O ZNF283 IL12RB2 RN7SL23P DENND1B ABCB10 NGLY1 DNAJC2 SCARNA21 SDF2 PCDH9 CENPV CUL2 ZNF736 PTPLAD2 ATP6V1D TRIM59 RELN ZNF845 DLEU1 SUCLA2 SEMA3C*

Table S16. GO associations (Enrichr Submissions TF-Gene Cooccurrence) of 101 rDNA-contacting genes that were downregulated after heat shock treatment and kept their contacts with rDNA. Related to Venn Diagram in Figure 5B – 101 genes, and Figure 5C, enriched terms.

Index	Name	Adjusted p-value
1	*ZNF765*	3.584e-7
2	*ZNF461*	0.000001971
3	*ZNF136*	0.000007942
4	*ZNF227*	0.000007942
5	*ZNF527*	0.000007942
6	*ZNF28*	0.00002144
7	*ZNF501*	0.00002144
8	*ZNF420*	0.00002144
9	*ZNF571*	0.00002144
10	*ZNF570*	0.00002144
11	*ZNF347*	0.00002144
12	*ZNF45*	0.00002144
13	*ZNF701*	0.00002144
14	*ZNF568*	0.00002144

15	*ZNF221*	0.00002144
16	*ZNF776*	0.00002144
17	*ZNF829*	0.00002144
18	*ZNF181*	0.0001044
19	*ZNF680*	0.0001044
20	*ZNF268*	0.0001044
21	*ZNF283*	0.0001044
22	*ZNF566*	0.0001044
23	*ZNF675*	0.0001044
24	*ZNF567*	0.0001044
25	*ZNF320*	0.0001044
26	*ZNF845*	0.0001044
27	*ZNF253*	0.0001044
28	*THAP5*	0.0001044
29	*ZNF417*	0.0001044
30	*ZNF440*	0.0004915
31	*ZNF345*	0.0004915
32	*ZNF626*	0.0004915
33	*ZNF468*	0.0004915
34	*ZNF141*	0.0004915
35	*ZNF808*	0.0004915
36	*ZNF431*	0.0004915
37	*ZNF607*	0.0004915
38	*ZNF480*	0.0004915
39	*ZNF470*	0.0004915
40	*ZNF780B*	0.0004915
41	*ZNF700*	0.0004915
42	*ZNF714*	0.0004915
43	*ZNF708*	0.0004915
44	*ZNF17*	0.0004915
45	*ZNF678*	0.0004915
46	*ZNF836*	0.0004915
47	*ZNF222*	0.002464
48	*ZNF180*	0.002464
49	*ZNF302*	0.002464
50	*ZNF429*	0.002464
51	*ZNF92*	0.002464
52	*ZNF582*	0.002464
53	*ZNF585B*	0.002464
54	*ZNF529*	0.002464
55	*ZNF233*	0.002464
56	*ZNF197*	0.002464
57	*ZNF543*	0.002464
58	*ZNF471*	0.002464
59	*ZNF510*	0.002464
60	*ZNF681*	0.002464
61	*ZNF780A*	0.002464
62	*ZNF155*	0.009670
63	*ZNF140*	0.009670
64	*ZNF37A*	0.009670
65	*ZNF583*	0.009670
66	*ZNF195*	0.009670
67	*ZNF654*	0.009670
68	*ZNF230*	0.009670
69	*ZNF484*	0.009670
70	*ZNF33A*	0.009670
71	*ZNF304*	0.009670
72	*ZNF234*	0.009670
73	*ZNF773*	0.009670
74	*ZNF229*	0.009670
75	*ZNF611*	0.009670
76	*ZNF528*	0.009670
77	*ZNF208*	0.009670
78	*ZNF594*	0.009670
79	*ZNF738*	0.009670
80	*ZNF772*	0.009670
81	*ZSCAN23*	0.009670
82	*ZBTB41*	0.009670
83	*ZNF383*	0.009670
84	*ZNF506*	0.009670

85	*ZNF790*	0.009670
86	*ZNF99*	0.009670
87	*ZNF676*	0.009670
88	*ZNF677*	0.009670
89	*ZNF625*	0.009670
90	*ZNF835*	0.009670
91	*ZNF56*	0.009670
92	*ZNF432*	0.03670
93	*ZNF223*	0.03670
94	*ZBTB6*	0.03670
95	*ZNF548*	0.03670
96	*ZNF44*	0.03670
97	*ZNF585A*	0.03670
98	*ZNF254*	0.03670
99	*ZNF717*	0.03670
100	*ZNF606*	0.03670

Table S17. Overlap of rDNA-contacting genes that decrease or increase the number of their contacts with rDNA clusters and change RNA expression. Related to Venn Diagram in Figure 5D – upregulated rDNA-contacting genes.

Names	total	elements
4C increased RNA-Seq upregulated	10	*TACC2 PRKCB CLSTN2 SSBP2 EXOC4 NTRK2 PDE9A GNG4 TMOD1 SPOCK1*
4C decreased RNA-Seq upregulated	10	*CCDC88B SERPING1 RNF182 C1QTNF6 IL4R PLEKHA6 FBLN1 RPS4XP22 PABPC1 OSBPL5*
4C increased	2085	*AL590652.1 ELMO2 HPSE2 FARP1 LRRC37A5P TNIP3 PIGX AC084116.3 C10orf90 LINC01201 BBOX1 ZEB2P1 KLHL13 XDH AEN CMTM8 PTPRR ACOT12 SLC10A7 PXYLP1 ITPK1 PAK1 AC021086.1 ANGPTL5 OR2B6 CNGB1 DAZAP1 MITF RTN1 RNVU1-14 STK32C FNBP1L COL4A5 AC010280.2 ZEB1 ADCK1 TRIM33 AC005062.1 NDUFAF4 MSH3 SLC16A14 MMS22L AP003469.4 BANK1 WSCD1 UNC5A PIKFYVE OOSP4B TMEM132E FMO10P NFIA NAT10 AC007389.1 RN7SL35P TMEM184A SYN3 TSC22D1 LHFPL2 AL133445.1 NMNAT1 LINC01655 C9orf43 ELOVL5 CYP2F1 AC011498.1 CNBD2 SSX6P ACSL3 LINC01515 CDC14A UTRN AL391358.1 GRPEL1 USP6NL SDAD1 GPC5 ENOX2 LRGUK TERF2IP CD160 PDCD5 SLC8A1 LINC02487 KPNA3 ANLN CASC16 CEP350 PRR16 ZNF32-AS3 CIDECP IGKV2D-38 ARHGEF9 AC106806.2 BMT2 DYSF OR1D4 MTHFD2P1 AL360175.1 EXOC3 PPIL4 LINC01269 AC025741.1 EEF1DP6 RN7SL571P AP000253.1 DNAJC5 LINC02438 IP6K1 MIR513A2 DKFZp779M0652 AC110079.1 PRY AC092944.1 ULK4 OPHN1 DLX6-AS1 AC091173.1 SNX18P14 AC007834.1 PPIAP33 GCC2 AC087283.1 RARRES3 SORT1 TMEM255A PREP USP7 HS2ST1 CD82 MAP3K13 AC022201.1 GCLM SLC35D2 KIAA1324 SNX19 C12orf75 CHFR COL18A1 PIK3CB RNU6ATAC4P NRIR PPP1R9A SAA3P CCDC185 LINC02068 LINC02015 AC006238.1 C14orf132 RSRC1 GCK P2RY10BP PRPS2 THRB CELF1 ASS1 SLC9A9 AP003306.1 LRRC52-AS1 AC092957.1 SERF1A AC112487.1 SRP72 ZZEF1 PTPN5 RN7SL52P FOXB1 ARSH PRR29-AS1 DDHD2 LINC01270 IGSF11 CDK17 DPYD SENP7 AC008448.1 PPBP BACE2 AL359643.1 AF279873.3 AL139158.2 LINC01760 ITPRID1 USP32 QKI KDM6A AP001528.1 MED27 WNT3 LIN52 SLC25A21 PDP1 AHCYL2 AL137027.1 TPT1P14 AC011487.1 POU6F1 Z68871.1 GDAP1L1 PLD1 CHRNB2 UBN2 GLG1 NDUFC1 AC034199.1 KIF2A AC090809.1 ZNF79 ARL6IP6 L2HGDH UBXN7 RARB AC006148.1 KCNQ5 AGPS PLCXD2 ZBTB20 TARID DRAM2 AC098487.1 RF00019 HLF RNU7-182P AC004830.1 TMED9 HEPHL1 COLEC11 APOBEC3B OR1D3P NBPF3 CEP85 NOP58 AF064858.2 C19orf67 AC073370.1 COP1 NADSYN1 LINC01794 PBX1 FN1 FAM126B AC008869.1 AL031733.1 AC005261.1 DLEC1 LINC01243 CAMSAP1 PHACTR1 RBM6 SORBS2 WAPL NFE2L2 TTC41P AC025263.1 ZNF275 SPINK5 KRCC1 RPS6KA6 C1orf112 LINC01445 CDHR1 AC112176.1 TFEC PROX1 FAM71F1 HPF1 PPM1H DCX GPBP1L1 RIC3 AC023245.1 ETFA BAALC-AS1 OTOA FSTL5 AHRR AL022324.1 DEF8 EIF4E2 KLHL6 MALINC1 GUSBP1 TTC13 CRABP1 AC009005.1 LINC02107 RNU6-477P CUL4B KCTD10 NEPNP SYTL4 TRBV5-6 ATP6AP1L ADGRG4 DCTN1 AL137145.2 USP17L24 APBA2 DDX39A DIXDC1 VTI1A CADPS2 LINC02077 FAM114A2 LRRC2 FTH1P8 HAGH TRIM45 SLC2A10 PTCRA ARHGEF26 AC008080.1 CRAT37 ASTN2 RBM26 BRINP3 SGK1 AC134698.4 DDI2 LEF1-AS1 LINC01237 RF00393 AL034397.1 CSMD3 CHST10 MIR4495 PECR FAM120A TRAPPC2 DISC1FP1 FHIT AC104695.2 NFKBIZ SPAG9 GPR135 SLC39A9 LINC00348 LINC01861 NKIRAS1 RPL7AP57 TCEANC AC156455.1 AC100781.1 BARD1 USP17L18 ARHGAP25 FAM19A1 AF038458.2 TRABD2B AC009262.1 SVIL-AS1 HNRNPA1P34 AC092646.2 USH2A C1QTNF7 LRP5 LINC01378 AL772202.1 NEGR1 GALNT2 CTNNB1 ELK1P1 AC092078.2 KIAA0753 ANKRD17 USP17L19 B3GNT2P1 UBE2J1 FAM157A AC083949.1 AC134915.1 CLIP2 PRDM6 RNF150 VEZT HNRNPA1P51 SLC35B1 GK5 SIGLEC1 RN7SKP49 SOCS5P2 AC078980.1 ADD1 ZNF136 ZSCAN12 AC096773.1 LAMC3 ANP32A UBE2Q2 DNAJC1 TAC1 PGGT1B AC062028.1 ASZ1 KCNN3 DPY19L3 KIAA0232 WIF1 FBXO3 AC093730.1 LINC01339 AC104170.1 LINC02117 LINC02031 AC079779.2 AP006296.1 HERC3 SH2D3C ZBTB47 IBA57 PDE4D LINC02147 AL163642.1 GAP43 POFUT2 LINC01799 PCDH11X EDNRB-AS1 OR1L3 NTPCR C10orf143 HIPK3 PCNPP2 AL133523.1 LINC01251 HNRNPCL1 MORC1 PPP6R2 KIF9 SETBP1 MIR548A1 PITPNM2 ZBTB7C AL158825.1 ZNF780B ENOPH1 EFCAB12 RN7SL290P RUFY1 PGLYRP4 RAB9AP5 ADRA1D RNU6-144P LINC02463 ORC5 ARSA PIH1D3 BIRC2 GAS1RR*

		ZC3H12C NLGN3 AC022387.1 PLS3 EGFEM1P AC093459.1 AC090001.1 AC005244.2 PIANP AL390729.1 CKS1BP6 NLRP9 IL1RAPL1 WDPCP AC007923.4 NUF2 GAL RCC1 PRNT OR52B3P ASAH2B GNGT1 MBD2 AMOTL2 PLA2G10 LINC01111 ZFPM2-AS1 PDK4 BLK LINC02220 FARS2 HRASLS SAMD5 AC074254.1 LINC02180 SLC44A3-AS1 RERGL RPP30 GTPBP2 RPH3A GOLGA2P7 CD109 CEACAMP11 GRID1 GREB1L PRDM1 SLCO6A1 SMAP1 EFR3B BBX AVL9 AC142381.4 ARMC2 AC021074.3 ZFYVE9 FER1L6 LINC01790 SMPD4P1 TBC1D16 AC122138.1 AC006272.2 CACNA2D2 SYN2 SEPT7 KIAA1109 PTPRC LINC02299 BEST3 LINC01681 LINC01365 URB2 AL031687.1 AC005798.1 CCND3P2 AC136489.1 CNBD1 SYNPO2L CFAP70 AC007100.1 HDDC2 Z93242.2 EMCN OR4C1P CCDC162P IGHMBP2 MIR4453HG TRAV19 ZNF767P CCSER2 AL139806.1 GPC4 LMO7 AL353651.1 COL4A2 MSC-AS1 C1S CCT4P1 ZNF84 AC132938.1 BRD7P5 ME2 LINC01554 AC104009.1 STARD8 GRAMD1B FOXP2 NKAIN1 AC093716.1 SIAH2 POLR3A ADGB EXT1 CLEC4D VSTM2B ADGRE5 TCHP HEATR5A IMPG2 ZNF385C AC073316.1 MMP3 FOXJ3 SLC35F5 OR1D5 HIGD1C BCAR1P2 AC106772.1 LINC02432 RSPH14 PARP12 SYNE3 XPO6 C6orf89 AC002091.1 ANKMY1 BBS9 C9orf72 SSBP3 CYP4F22 MAP3K21 FAHD2B HACL1 LINC01182 FAM86C2P CDC27P2 AC099520.1 KLHL1 AC004540.1 CCDC3 MFGE8 AC092106.1 KIDINS220 SH3BP2 PLCB4 HSD17B3 TLR10 WDFY3 MSH4 NARF AC239859.1 WDR92 AC026765.2 LINC00476 SAMHD1 RPS4XP3 UGGT2 DHX8 TRIM4 SH3YL1 CT55 FMO3 LINC00630 TDRD3 OVCH1-AS1 KLHL15 ICMT AC010931.3 ZSCAN5B PIP4K2A FARP2 LINC01995 MAPKAPK3 PACS1 AC010320.2 AL355075.4 INTS6-AS1 UBAC2 LINC02313 SLC25A17 TTC39A RORA-AS1 UGT2B25P AL512361.1 AL139383.1 AC124657.1 CRYZL2P-SEC16B LINC01795 LINC02283 LINC00273 BAZ2B TRHR EML6 CPSF6 CENPW LINC00992 ZNRF3 AP005433.1 AL121949.2 PRSS55 LINC00858 MINPP1 EYA3 XIST PPIAP85 OR7E111P MGAT4C SH3D21 IPCEF1 CYP2AB1P AC097486.1 D2HGDH C1RL-AS1 ARFIP1 LINC00877 KRTAP13-5P HSPA12B MPP6 TBL1XR1 DBNDD1 HELB SZT2 MUC17 MGST2 CTPS1 TMEM126A AL731661.1 PDE5A NCKAP1L PPIAP5 ZNF541 RBBP8 OR5G5P TFP1 NRGN MED13 SCLT1 SMIM31 TTI1 NSUN6 AP005436.2 MOCOS UBE2H TMPRSS7 AC099398.1 CALCR AC037450.1 BTBD11 MBTD1 AC025283.2 GRID2 ZNF423 USP33 UNC13A RPS8P4 CCT3 AC105362.1 PSMG3-AS1 RNU6-1113P GUSB COPS3 DSTNP5 EHMT1 TTC29 RAX GCN1 WDR27 MED21 ZNF415 ZDHHC13 PLPPR1 SPTBN2 TOMM70 KLHL31 ATP2B4 KANSL3 HHLA3 AL807761.4 FGF5 C4orf51 OR11Q1P AC079380.1 STPG2 REPS1 CCDC85C FBXW7 PDHX OR2B7P SUCLG2-AS1 AC090791.1 TBC1D32 MTPN GSTA6P C1orf105 GOLGA6L4 HEATR6 RUNX2 PDE4B KRT18P49 ATP5F1C MAPRE2 FGF10 POLR2A KRT7 KIF13A CROCCP2 MBNL3 KRT18P59 LINC01331 DLGAP5 AC048387.1 PHEX PHF8 WAC-AS1 DPEP1 SYBU SGCD TMEM87A PDE4DIP RSPH10B L3HYPDH ZNF204P RASGRP4 RNU6-374P SCN7A CD209 TIPRL AC009107.1 PLG TYR TP53TG1 IL36RN TMEM64 LINC01115 MED12L ZDHHC21 LEKR1 VPS54 LINC02580 STS AC082650.1 TM9SF3 TMEM123 LINC01476 SHROOM4 SLC9C2 RBM41 CLSPN RGS12 RAPGEF2 NAV3 PLEKHD1 CDH7 PRRG3 PRIMPOL CHIC1 SULF1 PRDM5 PUDPP1 BUD13 UBE2E2 KIF3A SUCO AC009387.1 C13orf42 IMMP2L IL32 FTCDNL1 EPHA5-AS1 PUDP SH2D4B TRIML2 KDM1B AC010148.1 AC026336.1 FAM168A RASA1 RN7SL468P QRFPR NONO EEF1GP2 FAT1 KLHL42 MBP CPS1 ALG5 LINC01798 NCOA2 TANGO6 SLC35F2 AC024940.1 DCAKD STAM2 PIK3R1 LINC02270 HELZ TRAK1 USP12 HMGN1P11 C17orf112 ADAT1 CENPI EIF4H AC118942.1 ANKRD33B KCNQ3 AC016205.1 ABCB11 KATNAL2 AC010493.1 BOC XRCC5 CFDP1 DLG5 PLA2G16 RAB1A RPL21P39 AC099673.1 NFXL1 AC090825.1 C2orf69P3 USO1 PROX2 RUFY3 GPR63 ZHX1 NFKB1 MANBA TMEM135 LINC02522 CLCA4-AS1 RBMXP4 KDM2B MARCO NSUN7 BRD7P1 RNF8 IGF2R MIR4500HG LINC02177 OR8B5P ST6GAL1 EIF3E IL9 ZMPSTE24 RAB28 DEDD2 UBE3A RNU6-320P CCDC144NL-AS1 NSFP1 TLE4 AC010327.4 SH3GL2 ST3GAL5 MRPL23 DHRS1 ORC4 AC135506.1 DEK FAM117B VPS25P1 AGBL2 AL022722.2 DCN AP001628.1 RSL24D1P9 PLPPR4 HTR2A GRK7 EPAS1 R3HDM1 NRBF2 RSF1 KRT12 MYLK3 MKRN1 PCNX3 POU2F1 AL133163.1 CLNS1AP1 LINC02444 GALNT1 PTCHD1 AC131011.1 ANKMY2 TMLHE STX6 AP003059.2 TPR BPI TTLL5 SPINK8 ZNF734P BNC2 MARCH11 KRBA1 AC107029.1 LINC00364 STAB2 CCT7P2 RTN3P1 AUTS2 FBXL4 BCAR1P1 AC106900.2 EIPR1 PTDSS2 NFU1 UGT8 ALG1 RNF123 SFXN5 AL035413.1 CCDC178 SESTD1 SLC4A4 LINC02384 ESR2 ACOT8 LINC01847 DNAI2 SMAP2 ZBTB46 MAP7D1 CCDC148 TWIST1 SELENOT ENPP7P6 JRKL MAPK6P1 EEA1 CRB1 BCAS3 AC010105.1 ADARB2 TTTY10 SLC16A1 AL078581.2 EHHADH SLC17A5 AC026826.2 SI AL449403.1 RNU6-535P KCNE4 RPSAP27 TEX15 BAAT SLC38A11 COX10-AS1 CH25H COL12A1 TMEM206 VGLL4 CHST3 AC114781.4 ABLIM1 THORLNC TFAP2E AP002414.5 C1orf43 COLQ RBMS1 FRMPD2B PPIP5K1 GOLPH3L SYAP1 GBX1 RF00096 DCAF17 LCEP1 PDE3B MCTP1 UCK2 LINC01639 CDIP1 NPIPB1P AFF3 TET1P1 DARS2 ERBB4 SLC30A9 DNASE1 OR2T33 RBMY2JP TNIK MEF2D AC093827.1 PANX1 MICU3 WWC2 GSTA1 RNF25 AC092364.1 AC004987.3 AC092447.7 MPPED2 JPT1 CCDC59 LINC01257 KMT2E CHRDL1 MEF2C-AS1 ADD3 PTEN ARSF TAF4B FRMD3 AFF1 SUSD5 RAP1A LINC02226 ATP1A3 ERI1 XRCC6P5 TUBGCP3 UGGT1 NBEAL1 LGR6 TEX36 RAC1P8 CLGN ENPP4 SMARCC1 CLHC1 FHL3 SLC37A3 NOSIP SYNPO2 NUP210L LINC00355 LINC02465 USP20 LMNTD1 L3MBTL2 TBC1D31 LYRM2 ST6GALNAC5 CHSY1 FBXO8 XAGE5 AL513329.1 SLC25A26 DIAPH2 CRY2 PNPLA4 DIP2C TCAF1 GRM8 RNF141 LIN54 AC004869.1 AL078590.3 MAML2 TCP10 NFATC1 BIN3 CD55 LYPLA1 SSX2 LNPEP AC023796.1 ATF3 DGKH AC005840.2 RPL22P22 LINC01934 CCDC39 LINC02532 NPHP1 FBN2 SLC16A7 AC234771.2 RTN4R PHF3 NEDD4 ZNF43 OR5AU1 TPM3P4 AC111152.2 ZRANB3 RBBP9 RBM33 KLHL23 SPATA17 FMO4 AC010198.1 CNTN4 TBC1D5 GPR173 GLRX2 CACHD1 PCMT1 LINC01556 AC017037.1 LINC02534 TMEM222 NUP62CL ARNT HSPBAP1 AC114550.1 MARK1 PRKCI PRTFDC1 CABP5 ABCA1 CCDC26 AL022310.1 GSTA9P SLC8A3 AHCYP3 MBOAT1 ALKAL1 PRY2 ARFGEF3 ULK1 ZNF175 MAP3K14 PHC3 PPP1R8 TNFRSF11B GOLPH3 C12orf50 CADM2 MALRD1 RPL9P15 USP24 C6orf203 PRKG2 AC090679.1 LINC02477 FANCL AC122683.1 AC017002.3 URI1 SOS2 RPS24P8 DCLK1 AC122685.1 KCNH2 CDK2AP2 LINC02320 CAMK2D USP10 IFNG-AS1 SDHAF3 MROH8 HMCN1 AC096554.1 ZMYND8 CCDC186 CDC27P1 C9orf163 WASH8P OR52I1 DHX30 LINC01695 LINC00649 FAM135A PTPRB STRIP2 AL390760.1 RN7SKP206 LINC01412 CASC3 EPHA5 LINC01525 FAM156B AL929601.2 AP003715.1 AC012468.1 CATSPERG ITSN2 SGIP1 NECAP2 PPARG AL353678.1 AL357315.2 MMAB OSER1-DT Z93930.2 SERPINB7 EVI5 AC073264.1 CICP22 SYCP1 ISX-AS1 ATP13A4 NPSR1-AS1 CACNG4 RNU5B-2P TENM3-AS1 RASGEF1C AC055758.2 RCC2 AC090115.1 ALDH1A2 LINC00535 SHCBP1L MTTP MAPK9 THSD1 ESRRG OTX1 NYX WIPF3 PARP14 DYM TMEM165 MAGEA9 TEKT3 AL663109.1 STOX2 DNAH1 AGAP9 ABCC4 VGLL1 RNF219-AS1 TRAT1 AP000282.1 TAMM41 LLGL1 AL357060.1 LINC01942 IP6K3 FAM169A DYTN HSFY4P MAP2K6 HUWE1 POLA1 ZC3HAV1 RRAS2 FAM149A TENM1 NBPF20 SEC14L3 DDX42 HTR3B TXNRD2 AL135938.1 MYPN KIAA1217 LINC01337 AC003958.2 ODAM AL512303.1 RP1 VANGL2 TBC1D2B KRTAP9-6 AC105415.1 SUB1 ANO1 DEFB103A WWC1 SMIM12 AP000442.2 SNX4 MYO1H SELENOF AC020718.1 SENP6 GAPDHP32 VEGFC SLC1A2 XRCC6P1 ZBTB16 AC105430.1 CADPS GOLGA8A STXBP5-AS1 LNP1 AC019186.1 CCDC158 AC012213.1 LINC01228 AL590867.1 LINC01477 NGB REV3L GAPDHP64 MAML3 RSPO4 RGS7 FAM167A NYNRIN EPRS LINC01470 PAXBP1P1 GRIN2B FMO8P NFYAP1 ZNF518A LINC01892 CCDC134 C6orf99 S1PR4 PHC2 LINC01142 CA5BP1 ROBO1 ST3GAL6 TNMD ARSB MYLK4 SPATA22 AL133371.1 AMPD1 AL731556.1 CWC27 MTCYBP14 RN7SL865P RBMY2NP ANKRD11 PRKX ITPR1 AC018521.1 AL137224.1 KIF5A EIF5A2 EMX2OS SULT1C2P1 LINC00278 SERF1B CNNM4 AC010754.1 ARMCX5-GPRASP2 AC018450.1 RABGAP1 LINC01924 AC107057.1 OXR1 CAMLG AC010132.1 LHX8 AL355615.1 CIDEC AC140658.3 AC104457.1 CD300LG ENPP7P8 ACO1 WDFY4 LINC02455 CTH GRHPR SEMA7A IL15 MEI4 PRRC2C PAPPA2 STK33 ANKRD18DP KCNK1 AC008565.1 DAPK2 PFKFB3 SIN3A OSTM1 DDX18P3 PHBP21 AC005562.1 MINDY3 CRYBG3 GALNT7 AC107220.1 AC010203.1 HHIPL2 COQ3 ATP5MC2 UBXN2B ALDH7A1 CSNK1D AC008591.1 EDEM2 AP005357.1 OR7E162P RCAN3 SLC26A11 ATG13 AL157400.3 GPR149 FCHSD2 STAT6 WDR64 ZNRF2 NEXN-AS1 THOP1 ADCY8 CXXC5 AC073587.1 TP53BP1 COL25A1 OCIAD1 ELP3 AK2 ZNF787 AC099518.1 VPS13A AC009961.1 UTP23 RAB9AP2 TES AGXT2 SH3TC1

		MIGA1 C8orf34 LINC02005 AC114316.2 MLLT3 ABLIM2 JAZF1 Z83844.1 BTG4 CCDC30 ELMOD2 SHC3 CCDC130 AC020704.1 RSPH10B2 CUX2 MTUS1 AFG1L HYDIN C1orf140 AC005229.1 AC018866.1 CCDC151 AC092894.1 MGMT MCEE LINC02583 LINC02513 DDX4 TXNDC16 LINC01249 CTNNA3 SPATS2 KPNA5 AKAP4 VPS35L ZNF184 AP001836.1 RAPGEF4 HEG1 AL357055.1 PPP1R3A FBXL19 SKA1 OR5F1 VDAC1P9 LAMC1 CLTC AL441964.1 AC006296.2 PBX3 TLR3 ZNF761 MTCYBP4 PDXDC2P-NPIPB14P AC090888.3 PRICKLE2 DSG1-AS1 CSRNP3 CDC7 TRAV8-4 ANGPT1 MCCC2 HEATR1 PSMD14 AC107021.2 TMEM154 AC006372.2 MRGPRX1 AL133372.2 LINC01048 NUP210 CREB5 LRRC47 AC004522.2 AC093106.2 B3GAT2 CACNA2D3 LAMP5 SLC4A10 ADGRG6 ACSS2 BAG6 AC012574.1 AC144568.2 AL355674.1 PKN2 XPR1 ITGA4 SEC23A FERMT2 TSSC2 PHLDB1 AL139231.1 TPD52 SPESP1 DACH2 AL390860.1 RGS2 PTPRK STK11IP ZC3H13 ZNF45 GFRA3 GAPVD1 TBC1D3P4 GOLGA8M LINC01060 PSG9 CACNA1D AL390718.1 PRKAA2 SGPP2 KCNK2 SORCS1 ARMC3 KIAA2012 DNM3 AC092818.1 IL1RAPL2 LINC01555 TBL1X SPAG17 AC005614.2 CHCHD3 EDIL3 CNNM1 IGKV2-38 SS18 AC138035.1 SCYL2 ITGB1BP1 LINC02531 ZC3H7B PPP1R12B VPS37C AL592291.1 SAP130 STARD6 LINC01120 PCDH1 PLXNA2 ZDHHC15 MRPL32 TRIM67 AC087672.2 OCIAD2 TRBV7-6 AL353072.1 CLCA4 MAOB AC064862.6 LINC02430 SMURF1 DEFA7P AC111152.1 AC113386.1 BTNL8 OR2A1-AS1 BCL2L11 FOXL2NB MIR4445 PCDH11Y KLF7 AHSA1 LINC00358 GDAP2 TRBV4-1 TRAV26-1 METAP1D AC092673.1 PLCB1 AL445647.1 LINC00899 BEND2 SNPH AC084064.1 UBE2Q2P8 TAF13 BRIP1 GRID2IP CDH9 SLC7A5 CYP20A1 RPS3AP25 SORBS1 LPA NDUFAF2 AC113355.1 OR3A3 PACSIN1 LRCH2 AC113398.1 AC097510.1 RNU4-73P MND1 LINC00383 ZC3H12D MGA AC092436.2 ZNF876P SRSF10 MAGI2 PPME1 NLRP2 TNPO3 MAP3K2 STRA8 AL133346.1 AC010476.1 AC008060.3 CYP4F62P RF00411 AC004943.2 GRIK5 AL392086.1 ARRDC3 TBPL1 ERBB3 SDK1 SLC16A13 ZNF844 NIN PLCL1 RN7SL605P SLC1A4 FREM3 GALNTL6 PSAT1 GRIN1 CALU OR1A1 LINC02488 CCDC144CP RPS3AP35 TNFSF13B DENND3 PUS7 AC007861.1 AC004840.2 XPA AC073172.2 MMAA INTS9 AC123905.1 GRM5 AC099508.2 PAMR1 IRF5 HNRNPM ARL14EP RPL31P40 DIAPH1 OPRD1 AC040963.1 ELOVL6 AL359837.1 COMMD10 AC016708.1 SACM1L MBD3L1 CX3CL1 ADGRL3 DLX3 UBE2E1 SLC30A10 NECAB1 LINC02149 IGF1R COL14A1 LRRC4B EFEMP2 PPDPFL RPL21P12 PCM1 TSG101 LINC00942 SLC20A2 AC007879.1 PAQR5 AC019197.1 JAM2 NPIPB10P ITGA9-AS1 SMIM36 AC025947.1 NCAPD2 OTUD6A PPM1L SMOC1 ABCB4 SLC25A33 FAM185A CNTNAP2 FRK AC117440.1 PARL TOGARAM1 AC018742.1 RYR2 RNVU1-15 AL049697.1 RNU6-1047P SLFN13 LINC02615 CBLN4 ZFYVE26 KCNIP4 RTP4 TBX20 AC011287.1 DST DZIP3 AC131211.1 CD48 RNU6-1032P RBMXP2 LNX2 ERP44 LINC02516 FGD5 AFMID AC022166.1 AC093599.1 JMJD1C EIF4E3 KCNH5 MAGEA1 NOS1AP RUSC2 AC003035.1 BAZ1A RBMY2KP TMC3 ZCCHC8 INPP5D KLHL29 OTOGL CFAP161 ZFAND3 AC118757.1 TCERG1L OPRM1 ACER2 LINC02475 ZFP64 IFNA4 CALML3-AS1 KIF13B CNKSR1 MPDZ SDHC EYS IL16 LINC02235 LINC01505 UBTFL10 ZNF517 HMGCLL1 XKR6 LINC02382 DOCK11P1 ACVR1B CD96 AC011890.1 CCDC15 AC087241.3 OR6C66P ROBO2 FAM83D OXNAD1 AC090833.1 FAM122C PDCD11 COQ8B NHSL1 RBMX MPP3 LINC00430 DTHD1 AC093802.1 FLT1P1 NT5C2 AC110995.1 NCALD OAF GAS7 AL122019.1 SLITRK1 AC092809.4 CLEC16A MTSS1 DYRK1A PPFIA1 RAB11A PROX1-AS1 HBS1L PPP2R2B DENND4C JAK1 AC078785.1 GPD2 CDK19 ITGA9 ZNF800 GALNT11 SCUBE2 ACSL6 RF00416 PITPNM3 EI24P2 SDR42E2 KCNB1 LYRM4 CCND2 CUL4A AC137579.1 LINC02099 HSP90AB3P PCP4 PTPN3 AC025260.1 FAM217B AC093426.1 RP1L1 GPRIN3 GNPAT AC093801.1 MYPOP RAP2C-AS1 PCSK5 LINC02386 CTPS2 TJP2 PGBD2 AC113208.2 AC003958.1 AL357079.1 GUSBP2 PAX3 TXNRD3 LINC01510 AL391361.2 AL356218.1 MYL6P3 SSX2IP LINC01748 PLB1 TTC17 TAPT1 ENC1 KYAT3 KCNMB2 POMT2 HPS5 AC092978.1 ST13 PTP4A1 MARS TOPAZ1 CFHR3 BMPR1B GTF3C6 VOPP1 RPL12P41 AL139344.1 OR7E109P AC245291.1 PSMA8 NLGN4Y DEFB103B AC073333.1 AL359317.1 MIOS AC096644.2 TDO2 AC096669.1 NAA25 LINC01234 CLUAP1 RNF19A RNU6-65P AC104071.1 AL591074.1 AC242426.1 SPATC1L AC010884.1 GMPS LINC00886 GPT2 AC034231.1 RABL2A NPS RN7SL761P GTF3C1 RNU6-364P DOCK3 CES4A USP17L17 AC142381.3 NDUFA10 PDSS2 SEPHS2 CCDC92 ETNK1 PRAME TEPP LARS2 U91319.1 B3GALNT2 CLNS1A NUP43 GPC6 CYP4F30P BCAT1 KBTBD12 GGTA1P WNK1 SLC2A9 ARL17A ADGRV1 SPRYD3 SCN2B ZNF398 MAMDC2 PDIA4 CAPN7 ZSCAN22 AC024909.1 AC073311.1 LINC00845 LINC02379 AC079950.1 CYP4F11 ZKSCAN7-AS1 KDM3A NOTCH3 TULP3 APBB1IP AL390962.1 ADGRG7 TRIM15 SOCS5P1 AC009313.1 RNA5SP45 ANP32B TAOK1 UNC45A RNU6-111P RPL3P7 PPP1R26-AS1 CASK NUDCD1 RASSF6 FLNA AL359853.2 TRBV5-1 AC119396.1 AC009093.2 AC138915.2 RNU6-641P SNAP91 DAPK1 MAP3K19 AC011474.1 RFTN2 AL161727.1 AF121898.1 AJ239322.1 LCE4A ALDH9A1 TTC1 SUSD1 AC092620.1 RNA5SP443 LRP11 PDLIM1P2 SIN3B OR51P1P MARCH1 EIF4A1P7 NPTXR FO393414.2 TP63 SEC22A GATA4 LMBR1 AC241644.2 ELMOD1 ZPBP SLC25A43 RETREG1 KIAA0825 SMAD5 GARS FAM49B DNAJC13 LINC00470 CNST LINC02208 IGLV3-4 TCP10L2 VPS13B FRMD4B PFDN2 AP001351.1 AC125603.2 CYP2R1 CRISP1 RF01210 RPS4XP18 RAB7A BIRC3 RNLS ZNHIT6 RNU6-830P DNAJC10 RNGTT RNF169 LINC01478
4C decreased	934	*AC092115.3 RNU7-66P CMTR2 AL031601.1 KRTAP4-12 AC137499.1 GLT1D1 AL589182.1 CYP39A1 LDB3 TMC4 EPB41L4B AP005901.1 CHCHD2P4 HS3ST3A1 KLHL14 ARL2BPP8 BTN2A1 LINC00520 SP140L G2E3 AC008667.3 AC004656.1 LINC01491 LRRC37A4P RNF185 MAGEB3 CXADR POLR3K POTED LINC02125 CCDC34 KIAA1210 RF00426 CECR2 LINC01915 LINC01701 LINC01441 AC133065.1 AL356800.1 ZNF257 C10orf120 GPR78 AC138701.1 GTF2IP4 ANGPT4 VAT1L AL031674.1 LINC01410 AC011193.1 AP003108.2 ABCD1P3 CHST15 PKIA-AS1 LINC02499 AC010615.4 AC024475.4 AP000547.1 ASIP CDH10 KL LINC01789 SAMSN1 SPANXN4 OR7E25P AL049812.2 LINC02250 TF FGF7P2 OR10T1P AP001341.1 ZNF331 POTEG AL021937.3 OR10J6P AKR1B15 RF00421 MIR548XHG PWRN3 CDC14C COX10 CENPU LINC01502 TMEM11 KRTAP29-1 MYH13 GNB4 LINC02284 AP005212.1 MMP27 BDH2P1 ACTR5 AGGF1P3 HK3 LCA5L GANC ZNF37CP FAM225B ZNF583 LINC01058 AC084373.1 OR4C9P AL136968.2 HS1BP3 AF064860.2 TRAV8-7 LINC02309 AC044784.2 PLSCR4 STRIP1 MSNP1 AC091826.2 AC012322.1 AL031289.1 GPX1P2 VRK2 AP006748.1 NDRG2 LINC00113 PHYKPL HPYR1 LINC01432 AL133173.1 LINC00960 ZNF962P ZNF384 AC026741.1 PSMC4 ZMYM5 AC089987.1 OR11L1 RCAN1 FAM193A EPHA7 LINC00408 OR4A9P EDDM3B SIRPG RAB5A AL355607.2 KANK3 AC006206.2 LINC01070 MIR17HG JRK AGGF1P2 SNX18P9 ZNF840P ANKRD20A5P AC037471.2 ARR3 AC016930.1 IL20RA LINC01194 GAS8 LCE2B LINC01907 AL162632.2 TRPM2 FRG1-DT LINC00507 S100B GABRG3 AL356095.1 AL137845.2 STRN3 CILP2 CMKLR1 LINC00575 TNFRSF19 NAP1L6 AC092423.1 AP000705.1 BMS1 MXRA5 AC124944.2 KRT74 SPINT4 AL121718.1 AL352984.1 RNA5SP519 AC016737.1 AL132708.1 ITGA2 RNU1-142P RPS3AP41 ZNF965P MBL1P AC068446.2 RNU4ATAC8P AP001605.1 IGHVIII-2-1 AC062021.1 NIPA2 OR4C14P AC119751.4 HUNK AP001574.1 LINC02311 GBP4 AC068643.1 MAPK1IP1L NEURL1-AS1 RPS23P5 ISCA1P3 REEP1 PQLC3 MKKS CHMP4C BCL2A1 AC140658.7 CR1 AC026434.2 AC009271.1 LINC02409 DTX4 AJAP1 USP40 SNX19P2 AC021439.1 IGHVIII-26-1 HFM1 AL121999.1 GSG1L STK24 TMEM231P1 NCOA5 RNA5SP478 AC099654.1 AC106871.1 AC005580.1 RN7SL568P SOGA1 NUTF2 PAGE1 TPT1P2 AC093074.1 AC092681.1 AC114501.2 EIF4E AF064858.1 AL669831.5 BPIFB1 STARP1 ZFAND1 LINC00320 AL109618.1 KLHDC7A TUNAR ERCC4 FAM83B OR4K15 NCOR1P1 NF1P4 ENTHD1 LINC02305 GADD45A AC018730.2 C5orf51 MOV10L1 RCHY1 AP000547.2 AL356276.2 CR383656.6 NF1P1 FAM201B NLK AC079062.1 NIPA2P2 IPPK AC063952.1 AP000534.2 MYEOV TMED8 OR5L2 BNIP3P6 PRIMA1 LINC02277 POLR2F KRTAP5-8 TMPRSS3 ATP1A4 AP003351.1 AC130464.1 RNF152 MEOX2 CEMIP DUX4L19 KCNG3 LINC00365 AC025884.1 OR4S2 LINC01495 AK6P2 AL139348.1 TMEM52B SH2D7 LINC01268 LINC01094 DUSP27 AC097493.3 OR8K4P NANOGNBP2 CYTIP AC099793.1 AC073325.1 AC020595.1 AL139393.1 AP000233.2 DPP9 AC008608.1 ABCD1P4 AC009567.1 GLDC CHEK2 SUZ12 LINC01684 AC118282.3 BLZF2P AF121897.1 ZNF729 SIGLEC30P TSPY5P AC005185.1 RAB22A AL158090.1 PJA1 DDX21 ANKRD20A9P ZNF688 PWP1 AL358292.1 ILDR1 GRXCR1 TTC22*

		STXBP4 OR5D15P AC008794.1 MED15 ZBED4 MT1HL1 INTS13 ZER1 LINC01648 OR4C2P OR52X1P AC091193.1 AC087883.1 AL049651.1 ISX RAD51AP1 FAM182A POTEM AC092966.1 LINC01687 LRRC1 CR383658.1 SEC14L6 CYB5R2 DET1 AL022332.1 ANKRD20A11P LINC01020 ACTBP8 AC007848.1 POTEB2 AC027612.4 SDR16C6P TM4SF1 ARHGAP27P1-BPTFP1-KPNA2P3 AL589743.2 AC060788.1 LINC02322 PNLIPRP3 ZMYM2 TAB3 AC069545.1 AC145543.1 RF00156 AC016044.1 ACTR3BP5 AC097532.1 AL365232.1 ERMN TRIM48 LCP1 AC013460.1 Z82249.1 RMRPP5 WBP11P1 ADCY2 KRT25 NOVA1 AC009169.1 MRPL58 GNG5P5 LINC01227 FRG1BP IATPR SH3BGR AC098826.1 SPRR2G TMEM211 AL121821.1 LINC02089 LINC02101 TRDV1 RPL21P41 ABCC13 AL663058.1 LINC02347 MARK2P9 ATP8B5P LINC02082 IGHV1OR15-9 ZNF91 PATE4 HACD4 PMP22 FAM111B AL137009.1 LINC01683 AC097532.2 PLEKHA5 AC023078.2 WRB CIB4 AP000470.1 LINC00317 POTEB CDC20B LINC00347 AL773545.1 AC068722.1 TFF1 SYNM AC025857.1 GLP2R MAP2K3 AL020994.2 IGKV1-5 AC126603.1 ANKRD34C AC245028.2 KMT2C MT2P1 DIO3OS IFNWP4 AC140847.2 LINC01644 OR6K4P C14orf177 AC005863.1 COMT AC018904.1 SNX19P1 IGHV3-76 AL353633.1 AC009081.1 CD1C AL355516.1 DUX4L16 RNU1-51P RF01182 AC106785.2 UGCG ORC3 AL035250.1 VWFP1 CHST8 SLC9B1P3 AL512310.1 RGMB LINC00839 AP000265.1 AL117190.1 HSPB8 LINC00884 AC090618.1 PXT1 RLBP1 KLRF2 LINC00308 CYCSP6 FP325330.1 AL049875.1 ZBTB34 ZNF826P KCNE1 ITLN1 TRMT61B AC015771.1 MAGEC3 ZSCAN5A FPR3 AC009652.1 TSPAN3 DSCR8 CYLC2 AL049775.2 TPP2 AC007333.1 RPL12P12 AC097501.2 BTNL9 LINC01920 URB1 AC108047.1 AC006273.1 BASP1 GRK6 BMPR1A RPL23AP12 ABCG1 CRLS1 SQSTM1 FAM207CP ABHD4 AL353148.1 AC068760.1 AL158042.1 DEFB116 CFAP46 OR7E104P KRT8P25 AL359081.1 LINC01262 LINC01203 SLC16A12 SOCS6 AKIRIN2 LINC00929 SOD3 CSF1 AC005476.2 LINC01603 AL121852.1 GRK3 RNA5SP495 AC092175.1 ANKRD30BP1 MPPED1 FO393418.1 AC090136.2 AF241725.1 LINC01809 PWRN2 CYCSP51 GPATCH2L AC087341.1 PAXIP1-AS2 AC005307.1 ETS2 FCRL3 CECR7 CTBP2P4 OVGP1 ARID5B RPL26P28 AL157756.1 AC105148.1 HIRA AC016573.1 RF00012 AL772155.1 CR383656.10 TMPRSS6 SERPINA1 MYBL2 RBM19 ZNF527 FEM1AP1 AL110503.1 CDR2 LINC01229 PSMC6 AC119751.1 AC026495.1 ITGB7 D21S2088E ZSCAN10 BRF1 NLRP13 ACTR3BP6 MANSC4 AF212831.1 WT1 OR4C5 MAPK14 AC023824.4 DYNLL2 PRR14L KSR1 AC138123.1 CFL1P6 TUFT1 AC006548.2 MTFMT RAF1 AP001116.1 AC009139.2 AC142384.1 EDN3 ROCK1P1 GRPEL2P1 PRLH AP000235.1 CR786580.1 PSMB2 SNRPD1 CST9L LINC02200 LINC02485 AP000542.1 AC132825.3 LINC01938 CYCSP17 RPL12P27 DMBT1P1 DIO2-AS1 HBD KBTBD11 REV1 GALC AL607077.1 AC245748.2 PWRN1 WDR4 AL137792.1 LINC01706 LINC01734 OR4C12 ATP5F1A EREG DIRAS2 AL392023.2 ABCB5 AC091198.1 HRASLS5 DUX4L50 ANKRD36BP2 OR4Q2 MS4A15 CSE1L NEK2P2 DUX4L9 CBX3P4 ZNF630 HNRNPA1P58 AC117569.1 MANEAL AL022324.3 AC091730.1 GRAMD4 PTPA PPIAP1 PPIAP27 C1orf185 AL590640.1 LINC02312 NSUN3 LINC00158 BHLHB9 GOPC FRMPD2 AC087516.2 LINC01425 NPM1P31 BCL2L13 POLR3C AL109935.1 MIR193BHG LGALS14 CPPED1 INTS4P1 AL161636.1 RPL21P89 BNIP3P41 AC008164.1 ABHD17C ZNF292 AL121821.2 TBX15 C21orf91-OT1 AC174048.1 OSGIN2 CRISP3 ZNF521 CR383656.4 GABPA SMYD2 TMPRSS2 LINC01692 TPTE2P4 AC007333.2 AL157359.2 PPIAP8 KIF3B CARMIL3 DUXAP10 AP003973.2 AC018618.1 AC119751.5 ZNF429 CTBP2P5 ACSM5 AC027458.1 AC118282.2 AL139246.3 WDR47 CA5AP1 AC010880.1 AC011507.1 AC010374.2 KRTAP13-6P OR4A5 ZNF610 LINC01310 ELOVL2-AS1 KDR BRD9 ACTR3BP2 OR2M5 OGT PYHIN5P AC122710.1 SYNDIG1 ZDHHC11 DPF3 MOV10 TMEM45B RPL39P40 ZNF337-AS1 MS4A1 DUX4L17 IGHV3-16 TMEM51 LINC02290 AGBL3 AL357500.1 SCARA5 AC022816.1 LINC00674 AC025277.1 AC122134.1 LINC00923 AC006987.2 AC026787.1 GPR137C AL161722.3 AC022695.2 AC116553.1 AL592494.1 LINC01146 AGGF1P1 AL021877.1 C7orf66 AL627422.1 RASGRP1 IGHV3-65 AL590623.1 LINC01533 BOLL ZNF451 DCUN1D4 AP001803.2 LINC00544 AP000472.1 AC011503.1 SLC12A1 KRTAP8-3P WDR35 SMC1B RN7SKP126 GNPTAB ABCC10 GUCY1B1 RARRES2P1 TMEM173 CA10 SLC4A1AP AC244502.1 IGHV7-34-1 AL445383.1 AC097374.1 SOX8 PGAP3 AC006455.1 BCL2L1 PIP4P2 HELLS GBP6 SHISA9 OR5J2 ZNF355P SNRPN AL606495.2 AL117329.1 OR4K17 SNHG17 IGLV3-19 LINC00458 SRD5A3-AS1 MIR4290HG MIR155HG AC069061.2 IGHV3-43 S100A7L2 HSH2D AC116353.2 SLX4IP YME1L1P1 ZNF569 OR4C15 TMEM161A FAM242A PRAMEF25 ARHGAP44 FO393415.1 LINC00707 AC111198.1 RPL23AP87 C2CD2 FAM189A2 AC090993.1 RNU6-576P IGKV2OR2-2 MAT1A AC002511.1 KCNC4 AL731574.1 AL110505.1 GNB5 AL359955.1 SLC30A5 C22orf34 AL160237.2 AC087463.1 AC010425.1 IGHV3-60 RANBP10 BACH1 LINC01053 POTEH EWSR1 AL603840.1 AF165147.1 TDRP CIZ1 AC020914.3 AC090525.1 LINC01689 AC008992.1 ZNF98 RNVU1-18 PLCD3 WSB2 PSG6 FAM227A FAM221A RNU4-45P AC023934.1 LINC00378 ADIPOR2 SART3 RN7SL766P EFCAB6-AS1 RNU1-131P FDXR RAB27A TBC1D2 SNRPCP1 AC023157.2 HLA-DQB2 IGKV2OR22-3 SLFN5 AC024651.2 AC018767.1 TUBGCP6 MORC2 AL132996.1 AL355493.2 CST2 AC097480.1 OR4N2 NUMA1 ZNF330 LINC01426 RN7SL659P LINC00702 RPL39P33 Z82198.3 ATE1 CEP44 AC027612.2 MYO5BP2 TTLL8 AC073314.1 OR8L1P ETV6 AL136018.1 ACTR3BP3 TESC RNU6-458P MFSD9 AC009299.1 KCNJ15 RPL21P91 ATIC LINC01224 FBXO32 FANK1 AL033381.1 AC012414.5 LINC01908 AP001979.2 AC100770.1 AMD1 SLC18B1 AP000959.1 CLIC6 TUBB1 ARMCX2 LINC02307 GTF2IP2 AL360270.3 IGF2BP1 USP3 RANGAP1 LINC02126 AC073133.2 AC073530.1 RXFP2 NF1P6
RNA-Seq upregulated	123	*PKNOX2 AMZ2 ASNSP1 AK7 DKK2 PIK3R5 RASGRF1 FNDC1 MAN2B1 UBE2L6 ADAMTS2 PPARGC1A ZDHHC11B PHF21B PCBP3 COL23A1 MAP7 LRRC16B DGCR5 DENND2A DICER1-AS1 GNG2 CD72 PDZD7 ULBP1 PARK2 MTND6P3 AMPD3 ADCYAP1R1 LSAMP KCNQ1 RPS6KA2 C2orf48 IGSF21 GRAMD1C SHC2 PCED1B PHC1 AK8 PALMD MTATP8P1 PAAF1 TP53I11 TOX2 TMEM108 NHS SHANK2 IQSEC3 SH3BP1 CRYL1 ASTN1 NSG1 PTPRU NHSL2 SHOX2 COL24A1 ELK3 TTC4 ROR1 EPHB2 MTND6P4 C1orf106 IER2 PTPRE PPM1J FAM129A TGM2 PHACTR3 DAB1 RIPPLY3 C9orf171 SCUBE1 KIAA1407 SGSM1 JAKMIP1 SH3KBP1 CDC42BPG SLIT3 KXD1 EGFLAM ABI3BP PLEKHH2 GAB2 hsa-mir-6723 SLC7A8 SPATA7 S100A11 RNF165 GLIS1 ACSS1 TNC THAP7-AS1 NFASC LOXL2 SLC2A12 SYT6 ADORA1 LINC00617 KHDRBS3 NR5A2 BRSK2 ANKRD29 LIPE-AS1 CAMK1D CLYBL DEPTOR PPFIBP2 COBL SARDH SYT17 NES TIMP2 SNX24 SPARC ANXA4 SYT9 JPH3 HSPG2 TRAP1 C8orf44 MAMLD1 FBLN5*

Table S18. GO associations of rDNA-contacting genes that were upregulated after heat shock treatment and increase their contacts with rDNA. Related to Venn Diagram in Figure 5D – 10 genes.

GO.ID	Description	padj	Genes
GO:0097458	neuron part	0.0084	PRKCB, CLSTN2, EXOC4, NTRK2,PDE9A,SPOCK1

GO:0045202	synapse	0.0113	PRKCB, CLSTN2, EXOC4, NTRK2,SPOCK1
GO:0014069	postsynaptic density	0.0478	CLSTN2,NTRK2,SPOCK1
KEGG:04727	GABAergic synapse	0.0408	PRKCB,GNG4
WP:WP4223	Ras Signaling	0.0489	PRKCB,NTRK2,GNG4

Table S19. GO associations (Enrichr Submissions TF-Gene Cooccurrence) of 123 rDNA-contacting genes that were upregulated after heat shock treatment and kept their contacts with rDNA. Related to Venn Diagram in Figure 5D – 123 genes, and Figure 5E, enriched terms.

Index	Name	Adjusted p-value
1	ADAMTS17	0.00001638
2	PRDM16	0.00001638
3	SCRT2	0.00001638
4	MEIS3	0.0001002
5	HIC1	0.0002716
6	DMRTB1	0.0002716
7	PRRX1	0.0002716
8	FBN1	0.0002716
9	TBX18	0.0002716
10	GLIS1	0.0002716
11	LHX4	0.0002716
12	GLI2	0.0008787
13	HIVEP3	0.0008787
14	NFATC4	0.0008787
15	EBF4	0.0008787
16	ALX4	0.0008787
17	ELF4	0.0008787
18	HES3	0.0008787
19	ZBTB7C	0.0008787
20	PAX7	0.0008787
21	HEYL	0.0008787
22	GRM6	0.0008787
23	ZNF536	0.0008787
24	DPF3	0.002737
25	GLIS2	0.002737

Table S20. GO associations of genes that are co-regulated by 123 rDNA-contacting genes shown in Figure 5 E (enriched terms).

GO.ID	Description	padj	Genes

**GO:MF**			
GO:0000981	DNA-binding transcription factor activity, RNA polymerase II-specific	3.0020889186981556e-16	PRDM16,SCRT2,MEIS3,HIC1,DMRTB1,PRRX1,TBX18,GLIS1,LHX4,GLI2,HIVEP3,NFATC4,EBF4,ALX4,ELF4,HES3,ZBTB7C,PAX7,HEYL,ZNF536,GLIS2
GO:0043565	sequence-specific DNA binding	6.766317740279593e-16	PRDM16,SCRT2,MEIS3,HIC1,DMRTB1,PRRX1,TBX18,GLIS1,LHX4,GLI2,NFATC4,EBF4,ALX4,ELF4,HES3,PAX7,HEYL,ZNF536,GLIS2
GO:0003700	DNA-binding transcription factor activity	1.6218692524044966e-15	PRDM16,SCRT2,MEIS3,HIC1,DMRTB1,PRRX1,TBX18,GLIS1,LHX4,GLI2,HIVEP3,NFATC4,EBF4,ALX4,ELF4,HES3,ZBTB7C,PAX7,HEYL,ZNF536,GLIS2
GO:0140110	transcription regulator activity	5.730692260911566e-14	PRDM16,SCRT2,MEIS3,HIC1,DMRTB1,PRRX1,TBX18,GLIS1,LHX4,GLI2,HIVEP3,NFATC4,EBF4,ALX4,ELF4,HES3,ZBTB7C,PAX7,HEYL,ZNF536,GLIS2
GO:0003677	DNA binding	1.078772693704691e-10	PRDM16,SCRT2,MEIS3,HIC1,DMRTB1,PRRX1,TBX18,GLIS1,LHX4,GLI2,HIVEP3,NFATC4,EBF4,ALX4,ELF4,HES3,PAX7,HEYL,ZNF536,GLIS2
GO:0003676	nucleic acid binding	5.094046931162048e-10	ADAMTS17,PRDM16,SCRT2,MEIS3,HIC1,DMRTB1,PRRX1,TBX18,GLIS1,LHX4,GLI2,HIVEP3,NFATC4,EBF4,ALX4,ELF4,HES3,ZBTB7C,PAX7,HEYL,ZNF536,DPF3,GLIS2
GO:0000976	transcription regulatory region sequence-specific DNA binding	3.1980974069268857e-9	SCRT2,PRRX1,TBX18,GLIS1,GLI2,NFATC4,EBF4,ALX4,ELF4,HES3,HEYL,ZNF536,GLIS2
GO:1990837	sequence-specific double-stranded DNA binding	6.201300596296805e-9	SCRT2,PRRX1,TBX18,GLIS1,GLI2,NFATC4,EBF4,ALX4,ELF4,HES3,HEYL,ZNF536,GLIS2
GO:0044212	transcription regulatory region DNA binding	1.717308909160837e-8	SCRT2,PRRX1,TBX18,GLIS1,GLI2,NFATC4,EBF4,ALX4,ELF4,HES3,HEYL,ZNF536,GLIS2
GO:0001067	regulatory region nucleic acid binding	1.7651201530658695e-8	SCRT2,PRRX1,TBX18,GLIS1,GLI2,NFATC4,EBF4,ALX4,ELF4,HES3,HEYL,ZNF536,GLIS2
GO:0003690	double-stranded DNA binding	2.223504167721871e-8	SCRT2,PRRX1,TBX18,GLIS1,GLI2,NFATC4,EBF4,ALX4,ELF4,HES3,HEYL,ZNF536,GLIS2
GO:0000977	RNA polymerase II regulatory region sequence-specific DNA binding	3.2487899485312494e-8	SCRT2,PRRX1,GLIS1,GLI2,NFATC4,EBF4,ALX4,ELF4,HES3,HEYL,ZNF536,GLIS2
GO:0001012	RNA polymerase II regulatory region DNA binding	3.510295518605051e-8	SCRT2,PRRX1,GLIS1,GLI2,NFATC4,EBF4,ALX4,ELF4,HES3,HEYL,ZNF536,GLIS2
GO:1901363	heterocyclic compound binding	0.0000018894696087891545	ADAMTS17,PRDM16,SCRT2,MEIS3,HIC1,DMRTB1,PRRX1,TBX18,GLIS1,LHX4,GLI2,HIVEP3,NFATC4,EBF4,ALX4,ELF4,HES3,ZBTB7C,PAX7,HEYL,ZNF536,DPF3,GLIS2
GO:0097159	organic cyclic compound binding	0.0000025120194207196536	ADAMTS17,PRDM16,SCRT2,MEIS3,HIC1,DMRTB1,PRRX1,TBX18,GLIS1,LHX4,GLI2,HIVEP3,NFATC4,EBF4,ALX4,ELF4,HES3,ZBTB7C,PAX7,HEYL,ZNF536,DPF3,GLIS2
GO:0000978	RNA polymerase II proximal promoter sequence-specific DNA binding	0.00011929626986549908	SCRT2,PRRX1,GLI2,NFATC4,ELF4,HES3,HEYL,ZNF536
GO:0000987	proximal promoter sequence-specific DNA binding	0.00014630761558464278	SCRT2,PRRX1,GLI2,NFATC4,ELF4,HES3,HEYL,ZNF536
GO:0001227	DNA-binding transcription repressor activity, RNA polymerase II-specific	0.006154396615738856	SCRT2,HIC1,GLIS1,NFATC4
GO:0046872	metal ion binding	0.03598926908109106	ADAMTS17,PRDM16,SCRT2,HIC1,DMRTB1,FBN1,GLIS1,LHX4,GLI2,HIVEP3,EBF4,ZBTB7C,ZNF536,DPF3,GLIS2
GO:0003712	transcription coregulator activity	0.037118154816446446	PRDM16,PRRX1,TBX18,NFATC4,HES3,HEYL
GO:0001216	DNA-binding transcription activator activity	0.03790861793015616	GLIS1,LHX4,EBF4,ALX4
GO:0001228	DNA-binding transcription activator activity, RNA polymerase II-specific	0.03790861793015616	GLIS1,LHX4,EBF4,ALX4
GO:BP			
GO:0043169	cation binding	0.04607857218181967	ADAMTS17,PRDM16,SCRT2,HIC1,DMRTB1,FBN1,GLIS1,LHX4,GLI2,HIVEP3,EBF4,ZBTB7C,ZNF536,DPF3,GLIS2
GO:0006357	regulation of transcription by RNA polymerase II	3.270836093066829e-14	PRDM16,SCRT2,MEIS3,HIC1,DMRTB1,PRRX1,FBN1,TBX18,GLIS1,LHX4,GLI2,HIVEP3,NFATC4,EBF4,ALX4,ELF4,HES3,ZBTB7C,PAX7,HEYL,ZNF536,DPF3,GLIS2
GO:0006366	transcription by RNA polymerase II	1.150184066512155e-13	PRDM16,SCRT2,MEIS3,HIC1,DMRTB1,PRRX1,FBN1,TBX18,GLIS1,LHX4,GLI2,HIVEP3,NFATC4,EBF4,ALX4,ELF4,HES3,ZBTB7C,PAX7,HEYL,ZNF536,DPF3,GLIS2
GO:0006355	regulation of transcription, DNA-templated	1.9630606469452975e-11	PRDM16,SCRT2,MEIS3,HIC1,DMRTB1,PRRX1,FBN1,TBX18,GLIS1,LHX4,GLI2,HIVEP3,NFATC4,EBF4,ALX4,ELF4,HES3,ZBTB7C,PAX7,HEYL,ZNF536,DPF3,GLIS2
GO:1903506	regulation of nucleic acid-templated	2.7459416954715368e-11	PRDM16,SCRT2,MEIS3,HIC1,DMRTB1,PRRX1,FBN1

	transcription		,TBX18,GLIS1,LHX4,GLI2,HIVEP3,NFATC4,EBF4,ALX4,ELF4,HES3,ZBTB7C,PAX7,HEYL,ZNF536,DPF3,GLIS2
GO:2001141	regulation of RNA biosynthetic process	2.8901419233767096e-11	*PRDM16,SCRT2,MEIS3,HIC1,DMRTB1,PRRX1,FBN1,TBX18,GLIS1,LHX4,GLI2,HIVEP3,NFATC4,EBF4,ALX4,ELF4,HES3,ZBTB7C,PAX7,HEYL,ZNF536,DPF3,GLIS2*
GO:0006351	transcription, DNA-templated	5.773081680136585e-11	*PRDM16,SCRT2,MEIS3,HIC1,DMRTB1,PRRX1,FBN1,TBX18,GLIS1,LHX4,GLI2,HIVEP3,NFATC4,EBF4,ALX4,ELF4,HES3,ZBTB7C,PAX7,HEYL,ZNF536,DPF3,GLIS2*
GO:0097659	nucleic acid-templated transcription	7.34018708432297e-11	*PRDM16,SCRT2,MEIS3,HIC1,DMRTB1,PRRX1,FBN1,TBX18,GLIS1,LHX4,GLI2,HIVEP3,NFATC4,EBF4,ALX4,ELF4,HES3,ZBTB7C,PAX7,HEYL,ZNF536,DPF3,GLIS2*
GO:0032774	RNA biosynthetic process	8.143580839718736e-11	*PRDM16,SCRT2,MEIS3,HIC1,DMRTB1,PRRX1,FBN1,TBX18,GLIS1,LHX4,GLI2,HIVEP3,NFATC4,EBF4,ALX4,ELF4,HES3,ZBTB7C,PAX7,HEYL,ZNF536,DPF3,GLIS2*
GO:0051252	regulation of RNA metabolic process	1.191622561925827e-10	*PRDM16,SCRT2,MEIS3,HIC1,DMRTB1,PRRX1,FBN1,TBX18,GLIS1,LHX4,GLI2,HIVEP3,NFATC4,EBF4,ALX4,ELF4,HES3,ZBTB7C,PAX7,HEYL,ZNF536,DPF3,GLIS2*
GO:2000112	regulation of cellular macromolecule biosynthetic process	3.4923679876930036e-10	*PRDM16,SCRT2,MEIS3,HIC1,DMRTB1,PRRX1,FBN1,TBX18,GLIS1,LHX4,GLI2,HIVEP3,NFATC4,EBF4,ALX4,ELF4,HES3,ZBTB7C,PAX7,HEYL,ZNF536,DPF3,GLIS2*
GO:0019219	regulation of nucleobase-containing compound metabolic process	5.640327061571119e-10	*PRDM16,SCRT2,MEIS3,HIC1,DMRTB1,PRRX1,FBN1,TBX18,GLIS1,LHX4,GLI2,HIVEP3,NFATC4,EBF4,ALX4,ELF4,HES3,ZBTB7C,PAX7,HEYL,ZNF536,DPF3,GLIS2*
GO:0045893	positive regulation of transcription, DNA-templated	6.239367831655941e-10	*PRDM16,MEIS3,PRRX1,GLIS1,LHX4,GLI2,HIVEP3,NFATC4,EBF4,ALX4,ELF4,HES3,ZBTB7C,PAX7,HEYL,DPF3,GLIS2*
GO:0010556	regulation of macromolecule biosynthetic process	6.926469430158706e-10	*PRDM16,SCRT2,MEIS3,HIC1,DMRTB1,PRRX1,FBN1,TBX18,GLIS1,LHX4,GLI2,HIVEP3,NFATC4,EBF4,ALX4,ELF4,HES3,ZBTB7C,PAX7,HEYL,ZNF536,DPF3,GLIS2*
GO:0031326	regulation of cellular biosynthetic process	1.442197284276073e-9	*PRDM16,SCRT2,MEIS3,HIC1,DMRTB1,PRRX1,FBN1,TBX18,GLIS1,LHX4,GLI2,HIVEP3,NFATC4,EBF4,ALX4,ELF4,HES3,ZBTB7C,PAX7,HEYL,ZNF536,DPF3,GLIS2*
GO:0034654	nucleobase-containing compound biosynthetic process	1.4499208549135163e-9	*PRDM16,SCRT2,MEIS3,HIC1,DMRTB1,PRRX1,FBN1,TBX18,GLIS1,LHX4,GLI2,HIVEP3,NFATC4,EBF4,ALX4,ELF4,HES3,ZBTB7C,PAX7,HEYL,ZNF536,DPF3,GLIS2*
GO:1903508	positive regulation of nucleic acid-templated transcription	1.5306001574128243e-9	*PRDM16,MEIS3,PRRX1,GLIS1,LHX4,GLI2,HIVEP3,NFATC4,EBF4,ALX4,ELF4,HES3,ZBTB7C,PAX7,HEYL,DPF3,GLIS2*
GO:1902680	positive regulation of RNA biosynthetic process	1.546376306982263e-9	*PRDM16,MEIS3,PRRX1,GLIS1,LHX4,GLI2,HIVEP3,NFATC4,EBF4,ALX4,ELF4,HES3,ZBTB7C,PAX7,HEYL,DPF3,GLIS2*
GO:0018130	heterocycle biosynthetic process	2.0460009517873608e-9	*PRDM16,SCRT2,MEIS3,HIC1,DMRTB1,PRRX1,FBN1,TBX18,GLIS1,LHX4,GLI2,HIVEP3,NFATC4,EBF4,ALX4,ELF4,HES3,ZBTB7C,PAX7,HEYL,ZNF536,DPF3,GLIS2*
GO:0019438	aromatic compound biosynthetic process	2.1115059526759605e-9	*PRDM16,SCRT2,MEIS3,HIC1,DMRTB1,PRRX1,FBN1,TBX18,GLIS1,LHX4,GLI2,HIVEP3,NFATC4,EBF4,ALX4,ELF4,HES3,ZBTB7C,PAX7,HEYL,ZNF536,DPF3,GLIS2*
GO:0009889	regulation of biosynthetic process	2.1562820447308307e-9	*PRDM16,SCRT2,MEIS3,HIC1,DMRTB1,PRRX1,FBN1,TBX18,GLIS1,LHX4,GLI2,HIVEP3,NFATC4,EBF4,ALX4,ELF4,HES3,ZBTB7C,PAX7,HEYL,ZNF536,DPF3,GLIS2*
GO:0051254	positive regulation of RNA metabolic process	3.5058729795361598e-9	*PRDM16,MEIS3,PRRX1,GLIS1,LHX4,GLI2,HIVEP3,NFATC4,EBF4,ALX4,ELF4,HES3,ZBTB7C,PAX7,HEYL,DPF3,GLIS2*
GO:1901362	organic cyclic compound biosynthetic process	4.438335128940947e-9	*PRDM16,SCRT2,MEIS3,HIC1,DMRTB1,PRRX1,FBN1,TBX18,GLIS1,LHX4,GLI2,HIVEP3,NFATC4,EBF4,ALX4,ELF4,HES3,ZBTB7C,PAX7,HEYL,ZNF536,DPF3,GLIS2*
GO:0045944	positive regulation of transcription by RNA polymerase II	6.591543029834332e-9	*MEIS3,PRRX1,GLIS1,LHX4,GLI2,NFATC4,EBF4,ALX4,ELF4,HES3,ZBTB7C,PAX7,HEYL,DPF3,GLIS2*

GO:0010468	regulation of gene expression	8.580411834342911e-9	*PRDM16,SCRT2,MEIS3,HIC1,DMRTB1,PRRX1,FBN1,TBX18,GLIS1,LHX4,GLI2,HIVEP3,NFATC4,EBF4,ALX4,ELF4,HES3,ZBTB7C,PAX7,HEYL,ZNF536,DPF3,GLIS2*
GO:0016070	RNA metabolic process	1.1671159884933607e-8	*PRDM16,SCRT2,MEIS3,HIC1,DMRTB1,PRRX1,FBN1,TBX18,GLIS1,LHX4,GLI2,HIVEP3,NFATC4,EBF4,ALX4,ELF4,HES3,ZBTB7C,PAX7,HEYL,ZNF536,DPF3,GLIS2*
GO:0010557	positive regulation of macromolecule biosynthetic process	1.6993290967664777e-8	*PRDM16,MEIS3,PRRX1,GLIS1,LHX4,GLI2,HIVEP3,NFATC4,EBF4,ALX4,ELF4,HES3,ZBTB7C,PAX7,HEYL,DPF3,GLIS2*
GO:0045935	positive regulation of nucleobase-containing compound metabolic process	1.822534983944457e-8	*PRDM16,MEIS3,PRRX1,GLIS1,LHX4,GLI2,HIVEP3,NFATC4,EBF4,ALX4,ELF4,HES3,ZBTB7C,PAX7,HEYL,DPF3,GLIS2*
GO:0031328	positive regulation of cellular biosynthetic process	3.6680523863023566e-8	*PRDM16,MEIS3,PRRX1,GLIS1,LHX4,GLI2,HIVEP3,NFATC4,EBF4,ALX4,ELF4,HES3,ZBTB7C,PAX7,HEYL,DPF3,GLIS2*
GO:0010628	positive regulation of gene expression	4.0191758708781125e-8	*PRDM16,MEIS3,PRRX1,GLIS1,LHX4,GLI2,HIVEP3,NFATC4,EBF4,ALX4,ELF4,HES3,ZBTB7C,PAX7,HEYL,DPF3,GLIS2*
GO:0034645	cellular macromolecule biosynthetic process	4.293608704614357e-8	*PRDM16,SCRT2,MEIS3,HIC1,DMRTB1,PRRX1,FBN1,TBX18,GLIS1,LHX4,GLI2,HIVEP3,NFATC4,EBF4,ALX4,ELF4,HES3,ZBTB7C,PAX7,HEYL,ZNF536,DPF3,GLIS2*
GO:0009891	positive regulation of biosynthetic process	4.857139591618365e-8	*PRDM16,MEIS3,PRRX1,GLIS1,LHX4,GLI2,HIVEP3,NFATC4,EBF4,ALX4,ELF4,HES3,ZBTB7C,PAX7,HEYL,DPF3,GLIS2*
GO:0044271	cellular nitrogen compound biosynthetic process	5.1725162664371973e-8	*PRDM16,SCRT2,MEIS3,HIC1,DMRTB1,PRRX1,FBN1,TBX18,GLIS1,LHX4,GLI2,HIVEP3,NFATC4,EBF4,ALX4,ELF4,HES3,ZBTB7C,PAX7,HEYL,ZNF536,DPF3,GLIS2*
GO:0009059	macromolecule biosynthetic process	8.163921949552918e-8	*PRDM16,SCRT2,MEIS3,HIC1,DMRTB1,PRRX1,FBN1,TBX18,GLIS1,LHX4,GLI2,HIVEP3,NFATC4,EBF4,ALX4,ELF4,HES3,ZBTB7C,PAX7,HEYL,ZNF536,DPF3,GLIS2*
GO:0090304	nucleic acid metabolic process	1.326955611913901e-7	*PRDM16,SCRT2,MEIS3,HIC1,DMRTB1,PRRX1,FBN1,TBX18,GLIS1,LHX4,GLI2,HIVEP3,NFATC4,EBF4,ALX4,ELF4,HES3,ZBTB7C,PAX7,HEYL,ZNF536,DPF3,GLIS2*
GO:0010467	gene expression	5.891905186180785e-7	*PRDM16,SCRT2,MEIS3,HIC1,DMRTB1,PRRX1,FBN1,TBX18,GLIS1,LHX4,GLI2,HIVEP3,NFATC4,EBF4,ALX4,ELF4,HES3,ZBTB7C,PAX7,HEYL,ZNF536,DPF3,GLIS2*
GO:0006139	nucleobase-containing compound metabolic process	0.0000016510748449903486	*PRDM16,SCRT2,MEIS3,HIC1,DMRTB1,PRRX1,FBN1,TBX18,GLIS1,LHX4,GLI2,HIVEP3,NFATC4,EBF4,ALX4,ELF4,HES3,ZBTB7C,PAX7,HEYL,ZNF536,DPF3,GLIS2*
GO:0051171	regulation of nitrogen compound metabolic process	0.0000020759422534127013	*PRDM16,SCRT2,MEIS3,HIC1,DMRTB1,PRRX1,FBN1,TBX18,GLIS1,LHX4,GLI2,HIVEP3,NFATC4,EBF4,ALX4,ELF4,HES3,ZBTB7C,PAX7,HEYL,ZNF536,DPF3,GLIS2*
GO:0046483	heterocycle metabolic process	0.0000030465204730217683	*PRDM16,SCRT2,MEIS3,HIC1,DMRTB1,PRRX1,FBN1,TBX18,GLIS1,LHX4,GLI2,HIVEP3,NFATC4,EBF4,ALX4,ELF4,HES3,ZBTB7C,PAX7,HEYL,ZNF536,DPF3,GLIS2*
GO:0006725	cellular aromatic compound metabolic process	0.0000035473831077694912	*PRDM16,SCRT2,MEIS3,HIC1,DMRTB1,PRRX1,FBN1,TBX18,GLIS1,LHX4,GLI2,HIVEP3,NFATC4,EBF4,ALX4,ELF4,HES3,ZBTB7C,PAX7,HEYL,ZNF536,DPF3,GLIS2*
GO:0080090	regulation of primary metabolic process	0.0000038052020871308514	*PRDM16,SCRT2,MEIS3,HIC1,DMRTB1,PRRX1,FBN1,TBX18,GLIS1,LHX4,GLI2,HIVEP3,NFATC4,EBF4,ALX4,ELF4,HES3,ZBTB7C,PAX7,HEYL,ZNF536,DPF3,GLIS2*
GO:1903507	negative regulation of nucleic acid-templated transcription	0.000004426030004615167	*PRDM16,SCRT2,HIC1,PRRX1,TBX18,GLIS1,GLI2,NFATC4,HES3,HEYL,ZNF536,DPF3,GLIS2*
GO:1902679	negative regulation of RNA biosynthetic process	0.000004513191997994469	*PRDM16,SCRT2,HIC1,PRRX1,TBX18,GLIS1,GLI2,NFATC4,HES3,HEYL,ZNF536,DPF3,GLIS2*
GO:0044249	cellular biosynthetic process	0.00000477630767301253	*PRDM16,SCRT2,MEIS3,HIC1,DMRTB1,PRRX1,FBN1,TBX18,GLIS1,LHX4,GLI2,HIVEP3,NFATC4,EBF4,ALX4,ELF4,HES3,ZBTB7C,PAX7,HEYL,ZNF536,DPF3,GLIS2*
GO:0031323	regulation of cellular metabolic process	0.000006222217064430252	*PRDM16,SCRT2,MEIS3,HIC1,DMRTB1,PRRX1,FBN1,TBX18,GLIS1,LHX4,GLI2,HIVEP3,NFATC4,EBF4,ALX*

			4,ELF4,HES3,ZBTB7C,PAX7,HEYL,ZNF536,DPF3,GLIS2
GO:1901576	organic substance biosynthetic process	0.0000064269313270522756	*PRDM16,SCRT2,MEIS3,HIC1,DMRTB1,PRRX1,FBN1,TBX18,GLIS1,LHX4,GLI2,HIVEP3,NFATC4,EBF4,ALX4,ELF4,HES3,ZBTB7C,PAX7,HEYL,ZNF536,DPF3,GLIS2*
GO:1901360	organic cyclic compound metabolic process	0.000007604372911969553	*PRDM16,SCRT2,MEIS3,HIC1,DMRTB1,PRRX1,FBN1,TBX18,GLIS1,LHX4,GLI2,HIVEP3,NFATC4,EBF4,ALX4,ELF4,HES3,ZBTB7C,PAX7,HEYL,ZNF536,DPF3,GLIS2*
GO:0060255	regulation of macromolecule metabolic process	0.00000796464501454331	*PRDM16,SCRT2,MEIS3,HIC1,DMRTB1,PRRX1,FBN1,TBX18,GLIS1,LHX4,GLI2,HIVEP3,NFATC4,EBF4,ALX4,ELF4,HES3,ZBTB7C,PAX7,HEYL,ZNF536,DPF3,GLIS2*
GO:0009058	biosynthetic process	0.000008490374934770334	*PRDM16,SCRT2,MEIS3,HIC1,DMRTB1,PRRX1,FBN1,TBX18,GLIS1,LHX4,GLI2,HIVEP3,NFATC4,EBF4,ALX4,ELF4,HES3,ZBTB7C,PAX7,HEYL,ZNF536,DPF3,GLIS2*
GO:0051253	negative regulation of RNA metabolic process	0.000009416149930194954	*PRDM16,SCRT2,HIC1,PRRX1,TBX18,GLIS1,GLI2,NFATC4,HES3,HEYL,ZNF536,DPF3,GLIS2*
GO:0000122	negative regulation of transcription by RNA polymerase II	0.000011392105286079536	*PRDM16,SCRT2,HIC1,PRRX1,GLIS1,GLI2,NFATC4,HES3,HEYL,ZNF536,GLIS2*
GO:0034641	cellular nitrogen compound metabolic process	0.000023788043221584267	*PRDM16,SCRT2,MEIS3,HIC1,DMRTB1,PRRX1,FBN1,TBX18,GLIS1,LHX4,GLI2,HIVEP3,NFATC4,EBF4,ALX4,ELF4,HES3,ZBTB7C,PAX7,HEYL,ZNF536,DPF3,GLIS2*
GO:0045934	negative regulation of nucleobase-containing compound metabolic process	0.000028365773626508063	*PRDM16,SCRT2,HIC1,PRRX1,TBX18,GLIS1,GLI2,NFATC4,HES3,HEYL,ZNF536,DPF3,GLIS2*
GO:0045892	negative regulation of transcription, DNA-templated	0.00003817159575710786	*PRDM16,SCRT2,HIC1,PRRX1,GLIS1,GLI2,NFATC4,HES3,HEYL,ZNF536,DPF3,GLIS2*
GO:0019222	regulation of metabolic process	0.000043256319500171044	*PRDM16,SCRT2,MEIS3,HIC1,DMRTB1,PRRX1,FBN1,TBX18,GLIS1,LHX4,GLI2,HIVEP3,NFATC4,EBF4,ALX4,ELF4,HES3,ZBTB7C,PAX7,HEYL,ZNF536,DPF3,GLIS2*
GO:0010558	negative regulation of macromolecule biosynthetic process	0.00005349004868139387	*PRDM16,SCRT2,HIC1,PRRX1,TBX18,GLIS1,GLI2,NFATC4,HES3,HEYL,ZNF536,DPF3,GLIS2*
GO:0051173	positive regulation of nitrogen compound metabolic process	0.00007366711278222323	*PRDM16,MEIS3,PRRX1,GLIS1,LHX4,GLI2,HIVEP3,NFATC4,EBF4,ALX4,ELF4,HES3,ZBTB7C,PAX7,HEYL,DPF3,GLIS2*
GO:0031327	negative regulation of cellular biosynthetic process	0.00008715277826348149	*PRDM16,SCRT2,HIC1,PRRX1,TBX18,GLIS1,GLI2,NFATC4,HES3,HEYL,ZNF536,DPF3,GLIS2*
GO:0009890	negative regulation of biosynthetic process	0.00010332345484069701	*PRDM16,SCRT2,HIC1,PRRX1,TBX18,GLIS1,GLI2,NFATC4,HES3,HEYL,ZNF536,DPF3,GLIS2*
GO:0031325	positive regulation of cellular metabolic process	0.00012898462093474485	*PRDM16,MEIS3,PRRX1,GLIS1,LHX4,GLI2,HIVEP3,NFATC4,EBF4,ALX4,ELF4,HES3,ZBTB7C,PAX7,HEYL,DPF3,GLIS2*
GO:0010604	positive regulation of macromolecule metabolic process	0.00015564996393351493	*PRDM16,MEIS3,PRRX1,GLIS1,LHX4,GLI2,HIVEP3,NFATC4,EBF4,ALX4,ELF4,HES3,ZBTB7C,PAX7,HEYL,DPF3,GLIS2*
GO:2000113	negative regulation of cellular macromolecule biosynthetic process	0.0003332442843287556	*PRDM16,SCRT2,HIC1,PRRX1,GLIS1,GLI2,NFATC4,HES3,HEYL,ZNF536,DPF3,GLIS2*
GO:0009893	positive regulation of metabolic process	0.00048241291702045603	*PRDM16,MEIS3,PRRX1,GLIS1,LHX4,GLI2,HIVEP3,NFATC4,EBF4,ALX4,ELF4,HES3,ZBTB7C,PAX7,HEYL,DPF3,GLIS2*
GO:0032502	developmental process	0.0012623921674292649	*PRDM16,SCRT2,HIC1,PRRX1,FBN1,TBX18,LHX4,GLI2,HIVEP3,NFATC4,EBF4,ALX4,ELF4,HES3,ZBTB7C,PAX7,HEYL,GRM6,ZNF536,DPF3,GLIS2*
GO:0044260	cellular macromolecule metabolic process	0.004171357855575304	*PRDM16,SCRT2,MEIS3,HIC1,DMRTB1,PRRX1,FBN1,TBX18,GLIS1,LHX4,GLI2,HIVEP3,NFATC4,EBF4,ALX4,ELF4,HES3,ZBTB7C,PAX7,HEYL,ZNF536,DPF3,GLIS2*
GO:0048523	negative regulation of cellular process	0.005357241965811612	*PRDM16,SCRT2,MEIS3,HIC1,PRRX1,FBN1,TBX18,GLIS1,LHX4,GLI2,NFATC4,HES3,ZBTB7C,PAX7,HEYL,ZNF536,DPF3,GLIS2*
GO:0007275	multicellular organism development	0.00552760629142322	*PRDM16,SCRT2,HIC1,PRRX1,FBN1,TBX18,LHX4,GLI2,HIVEP3,NFATC4,EBF4,ALX4,HES3,PAX7,HEYL,GRM6,ZNF536,DPF3,GLIS2*
GO:0043170	macromolecule metabolic process	0.006920674025020102	*ADAMTS17,PRDM16,SCRT2,MEIS3,HIC1,DMRTB1,PRRX1,FBN1,TBX18,GLIS1,LHX4,GLI2,HIVEP3,NFATC4,EBF4,ALX4,ELF4,HES3,ZBTB7C,PAX7,HEYL,ZNF536,DPF3,GLIS2*
GO:0009968	negative regulation of signal	0.007029022775027719	*PRDM16,SCRT2,MEIS3,HIC1,FBN1,TBX18,NFATC4,*

	transduction		*HEYL,ZNF536,GLIS2*
GO:0010629	negative regulation of gene expression	0.00824922522890636	*PRDM16,SCRT2,HIC1,PRRX1,GLIS1,GLI2,NFATC4,HES3,HEYL,ZNF536,DPF3,GLIS2*
GO:0051172	negative regulation of nitrogen compound metabolic process	0.009553645532495566	*PRDM16,SCRT2,HIC1,PRRX1,TBX18,GLIS1,GLI2,NFATC4,HES3,HEYL,ZNF536,DPF3,GLIS2*
GO:0010648	negative regulation of cell communication	0.014395035624266382	*PRDM16,SCRT2,MEIS3,HIC1,FBN1,TBX18,NFATC4,HEYL,ZNF536,GLIS2*
GO:0023057	negative regulation of signaling	0.014775101616241095	*PRDM16,SCRT2,MEIS3,HIC1,FBN1,TBX18,NFATC4,HEYL,ZNF536,GLIS2*
GO:0031324	negative regulation of cellular metabolic process	0.0195907285827505	*PRDM16,SCRT2,HIC1,PRRX1,TBX18,GLIS1,GLI2,NFATC4,HES3,HEYL,ZNF536,DPF3,GLIS2*
GO:0048856	anatomical structure development	0.022443281983878204	*PRDM16,SCRT2,HIC1,PRRX1,FBN1,TBX18,LHX4,GLI2,HIVEP3,NFATC4,EBF4,ALX4,HES3,PAX7,HEYL,GRM6,ZNF536,DPF3,GLIS2*
GO:0045595	regulation of cell differentiation	0.024063479040356613	*PRDM16,SCRT2,PRRX1,FBN1,GLI2,NFATC4,HES3,ZBTB7C,PAX7,HEYL,ZNF536*
GO:0006807	nitrogen compound metabolic process	0.03029468182054811	*ADAMTS17,PRDM16,SCRT2,MEIS3,HIC1,DMRTB1,PRRX1,FBN1,TBX18,GLIS1,LHX4,GLI2,HIVEP3,NFATC4,EBF4,ALX4,ELF4,HES3,ZBTB7C,PAX7,HEYL,ZNF536,DPF3,GLIS2*
GO:0048522	positive regulation of cellular process	0.031169395234697667	*PRDM16,MEIS3,HIC1,PRRX1,GLIS1,LHX4,GLI2,HIVEP3,NFATC4,EBF4,ALX4,ELF4,HES3,ZBTB7C,PAX7,HEYL,DPF3,GLIS2*
GO:0048519	negative regulation of biological process	0.03733585974290125	*PRDM16,SCRT2,MEIS3,HIC1,PRRX1,FBN1,TBX18,GLIS1,LHX4,GLI2,NFATC4,HES3,ZBTB7C,PAX7,HEYL,ZNF536,DPF3,GLIS2*
GO:0048518	positive regulation of biological process	0.04169304757910125	*PRDM16,MEIS3,HIC1,PRRX1,GLIS1,LHX4,GLI2,HIVEP3,NFATC4,EBF4,ALX4,ELF4,HES3,ZBTB7C,PAX7,HEYL,GRM6,DPF3,GLIS2*
GO:0007399	nervous system development	0.04300712142418596	*PRDM16,SCRT2,PRRX1,LHX4,GLI2,NFATC4,HES3,PAX7,HEYL,ZNF536,DPF3,GLIS2*
GO:0048731	system development	0.0432020590179772	*PRDM16,SCRT2,PRRX1,FBN1,TBX18,LHX4,GLI2,HIVEP3,NFATC4,ALX4,HES3,PAX7,HEYL,GRM6,ZNF536,DPF3,GLIS2*
GO:0021532	neural tube patterning	0.044736223506683194	*GLI2,HES3,PAX7*
GO:0060284	regulation of cell development	0.04505117736541354	*SCRT2,PRRX1,FBN1,GLI2,NFATC4,HES3,HEYL,ZNF536*

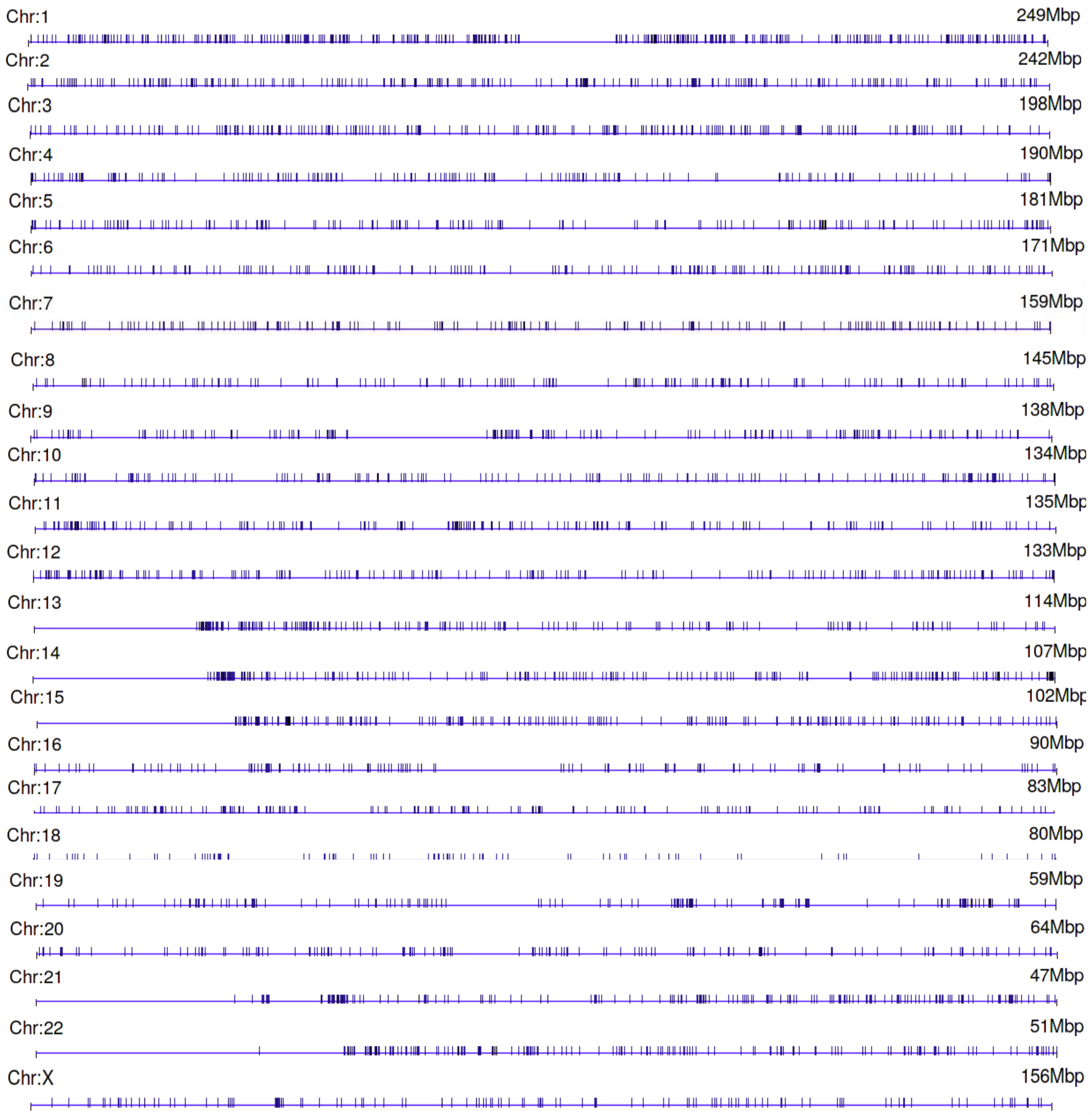

Figure S1. The distribution of 4920 selected rDNA-contacting genes on human chromosomes. The search was performed using the http://bioinformatics.sdstate.edu/go/ resource. All chromosomes possess rDNA-contacting genes. Some gaps are observed, which correspond to regions that are not included in the sequenced portion of the human genome, including centromere regions, the small arms of acrocentric chromosomes, and the gene desert areas.
